# Bioinformatics approaches for classification and investigation of the evolution of the Na/K-ATPase alpha-subunit

**DOI:** 10.1186/s12862-022-02071-0

**Published:** 2022-10-26

**Authors:** Marzieh Shahnazari, Zahra Zakipour, Hooman Razi, Ali Moghadam, Abbas Alemzadeh

**Affiliations:** 1 Department of Plant Production and Genetics, School of Agriculture, Shiraz University, Shiraz, Iran; 2 Institute of Biotechnology, Shiraz University, Shiraz, Iran

**Keywords:** α-subunit of Na, K-ATPase, Evolution, Decision tree, Phylogenetic tree

## Abstract

**Background:**

Na,K-ATPase is a key protein in maintaining membrane potential that has numerous additional cellular functions. Its catalytic subunit (α), found in a wide range of organisms from prokaryotes to complex eukaryote. Several studies have been done to identify the functions as well as determining the evolutionary relationships of the α-subunit. However, a survey of a larger collection of protein sequences according to sequences similarity and their attributes is very important in revealing deeper evolutionary relationships and identifying specific amino acid differences among evolutionary groups that may have a functional role.

**Results:**

In this study, 753 protein sequences using phylogenetic tree classification resulted in four groups: prokaryotes (I), fungi and various kinds of Protista and some invertebrates (II), the main group of invertebrates (III), and vertebrates (IV) that was consisted with species tree. The percent of sequences that acquired a specific motif for the α/β subunit assembly increased from group I to group IV. The vertebrate sequences were divided into four groups according to isoforms with each group conforming to the evolutionary path of vertebrates from fish to tetrapods. Data mining was used to identify the most effective attributes in classification of sequences. Using 1252 attributes extracted from the sequences, the decision tree classified them in five groups: Protista, prokaryotes, fungi, invertebrates and vertebrates. Also, vertebrates were divided into four subgroups (isoforms). Generally, the count of different dipeptides and amino acid ratios were the most significant attributes for grouping. Using alignment of sequences identified the effective position of the respective dipeptides in the separation of the groups. So that ^208^GC is apparently involved in the separation of vertebrates from the four other organism groups, and ^41^DH, ^431^FK, and ^451^KC were involved in separation vertebrate isoform types.

**Conclusion:**

The application of phylogenetic and decision tree analysis for Na,K-ATPase, provides a better understanding of the evolutionary changes according to the amino acid sequence and its related properties that could lead to the identification of effective attributes in the separation of sequences in different groups of phylogenetic tree. In this study, key evolution-related dipeptides are identified which can guide future experimental studies.

**Supplementary Information:**

The online version contains supplementary material available at 10.1186/s12862-022-02071-0.

## Background

The p-type ATPase pumps, as primary membrane transporters using ATP hydrolysis, accomplish translocation of a broad range of specificities for small cations and also phospholipids across the respective membranes [1]. They have been found in all domains of life and are divided into five major families (P1–P5) according to specificity for substrate and not on the basis of evolutionary relationship. Some of these families are divided into two or more subfamilies [1, 2]. The P-Type II ATPases with specificity for Ca^2+^, K^+^ and Na^+^ are divided into five subfamilies including A, B, C, D, and E; which are also known as SERCA, PMCA, NK/HK, ENA, and ACU, respectively [2, 3]. The P-type ATPases are widely involved in different basic cellular processes, by maintenance of the proper gradients for essential ions. P-Type II pumps have vital importance in many cellular activities, including regulation of secondary active transporters, the cellular signaling system, and Ca^+2^ compartmentalization [4].

The Na, K-ATPase pump, NKA, is a P-type ATPase membrane enzyme which is responsible for the creation and maintenance of an electrochemical gradient through the efflux of three sodium ions and influx of two potassium ions across the plasma membrane. This pump functions as a driving force for the secondary active transport of molecules and regulation of the cell volume, pH homeostasis and signal transducer [5–7]. NKA is a heterodimer pump with two or three subunits in eukaryotes [8], which are designated α, β and γ [9]. These subunits have a high degree of conservation across species [7]. The α-subunit is a membrane protein with ten membrane-spanning helices and two large intracellular loops and C and N-terminal cytoplasmic tails. This subunit has four isoforms, α1, α2, α3 and α4, in vertebrates. The structural basis of the difference between them, have occurred in distinct domains rather than random changes throughout the sequence [10]. The most variable parts of α-subunit are N-terminus as isoform specificity in the rate of K^+^ de-occlusion, the extracellular ouabain binding site between transmembrane segments 1 and 2, and an 11 amino acid sequence that is an isoform-specific region in large central loop [7, 11]. The most similarity region among α isoforms is related to transmembrane hydrophobic regions, the cytoplasmic mid-region around the phosphorylation site (Asp369), and the C-terminus [7]. Numerous studies have been done to identify conserved motifs and amino acids in similar or different regions and their role in ion transport mechanism and other properties of the enzyme obtained during evolution [12–16].

Protein analysis of different taxonomic groups can provide information on their evolution and division. The primary structure of a protein determines next structures and its function and evolutionary characteristics [17–19]. There are many important amino acid attributes including the physicochemical properties of amino acids, their compositions and other sequence descriptors which have been widely applied in computational biology [18, 19].

Due to an exponential growth of biological data, the use of bioinformatics tools is very useful [20]. Transformation of existing or extracted data from sequences into clear and comprehensible information by bioinformatics tools and using classification and prediction techniques may be a way to better understand the differences and similarities between different isoforms of a protein or the same isoforms between the species [21–23]. Different classification techniques or algorithms have been used by different researchers to classify and predict proteins based on their sequences or other information of amino acids sequences [23–25].

In biology, phylogenetic analysis is a common and powerful sequence-based technique with the purpose of discovering the evolutionary history of organisms and their relationships. It also can depict a hypothesis about the evolutionary ancestry of a set of genes, protein families, species, or other taxa [26, 27]. The phylogenetic analysis of enzyme sequences applies as a strong method for the organization and interpretation of the taxa [28]. This method performs grouping by alignment and finding homology among sequences and provides clear and valuable information about origins and possible functions of the proteins [27–30].

In addition to determining evolutionary changes of proteins at the sequence level, amino acid sequence attributes can also be useful for this purpose [22, 23]. Machine learning techniques can disclose the underlying mechanism of protein function using diverse amino acid properties and discovering the rules among them [31]. Classification methods were used to determine which attributes should be included in the models to find the pattern of the relationship between the attributes and determining which attributes play important roles in the prediction of unknown proteins and even cell location of protein [32, 33].

In this study, we used two important methods of clustering and classification, phylogenetic and decision tree, to gain a comprehensive understanding of NKA protein relationships among different taxonomic groups of organisms and types of vertebrate’ isoforms. Also, we tried to determine the most important amino acid attributes involved in the classification of sequences based on decision tree.

## Results

### Phylogenetic tree-clustering analysis

#### Phylogenetic relationships of Na, K-ATPases among different taxonomic groups

In this study, a phylogenetic analysis was done to show the relationship among taxonomic groups for α-NKA proteins using 753 sequences that belonged to five groups including vertebrates (323 sequences), invertebrates (275 seq.), fungi (62 seq.), Protista (49 seq.) and prokaryotes (44 seq.).

**Fig. 1:**
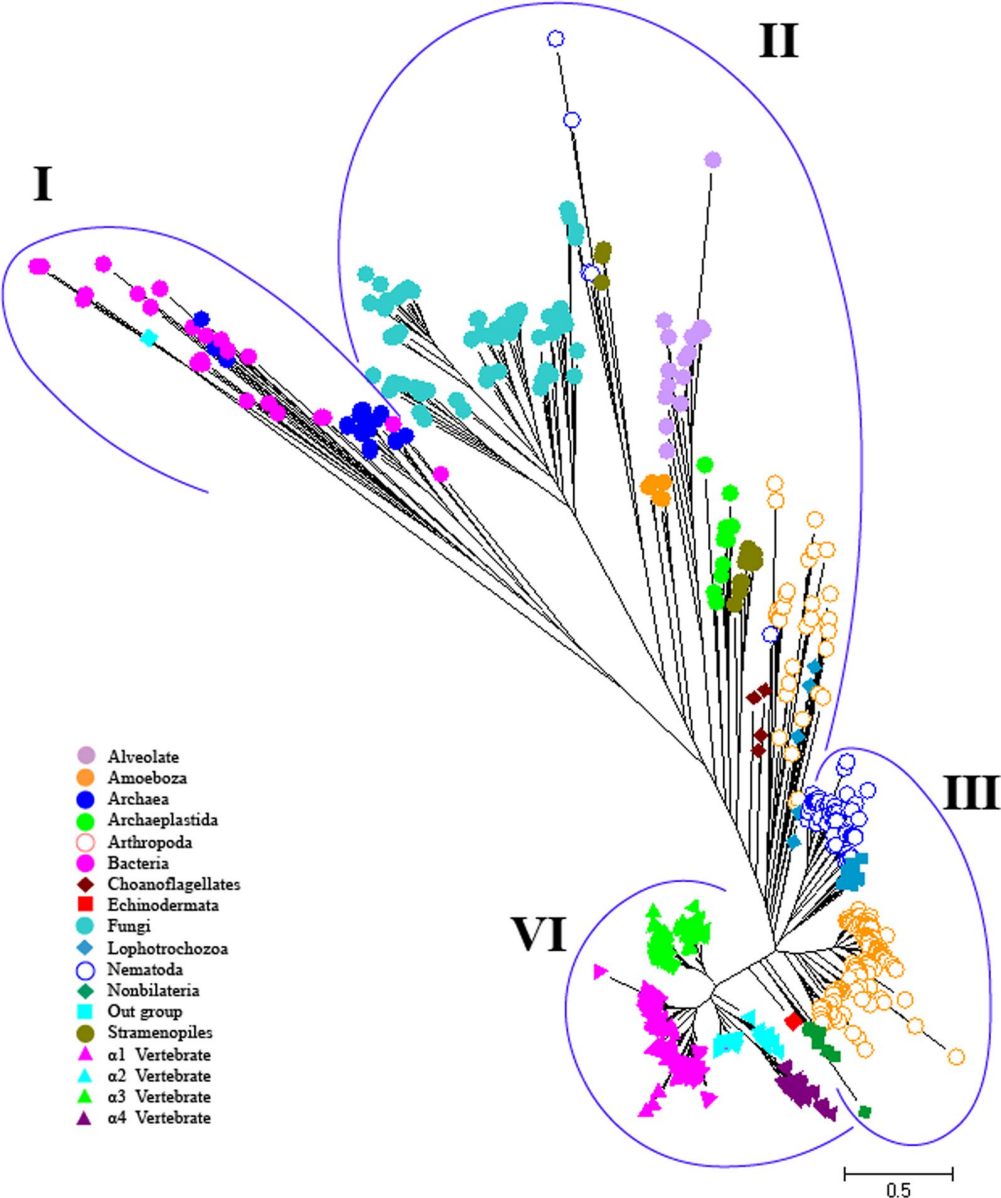
The phylogenetic tree of Na,K-ATPases for all organisms. Different symbols and colors were used to distinguish organisms and the type of isoforms. The scale indicates the number of amino acid substitutions per site

According to the topology of phylogenetic tree and a quantitative analysis for clustering within the phylogeny (Additional file 1: Fig S1), we classified all proteins in four groups containing prokaryotes (bacteria and archaea) (I), fungi and various kinds of Protista (Opisthokonta, Alveolate, Amoebozoa, Archaeplastida and Stramenopiles) and some invertebrates (II), the main group of invertebrates (III), and vertebrates (IV) (Fig. 1; Additional file 1: Fig S1). All archaea and bacteria sequences were clustered in the same clade. All sequences in group I lack the motif which is required for α/β subunit assembly, Ser-Tyr-Gly-Gln/Glu [34], suggesting that these subunits exist by themselves.

**Fig. 2:**
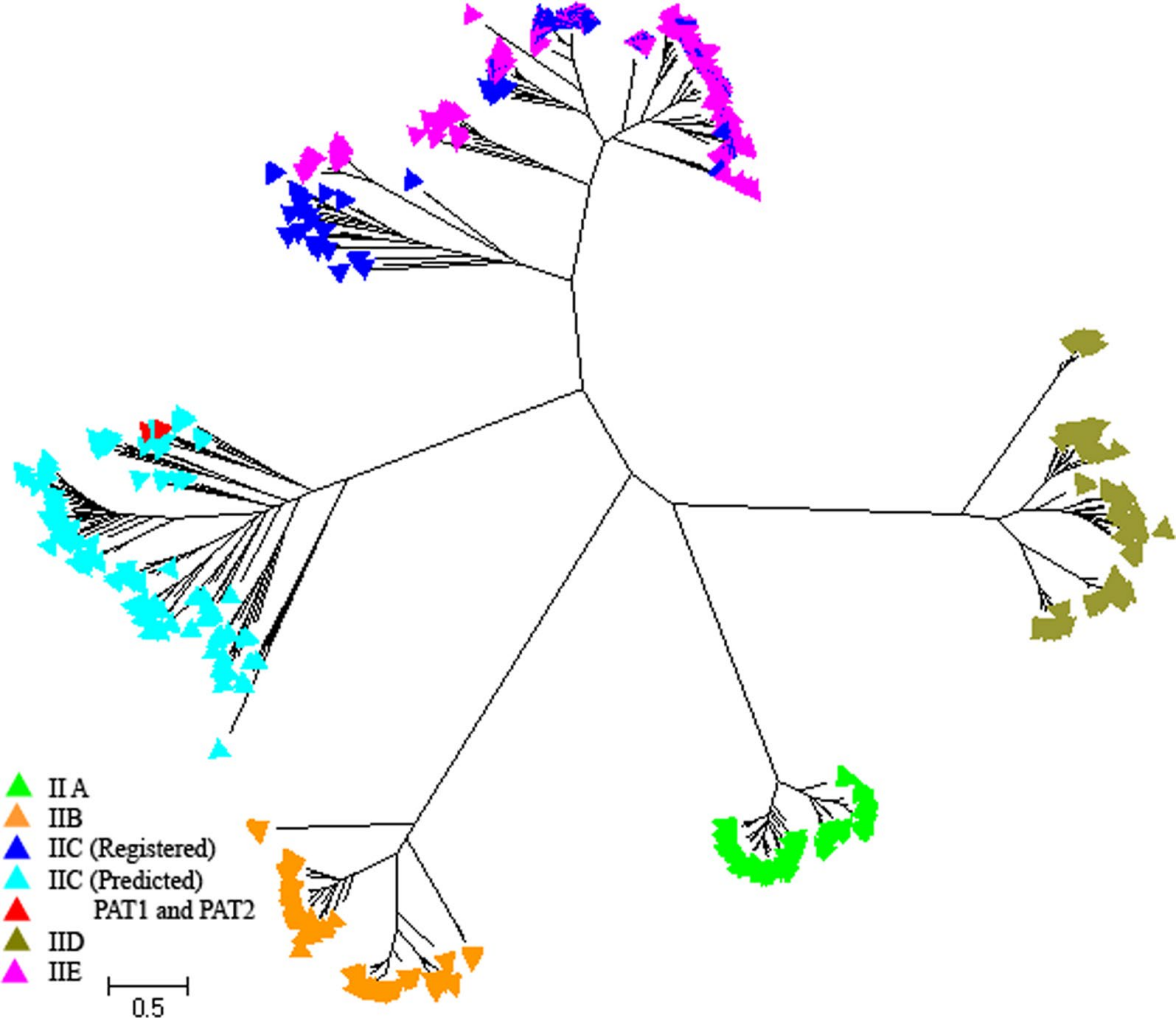
The phylogenetic tree of different pumps in fungal sequences. Different colors were used to distinguish different kind of pumps

The presence of NKA in the fungi *Blastocladiella emersonii* was confirmed using bioinformatics, molecular and biochemical studies [35–37]. The structure of Be PAT1 and Be PAT2, isolated from *Blastocladiella emersonii*, were studied very well and specific motifs in their sequences were determined [35, 36]. We used these proteins as an indicator to distinguish NKA protein from P-Type IIE ATPases in a fungal phylogenetic tree with 680 sequences belonging to different groups of P-Type II ATPase (Fig. 2; Additional file 1: Fig S2). In this study, in addition to the main groups of fungi, NKA has been found in Chytridiomycota fungi that are the basal fungal taxa [38]. There were some NKA sequences from different phyla of fungi including Blastocladiomycota, Chytridiomycota, Mucoromycota, Zygomycota, Ascomycota and Basidiomycota. All of these fungal sequences were classified in group II (Fig. 1).

As the results show in addition to fungi, all sequences from Alveolate, Archaeplastid, Amobozoa, and Stramenopile were also placed in group II so that the sequences belonging to each of them were completely next to each other, only three sequences of Stramenopile with six sequences of nematode placed near fungi (Fig. 1). These nematode sequences with special characters were also belonged to *Caenorhabditis elegans*, Ce2C3, Ce2C4, and Ce2C5, and Toxocara canis, Tc1, Tc2, and Tc3, without the presence of any consensus sequence for α/β subunit assembly (SYGQ motif). These species have at least one sequence in the main group of invertebrates (group III) that possess the required motif for α/β subunit assembly. In general, 15.27% of invertebrate sequences were in group II and 84.73% in group III.

None of sequences from fungi and Protista except in Choanoflagellate, have the subunit-assembly motif. The presence of some Oomycetes species and slim mold (*Dictyostelium discoideum* and *Cavenderia fasciculate*) in this group is not surprising since they are a lineage of fungus-like eukaryotic microorganisms [39].

The position of Choanoflagellate sequences is important because it has been considered as the closest living relative to animals [40] and can help us to figure out the origin of α-NKA in animals. We have found four sequences from two species of Choanoflagellate, *Salpingoeca rosetta* and *Monosiga brevicollis*, in group II next to animals (arthropoda) (Fig. 1). In addition, there were some sequences from green, red and brown algae placed in the group II, but no sequences from higher plants. Although the higher plants are made up of the green algae, but until now, no report has indicated a presence NKA in higher plants.

Most invertebrates were in group III so the nematodes were placed next to each other along with sequences of Lophotocozoa as well as the arthropoda altogether. Then, the sequences belonging to Nonbilateria were placed separately next to them. A sequence of Echinodermata was placed in this group, close to the vertebrates. Vertebrates were well separated in group IV from others. The sequences were divided into four isoform groups and interestingly sequences of α2 and α4-isoforms were placed on one branch but separately. Interestingly, we found that the percent of sequences which have the specific motif for the α/β subunit assembly (the presence of consensus sequence or with at most one different amino acid (but similar according pairwise alignment) from SYGQ motif)) increased from group I to group IV (group I (0%), group II (22.22%), group III (87.66%), and group IV (100%)).

#### Phylogenetic tree of life for different taxonomic groups using ssu rRNA

**Fig. 3:**
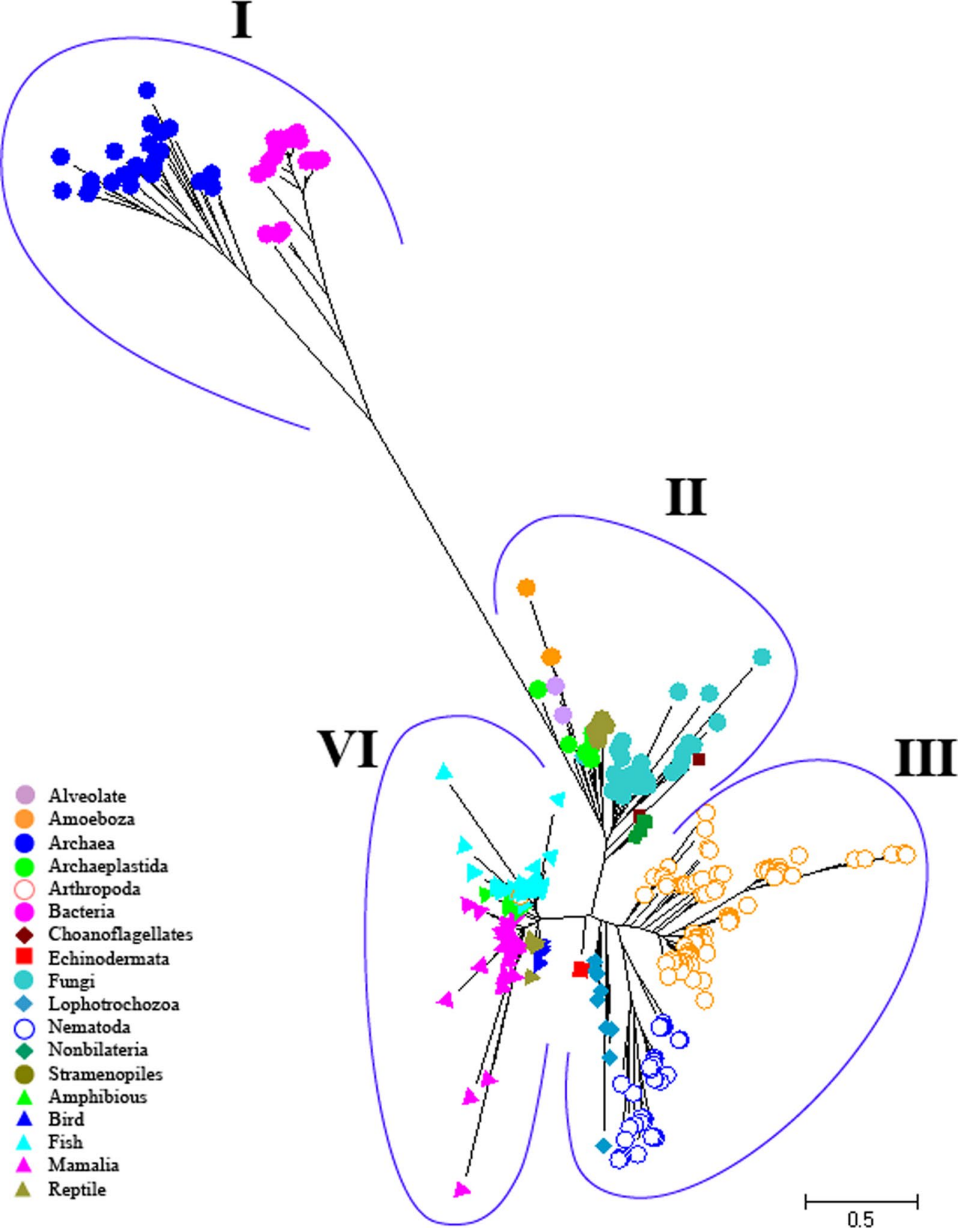
The phylogenetic tree for 378 sequences of ssu Rrna (16S/18S rRNA) from various organisms of three life domains which were used for construct NAK phylogenetic tree. Different symbols and colors were used to distinguish organisms and the type of isoforms. The scale indicates the number of amino acid substitutions per site

For a more in-depth study of the evolution of NKA, the phylogenetic tree of life was drawn using 378 ssu rRNA (16S/18S rRNA) sequences belonging to 375 species from different taxonomic groups studied for NKA. According to the Fig. 3 and Additional file 1: Fig. S3 the tree is clearly divided into four groups containing prokaryotes (bacteria and archaea) (I), fungi and various kinds of Protista and some invertebrates (Nonbilateria) (II), the main group of invertebrates (III), and vertebrates (IV). These results were largely consistent with the phylogeny tree of NKA among different taxonomic groups and confirm the accuracy of the grouping performed for it. Invertebrates were all completely in-group III, which includes arthropod, nematodes and Lophotrocozoa. In addition, the vertebrates were also completely separated from fish to type of tetrapods.

#### Phylogenetic relationships of Na, K-ATPases among vertebrate

**Fig. 4:**
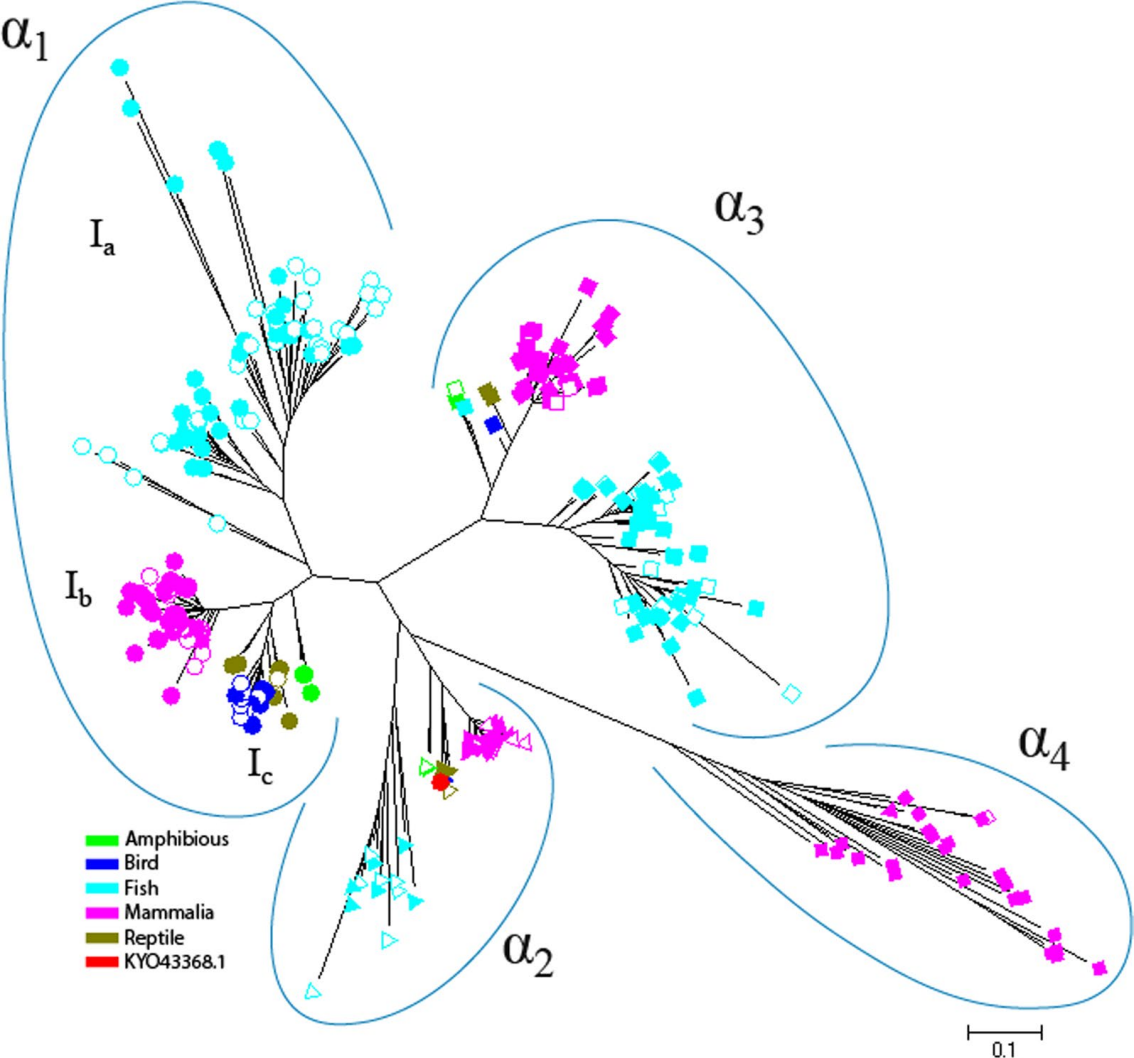
The phylogenetic tree of Na,K-ATPase for vertebrate organisms. Different symbols and colors were used to distinguish organisms and the type of isoforms. The hollow shapes indicated the isoform was predicted by the phylogenetic tree in this study. Circle, triangle, square and diamond shapes were used to show α1, α2, α3 and α4, respectively. The scale indicates the number of amino acid substitutions per site

Because of the complete separation of vertebrate α-NKA from the others in Fig. 1, a phylogenetic analysis was performed to investigate the relationship among various vertebrate isoforms (Fig. 4; Additional file 1: Fig S4). Of the 323 sequences that belong to vertebrates, 231 of them had previously been identified which isoform they belonged to (in database), and 92 sequences were specified as α1, α2, α3, or α4 based on their placement in the phylogenetic tree relative to sequences of known isoform. Also, we found a sequence (accession number: KYO43368.1) from *Alligator mississippiensis*, that was previously classified as α1 (in database), but in the phylogenetic tree, it was placed next to the sequences in α2 clade. Sequence alignment for this sequence and a set of sequences belonging to each of four isoforms indicated the existence of the α2 specific motif that had been identified as consensus sequence HERED in previous studies [11, 41]. Then this protein should be considered as α2 (Additional file 1: Fig. S5). The vertebrate’s sequences have the required motif for α/β subunit assembly that indicates they can assemble with β subunit.

The results showed that all isoforms, α1, α2, α3, and α4, were completely separated in vertebrates. We also figured out that α4 isoform, which is found only in mammals, was clustered as a separated group (Fig. 4). Interestingly, we found in each isoform, the isoforms belonging to fishes and mammalians were clearly separated from those of other vertebrates. There was only one sequence related to the lungfish, *Protopterus annectens*, which was placed between mammalian sequences and other vertebrate sequences in α3 subunit cluster (Fig. 4).

**Fig. 5:**
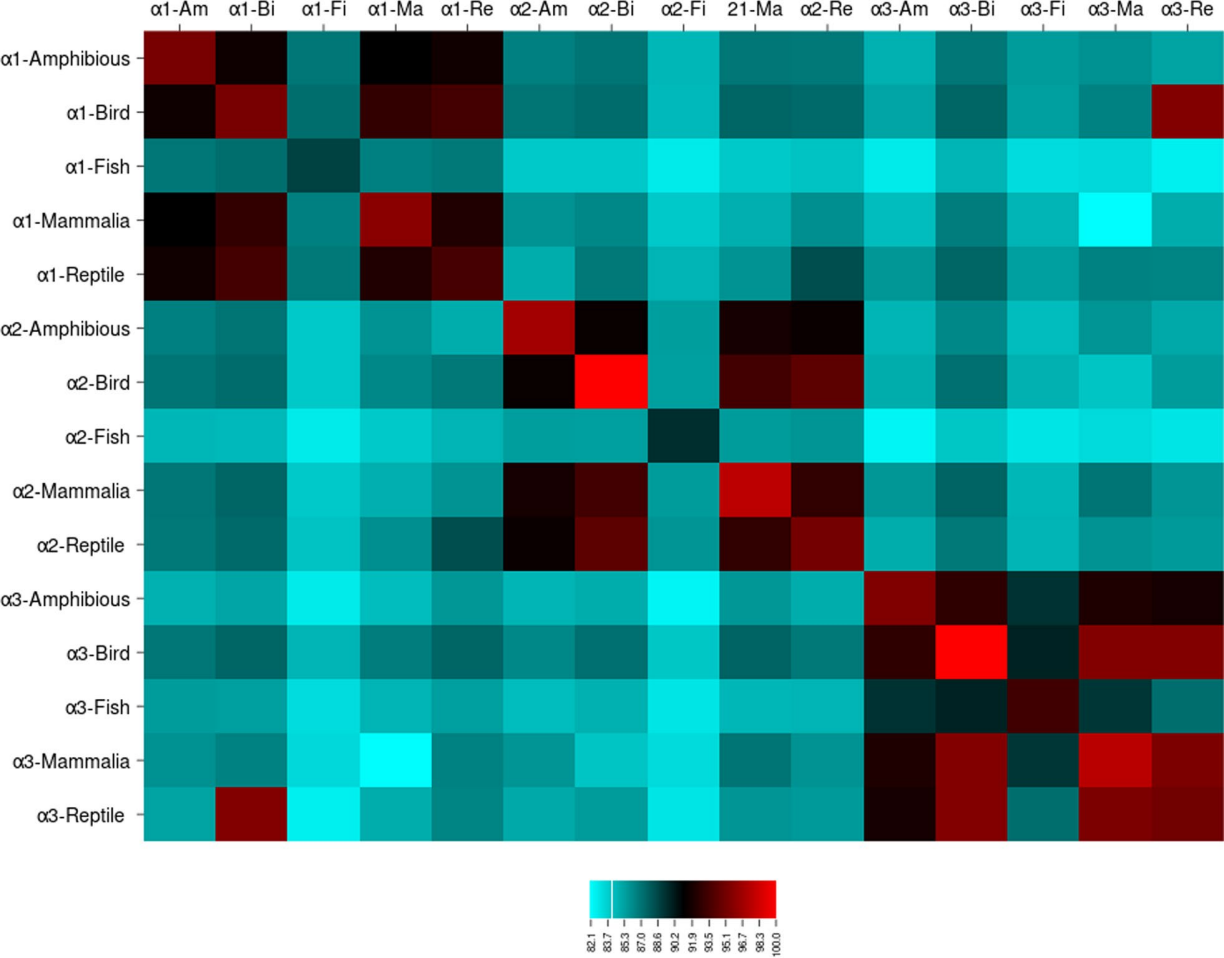
The changes of similarity rate of different isoforms within and among different groups of vertebrates. Am: amphibious, Fi: fish, Bi: bird, Ma: Mammalia, Re: reptile

In vertebrates the α1 isoform fell into three major groups (Fig. 4): the first group (Ia) α-NKA is only from fishes, the second more inclusive group (Ib) is only from mammalian species, and the third one (Ic) comes from amphibians, reptiles and birds. In fact, fish are completely separated from the tetrapods, which was clearly observed in the α3 isoform and most sequences of α2 isoform. As shown in the phylogenetic tree, similarity rate of different isoforms among different groups of vertebrates is greater than to different isoforms in a group (Fig. 5 and Additional file 1: Table S1).

According Fig. 5, the similarity rate between fish and tetrapod for each isoform is less than the similarity rate between tetrapod with each other. In general, the similarity rate between the different organisms of vertebrate for the α3 isoform is greater than the α1 and α2 isoforms. The high similarity between the α1 and α3 isoforms for bird and reptile respectively, is due to the few number of sequences to be investigated in this case (Fig. 5). Among the types of isoforms, the α2 and α4 isoforms were located in a sister clade with 99% replication (Fig. 4). Interestingly, the α2 isoforms of fish sequences were placed next to the α4 isoforms of mammals. Among the vertebrate α-NKAs, the Chondrichthyes presented four α1 sequences, *Callorhinchus milii*, *Himantura signifer*, *Squalus acanthias*, and *Tetronarce californica* that fell into a separate group from other fish α1 sequences (Fig. 4).

### Decision tree-classification analysis

#### Decision tree for α-NKA in different organism groups

To investigate the role of extracted attributes from primary structure of α-NKA protein in different organism groups, the decision tree analysis was done for five groups of prokaryote, Protista, fungi, invertebrates and vertebrates. after extraction of 1252 attributes from6 753 sequences, data cleansing was done to increase the ability to process attributes that led to a reduction in attributes to 660. The 10 datasets created using weighting algorithms. The PCA and info gain ratio dataset had a minimum and maximum of attributes (9 and 22 attributes) (Additional file 1: Table. S2). Using ten datasets along with FCD dataset, 176 trees were created with minimum and maximum of performance 45.54% and 99.33%, respectively (Table 1). To compare and determine the best and most efficient model to construct a decision tree, the percentage of performance of each model was used. The best performance was related to the Decision Tree model with information gain criteria when run on FCD dataset.

**Table 1:** The percentage of accuracy for each model with different criterion for different isoforms of α-NKA in vertebrates

Tree model	Decision tree				Random Forest			
Criterion	Gain ratio	Information gain	Gini index	Accuracy	Gain ratio	Information gain	Gini index	Accuracy
FCDS	50.00	99.57	98.71	98.28	95.69	97.84	96.98	97.41
Chi square	50.00	98.71	98.28	98.28	98.28	99.57	97.84	97.41
Info gain	99.14	98.71	97.41	97.84	98.71	98.71	99.14	98.71
Deviation	93.10	97.84	96.55	96.12	94.83	94.83	93.97	93.1
Gini index	99.14	98.28	97.41	97.84	99.14	99.14	98.71	98.71
Info gain ratio	50.00	99.14	98.28	97.41	98.28	98.28	98.71	97.84
PCA	50.00	98.71	98.71	98.71	94.40	97.41	95.26	96.12
Relief	98.28	97.84	97.84	97.84	96.12	96.98	96.55	95.26
Rule	50.00	98.28	98.28	98.28	98.28	98.28	97.41	96.12
Uncertainty	99.14	98.71	98.28	96.98	98.28	98.71	98.71	98.71
SVM	98.28	98.28	98.28	98.28	98.71	97.84	96.98	96.55

Tree model	Decision Stumpt				Random Tree			
Criterion	Gain ratio	Information gain	Gini index	Accuracy	Gain ratio	Information gain	Gini index	Accuracy
FCDS	50.00	73.28	71.98	72.41	72.41	86.21	89.22	50.43
Chi square	50.00	71.98	71.98	71.98	75.86	91.81	91.81	74.57
Info gain	49.57	73.28	71.98	72.41	81.47	90.52	90.52	82.33
Deviation	50.00	50.00	65.95	49.14	40.09	88.36	88.79	45.69
Gini index	49.57	73.28	71.98	72.41	92.67	92.67	93.10	74.57
Info gain ratio	50.00	73.28	71.98	72.41	49.57	78.88	91.81	49.57
PCA	50.00	70.69	70.69	70.69	57.33	66.81	70.26	51.72
Relief	72.41	72.41	72.41	65.95	89.66	78.02	82.76	83.62
Rule	50.00	70.69	70.69	70.69	58.05	63.36	85.78	51.29
Uncertainty	49.57	73.28	71.98	72.41	97.83	95.26	95.26	93.10
SVM	73.28	73.28	71.98	72.41	84.91	88.36	83.62	85.34

**Fig. 6:**
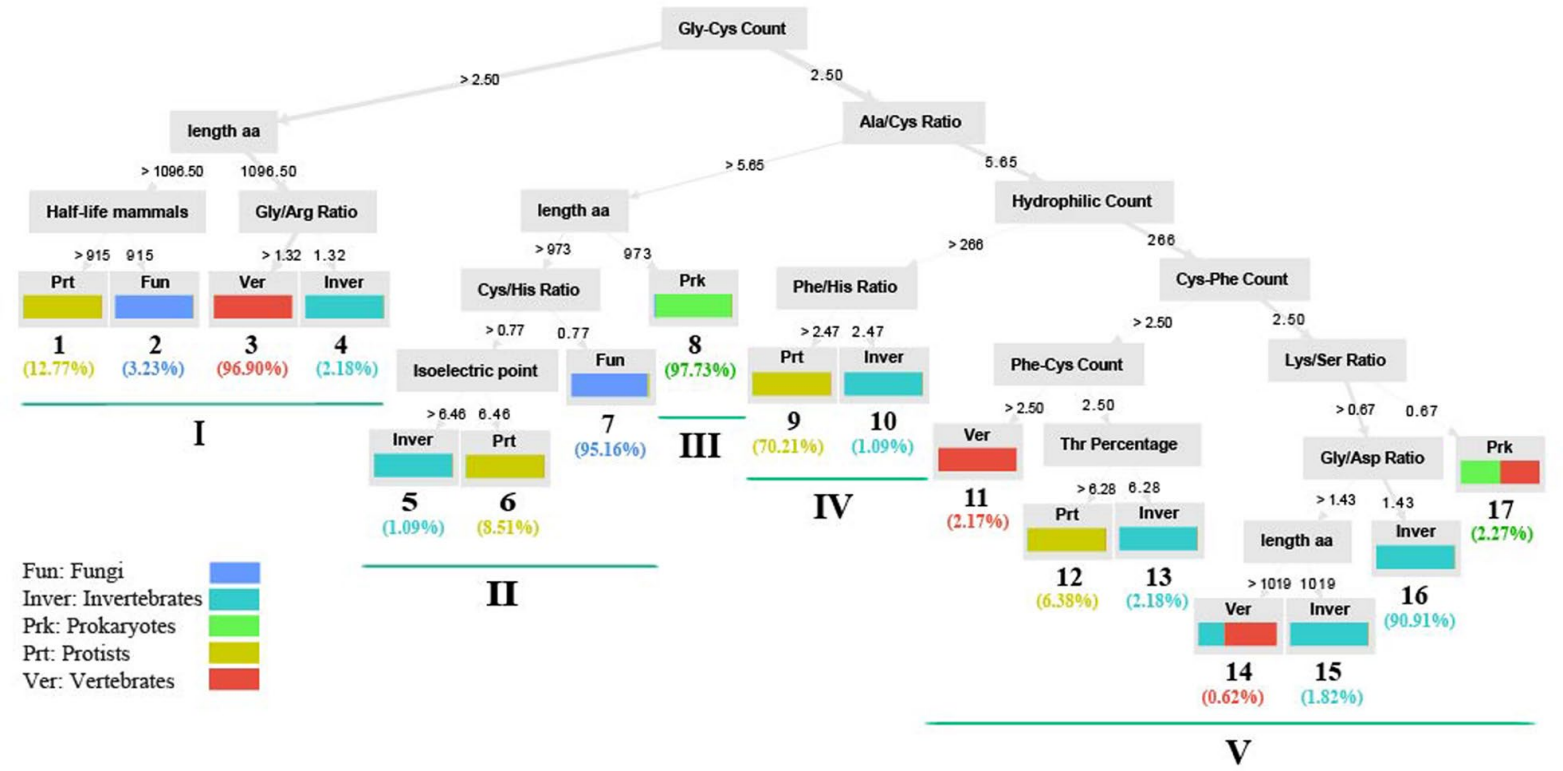
The Decision tree of different isoforms of α-Na,K-ATPase for vertebrate organisms. The tree generated using Random Forest model with information gain criteria when run on FCDS dataset. aa: amino acid

In spite of different isoforms in vertebrate, the biochemical properties and the length of the protein were important in the classification of different organisms (Fig. 6). In the decision tree, the organisms with different levels of evolution were separated in different routes. Most vertebrates were separated through route I, most fungi through route II, most prokaryotes through route III, most Protista through route IV, and most invertebrates through route V (Fig. 6). The most effective and basal attribute in the classification of organisms was the count of Gly-Cys (Fig. 6) and the highest value for this attribute was observed in vertebrates, which are evolutionarily superior. On the other hand, the number of Gly-Cys in all prokaryotes, which are evolutionary inferior, is less than 2.5 and then their sequences were separated from other organisms in two paths (Fig. 6). Most prokaryote’s sequences were isolated from others through path 8 in just three steps, the number of Gly-Cys was less than 2.5, then the ratio of Ala/Cys was less than 5.647 and then the length was less than 973 (Fig. 6). The fungal sequences were also separated from others in two paths that most of them isolated through path seven (route II) (Fig. 6). This path was common with the main path of prokaryotes and in the last step was separated by the length of protein, and then separated from Protista and invertebrate if the ratio of Cys/His was less or equal to 0.77 (Fig. 6).

Because the number of Gly-Cys played an important role in the separation of different organism groups, it was further analyzed by the alignment. The GC dipeptide presented in three positions, ^142^GC, ^208^GC, and ^702^GC with different percentages among different groups of metazoa, while we could not find them in prokaryotes, Protista and fungi (all position numbers in this paper refer to the sequence in GenBank accession number ADB19852.1, *Sus scrofa*). All vertebrate’s sequences had these dipeptides, but no nematoda’s sequences with Stramenopiles in phylogenetic tree groups II (Fig. 1) had these dipeptides (Additional file 1: Figs. S6, S7 and S8) although there were some invertebrates in phylogenetic tree group II and III that had ^142^GC and ^702^GC dipeptides. But, ^208^GC dipeptide was present only in vertebrate’s sequences.

#### Decision tree for different isoforms of α-NKA in vertebrates

**Fig. 7:**
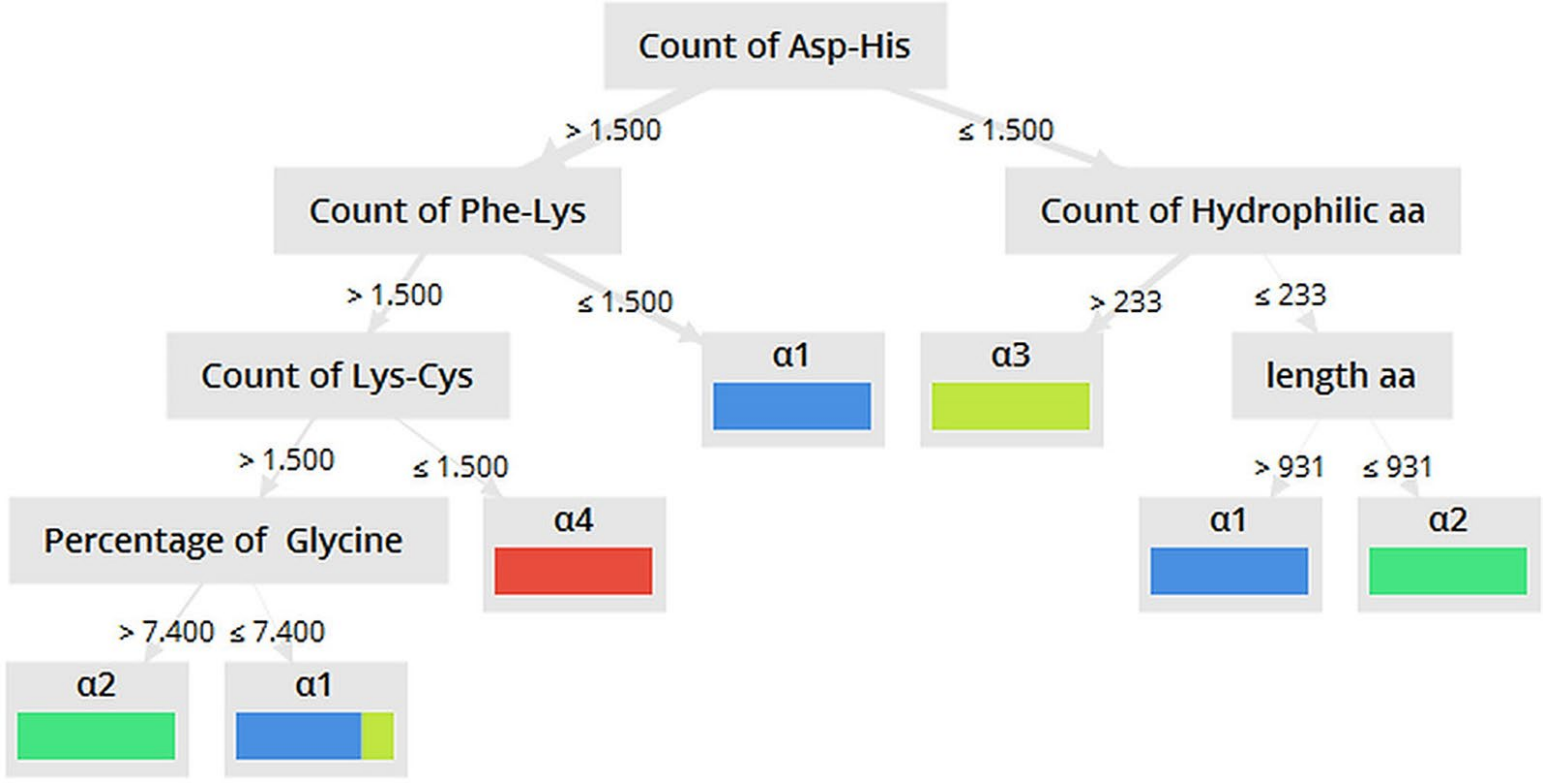
The Decision tree of α-Na,K-ATPase for all organisms. The tree generated using Decision tree model with information gain criteria when run on FCDS dataset. aa: amino acid

Decision tree analysis was done to identify the most important traits in separation sequences of vertebrates’ isoforms into four groups (α1, α2, α3, and α4). In this study, sequences were used whose isoform group was identified in databases (231 seq.). Data cleansing led to a reduction in attributes from 1252 to 577. The 10 datasets created using weighting algorithms had a minimum and a maximum of 24 and 73 attributes in relief and PCA dataset, respectively (Additional file 1: Table. S3) which along with FCD dataset, created 176 trees. The results indicated that the performance of the decision tree varied from 40.9% to 99.57% (Table 2). The best performance was related to the Decision Tree and Random Forest model with information gain criteria when run on FCD and Chi square dataset, respectively. Since the tree obtained from Random Forest model, by creating fewer branches, showed a simpler grouping of sequences, it was selected as the best tree. In this model, the count of Asp-His was the basal and most protein attribute. Using this model, four isoforms completely separated from each other based on the dipeptide count. The count of Asp-His plays a basic role. If the count of Asp-His is equal to or less than 1.5, and, in the next step, the count of hydrophilic amino acids is more than 233, the sequence is recognized as α3. Most of α3 sequences were separated from other isoforms based on this path (Fig. 7). All α4 sequences were also completely separated from α2 in a special path (Fig. 7).

**Table 2:** The percentage of performance for each model with different criterion for α-NKA in different organisms

Tree model	Decision tree				Random Forest			
Criterion	Gain ratio	Information gain	Gini index	Accuracy	Gain ratio	Information gain	Gini index	Accuracy
FCDS	97.74	99.33	98.80	98.80	84.95	92.28	86.42	84.55
Chi square	98.27	98.33	98.67	98.93	96.27	95.34	94.14	92.68
Info gain	96.01	98.54	97.74	97.47	91.08	94.27	93.21	88.95
Deviation	88.81	93.34	90.01	86.55	79.36	82.16	76.17	75.63
Gini index	97.47	98.40	97.74	96.14	92.14	91.74	90.01	89.61
Info gain ratio	98.40	98.67	98.28	97.47	94.54	96.27	94.54	92.28
PCA	59.12	98.14	97.74	91.74	90.28	90.81	90.15	85.89
Relief	91.88	96.94	96.14	90.01	84.95	86.68	84.95	84.02
Rule	97.60	97.60	97.60	93.21	94.14	96.54	93.21	89.88
Uncertainty	94.54	98.54	98.14	95.61	92.81	90.81	90.68	87.08
SVM	95.87	97.74	96.54	92.41	85.35	87.08	84.95	82.82

Tree model	Decision Stumpt				Random Tree			
Criterion	Gain ratio	Information gain	Gini index	Accuracy	Gain ratio	Information gain	Gini index	Accuracy
FCDS	51.26	77.36	77.36	77.36	48.20	82.56	84.42	76.56
Chi square	51.26	77.23	77.23	77.23	92.01	87.75	91.88	84.69
Info gain	51.26	77.36	77.36	77.36	85.22	88.95	91.08	78.83
Deviation	47.14	62.72	63.38	47.14	49.93	80.16	85.09	45.54
Gini index	51.00	77.36	77.36	77.36	85.89	89.88	89.21	83.22
Info gain ratio	51.26	77.36	77.36	77.36	51.53	90.95	95.21	85.62
PCA	51.26	70.04	72.57	72.57	50.73	81.09	88.15	75.50
Relief	77.36	77.36	77.36	77.36	83.36	83.09	85.75	74.70
Rule	51.26	72.97	72.70	72.70	47.14	49.13	78.30	49.53
Uncertainty	51.26	77.36	77.36	77.36	85.89	89.75	89.88	83.49
SVM	77.36	77.36	77.36	77.36	82.56	79.89	81.89	82.82

To determine the possible role and function of each of these dipeptides, the alignment for sequences was done. The results showed that three special dipeptides, ^41^DH, ^431^FK, and ^451^KC had more important roles in separating different isoforms. The dipeptide of ^41^DH is located between conserved motif ^33^LKKE and conserved amino acid ^52^K in all isoforms except α3 but in α3 there is a EH dipeptide (Additional file 1: Fig. S9). In the decision tree, α3 was separated from other isoforms in the first step by the number of DH dipeptide (Fig. 7). Another dipeptide, ^431^FK dipeptide, which is close to ^447^GDASE (Additional file 1: Fig. S10), separated α1 from α2 and α4 in the decision tree. FK dipeptide located in this position in all isoforms except α1. Mostly FL, FQ and EH dipeptides are present in this position of α1 isoform (Additional file 1: Fig. S10). In addition, in the position 456, close to ^447^GDASE, there is the KF dipeptide in α4, but in other isoforms, a KC dipeptide is present in this position, and α4 was separated from other isoforms based on this dipeptide in decision tree (Additional file 1: Fig. S10).

A mapping of states of each of dipeptides ^41^DH, ^431^FK, and ^451^KC for each of vertebrate sequences on the phylogenetic tree was done that reflected result of the decision tree and alignment in different appearance (Additional file 1). The result showed the sequences containing dipeptides ^41^DH and ^431^FK separated α3 and α1 isoforms from the others and then ^431^FK dipeptide separated α4 isoform from others (in fact, after α3 and α1 isoforms separated from others, ^431^FK dipeptide separated α2 and α4 isoforms) (Additional file 1: Fig. S1, S2 and S3). Actually sequences that have dipeptides ^431^FK and ^451^KC have evolved as α1 isoforms, while the sequences with dipeptides ^41^DH and ^431^FK have evolved in α3 isoforms. Also, sequences with dipeptides ^451^KC have evolved in the α2 isoforms. Finally, the sequences containing dipeptide KF in site of amino acid 451 belong to α4 isoforms.

## Discussion

In this study, Na/K-ATPase pumps were studied in different organisms to find the evolutionary relationships and how evolution impacted structural changes using phylogenetic analysis and decision tree and attribute weighting.

The study of the four groups obtained from the analysis of the phylogeny of different organisms provides information on structural changes according to their evolutionary position from prokaryotes to complex eukaryotes. No such evolutionary process was observed in the results of Saez et al. in investigation P-type ATPase IIC [42]. Therefore, increasing the number of sequences can lead to a more comprehensive understanding of evolutionary relationships. One of the important structural changes is the presence of α/β subunit assembly motif, since the functional expression of the pump is associated with the assembly of the α- and β-subunits. Actually, β-subunit is important in the maturation and transport of the enzyme to the plasma membrane [43]. However, it has been supposed that some sequences without this motif may exist without β-subunit or have a homologous sequence in their structure [34, 44]. It is also possible that sequences without α/β subunit assembly motif, have a role other than ion transfer function, like Ce2C3 and Ce2C5 from *C. elegans*, which were included in group II with some sequence of Stramenopile, in this study. The lack of α/β subunit assembly motif is not the only reason for the presence of *C. elegans* and *T. canis* sequences in this group, since a number of species in group III do not have this motif. Therefore, other differences such as the lack of motifs and amino acid positions may cause this grouping.

Okamura et al. [34] suggested two possible models for the evolution of α-NKA, which were based on the α/β subunit assembly: model 1) the ancestral form possessed a specific motif for subunit assembly and was lost during evolution, and model 2) the ancestral form lacked this motif and obtained it during evolution. Our results support model 2 because most of the sequences in group I belong to prokaryotes. Moreover, as we move from group I to group IV, the organisms have a higher evolutionary level. Finally, in group IV, we have vertebrate sequences. Based on these results, it may be suggested that the α/β subunit assembly originated after eukaryotes diverged from prokaryotes and during evolution the assembly site arose from the ancestral form.

It has been previously suggested that the β subunit appeared before the emergence of Metazoans in a Holozoan ancestor [45]. Our results can examine this proposal in more detail. The presence of this motif in some sequences from choanoflagellate indicated the emergence of the β subunit before Metazoans. Also our results suggest that the motif of the α/β subunit assembly acquisition occurred at a more primitive level of evolution due to its absence in the fungi and Protista except choanoflagellate. With attention to this point, the fungal kingdom belongs to Holomycota, and also the emergence of Protista existed before the divergence of Holozoa and Holomycotoa, so it may have suggested that the subunit-assembly motif appeared in Holozoa after diverged from Holomycota. Phylogenetic analysis identified the relationship of type of isoforms in vertebrates. This evolutionary path began in fish and then other groups (bird, reptile and mammal) originated from its [46]. As the phylogenetic tree showed, the evolutionary relationship of every isoform mostly corresponds to the evolutionary and taxonomic relationship among different groups of vertebrates.

The phylogenetic tree and the high similarity between the groups in each isoform suggest that the separation of the isoforms occurred in fish ancestors before the splitting of the groups. The high similarity between different organisms for α3 isoform compared to α1 and α2 isoforms can confirm the suggestion of Broude et al. to separate the ancestor of them from α3 isoform [47]. Placement of a sequence of lungfish next to other vertebrates confirms the suggestion of Romer and Williams [48] for a close association of tropical lungfish with the ancestry of land vertebrates. Separation of sequences of Chondrichthyes from other fish in group of α1 isoform is consistent with the results of Romer and Williams [48] that showed Chondrichthyes differ from their relatives in some attributes.

Complete separation of types of isoforms was associated with a close relationship between α2 and α4 confirming the suggestion of Clausen et al. [49] that the α4 may originate from a gene duplication of α2 in mammals. Placing the α2 isoform of fish next to α4 isoform of mammals was similar to the results of Saez et al. [42] in which the α4 isoform also showed the long length of branches. Also, the lack of isoforms in invertebrates, similar to types of vertebrate, indicates the emergence of isoforms after splitting which is in agreement with the results of phylogenetic tree analysis from previous studies [34, 42]. The presence of the motif of α/β subunit assembly in most vertebrates is to be expected, it has been previously shown that this assembly is necessary for their proper function [43].

Phylogenetic analysis used homology of sequences to determine the evolutionary relationship. To make full use of sequence information, the traits extracted from them were analyzed using the attribute weighting and decision tree to identify the factors affecting the difference between isoforms and types α-NKA proteins in taxonomic groups.

In this study, a combination of different attributes of protein structure was used, which increases the classification efficiency [50]. Then, different weighting algorithms were used to determine the most important attributes separating isoform types. Of all these attributes, the number of different dipeptides and amino acid ratios were more replicated in different models than the other attributes. As the results showed, the types of dipeptides resulting from the combination of different amino acids and their different ratios were identified as the most important feature in different weighting algorithms. It has been previously reported that the dipeptide count is a significant protein attribute in the classification of different proteins and prediction of their function [22, 23]. So, in α-NKA, the kind of isoform can be distinguished from special attributes such as dipeptide compositions and the ratio of amino acids.

The decision tree is a powerful classification method and for the first time, this method was used to classify different isoforms of α-NKA in vertebrates and also α-NKA in all organisms based on sequence-based features. To compare and determine the best and most efficient model to construct a decision tree, the percentage of performance of each model was used. The wide range of performance value for the types of trees generated in both groups indicated that different trees have different capabilities in the classification of different organisms based on NKA α-subunit and the classification of different isoforms of NKA α-subunits.

Different types of amino acid traits, especially dipeptides, were involved in the generation of these trees. The deep analysis of dipeptides may identify conserved amino acids and motifs that may play an important role in the differentiation of different groups.

In this study, the ^208^GC dipeptide as a basic attribute in the separation of vertebrates from other organisms may play a role in the enzyme dephosphorylation and activity inhibition of enzyme and prevents spatial conformation stabilization due to proximity to the conserved motif TGES in the first cytoplasmic loop [51, 52]. In general, conserved areas and motifs for the NKA α-subunit increase in more complex organisms. There is a similar trend of change for the presence the α/β subunit assembly motif [53].

To distinguish types of NKA α-isoforms, ^41^DH, ^431^FK, and ^451^KC dipeptides were effective. The conserved motif ^33^LKKE and conserved amino acid ^52^K are on both sides of the ^41^DH dipeptide that plays an important role in the enzyme regulation [16]. ^431^FK, and ^451^KC dipeptides are on both sides of the ^447^GDASE motif that has a critical role in binding to ATP [54, 55]. Thus, it may be concluded that these dipeptides in this position may play an important role in the function of different vertebrate isoforms. These dipeptides are actually specific attributes that can lead to a better understanding of the phylogenetic tree. Palmgren et al. studied the evolution of P2A and P5A ATPases using the phylogenetic tree and by in-depth investigation of protein sequences identified synapomorphies (attributes) belonging to each group in the phylogenetic tree that including conserved amino acids [56].

The decision tree results were consistent with the phylogenetic tree’s results and both methods were able to separate α2 from α4, despite their high similarity. This supports the hypothesis that α4 may originate from an α2 gene duplication Clausen et al. [49].

In general, according to the position of the identified dipeptides in relation to functional conserved sites, their possible predicted role can be investigated through experimental studies including amino acid substitution and mutagenesis.

## Conclusions

The classification of different isoforms of proteins or different organisms based on specific proteins can improve the understanding of protein evolution. Investigation of the similarities and differences among protein sequences using simple methods may lead to wrong conclusions about the the evolutionary path of proteins on. Thus, it is important to use more sophisticated and efficient methods with a strong statistical basis to determine the relation among different isoforms and the same protein in different organisms. Here, for the first time, two different methods, the phylogenetic tree and the decision tree, were simultaneously used to investigate the relationship between different isoforms of α-Na,K-ATPase in vertebrates and compare this enzyme among different organisms. Phylogenetic analysis showed that the sequences were divided into four groups according to the evolutionary process from prokaryotes to complex vertebrates, and in vertebrates into four isoform types. This enabled the determination of the evolutionary path of the isoforms. Also, the decision tree along with alignment showed that some protein attributes that play an important role in the evolutionary process of this protein, and probably in the function of different isoforms of this protein. Thus, despite the variety of experimental methods for identifying functional protected structures, it is possible to obtain hidden information within the sequence by combining bioinformatics methods to find a possible functional position in the evolutionary path.

## Methods

### Sequences collection

Seven hundred and fifty-three sequences of Na/K ATPase pumps alpha-subunit (Additional file 1: Table. S4) from various organisms of three life domains (bacteria, archaea and eukaryote) were extracted from the UniProt (https://www.uniprot.org/) and NCBI (https://www.ncbi.nlm.nih.gov/) through a blast search. Since brine shrimp (*Artemia* spp.) as the halophilic organisms have different evolutionary strategies, including high pump activity, α1 subunit of *A. franciscana* with high intraspecific diversity were used as the query sequence (UniProt accession number P28774; [42, 57, 58]). The accession number of some sequences related to bacteria, archaea and fungi were collected from some literatures. Then 1,252 amino acid attributes including weight, length, aliphatic index, isoelectric point, frequency, and count of hydrophobic and hydrophilic residues, frequency and count of each amino acid, frequency and count of dipeptides, frequency and count of each element (H, C, O, S, and N), frequency and count of positively and negatively charged amino acids, amino acid ratio, frequency and count of dipeptides and other secondary protein attributes which were extracted by CLC bio Protein Workbench Software version 7.6 (QIAGEN). A dataset of the sequence attributes was imported into Rapid Miner Studio 7.6 (Rapid-I, Dortmund, Germany).

Also, 335 sequences of ssu rRNA from various organisms of three life domains were collected from SILVA and RNAcentral databases (Additional file 1: Table. S5) [59, 60].

### Phylogenetic tree-clustering analysis

Phylogenetic trees were drawn for two datasets including whole sequences that belonged to different groups of organisms (753 sequences) and sequences belonging to four isoform of vertebrates (323 sequences). Phylogenetic analysis was also performed for 680 fungal sequences belonging to different groups of P-Type II ATPase to separate NKA proteins (P-Type IIC ATPase) from P-Type IIE ATPase, accurately. Phylogenetic analysis was performed as follows. Also, phylogenetic tree was draw for sequences of ssu rRNA (335 sequences). Multiple sequence alignment of α-NKA sequences was carried out using MAFFT v7 [61]. A phylogenetic tree was generated by maximum-likelihood method using PhyML v3 [62]. Smart Model Selection in PhyML was used for the selection of the best model with Akaike Information Criterion (AIC) [63]. The best models for each of four trees (753, 323, 680 and 335 sequences) were LG + G + I + F, Q.insect + R, and Blosum62 + R + F. value of log likelihood for each of them were -372,394.32, -57,691.68, and -145,967.61, respectively. Then the phylogenetic tree was drawn with aBayes criteria for branch supports. The phylogenetic trees were visualized using MEGA7.0 software (http://www.megasoftware.net/).

### Decision tree- classification analysis

Decision tree analysis was drawn to identify the most important attributes in different groups of organisms and vertebrate isoforms. Therefore, the decision tree was drawn for five different taxonomic groups of organisms (vertebrates, invertebrates, fungi, Protista and prokaryotes) and four isoform types (α1, α2, α3 and α4) in vertebrates. For this purpose, extracting amino acid attributes for each sequence was imported into Rapid Miner Studio 7.6 (Rapid-I, Dortmund, Germany). Then dataset was cleaned and 10 new data sets were created using different weighting algorithms, which were used to create types of decision tree. The best decision tree was selected to introduce the most important attributes based on percentage of performance. Decision tree analysis was performed as follows.

### Data cleansing

Data cleansing algorithms were used to remove useless, correlated and repetitive attributes from dataset. These attributes consisted in numerical attributes with standard deviation less or equal to a given deviation threshold (0.1) and strongly correlated attributes, with correlation greater than 0.9, respectively. The final dataset was labeled as Final Clean Dataset (FCD).

### Weighting algorithms

Ten following weighting models were employed to figure out the most effective protein attributes for classification in Rapid Miner Studio 7.6 software by defaults. Normalized data were evaluated by different weighting algorithms and the importance of each attribute regarding the target label determined from 0 (lowest) to 1 (highest).

### Weighting by Chi-square

In this model, the relevance of attributes was determined by calculating the weight of attributes with respect to the class attribute using the Chi-square statistic.

### Weighting by deviation

The weight of each attribute was determined with respect to the label attribute on the basis of standard deviation of the attributes. The values were normalized by dividing by average, minimum or maximum of the attributes and then the relevance of attributes was calculated.

### Weighting by Gini index

This model was applied to reveal the relevance of attributes on the basis of Gini index and assigns weights to them accordingly.

### Weighting by information gain ratio

By this approach, the weight of attributes was determined with respect to the label attribute by calculating the information gain ratio of class distribution.

### Weighting by information gain

By this approach, the weight of attributes was determined with respect to the label attribute by calculating the information gain of class distribution.

### Weighting by Principle Component Analysis, PCA

Here, the components of PCA were used to weight each attribute.

### Weighting by relief

Using this method, the relevance of attributes was determined by sampling, and estimating the value of each attribute according to how well the values distinguish between examples from the same and different classes.

### Weighting by Rule

In this model, the relevance of attributes was determined by constructing a rule for each attribute and calculating the error.

### Weighting by Support vector machine, SVM

Regarding SVM, the coefficients of the normal vector of a linear SVM were used to determine the weight of each attribute.

### Weighting by uncertainty

By this Model, the weight of attributes was determined with respect to the label attribute by calculating the symmetrical uncertainty with respect to the class.

### Attribute evaluation

After running the attribute weighting models on the dataset, each attribute is assigned a score from 0 to 1, indicating the importance of attribute in the classification for different taxonomic groups of organisms (vertebrates, invertebrates, fungi, Protista and prokaryotes) and isoform types in vertebrates (α1, α2, α3 and α4). In taxonomic groups of organisms, the attributes with a score equal to or higher than 0.7, except deviation and PCA equal to or higher than 0.8 and Gini index, info gain and uncertainty equal to or higher than 0.6 were selected and others were removed. In isoform types of vertebrates, the attributes with a score equal to or higher than 0.7, except Chi-square, Rule, and uncertainty equal to or higher than 0.6, relief equal to or higher than 0.5, and deviation equal to or higher than 0.26 were selected and others were removed. Supervised models were applied at first to basic dataset, and then for 10 new datasets were created from the basic dataset by above attribute weighting algorithms.

### Decision tree models

Indeed, four models of decision trees (Decision Tree, Random Forest, Decision Stumpt and Random Tree) with four criteria (Gain ratio, Information gain, Gini index and Accuracy) were run on eleven datasets including FCD dataset and ten other datasets namely as Chi-square, Info gain, Deviation, Gini index, info gain ratio, PCA, Relief, and Rule to pick out the best models to classify different group of organisms and isoforms of vertebrates based on α-NKA proteins. Default criteria were used for each model in Rapid Miner Studio 7.6 software.

## Supplementary Information

**Additional file 1**. Supplementary figures and tables.

## Data Availability

The datasets generated during the current study are available in the [Figshare] repository, [https://figshare.com/s/aac95fdcd29551027278].
