# Supplementary material for: Bioinformatics approaches for classification and investigation of the evolution of the Na/K-ATPase alpha-subunit
Source: BMC Ecol Evol. 2022 Oct 26;22:122. doi: 10.1186/s12862-022-02071-0 (PMC9609216; doi:10.1186/s12862-022-02071-0)
Supplement: Supplementary file 1 — Additional file 1. Supplementary figures and tables. [file 12862_2022_2071_MOESM1_ESM.zip › Additional file 1 Fig. S10.pdf]

a4.XP_004448484.1	IAGLCNRADFKANQESLPLAKRATAGDASESALLKFVEQTYGSVKEMREKNPKVAEIPFN	492
a4.XP_023103614.1	IAGLCNRADFKANQETLPIVKRATTGDASESALLKFIEHSYSSVKEMREKNPKVAEIPFN	493
a4.XP_010593170.2	IAGLCNRADFKANQETLPIAKRETTGDASESALLKFIEQTYSPVKEMRNKSPKVAEIPFN	452
a4.XP_020740848.1	IAGLCNRADFKANEENLPIAKRATTGDASESALLKFIEQSYSSVKEIREKNPKVAEIPFN	492
a4.XP_023496657.1	IAGLCNRADFKPDQETLPIAKRATTGDASESALLKFIEQSYSSVKEMREKSPKVAEIPFN	498
a4.XP_006096963.1	IAGLCNRADFKANQEDVPIAKRTTAGDASESALLKFIEQSYCSVKEMREKSPKVAEIPFN	493
a4.XP_006922963.1	IAGLCNRADFKANQETLPIAKRATAGDASESALLKFIEQFYSSVKEMREKSPKVAEIPFN	492
a4.XP_011371380.1	IAGLCNRADFKANQETLPIAKRATAGDASESALLKFIEQFYSSVKEMREKSPKVAEIPFN	492
a4.XP_545754.3	IAALCNRADFKPNQETLPIAKRATTGDASESALLKFMEQSYSSVKEMREQNPKVAEIPFN	493
a4.XP_021537588.1	IAGLCNGADFKAHQETLPIAKRATAGDASESALLKFIEQSYSSVKEMREKNPKVAEIPFN	492
a4.XP_021506251.1	IAGLCNRADFKPHQESVPITKRTTTGDASESALLKFIEQSYSPVNEMRQKNPKVAEIPFN	492
a4.NP_074039.	IAGLCNRADFKPHQESLPITKRTTTGDASESALLKFIEQSYSPVSEMRQKNPKVAEIPFN	490
a4.XP_021014708.1	IAGLCNRADFKPHQESVPIAKRTTTGDASESALLKFIEQSYNPVSEMRQKNPKVAEIPFN	494
a4.NP_038762..	IAGLCNRADFKPHQESVPIAKRATTGDASESALLKFIEQSYNPVSEMRQKNPKVAEIPFN	494
a4.XP_020024800.1	IAGLCNRADFKPHQETLPITKRATTGDASESALLKFIEQSYSSVKEMREKNPKVAEIPFN	491
a4.XP_003795244.1	IAGLCNRADFKPQQETVPVAKRATTGDASESALLKFIEQSHGSVAEMREKYPKVAEIPFN	492
a4.XP_004639995.1	IAGLCNRADFKAEQEMLPIAKRETTGDASESALLKFVEQSFSSVKEMREKNPKVAEIPFN	496
a4.XP_021568356.1	IAGLCNRADFKAHQETLPIAKRITTGDASESALLKFVEQSYSSVAEMRERSPKVAEIPFN	491
a4.XP_012604632.1	IAGLCNRADFKPNQERLSIAKRTTTGDASESALLKFIEQTYSPVSKMREKNPKVAEIPFN	490
a4.NP_653300	IAGLCNRADFKANQEILPIAKRATTGDASESALLKFIEQSYSSVAEMREKNPKVAEIPFN	491
a4.XP_003892961.1	IAGLCNRADFKANQEILPIAKRATTGDASESALLKFVEQSYSSVAEMREKNPKVAEIPFN	491
a4.XP_023069991.1	IAGLCNRADFKANQEILPIAKRATTGDASESALLKFIEQSYSSVAEMREKNPKVAEIPFN	491
a4.UPI0001C650F7	IAGLCNRADFKTNQEHLPIAKRSTTGDASESALLKFIEQTYSSVTEMREKSPKVAEIPFN	489
a4.XP_013220247.1	IAGLCNRADFKAHQETLPIAQRETTGDASESALLKFIEQSYSSVKEMREKYPKVAEIPFN	449
a1.XP_023390675.1	IAGLCNRAVFQANQENLPILKRAVAGDASESALLKCIELCCGSVKEMREQYAKIVEIPFN	452
a2.Q98SL3	VAGLCNRADFLPGQESVPILKRDTAGDASESALLKCIELSCGSVRSLREKNNKVAEIPFN	470
a1.BAJ13363.1	VAGLCNRAVFLAEQNNVPILKRDVSGDASETALLKCIELCCGSVKDMREKYSKVVEIPFN	481
a1.UPI00001DFF4A	VAGLCNRAVFLAEQNNVPILKRDVSGDASETALLKCIELCCGSVKDMREKYSKVVEIPFN	482
a1.UPI00025FADDE	VAGLCNRAVFLAGQNDVPILKRNIAGDASEAALLKCIELCCGSVSEMREKYPKIAEIPFN	480
a1.NP_571762.1	VAGLCNRAVFRAEQSHLPVLNRETAGDASESALLKCIELCCGSVIEMREKYRKICEIPFN	483
a1.NP_571763.1	VAGLCNRAVFQSNQSHIPVLKRDTAGDASESALLKCIELSCGSVAEMRENYTKLAEIPFN	484
a1.NP_835200.1	VAGLCNRAVFQSNQSHLPVLRRETAGDASESALLKCIELCCGSVTGMRENYPKVAEIPFN	483
a3.UPI000C736357	IAGLCNRAVFKGGQDNIPVLKRDVAGDASESALLKCIELSSGSVKLMRERNKKVAEIPFN	469
a3.F7E0B8	IAALCNRAVFKAGNDNIPVLKRDVAGDASESALLKCIELSCGSVKAMREKSKKVAEIPFN	485
a3.UPI0003CD047A	IAGLCNRAVFKGGQDNVPVLKRDVAGDASESALLKCIELSSGSVKLMRERNKKVAEIPFN	488
a3.UPI000C73EFA7	IAGLCNRAVFKGGQDNIPVLKRDVAGDASESALLKCIELSSGSVKLMRERNKKVAEIPFN	509
a3.UPI00049A9E19	VAALCNRAVFKAGQDNLPVLKRDVAGDASESALLKCIELSCGSVKLMREKYRKVAEIPFN	492
a3.XP_025028557.1	IAGLCNRAVFKGGQENVPILKRDVAGDASESALLKCIELSSGSVKLMREKNRKVAEIPFN	487
a3.XP_020663591.1	IAGLCNRAVFKGGQENVPILKRDVAGDASESALLKCIELSSGSVKLMREKNKKVAEIPFN	548
a3.UPI0000124FC2	IAGLCNRAVFKGGQENVPILKRDVAGDASESALLKCIELSSGSVKVMRERNKKVAEIPFN	470
a3.UPI000BAD5294	IAGLCNRAVFKGGQDNIPVLKRDVAGDASESALLKCIELSSGSVKLMRERNKKVAEIPFN	469
a3.XP_020948935.1	IAGLCNRAVFKGGQDNIPVLKRDVAGDASESALLKCIELSSGSVKLMRERNKKVAEIPFN	487
a3.XP_012613923.1	IAGLCNRAVFKGGQDNIPVLKRDVAGDASESALLKCIELSSGSVKLMRERNKKVAEIPFN	473
a3.UPI000C2D7C35	IAGLCNRAVFKGGQDNIPVLKRDVAGDASESALLKCIELSSGSVKLMRERNKKVAEIPFN	490
a3.XP_006903931.1	IAGLCNRAVFKGGQDNIPVLKRDVAGDASESALLKCIELSSGSVKLMRERNKKVAEIPFN	443
a3.XP_023380497.1	IAGLCNRAVFKGGQDNIPVLKRDVAGDASESALLKCIELSSGSVKLMRERNKKVAEIPFN	544
a3.UPI0002B3612F	IAGLCNRAVFKGGQDNVPVLKRDVAGDASESALLKCIELSSGSVKLMRERNKKVAEIPFN	483
a3.XP_020726792.1	IAGLCNRAVFKGGQDNVPVLKRDVAGDASESALLKCIELSSGSVKLMRERNKKVAEIPFN	438
a3.UPI000226419C	IAGLCNRAVFKGGQDNVPVLKRDVAGDASESALLKCIELSSGSVKLMRERNKKVAEIPFN	488
a3.UPI000C7286EF	IAGLCNRAVFKGGQDNIPVLKRDVAGDASESALLKCIELSSGSVKLMRERNKKVAEIPFN	473
a3.UPI0007A6EC9C	IAGLCNRAVFKGGQDNIPVLKRDVAGDASESALLKCIELSSGSVKLMRERNKKVAEIPFN	473
a3.UPI000C2DAA95	IAGLCNRAVFKGGQDNIPVLKRDVAGDASESALLKCIELSSGSVKLMRERNKKVAEIPFN	479
a3.UPI000C740E55	IAGLCNRAVFKGGQDNIPVLKRDVAGDASESALLKCIELSSGSVKLMRERNKKVAEIPFN	484
a3.XP_021021704.1	IAGLCNRAVFKGGQDNIPVLKRDVAGDASESALLKCIELSSGSVKLMRERNKKVAEIPFN	486
a3.UPI00035B05DE	IAGLCNRAVFKGGQDNIPVLKRDVAGDASESALLKCIELSSGSVKLMRERNKKVAEIPFN	508
a3.XP_023600635.1	IAGLCNRAVFKGGQDNIPVLKRDVAGDASESALLKCIELSSGSVKLMRERNKKVAEIPFN	452
a3.UPI000C732D5F	IAGLCNRAVFKGGQDNIPVLKRDVAGDASESALLKCIELSSGSVKLMRERNKKVAEIPFN	484
a3.NP_036638.	IAGLCNRAVFKGGQDNIPVLKRDVAGDASESALLKCIELSSGSVKLMRERNKKVAEIPFN	473
a.UPI000CB4CAB6	IAGLCNRAVFKGGQDNIPVLKRDVAGDASESALLKCIELSSGSVKLMRERNKKVAEIPFN	484
a3.XP_022441242.1	IAGLCNRAVFKGGQDNIPVLKRDVAGDASESALLKCIELSSGSVKLMRERNKKVAEIPFN	487
a3.UPI000651771D	IAGLCNRAVFKGGQDNIPVLKRDVAGDASESALLKCIELSSGSVKLMRERNKKVAEIPFN	473
a3.UPI000C7355ED	IAGLCNRAVFKGGQDNIPVLKRDVAGDASESALLKCIELSSGSVKLMRERNKKVAEIPFN	498
a3.XP_021590883.1	IAGLCNRAVFKGGQDNIPVLKRDVAGDASESALLKCIELSSGSVKLMRERNKKVAEIPFN	510
a3.XP_024433413.1	IAGLCNRAVFKGGQDNIPVLKRDVAGDASESALLKCIELSSGSVKLMRERNKKVAEIPFN	473
a3.UPI000C2EDFE7	IAGLCNRAVFKGGQDNIPVLKRDVAGDASESALLKCIELSSGSVKLMRERNKKVAEIPFN	484
a3.UPI000C2E3154	IAGLCNRAVFKGGQDNIPVLKRDVAGDASESALLKCIELSSGSVKLMRERNKKVAEIPFN	486
a3.XP_023507169.1	IAGLCNRAVFKGGQDNIPVLKRDVAGDASESALLKCIELSSGSVKLMRERNKKVAEIPFN	474
a3.KFO33633.1	IAGLCNRAVFKGGQDNIPVLKRDVAGDASESALLKCIELSSGSVKLMRERNKKVAEIPFN	443
a3.UPI000C71DF25	IAGLCNRAVFKGGQDNIPVLKRDVAGDASESALLKCIELSSGSVKLMRERNKKVAEIPFN	484
a3.UPI0001914BDE	IAGLCNRAVFKGGQDNIPVLKRDVAGDASESALLKCIELSSGSVKLMRERNKKVAEIPFN	486

a3.XP_003799510.1	IAGLCNRAVFKGGQDNIPVLKRDVAGDASESALLKCIELSSGSVKLMRERNKKVAEIPFN	486
a3.XP_008065591.1	IAGLCNRAVFKGGQDNIPVLKRDVAGDASESALLKCIELSSGSVKLMRERNKKVAEIPFN	443
a3.sp|P13637.3|	IAGLCNRAVFKGGQDNIPVLKRDVAGDASESALLKCIELSSGSVKLMRERNKKVAEIPFN	473
a3.UPI000050D2B6	VAGLCNRAQFKAGQDALPILKRDVAGDASESALLKCIELCCGSVRANRDRNKKVAEIPFN	468
a3.UPI0007F716B9	IAGLCNRAVFLAEQSNIPILKRDVAGDASESALLKCIELCCGSVQGMREKTPKIAEIPFN	469
a3.UPI0000E9CD46	IAALCNRAQFKAGQDPIPILKRDVAGDASESALLKCIELSCGSVRAMRDRNKKVAEIPFN	481
a3.UPI0007F7EA5B	IAALCNRAQFKAGQESVAILKRDVAGDASESALLKCIELSCGSVRQMRDRNKKVAEIPFN	482
a3.XP_020466584.1	VAALCNRAQFKAGQDSLPILKRDVAGDASESALLKCIELSCGSVRAMREKNKKVAEIPFN	483
a3.UPI0006B30A18	IAALCNRAQFKAGQDSVAILKRDVAGDASESALLKCIELSCGSVRMMRERNKKVAEIPFN	484
a3.UPI0004448FEC	IAALCNRAQFKAAQDSVPILKRDVAGDASESALLKCIELSCGSVRLMRDKNKKVAEIPFN	469
a3.XP_023187147.1	IAALCNRAQFKAGQDSVSILKRDVAGDASESALLKCIELSCGSVRLMRDKNKKVAEIPFN	482
a3.UPI0000124FC4	VAPLCNRAQFKPRQDSVSILKRDVAGDASESALLKCIELSCGSVRMMRDKNKKVAEIPFN	470
a3.UPI00025FB25F	VAALCNRAQFKAGQDSVAILKRDVAGDASESALLKCIELSCGSVRMMRDKNKKVAEIPFN	470
a3.UPI0000E3A2FA	VAALCNRAQFKAGQDQLPILKRDVAGDASESALLKCIELSCGSVRAMRDRNKKVAEIPFN	483
a3.UPI00003628C3	IAGLCNRAQFKAGQDSLPILKRDVAGDASESALLKCIELSFGVVRAMRDKNKKVAEIPFN	480
a3.XP_020504733.1	IAGLCNRAQFKAGQDQLPILKRDVAGDASESALLKCIELSCGSVRAMRDRNKKVAEIPFN	485
a3.UPI00032B9010	VAALCNRAQFKAGQDSIAILKRDVAGDASESALLKCIELSCGSVRLMRDKNKKVAEIPFN	470
a3.XP_022612296.1	VAALCNRAQFKAGQDSVAILKRDVAGDASESALLKCIELSCGSVRLMRDKNKKVAEIPFN	470
a3.XP_023285663.1	VAALCNRAQFKAGQDSVAILKRDVAGDASESALLKCIELSCGSVRLMRDKNKKVAEIPFN	483
a3.XP_021427657.1	VAALCNRAQFKAAQDQLPILKRDVAGDASESALLKCIELSCGSVRQMREKNKKVAEIPFN	488
a3.XP_024297426.1	VAALCNRAQFKAAQDQLPILKRDVAGDASESALLKCIELSCGSVRQMREKNKKVAEIPFN	493
a3.UPI00001DFF47	VAALCNRAVFKAGQDQLPILKRDTAGDASESALLKCIELSCGSVKQIREKNKKVAEIPFN	471
a3.UPI0006B7181A	VAALCNRAVFKAGQDQLPILKRDTAGDASESALLKCIELSCGSVKQIREKNKKVAEIPFN	484
a3.UPI000293B6B0	VAGLCNRAVFKAGQESLPILKRDVAGDASESALLKCIELSCGSVKGMRDKYKKVAEIPFN	482
a3.UPI0000E3AF2C	IAALCNRAVFKAGQDALPILKRDVAGDASESALLKCIELSCGSVKAMREKNKKVAEIPFN	482
a3.XP_024920682.1	IASLCNRAVFKAGQEALPILKREVAGDASESALLKCIELSCGPVKIMRDKNKKVAEIPFN	483
a3.UPI00016E235F	IAALCNRAVFKAGQEALPILKREVAGDASESALLKCIELSCGSVKAMRDKNKKVAEIPFN	485
a3.UPI00032B6FE9	IAALCNRAVFKAGQEALPILKREVAGDASESALLKCIELSCGAVKIMRDKNKKVAEIPFN	483
a3.XP_012711044.2	IAALCNRAVFKAGQESLPILKRDVAGDASESALLKCIELSCGSVKAIREKNKKVAEIPFN	482
a3.UPI00025F91A4	IAALCNRAVFKAGQESLPILKRDVAGDASESALLKCIELSCGSVKAMRDKNKKVAEIPFN	482
a3.XP_020793662.1	IATLCNRAVFKAGQEALPILKREVAGDASESALLKCIELSCGAVKAMRDKNKKVAEIPFN	482
a3.XP_024153267.1	IASLCNRAVFKAGQESLPILKRDVAGDASESALLKCIELSCGSVKALRDKNKKVAEIPFN	482
a3.XP_023117914.1	IAALCNRAVFKAGQESLPILKREVAGDASESALLKCIELSCGAVKSMRDKNKKVAEIPFN	482
a3.XP_022053465.1	IAALCNRAVFKAGQESLPILKREVAGDASESALLKCIELSCGAVKSMRDKNKKVAEIPFN	482
a3.NP_571759.2	VAALCNRAVFKAGQESLPILKRDVAGDASESALLKCIELSCGSVKAMRDKNKKVAEIPFN	483
a3.W5UML4	VASLCNRAVFKAGQESLPILKRDVAGDASESALLKCIELSCGSVKALRDKNKKVAEIPFN	484
a3.W5L4G0	VAALCNRAVFKAGQESLPILKRDVAGDASESALLKCIELSCGSVKAMRDKNKKVAEIPFN	482
a3.UPI0005D90DB9	VAALCNRAVFKAGQESLPILKRDVAGDASESALLKCIELSCGSVKAMRDKNKKVAEIPFN	482
a3.XP_023665796.1	VAALCNRAVFKAGQEPLPILKRDVAGDASESALLKCIELSCGSVKAMREKYKKVAEIPFN	482
a3.XP_015461719.2	VASLCNRAVFKAGQDSLPILKRDVAGDASESALLKCIELSCGSVKQMRERNKKVAEIPFN	484
a3.BAB60722.1	VAALCNRAVFKAQQDSLPILKRDVAGDASESALLKCIELSCGSVKMMREKNKKVAEIPFN	482
a3.UPI000054C9F5	VAALCNRAVFKAGQDSLPILKRDVAGDASESALLKCIELSSGSVKAMREKNKKVAEIPFN	483
a2.NP_571758.1	VGGLCNRAVFKAGQEEIPIRTRDTAGDASESALLKCVEILSGNVETLRGNNRKVAEIPFN	478
a2.UPI00000FE1CF	VAGLCNRAVFKSGQDELPILMRDTAGDASESALLKCIELCCGNVREMRARNRKVVEIPFN	469
a2.XP_020507674.1	VAGLCNRADFRAGQEDFPIVMRETAGDASESALLKCIELCCGSVREMRARNPKMVEIPFN	326
a2.BAO02373.1	VAGLCNRADFKAGQEDFPLQMRETAGDASESALLKCIELCCGSVRDMRARNPKVAEIPFN	471
a1.NP_571761.1	VAGLCNRAVFLAEQENIPILKRDVAGDASESALLKCIELCCGSVKEMREKYNKISEIPFN	486
a1.AJR20270.1	IAGLCNRAVFLADQDNVPILKRDTAGDASESALLKCIELCCGSVKDMRDKYTKIAEIPFN	486
a1.XP_023690671.1	IAGLCNRAVFLAEQTDVPILKRDVAGDASESALLKCIELCCGSVKEMRDKYAKIAEIPFN	484
a1.XP_008322794.1	VAGLCNRAVFLAEQSNVPILKRDVAGDASESALLKCIELCCGSVQGMREKNPKIAEIPFN	515
a1.XP_020497843.1	IAGLCNRAVFLAEQSNLPILKRDVAGDASESALLKCIELCCGSVQEMREKYPKISEIPFN	496
a1.UPI00066EFDEA	IAGLCNRAVFLAEQSNIAILKRDVAGDASESALLKCIELCCGSVQEMREKNPKISEIPFN	488
a1.XP_004571307.1	IAGLCNRAVFLAEQSNVPILKRDVAGDASESALLKCIELCCGSVQEMREKTPKIAEIPFN	484
a1.XP_022617258.1	IAGLCNRAVFLAEQSNIPILKRDVAGDASESALLKCIELCCGSVQEMREKSPKISEIPFN	484
a1.BAN17691.1	IAGLCNRAVFLAEQSSVPILKRDVAGDASESALLKCIELCCGSVQEMRDKTPKISEIPFN	484
a1.XP_012714443.1	VAGLCNRAVFLAEQSNIPILKRDVAGDASESALLKCIELCCGSVAQMREKTPKVAEIPFN	484
a1.XP_017282368.1	IAGLCNRAVFLAEQSNIPILKRDVAGDASESALLKCIELCCGSVQEMREKTPKIAEIPFN	488
a1.XP_004066573.1	IAGLCNRAVFLAEQSNIPILKRDVAGDASESALLKCIELCCGSVQEMRDRSPKIAEIPFN	484
a1.XP_024144684.1	IAGLCNRAVFLAEQSNIAILKRDVAGDASESALLKCIELCCGSVQEMREKSPKIAEIPFN	484
a1.sp|P25489.1|	IAGLCNRAVFLAEQIDVPILKRDVAGDASESALLKCIELCCGSVKEMREKFTKVAEIPFN	486
a1.XP_022536277.1	VAGLCNRAVFLAEQTDVPILKRDVAGDASESALLKCIELCCGSVKEMREKYSKAAEIPFN	484
a1.Q9DEU1	VAGLCNRAVFLAEQTDVPILKRDVAGDASESALLKCIELCCGSVKDMREKYTKVAEIPFN	484
a1.sp|Q92030.1|	IAGLCNRAVFLAEQSNVPILKRDVAGDASESALLKCIELCCGSVNDMRDKHVKIAEIPFN	482
a1.AJR20271.1	VAGLCNRAVFLAEQSNVPILKRETAGDASESALLKCIELCCGPVKDMRDKYPKISEIPFN	500
a1.Q90X33	VAGLCNRAVFLADQRNVPILKRDTAGDASESALLKCIELCCGSVNEMREKYTKIAEIPFN	484
a1.XP_004066575.1	IAGLCNRAVFLAEQDKVPILKRDVAGDASEAALLKCIELTCGSVNAFREKYPKIAEIPFN	482
a1.XP_024144685.1	VAGLCNRAVFLAEQENVPILKRNVAGDASEAALLKCIELTCGSVNAIREKYPKIAEIPFN	482
a1.XP_020476182.1	VAGLCNRAVFLAEQSNLPILKREVAGDASEAALLKCIELCCGSVKDMREKYPKIAEIPFN	486
a1.UPI000443A733	IAGLCNRAVFLAEQNNVPILKRDVAGDASEAALLKCIELCCGSVKDMREKYPKVAEIPFN	483
a1.XP_023185631.1	IAGLCNRAVFLAEQSNVPILKRDVAGDASEAALLKCIELCCGSVKDMREKYPKVAEIPFN	483
a1.sp|Q9YH26.2|	IAGLCNRAVFLADQSNIPILKRDVAGDASEAALLKCIELCCGSVNEMREKYPKIAEIPFN	483

a1.UPI00022B0848	IAGLCNRAVFLADQSNIPILKRDVAGDASEAALLKCIELCCGSVNEMREKYPKIAEIPFN	483
a1.XP_020792263.1	IAGLCNRAVFLADQNNVPILKRDVAGDASEAALLKCIELCCGSVAGMREKYPKVAEIPFN	484
a1.AKQ12834.1	IAGLCNRAVFLAEQSNIPILKRDVAGDASEAALLKCIELCCGSVGGMRDKYYKVSEIPFN	485
a1.XP_023275950.1	IAGLCNRAVFLAEQGNVPILKRDVAGDASEAALLKCIELVCGSVGGMRDKYPKNAEIPFN	484
a1.EMP33651.1	VAGLCNRAVFQANQENVPILKRAVAGDASESALLKCIEVCCGSVKEMRERNAKVVEIPFN	363
a1.XP_004853865.1	IAGLCNRAVFQANQENVPILKRAVAGDASESALLKCIELCCGSVKEMRDRYAKIVEIPFN	484
a1.ACB20771.2	IAGLCNRAVFQANQENVPILKRAVAGDASESALLKCIELCCGSVNEMRDRYAKIVEIPFN	484
a1.XP_023557491.1	IAGLCNRAVFQANQENIPILKRAVAGDASESALLKCIELCCGSVKEMRDRYAKIVEIPFN	453
a1.AAA41671.1	IAGLCNRAVFQANQENLPILKRAVAGDASESALLKCIEVCCGSVMEMREKYTKIVEIPFN	483
a1.XP_005076578.1	IAGLCNRAVFQANQENLPILKRTVAGDASESALLKCIEVCCGSVMEMREKYAKIVEIPFN	483
a1+A2823.XP_021504168.1	IAGLCNRAVFQANQENLPILKRAVAGDASESALLKCIEVCCGSVMEMREKYTKIVEIPFN	483
a1.XP_021051287.1	IAGLCNRAVFQANQENLPILKRAVAGDASESALLKCIEVCCGSVMEMREKYSKIVEIPFN	483
a1.XP_021013125.1	IAGLCNRAVFQANQENLPILKRAVAGDASESALLKCIEVCCGSVMEMREKYSKIVEIPFN	483
a1.NP_659149	IAGLCNRAVFQANQENLPILKRAVAGDASESALLKCIEVCCGSVMEMREKYSKIVEIPFN	483
a1.UPI000C2F2801	IAGLCNRAVFQANQENLPILKRAVAGDASESALLKCIELCCGSVKEMRERYAKIVEIPFN	487
a1.XP_020858281.1	IAGLCNRAVFQANQENLPILKRSVAGDASESALLKCIEVCCGSVKEMRDRYTKIVEIPFN	481
a1.XP_004380410.1	IAGLCNRAVFQANQDNLPILKRAVAGDASESALLKCIELCCGSVKEMRERYTKIVEIPFN	481
a1.UPI0001FB338F	IAGLCNRAVFQANQENIPILKRAVAGDASESALLKCIELCCGSVKEMRDRYPKIVEIPFN	481
a1.UPI0002B3D77C	IAGLCNRAVFQANQDNLPILKRAVAGDASESALLKCIEVCCGSVKEMRERYTKIVEIPFN	477
a1.UPI0000124FBE	IAGLCNRAVFQANQDNLPILKRAVAGDASESALLKCIEVCCGSVKEMRERYAKIVEIPFN	481
a1.XP_020747989.1	IAGLCNRAVFQANQDNLPILKRAVAGDASESALLKCIEVCCGSVKEMRERYAKIVEIPFN	481
a1.XP_010587900.1	IAGLCNRAVFQANQENIPILKRAVAGDASESALLKCIELCCGSVKEMREQYTKIVEIPFN	452
a1.XP_020012504.1	IAGLCNRAVFQANQENLPILKRAVAGDASESALLKCIELCCGSVKEMRERYAKIVEIPFN	483
a1.XP_005334975.1	IAGLCNRAVFQANQENLPILKRAVAGDASESALLKCIELCCGSVKEMRDRYAKIVEIPFN	483
a1.XP_024426171.1	IAGLCNRAVFQANQDNLPILKRAVAGDASESALLKCIELCCGSVKEMRERYAKIVEIPFN	481
a1.XP_023975434.1	IAGLCNRAVFQANQENLPILKRAVAGDASESALLKCIELCCGSVKEMRERYTKIVEIPFN	481
a1.XP_022439684.1	IAGLCNRAVFQANQENLPILKRAVAGDASESALLKCIELCCGSVKEMRERYTKIVEIPFN	481
a1.XP_024620662.1	IAGLCNRAVFQANQENLPILKRAVAGDASESALLKCIELCCGSVKEMRERYTKIVEIPFN	481
a1.XP_011283388.1	IAGLCNRAVFQANQENLPILKRAVAGDASESALLKCIELCCGSVKEMRDRYTKIVEIPFN	481
a1.XP_006919736.1	IAGLCNRAVFQANQENLPILKRAVAGDASESALLKCIELCCGSVKEMREQYAKIVEIPFN	452
a1.UPI000C746E0C	IAGLCNRAVFQANQENLPILKRAVAGDASESALLKCIELCCGSVKEMRERYAKIVEIPFN	494
a1.UPI000C2E744C	IAGLCNRAVFQANQENLPILKRAVAGDASESALLKCIELCCGSVKEMRERYTKIVEIPFN	487
a1.XP_020944376.1	IAGLCNRAVFQANQENLPILKRAVAGDASESALLKCIELCCGSVKEMRERYTKIVEIPFN	481
a1.XP_008071711.2	IAGLCNRAVFQANQENLPILKRAVAGDASESALLKCIELCCGSVKEMRDRYAKIVEIPFN	488
a1.UPI000C2E4C26	IAGLCNRAVFQANQENLPILKRAVAGDASESALLKCIELCCGSVKEMRERYAKIVEIPFN	479
a1.XP_012663099.1	IAGLCNRAVFQANQENLPILKRAVAGDASESALLKCIEVCCGSVKEMRERYAKIVEIPFN	483
a1.XP_012617266.1	IAGLCNRAVFQANQENLPILKRAVAGDASESALLKCIELCCGSVKEMRERYAKIVEIPFN	483
a1.XP_012314296.1	IAGLCNRAVFQANQENLPILKRAVAGDASESALLKCIELCCGSVKEMRERYAKIVEIPFN	483
a1.UPI0001C9F9BA	IAGLCNRAVFQANQENLPILKRAVAGDASESALLKCIELCCGSVKEMRERYAKIVEIPFN	483
a1.XP_023078532.1	IAGLCNRAVFQANQENLPILKRAVAGDASESALLKCIELCCGSVKEMRERYAKIVEIPFN	483
a1.NP_000692	IAGLCNRAVFQANQENLPILKRAVAGDASESALLKCIELCCGSVKEMRERYAKIVEIPFN	483
a1.XP_008971666.1	IAGLCNRAVFQANQENLPILKRAVAGDASESALLKCIELCCGSVKEMRERYAKIVEIPFN	452
a1.XP_016780478.1	IAGLCNRAVFQANQENLPILKRAVAGDASESALLKCIELCCGSVKEMRERYAKIVEIPFN	483
a1.ETE67008.1	IAGLCNRAVFQANQENVPILKRAVAGDASESALLKCIELCCGSVKELREKNPKVVEIPFN	482
a1.XP_007435355.1	IAGLCNRAVFQANQENVPILKRAVAGDASESALLKCIELCCGSVKELRDKNPKVVEIPFN	452
a1.XP_020645227.1	IAGLCNRAVFQANQENVPILKRTVAGDASESALLKSIELCCGSVKEQREKNPKVVEIPFN	452
a1.XP_025067531.1	IAGLCNRAVFQANQENVPILKRAVAGDASESALLKCIELCCGSVKEMRGRNPKVAEIPFN	452
a1.XP_005292736.1	VAGLCNRAVFQANQENVPILKRAVAGDASESALLKCIEVCCGSVKEMRERNAKVVEIPFN	484
a1.XP_006132947.1	VAGLCNRAVFQANQENLPILKRAVAGDASESALLKCIEVCCGSVKEMRERNAKVVEIPFN	452
a1.NP_990852.	IAGLCNRAVFQANQENVPILKRAVAGDASESALLKCIELCCGSVKEMRERYPKVVEIPFN	481
a1.XP_021253236.1	VAGLCNRAVFQANQENVPILKRAVAGDASESALLKCIELCCGSVKEMRERYPKVVEIPFN	481
a1.UPI00051ECCC0	VAGLCNRAVFQANQDNVPILKRAVAGDASESALLKCIELCCGSVKEMRERYPKVVEIPFN	476
a1.XP_023796730.1	IAGLCNRAVFQAGQENVPILKRAVAGDASESALLKCIELCCGSVKEMRERYPKVVEIPFN	452
a1.XP_021404823.1	IAGLCNRAVFQAGQENVPILKRAVAGDASESALLKCIELCCGSVKQMRERYPKVVEIPFN	481
a1.UPI0004FDA0CB	VAGLCNRAVFQANQENVPILKRAVAGDASESALLKCIELCCGSVKEMRERYPKVVEIPFN	479
a1.KFW61640.1	VAGLCNRAVFQASQENVPILKRAVAGDASESALLKCIELCCGSVKEMRERYPKVVEIPFN	478
a1.XP_005511501.1	VAGLCNRAVFQANQENVPILKRAVAGDASESALLKCIELCCGSVKEMRERYPKVVEIPFN	452
a1.OPJ66608.1	VAGLCNRAVFQANQENVPILKRAVAGDASESALLKCIELCCGSVKEMRERYPKVVEIPFN	484
a1.sp|P30714.2|	IAGLCNRAVFPAGQENTPILKRDVVGDASESALLKCIELCCGSVKDMREKNQKVAEIPFN	483
a1.NP_989407.1	VAGLCNRAVFQAGQENTPILKRDVAGDASESALLKCIELCCGSVRDMREKNPKVAEIPFN	483
a2.XP_020653823.1	IAGLCNRAVFKAGQENIPISKRDTAGDASESALLKCIELSCGCVRKMRDKSPKVTEIPFN	481
a2.UPI0000124FC0	IAGLCNRAVFKPGQENISISKRDTAGDASESALLKCIQLSCGSVKKMRDKNPKVTEIPFN	478
a2.XP_005293820.1	IAGLCNRAVFKPGQENVSISKRDTAGDASESALLKCIQLSCGSVKKMRDKNPKVTEIPFN	481
a1.KYO43368.1	IAGLCNRAVFKAGQENVSISKRDTAGDASESALLKCIQLSCGSVKKMRDKNPKVTEIPFN	487
a2.XP_006038189.1	IAGLCNRAVFKAGQENVSISKRDTAGDASESALLKCIQLSCGSVKKMRDKNPKVTEIPFN	481
a2.UPI0000F6BCEB	IAGLCNRAVFKVGQEKVAVSKRDTAGDASESALLKCIELSCGSVRKLRDRNPKVAEIPFN	481
a2.XP_023616468.1	IAGLCNRAVFKGGQENISVSKRDTAGDASESALLKCIELSCGSVRKMRDRNPKVAEIPFN	481
a2.UPI000226F4AA	IAGLCNRAVFKAGQENISVSKRDTAGDASESALLKCIELSCGSVRKMRERNPKVAEIPFN	508
a2.UPI00005E9366	IAGLCNRAVFKAGQENISVSKRDTAGDASESALLKCIELSCGSVRKMRERNPKVAEIPFN	481
a2.XP_020835237.1	IAGLCNRAVFKAGQENISVSKRDTAGDASESALLKCIELSCGSVRKMRDRNPKVAEIPFN	481
a2.XP_021537506.1	IAGLCNRAVFKAGQENISVSKRDTAGDASESALLKCIELSCGSVRKMRDRNPKVAEIPFN	542

```
a2.UPI0002B2E326      IAGLCNRAVFKAGQENISVSKRDTAGDASESALIKCIELSCGSVRKMRDRNPKVAEIPFN    474
a2.XP_020024807.1     IAGLCNRAVFKAGQENISVSKRDTAGDASESALIKCIELSCGSVRKMRDRNPKVAEIPFN    481
a2.NP_036637.         IAGLCNRAVFKAGQENISVSKRDTAGDASESALIKCIELSCGSVRKMRDRNPKVAEIPFN    481
a2.XP_004390257.1     IAGLCNRAVFKAGQENISVSKRDTAGDASESALIKCIELSCGSVRKMRDRNPKVAEIPFN    481
a2.XP_003415228.1     IAGLCNRAVFKAGQENISVSKRDTAGDASESALIKCIELSCGSVKKMRERNPKVAEIPFN    481
a2.XP_020740887.1     IAGLCNRAVFKAGQENISVSKRDTAGDASESALIKCIELSCGSVRKMRDRNPKVAEIPFN    481
a2.XP_022415031.1     IAGLCNRAVFKAGQENISVSKRDTAGDASESALIKCIELSCGSVRKMRDRNPKVAEIPFN    481
a2.XP_007129684.1     IAGLCNRAVFKAGQENISVSKRDTAGDASESALIKCIELSCGSVRKMRDRNPKVAEIPFN    481
a2.XP_004448489.1     IAGLCNRAVFKAGQENISVSKRDTAGDASESALIKCIELSCGSVRKMRERNPKVAEIPFN    481
a2.XP_004858786.1     IAGLCNRAVFKAGQENISVSKRDTAGDASESALIKCIELSCGSVRKMRDRNPKVAEIPFN    481
a2.XP_003466610.1     IAGLCNRAVFKAGQENVSVSKRDTAGDASESALIKCIELSCGSVRKMRDRNPKVAEIPFN    481
a2.XP_004639996.1     IAGLCNRAVFKAGQENISVSKRDTAGDASESALIKCIELSCGSVRKMRDRNPKVAEIPFN    481
a2.XP_005339432.1     IAGLCNRAVFKAGQENISVSKRDTAGDASESALIKCIELSCGSVRKMRDRNPKVAEIPFN    481
a2.XP_545753.3so      IAGLCNRAVFKAGQENISVSKRDTAGDASESALIKCIELSCGSVRKMRDRNPKVAEIPFN    481
a2.XP_019677883.4     IAGLCNRAVFKAGQENISVSKRDTAGDASESALIKCIELSCGSVRKMRDRNPKVAEIPFN    481
a2.XP_023069989.1     IAGLCNRAVFKAGQENISVSKRDTAGDASESALIKCIELSCGSVRKMRDRNPKVAEIPFN    390
a2.XP_003795245.1     IAGLCNRAVFKAGQENISVSKRDTAGDASESALIKCIELSCGSVRKMRDRNPKVAEIPFN    481
a2.NP_000693          IAGLCNRAVFKAGQENISVSKRDTAGDASESALIKCIELSCGSVRKMRDRNPKVAEIPFN    481
a2.XP_008056914.2     IAGLCNRAVFKAGQENISVSKRDTAGDASESALIKCIELSCGSVRKMRDRNPKVAEIPFN    481
a2.XP_012604635.1     IAGLCNRAVFKAGQENISVSKRDTAGDASESALIKCIELSCGSVRKMRDRNPKVAEIPFN    481
```
