# Supplementary material for: Bioinformatics approaches for classification and investigation of the evolution of the Na/K-ATPase alpha-subunit
Source: BMC Ecol Evol. 2022 Oct 26;22:122. doi: 10.1186/s12862-022-02071-0 (PMC9609216; doi:10.1186/s12862-022-02071-0)
Supplement: Supplementary file 1 — Additional file 1. Supplementary figures and tables. [file 12862_2022_2071_MOESM1_ESM.zip › Additional file 1 Fig. S5.pdf]

```
F7E0B8-a3           EKSKKVAEIPFNSTNKYQLSIHETEDPNDNRYLLVMKGAPERILDVCSTILIQGKEQPLD
UPI000C732D5F-a3    ERNKKVAEIPFNSTNKYQLSIHETEDPNDNRYLLVMKGAPERILDRCSTILLQGKEQPLD
sp|P13637.3|-a3     ERNKKVAEIPFNSTNKYQLSIHETEDPNDNRYLLVMKGAPERILDRCSTILLQGKEQPLD
XP_012613923.1-a3   ERNKKVAEIPFNSTNKYQLSIHETEDPNDNRYLLVMKGAPERILDRCSTILLQGKEQPLD
UPI0000F6BCEB-a2    DRNPKVAEIPFNSTNKFQLSIHEREDSP-EGHLLVMKGAPERILDRCSTIMLHGAEQPLD
KYO43368.1          DKNPKVTEIPFNSTNKYQLSIHEREDDP-EGYILVMKGAPERILDRCSTILLQGQELPLD
XP_006038189.1-a2   DKNPKVTEIPFNSTNKYQLSIHEREDDP-EGYILVMKGAPERILDRCSTILLQGQELPLD
NP_000693.-a2       DRNPKVAEIPFNSTNKYQLSIHEREDSP-QSHVLVMKGAPERILDRCSTILVQGKEIPLD
XP_012604635.1-a2   DRNPKVAEIPFNSTNKYQLSIHEREDSP-QSHVLVMKGAPERILDRCSTILVQGKEIPLD
sp|P30714.2|-a1     EKNQKVAEIPFNSTNKYQLSVHKNANPSESRYLLVMKGAPERILDRCSSILLQGKEQPLD
NP_989407.1-a1      EKNPKVAEIPFNSTNKYQLSVHKNANPSESRYILVMKGAPERILDRCSTIVLQGKEQPLD
XP_025067531.1-a1   GRNPKVAEIPFNSTNKYQLSIHKNANASESCHLLVMKGAPERILDRCSSILIHGKEQPLD
NP_000692.-a1       ERYAKIVEIPFNSTNKYQLSIHKNPNTSEPQHLLVMKGAPERILDRCSSILLHGKEQPLD
```
