# Supplementary material for: Bioinformatics approaches for classification and investigation of the evolution of the Na/K-ATPase alpha-subunit
Source: BMC Ecol Evol. 2022 Oct 26;22:122. doi: 10.1186/s12862-022-02071-0 (PMC9609216; doi:10.1186/s12862-022-02071-0)
Supplement: Supplementary file 1 — Additional file 1. Supplementary figures and tables. [file 12862_2022_2071_MOESM1_ESM.zip › Additional file 1 Fig. S6.pdf]

```
Ver.NP_0010804          GIVLAAVVIITGCF--------------------------------------SYYQEAK-  155
Ver.F7E0B8              GIVLAAVVIITGCF--------------------------------------SYYQEAK-  155
Ver.UPI000C736357       GIVLAAVVIITGCF--------------------------------------SYYQEAK-  139
Ver.UPI0003CD047A       GIVLAAVVIITGCF--------------------------------------SYYQEAK-  158
Ver.UPI000C73EFA7       GIVLAAVVIITGCF--------------------------------------SYYQEAK-  179
Ver.UPI00049A9E19       GIVLAAVVIITGCF--------------------------------------SYYQEAK-  162
Ver.XP_025028557.1      GIVLAAVVIITGCF--------------------------------------SYYQEAK-  157
Ver.XP_020663591.1      GIVLAAVVIITGCF--------------------------------------SYYQEAK-  218
Ver.UPI0000124FC2       GIVLAAVVIITGCF--------------------------------------SYYQEAK-  140
Ver.UPI000BAD5294       GIVLAAVVIITGCF--------------------------------------SYYQEAK-  139
Ver.XP_020948935.1      GIVLAAVVIITGCF--------------------------------------SYYQEAK-  157
Ver.XP_012613923.1      GIVLAAVVIITGCF--------------------------------------SYYQEAK-  143
Ver.UPI000C2D7C35       GIVLAAVVIITGCF--------------------------------------SYYQEAK-  160
Ver.XP_006903931.1      GIVLAAVVIITGCF--------------------------------------SYYQEAK-  113
Ver.XP_023380497.1      GIVLAAVVIITGCF--------------------------------------SYYQEAK-  214
Ver.UPI0002B3612F       GIVLAAVVIITGCF--------------------------------------SYYQEAK-  153
Ver.XP_020726792.1      GIVLAAVVIITGCF--------------------------------------SYYQEAK-  108
Ver.ELK32312.1          GIVLAAVVIITGCF--------------------------------------SYYQEAK-  182
Ver.UPI000226419C       GIVLAAVVIITGCF--------------------------------------SYYQEAK-  158
Ver.UPI000C7286EF       GIVLAAVVIITGCF--------------------------------------SYYQEAK-  143
Ver.UPI0007A6EC9C       GIVLAAVVIITGCF--------------------------------------SYYQEAK-  143
Ver.UPI000C2DAA95       GIVLAAVVIITGCF--------------------------------------SYYQEAK-  149
Ver.UPI000C740E55       GIVLAAVVIITGCF--------------------------------------SYYQEAK-  154
Ver.UPI000CB4CAB6       GIVLAAVVIITGCF--------------------------------------SYYQEAK-  154
Ver.PNJ19200.1          GIVLAAVVIITGCF--------------------------------------SYYQEAK-  143
Ver.XP_021021704.1      GIVLAAVVIITGCF--------------------------------------SYYQEAK-  156
Ver.UPI00035B05DE       GIVLAAVVIITGCF--------------------------------------SYYQEAK-  178
Ver.XP_023600635.1      GIVLAAVVIITGCF--------------------------------------SYYQEAK-  122
Ver.UPI000C732D5F       GIVLAAVVIITGCF--------------------------------------SYYQEAK-  154
Ver.PNI95395.1          GIVLAAVVIITGCF--------------------------------------SYYQEAK-  143
Ver.NP_036638.          GIVLAAVVIITGCF--------------------------------------SYYQEAK-  143
Ver.XP_022441242.1      GIVLAAVVIITGCF--------------------------------------SYYQEAK-  157
Ver.UPI000651771D       GIVLAAVVIITGCF--------------------------------------SYYQEAK-  143
Ver.UPI000C7355ED       GIVLAAVVIITGCF--------------------------------------SYYQEAK-  168
Ver.XP_021590883.1      GIVLAAVVIITGCF--------------------------------------SYYQEAK-  180
Ver.XP_021106581.1      GIVLAAVVIITGCF--------------------------------------SYYQEAK-  156
Ver.XP_024433413.1      GIVLAAVVIITGCF--------------------------------------SYYQEAK-  143

Ver.UPI000C2EDFE7       GIVLAAVVIITGCF--------------------------------------SYYQEAK-  154
Ver.UPI000C2E3154       GIVLAAVVIITGCF--------------------------------------SYYQEAK-  156
Ver.XP_023507169.1      GIVLAAVVIITGCF--------------------------------------SYYQEAK-  144
Ver.KFO33633.1          GIVLAAVVIITGCF--------------------------------------SYYQEAK-  113
Ver.UPI000C71DF25       GIVLAAVVIITGCF--------------------------------------SYYQEAK-  154
Ver.UPI0001914BDE       GIVLAAVVIITGCF--------------------------------------SYYQEAK-  156
Ver.XP_003799510.1      GIVLAAVVIITGCF--------------------------------------SYYQEAK-  156
Ver.XP_008065591.1      GIVLAAVVIITGCF--------------------------------------SYYQEAK-  113
Ver.sp|P13637.3|        GIVLAAVVIITGCF--------------------------------------SYYQEAK-  143
Ver.KPP65694.1          GIVLSAVVIITGCF--------------------------------------SYFQEAK-  117
Ver.XP_023665796.1      GIVLSAVVIITGCF--------------------------------------SYFQEAK-  152
Ver.XP_015461719.2      GIVLSAVVIITGCF--------------------------------------SYFQEAK-  154
Ver.BAB60722.1          GIVLSAVVIITGCF--------------------------------------SYFQEAE-  152
Ver.UPI000054C9F5       GIVLSAVVIITGCF--------------------------------------SYFQEAK-  153
Ver.UPI0004E4D157       GIVLSAVVVVTGCF--------------------------------------SYFQEAK-  152
Ver.UPI000293B6B0       GIVLSAVVVVTGCF--------------------------------------SYFQEAK-  152
Ver.UPI0000E3AF2C       GIVLSAVVIITGCF--------------------------------------SYFQEAK-  152
Ver.XP_024920682.1      GIVLSAVVIITGCF--------------------------------------SYFQEAK-  153
Ver.UPI0003B0448B       GIVLSAVVIITGCF--------------------------------------SYFQEAK-  152
Ver.UPI00016E235F       GIVLSAVVIITGCF--------------------------------------SYFQEAK-  155
Ver.UPI00032B6FE9       GIVLSAVVIITGCF--------------------------------------SYFQEAK-  153
Ver.XP_004074116.1      GIVLSAVVIITGCF--------------------------------------SYFQEAK-  152
Ver.KKF19362.1          GIVLSAVVIITGCF--------------------------------------SYFQEAK-  166
Ver.XP_012711044.2      GIVLSAVVIITGCF--------------------------------------SYFQEAK-  152
Ver.UPI00025F91A4       GIVLSAVVIITGCF--------------------------------------SYFQEAK-  152
Ver.XP_020793662.1      GIVLSAVVIITGCF--------------------------------------SYFQEAK-  152
Ver.XP_024153267.1      GIVLSAVVIITGCF--------------------------------------SYFQEAK-  152
Ver.XP_023117914.1      GIVLSAVVIITGCF--------------------------------------SYFQEAK-  152
Ver.XP_022053465.1      GIVLSAVVIITGCF--------------------------------------SYFQEAK-  152
Ver.NP_571759.2         GIVLSAVVIITGCF--------------------------------------SYFQEAK-  153
Ver.W5UML4              GIVLSAVVIITGCF--------------------------------------SYFQDAK-  154
Ver.W5L4G0              GIVLSAVVIITGCF--------------------------------------SYFQEAK-  152
Ver.UPI0005D90DB9       GIVLSAVVIITGCF--------------------------------------SYFQEAK-  152
```

```
Ver.XP_021427657.1        GIVLSAVVIITGCF--------------------------------------SYFQEAK-  158
Ver.XP_024297426.1        GIVLSAVVIITGCF--------------------------------------SYFQEAK-  163
Ver.UPI00001DFF47         GIVLSVVVVVVTGCF--------------------------------------SYFQEAK-  141
Ver.UPI0006B7181A         GIVLSVVVVVVTGCF--------------------------------------SYFQEAK-  154
Ver.UPI00079DB5F5         GIVLTAVVVITGCF--------------------------------------SYYQEAK-  139
Ver.UPI000050D2B6         GIVLTAVVVITGCF--------------------------------------SYFQEAK-  138
Ver.UPI0007F716B9         GIVLTAVVVITGCF--------------------------------------SYFQEAK-  139
Ver.UPI0000E9CD46         GIVLTAVVVITGCF--------------------------------------SYFQEAK-  151
Ver.UPI0007F7EA5B         GIVLSAVVIITGCF--------------------------------------SYFQEAK-  152
Ver.UPI00079E2EF3         GIVLTAVVVITGCF--------------------------------------SYFQEAK-  139
Ver.UPI0004448FEC         GIVLTAVVVITGCF--------------------------------------SYFQEAK-  139
Ver.XP_023187147.1        GIVLTAVVVITGCF--------------------------------------SYFQEAK-  152
Ver.UPI0006B30A18         GIVLTAVVVITGCF--------------------------------------SYFQEAK-  154
Ver.XP_017270842.1        GIVLTAVVVITGCF--------------------------------------SYFQEAK-  154
Ver.T2B507                GIVLTAVVVITGCF--------------------------------------SYFQEAK-  140
Ver.XP_020466584.1        GIVLTAVVVITGCF--------------------------------------SYFQEAK-  153
Ver.UPI0000124FC4         GIVLTAVVVITGCF--------------------------------------SYFQEAK-  140
Ver.XP_004550929.1        GIVLTAVVVITGCF--------------------------------------SYFQEAK-  153
Ver.UPI00025FB25F         GIVLTAVVVITGCF--------------------------------------SYFQEAK-  140
Ver.UPI0000E3A2FA         GIVLTAVVVITGCF--------------------------------------SYFQEAK-  153
Ver.UPI00003628C3         GIVLTAVVIITGCF--------------------------------------SYFQEAK-  150
Ver.AGR87394.1            GIVLTAVVIITGCF--------------------------------------SYFQEAK-  139
Ver.XP_020504733.1        GIVLTAVVVITGCF--------------------------------------SYFQEAK-  155
Ver.AHB86586.1            GIVLTAVVIITGCF--------------------------------------SYFQEAK-  140
Ver.UPI00032B9010         GIVLTAVVIITGCF--------------------------------------SYFQEAK-  140
Ver.XP_022612296.1        GIVLTAVVIITGCF--------------------------------------SYFQEAK-  140
Ver.XP_023285663.1        GIVLTAVVIITGCF--------------------------------------SYFQEAK-  153
Ver.ELK38498.1            GIVLAVVVFITGCF--------------------------------------SYFQEAK-  113
Ver.XP_545754.3           GMVLAIVVMITGCF--------------------------------------SYYQEAK-  163
Ver.Q98SL3                GVVLAAVVIITGCF--------------------------------------SYYQESK-  139
Ver.NP_0011179            GVVLSAVVIITGCF--------------------------------------SYYQEAK-  143
Ver.UPI0006B74D9B         GVVLSAVVIITGCF--------------------------------------SYYQEAK-  142
Ver.XP_020796901.1        GVVLAAVVIITGCF--------------------------------------SYFQEAK-  185
Ver.NP_571758.1           GVVLSAVVIITGCF--------------------------------------SYYQEAK-  148
Ver.XP_005796664.1        GVVLAAVVIITGCF--------------------------------------SYFQESK-  141
Ver.UPI00000FE1CF         GVVLAAVVIVTGCF--------------------------------------SYFQEAK-  139

Ver.AHB86585.1            GVVLSAVVIITGCF--------------------------------------SYFQEAK-  140
Ver.KKF24497.1            GVVLAAVVIITGCF--------------------------------------SYFQEAK-  141
Ver.XP_020507674.1        ------------------------------------------------------------  0
Ver.XP_023135081.1        GVVLAAVVIITGCF--------------------------------------SYFQEAK-  141
Ver.BAO02373.1            GVVLAAVVIITGCF--------------------------------------SYFQEAK-  141
Ver.UPI0003D8328F         GIVLAGVVIITGCF--------------------------------------SYYQEAK-  149
Ver.NP_0010831            GVVLAAVVIVTGCF--------------------------------------SYYQEAK-  151
Ver.UPI0000F6BCEB         GVVLAAVVIVTGCF--------------------------------------SYYQEAK-  151
Ver.ELK38499.1            GVVLAAVVIVTGCF--------------------------------------SYYQEAK-  151
Ver.EPQ02424.1            GVVLAAVVIVTGCF--------------------------------------SYYQEAK-  179
Ver.XP_023616468.1        GVVLAAVVIVTGCF--------------------------------------SYYQEAK-  151
Ver.XP_021054982.1        GIVLAAVVIVTGCF--------------------------------------SYYQEAK-  151
Ver.NP_036637.            GIVLAAVVIVTGCF--------------------------------------SYYQEAK-  151
Ver.XP_004858786.1        GVVLAAVVIVTGCF--------------------------------------SYYQEAK-  151
Ver.XP_003466610.1        GVVLAAVVIVTGCF--------------------------------------SYYQEAK-  151
Ver.XP_004639996.1        GVVLAAVVIVTGCF--------------------------------------SYYQEAK-  151
Ver.XP_005339432.1        GVVLAAVVIVTGCF--------------------------------------SYYQEAK-  151
Ver.XP_003795245.1        GVVLAAVVIVTGCF--------------------------------------SYYQEAK-  151
Ver.XP_008056914.2        GVVLAAVVIVTGCF--------------------------------------SYYQEAK-  151
Ver.XP_012604635.1        GVVLAAVVIVTGCF--------------------------------------SYYQEAK-  151
Ver.XP_004390257.1        GVVLAAVVIVTGCF--------------------------------------SYYQEAK-  151
Ver.XP_020024807.1        GVVLAAVVIVTGCF--------------------------------------SYYQEAK-  151
Ver.NP_0011253            GVVLAAVVIVTGCF--------------------------------------SYYQEAK-  151
Ver.XP_023069989.1        GVVLAAVVIVTGCF--------------------------------------SYYQEAK-  60
Ver.PNI19721.1            GVVLAAVVIVTGCF--------------------------------------SYYQEAK-  151
Ver.NP_000693.            GVVLAAVVIVTGCF--------------------------------------SYYQEAK-  151
Ver.NP_0012526            GVVLAAVVIVTGCF--------------------------------------SYYQEAK-  151
Ver.XP_021537506.1        GVVLAAVVIVTGCF--------------------------------------SYYQEAK-  212
Ver.UPI0002B2E326         GVVLAAVVIVTGCF--------------------------------------SYYQEAK-  144
Ver.XP_003415228.1        GVVLAAVVIVTGCF--------------------------------------SYYQEAK-  151
Ver.XP_004448489.1        GVVLAAVVIVTGCF--------------------------------------SYYQEAK-  151
Ver.XP_006922964.1        GVVLAAVVIVTGCF--------------------------------------SYYQEAK-  151
Ver.XP_545753.3           GVVLAAVVIVTGCF--------------------------------------SYYQEAK-  151
Ver.XP_019677883.4        GVVLAAVVIVTGCF--------------------------------------SYYQEAK-  151
Ver.NP_0011650            GVVLAAVVIVTGCF--------------------------------------SYYQEAK-  151
```

Ver.NP_0010749	GVVLAAVVIVTGCF---------------------------------------SYYQEAK-	151
Ver.XP_020740887.1	GVVLAAVVIVTGCF---------------------------------------SYYQEAK-	151
Ver.XP_022415031.1	GVVLAAVVIVTGCF---------------------------------------SYYQEAK-	151
Ver.XP_007129684.1	GVVLAAVVIVTGCF---------------------------------------SYYQEAK-	151
Ver.UPI000226F4AA	GVVLAAVVIVTGCF---------------------------------------SYYQEAK-	151
Ver.UPI00005E9366	GVVLAAVVIVTGCF---------------------------------------SYYQEAK-	151
Ver.XP_020835237.1	GVVLAAVVIVTGCF---------------------------------------SYYQEAK-	151
Ver.XP_020653823.1	GVVLAAVVIVTGCF---------------------------------------SYYQEAK-	151
Ver.XP_026576074.1	GVVLAAVVIVTGCF---------------------------------------SYYQEAK-	151
Ver.UPI0000124FC0	GVVLAAVVIVTGCF---------------------------------------SYYQEAK-	148
Ver.XP_005293820.1	GVVLAAVVIVTGCF---------------------------------------SYYQEAK-	151
Ver.KYO43368.1	GVVLAAVVIVTGCF---------------------------------------SYYQEAK-	157
Ver.XP_006038189.1	GVVLAAVVIVTGCF---------------------------------------SYCQEAK-	151
Ver.XP_023390675.1	GVVLSAVVIITGCF---------------------------------------SYYQEAK-	122
Ver.ELK30843.1	GVVLSAVVIITGCF---------------------------------------SYYQEAK-	160
Ver.EMP33651.1	GVVLAAVVIITGCF---------------------------------------SYYQEAK-	33
Ver.UPI000056D0DB	GVVLSTVVIVTGCF---------------------------------------SYYQEAK-	158
Ver.AHD24596.1	GIVLSTVVIITGCF---------------------------------------SYYQEAK-	154
Ver.UPI00001261C4	GVVLSTVVIITGCF---------------------------------------SYYQEAK-	153
Ver.UPI000C2F2801	GVVLSAVVIITGCF---------------------------------------SYYQEAK-	157
Ver.NP_571763.1	GLVLAFVVTVNGCF---------------------------------------SYYQEAK-	154
Ver.UPI00025FADDE	GIVLAAVVIITACF---------------------------------------SYYQEAK-	153
Ver.UPI0000318264	GVVLSAVVIITGCF---------------------------------------SYYQEAK-	153
Ver.UPI0006B827F3	GVVLSVVVIVTGCF---------------------------------------SYYQEAK-	188
Ver.XP_004066575.1	GIVLSAVVIITGCF---------------------------------------SYYQEAK-	152
Ver.XP_024144685.1	GIVLSAVVIITGCF---------------------------------------SYYQEAK-	152
Ver.UPI0006B31231	GIVLSAVVIITGCF---------------------------------------SYYQEAK-	152
Ver.UPI0006B37238	GVVLSAVVIITGCF---------------------------------------SYYQEAK-	155
Ver.ADD60471.1	GVVLSAVVIITGCF---------------------------------------SYYQEAK-	152
Ver.UPI000157ACC7	GVVLSAVVIITGCF---------------------------------------SYYQEAK-	155
Ver.AAT48993.1	GVVLSAVVIITGCF---------------------------------------SYYQEAK-	153
Ver.XP_020476182.1	GVVLSAVVIITGCF---------------------------------------SYYQEAK-	156
Ver.UPI00079E81D7	GIVLSAVVIITGCF---------------------------------------SYYQEAK-	153
Ver.UPI000274DF5C	GVVLSAVVIITGCF---------------------------------------SYYQEAK-	146
Ver.NP_0012969	GIVLSAVVIITGCF---------------------------------------SYYQEAK-	153
Ver.UPI000443A733	GIVLSAVVIITGCF---------------------------------------SYYQEAK-	153
Ver.XP_023185631.1	GIVLSAVVIITGCF---------------------------------------SYYQEAK-	153
Ver.ALA65287.2	GVVLSAVVIITGCF---------------------------------------SYYQEAK-	152
Ver.AKQ12834.1	GVVLSAVVIITGCF---------------------------------------SYYQEAK-	155
Ver.XP_023275950.1	GVVLSAVVIITGCF---------------------------------------SYYQEAK-	154
Ver.ADB03120.1	GIVLSAVVIITGCF---------------------------------------SYYQEAK-	153
Ver.UPI00022B0848	GIVLSAVVIITGCF---------------------------------------SYYQEAK-	153
Ver.AGZ87948.1	GIVLSAVVIITGCF---------------------------------------SYYQEAK-	153
Ver.sp|Q9YH26.2|	GIVLSAVVIITGCF---------------------------------------SYYQEAK-	153
Ver.AGO02179.1	GIVLSAVVIITGCF---------------------------------------SYYQEAK-	153
Ver.AHB86584.1	GVVLSAVVIITGCF---------------------------------------SYYQEAK-	153
Ver.XP_023121557.1	GVVLSAVVIITGCF---------------------------------------SYYQEAK-	153
Ver.XP_022078036.1	GVVLSAVVIITGCF---------------------------------------SYYQEAK-	153
Ver.XP_020792263.1	GVVLSAVVIITGCF---------------------------------------SYYQEAK-	154
Ver.AGR87393.1	GVVLSAVVIITGCF---------------------------------------SYYQEAK-	154
Ver.ABF58911.1	GIVLSAVVMITGCF---------------------------------------SYYQEAK-	154
Ver.Q90X33	GIVLAGVVIITGCF---------------------------------------SYYQEAK-	154
Ver.XP_022530668.1	GIVLSAVVMITGCF---------------------------------------SYYQEAK-	154
Ver.AJR20271.1	GIVLSAVVIITGCF---------------------------------------SYYQEAK-	170
Ver.XP_020328431.1	GVVLSAVVIVTGCF---------------------------------------SYYQEAK-	155
Ver.XP_021426673.1	GVVLSAVVIVTGCF---------------------------------------SYYQEAK-	155
Ver.XP_023653512.1	GVVLSAVVIITGCF---------------------------------------SYYQEAK-	154
Ver.sp|Q92030.1|	GVVLSAVVIITGCF---------------------------------------SYYQEAK-	152
Ver.ALB35496.1	GVVLSAVVIITGCF---------------------------------------SYYQEAK-	152
Ver.NP_571761.1	GIVLSAVVMITGCF---------------------------------------SYYQEAK-	156
Ver.AJR20270.1	GIVLSAVVMITGCF---------------------------------------SYYQEAK-	156
Ver.XP_023690671.1	GVVLSAVVVITGCF---------------------------------------SYYQEAK-	154
Ver.XP_008322794.1	GVVLSAVVIITGCF---------------------------------------SYYQEAK-	185
Ver.XP_017282368.1	GVVLSAVVIITGCF---------------------------------------SYYQEAK-	158
Ver.XP_004066573.1	GVVLSAVVIITGCF---------------------------------------SYYQEAK-	154
Ver.XP_024144684.1	GVVLSAVVIITGCF---------------------------------------SYYQEAK-	154
Ver.XP_020497843.1	GVVLSAVVIITGCF---------------------------------------SYYQEAK-	166
Ver.UPI000027C768	GVVLSAVVIITGCF---------------------------------------SYYQEAK-	149
Ver.XP_004571307.1	GVVLSAVVIITGCF---------------------------------------SYYQEAK-	154
Ver.XP_012714443.1	GVVLSAVVIITGCF---------------------------------------SYYQEAK-	154
Ver.XP_023185013.1	GVVLSAVVIITGCF---------------------------------------SYYQEAK-	154

```
Ver.UPI00066EFDEA        GVVLSAVVIITGCF---------------------------------------SYYQEAK-  158
Ver.XP_022617258.1       GVVLSAVVIITGCF---------------------------------------SYYQEAK-  154
Ver.BAN17691.1           GVVLSAVVIITGCF---------------------------------------SYYQEAK-  154
Ver.sp|P25489.1|         GVVLSAVVIITGCF---------------------------------------SYYQDAK-  156
Ver.XP_022536277.1       GVVLSAVVIITGCF---------------------------------------SYYQEAK-  154
Ver.Q9DEU1               GVVLSAVVIITGCF---------------------------------------SYYQEAK-  154
Ver.AGR45921.1           GIVLAAVVIITGCF---------------------------------------SYYQEAK-  154
Ver.sp|P30714.2|         GVVLSAVVIITGCF---------------------------------------SYYQEAK-  153
Ver.NP_0010840           GVVLSAVVIITGCF---------------------------------------SYYQEAK-  155
Ver.NP_989407.1          GVVLSAVVIITGCF---------------------------------------SYYQEAK-  153
Ver.XP_004853865.1       GVVLSAVVIITGCF---------------------------------------SYYQEAK-  154
Ver.ACB20771.2           GVVLSAVVIITGCF---------------------------------------SYYQEAK-  154
Ver.XP_023557491.1       GVVLSAVVIITGCF---------------------------------------SYYQEAK-  123
Ver.ERE90024.1           GVVLSAVVIITGCF---------------------------------------SYYQEAK-  173
Ver.XP_005076578.1       GVVLSAVVIITGCF---------------------------------------SYYQEAK-  153
Ver.AAA41671.1           GVVLSAVVIITGCF---------------------------------------SYYQEAK-  153
Ver.XP_021504168.1       GVVLSAVVIITGCF---------------------------------------SYYQEAK-  153
Ver.XP_021051287.1       GVVLSAVVIITGCF---------------------------------------SYYQEAK-  153
Ver.XP_021013125.1       GVVLSAVVIITGCF---------------------------------------SYYQEAK-  153
Ver.NP_659149.           GVVLSAVVIITGCF---------------------------------------SYYQEAK-  153
Ver.EPQ03777.1           GVVLSAVVIITGCF---------------------------------------SYYQEAK-  187
Ver.XP_020858281.1       GVVLSAVVIITGCF---------------------------------------SYYQEAK-  151
Ver.XP_004380410.1       GVVLSAVVIITGCF---------------------------------------SYYQEAK-  151
Ver.AGY54951.1           GVVLSAVVIITGCF---------------------------------------SYYQEAK-  151
Ver.UPI0001FB338F        GVVLSAVVIITGCF---------------------------------------SYYQEAK-  151
Ver.NP_0011565           GVVLAAVVIITGCF---------------------------------------SYYQEAK-  153
Ver.XP_010587900.1       GVVLSAVVIITGCF---------------------------------------SYYQEAK-  122
Ver.UPI0002B3D77C        GVVLSAVVIITGCF---------------------------------------SYYQEAK-  147
Ver.NP_0010702           GVVLSAVVIITGCF---------------------------------------SYYQEAK-  151
Ver.OWK04910.1           GVVLSAVVIITGCF---------------------------------------SYYQEAK-  122
Ver.UPI0000124FBE        GVVLSAVVIITGCF---------------------------------------SYYQEAK-  151
Ver.NP_0010093           GVVLSAVVIITGCF---------------------------------------SYYQEAK-  151

Ver.XP_020747989.1       GVVLSAVVIITGCF---------------------------------------SYYQEAK-  151
Ver.XP_020012504.1       GVVLSAVVIITGCF---------------------------------------SYYQEAK-  153
Ver.XP_005334975.1       GVVLSAVVIITGCF---------------------------------------SYYQEAK-  153
Ver.NP_0010033           GVVLSAVVIITGCF---------------------------------------SYYQEAK-  151
Ver.XP_022352592.1       GVVLSAVVIITGCF---------------------------------------SYYQEAK-  151
Ver.XP_011283388.1       GVVLSAVVIITGCF---------------------------------------SYYQEAK-  151
Ver.XP_024426171.1       GVVLSAVVIITGCF---------------------------------------SYYQEAK-  151
Ver.XP_023975434.1       GVVLSAVVIITGCF---------------------------------------SYYQEAK-  151
Ver.XP_022439684.1       GVVLSAVVIITGCF---------------------------------------SYYQEAK-  151
Ver.XP_024620662.1       GVVLSAVVIITGCF---------------------------------------SYYQEAK-  151
Ver.XP_006919736.1       GVVLSAVVIITGCF---------------------------------------SYYQEAK-  122
Ver.UPI000C746E0C        GVVLSAVVIITGCF---------------------------------------SYYQEAK-  164
Ver.UPI000C2E744C        GVVLSAVVIITGCF---------------------------------------SYYQEAK-  157
Ver.XP_020944376.1       GVVLSAVVIITGCF---------------------------------------SYYQEAK-  151
Ver.XP_008071711.2       GVVLSAVVIITGCF---------------------------------------SYYQEAK-  158
Ver.XP_012663099.1       GVVLSAVVIITGCF---------------------------------------SYYQEAK-  153
Ver.UPI000C2E4C26        GVVLSAVVIITGCF---------------------------------------SYYQEAK-  149
Ver.XP_012617266.1       GVVLSAVVIITGCF---------------------------------------SYYQEAK-  153
Ver.XP_012314296.1       GVVLSAVVIITGCF---------------------------------------SYYQEAK-  153
Ver.UPI0001C9F9BA        GVVLSAVVIITGCF---------------------------------------SYYQEAK-  153
Ver.NP_000692.           GVVLSAVVIITGCF---------------------------------------SYYQEAK-  153
Ver.XP_008971666.1       GVVLSAVVIITGCF---------------------------------------SYYQEAK-  122
Ver.XP_016780478.1       GVVLSAVVIITGCF---------------------------------------SYYQEAK-  153
Ver.XP_023078532.1       GVVLSAVVIITGCF---------------------------------------SYYQEAK-  153
Ver.NP_0012536           GVVLSAVVIITGCF---------------------------------------SYYQEAK-  153
Ver.PNJ46178.1           GVVLSAVVIITGCF---------------------------------------SYYQEAK-  153
Ver.ETE67008.1           GVVLAAVVIITGCF---------------------------------------SYYQEAK-  152
Ver.XP_007435355.1       GVVLAAVVIITGCF---------------------------------------SYYQEAK-  122
Ver.XP_020645227.1       GVVLSAVVIITGCF---------------------------------------SYYQEAK-  122
Ver.XP_006132947.1       GVVLAAVVIITGCF---------------------------------------SYYQEAK-  122
Ver.XP_005292736.1       GVVLAAVVIITGCF---------------------------------------SYYQEAK-  154
Ver.XP_024064252.1       GVVLAAVVIITGCF---------------------------------------SYYQEAK-  154
Ver.XP_025067531.1       GIVLAAVVIITGCF---------------------------------------SYYQEAK-  122
Ver.NP_990852.           GVVLAAVVIITGCF---------------------------------------SYYQEAK-  151
Ver.XP_021253236.1       GVVLAAVVIITGCF---------------------------------------SYYQEAK-  151
Ver.UPI00051ECCC0        GIVLSAVVIVTGCF---------------------------------------SYYQEAK-  146
Ver.KFU90062.1           GVVLAAVVIITGCF---------------------------------------SYYQEAK-  148
Ver.XP_030317504.1       GVVLAAVVIITGCF---------------------------------------SYYQEAK-  148
Ver.KFP84941.1           GVVLSAVVIVTGCF---------------------------------------SYYQEAK-  148
```

Ver.XP_030327328.1	GVVLAAVVIITGCF--------------------------------------SYYQEAK-	151
Ver.XP_023796730.1	GIVLAAVVIITGCF--------------------------------------SYYQEAK-	122
Ver.XP_021404823.1	GIVLAAVVIITGCF--------------------------------------SYYQEAK-	151
Ver.XP_025966334.1	GIVLAAVVIITGCF--------------------------------------SYYQEAK-	151
Ver.UPI0004FDA0CB	GVVLAAVVIITGCF--------------------------------------SYYQEAK-	149
Ver.KQK85052.1	GVVLAAVVIITGCF--------------------------------------SYYQEAK-	160
Ver.NP_0012973	GIVLAAVVIITGCF--------------------------------------SYYQEAK-	153
Ver.KFW61640.1	GVVLAAVVIITGCF--------------------------------------SYYQEAK-	148
Ver.XP_005511501.1	GVVLAAVVIITGCF--------------------------------------SYYQEAK-	122
Ver.OPJ66608.1	GVVLAAVVIITGCF--------------------------------------SYYQEAK-	154
Art.EFX69525.1(Group II)	SIILILLVIGTTIF--------------------------------------AYYQERQ-	134
Rinv.CRX73232.1(Group II)	GTALAVVVLVTGIF--------------------------------------SYYQDSK-	122
Prt.XP_001742517.1	GIVLAAVVIITGVF--------------------------------------SYFQEGR-	160
Prt.EGD73524.1	GIVLAAVVVITGCF--------------------------------------SYYQEGR-	255
Rinv.PIS80793.1	GTVLVVVVVISGIF--------------------------------------TYYQESK-	165
Ver.ACB20770.2	GVVLSAVVIITGCF--------------------------------------SYYQEAK-	141
Art.AAF17586.1	GIALTVLVIVTGLF--------------------------------------TYFQVHK-	142
Art.AFU25666.1(Group II)	GTVLVLVCVITGVF--------------------------------------AYSQEAK-	137
Art.AFU25665.1	GVVLVAVVIITGIF--------------------------------------SYYQQAK-	178
Art.XP_021915175.1	GIVLAAVVIVTGIF--------------------------------------SYYQESK-	173
Art.EFN85240.1	GIVLAAVVIVTGIF--------------------------------------SYYQESK-	210
Art.OXA63786.1(Group II)	GLVLTIVVLITGIF--------------------------------------SFYQESK-	133
Art.ODN01960.1(Group II)	GLVLTVVVVVVTGIF-------------------------------------SYYQESK-	139
Rinv.XP_020602016.1	--	0
Rinv.G4VGA0	GIVLSVVVVVTGCF--------------------------------------SYYQESK-	149
Art.ODM98837.1	GSVLVGVVIITGIF--------------------------------------SYLQEAK-	132
Art.AGZ13696.1-	GVVLTGVVVVTGVF--------------------------------------SYYQERK-	149
Rinv.KXJ20388.1	GLTLTIVVIITGIF--------------------------------------SYYQESK-	159
Nem.CDW54807.1	GIVLAAVVIITGCF--------------------------------------QYYQEAK-	108
Nem.KRZ50957.1	GVVLAAVVIITGCF--------------------------------------QYYQEAK-	194
Nem.KRX38989.1	GVVLAAVVIITGCF--------------------------------------QYYQEAK-	194
Nem.KRY10799.1	GVVLAAVVIITGCF--------------------------------------QYYQEAK-	223
Nem.KRY70033.1	GVVLAAVVIITGCF--------------------------------------QYYQEAK-	161
Nem.KRZ74907.1	GVVLAAVVIITGCF--------------------------------------QYYQEAK-	161
Nem.KRZ04306.1	GVVLAAVVIITGCF--------------------------------------QYYQEAK-	161
Nem.KRX24043.1	GVVLAAVVIITGCF--------------------------------------QYYQEAK-	194
Nem.KRY48181.1	GVVLAAVVIITGCF--------------------------------------QYYQEAK-	161
Nem.KRY33278.1	GVVLAAVVIITGCF--------------------------------------QYYQEAK-	194
Rinv.OQV18895.1(Group II)	GCVLAGVVFLTGCF--------------------------------------SFFQRAK-	147
Nem.XP_003369418.1	GTVLAVVVIVTGCF--------------------------------------QYYQEAK-	197
Nem.UPI0007A1BF54	GIVLMTVVVITGCF--------------------------------------QYYQESK-	163
Nem.UPI000183E9C3	GIVLMTVVVITGVF--------------------------------------QYYQESK-	152
Nem.UPI0006101D56	GIVLMTVVVITGCF--------------------------------------QYYQESK-	101
Nem.UPI000601D02E	GIVLMAVVIITGCF--------------------------------------QYYQESK-	130
Nem.UPI00060CD0E5	GIVLMAVVIITGCF--------------------------------------QYYQESK-	130
Nem.UPI00060630B9	GLVLMGVVIITGCF--------------------------------------QYYQESK-	186
Nem.UPI00060EAC2D	GIVLMTVVIIGGVF--------------------------------------QYYQESK-	129
Nem.UPI000609E432	GLVLMCVVIITGVF--------------------------------------QYYQENK-	130
Nem.UPI000609F1C5	GIVLMSVVIITGCF--------------------------------------QYYQENK-	130
Ver.NP_835200.1	GIVLTFVVTVNGCF--------------------------------------SYSQEAK-	153
Ver.NP_571762.1	GLVLAFVVIVNGWF--------------------------------------SFYQESK-	153
Ver.BAJ13363.1	GLVLAVVVIITGCF--------------------------------------SYYQEAK-	158
Ver.UPI00001DFF4A	GLVLAVVVIITGCF--------------------------------------SYYQEAK-	158
Ver.UPI00002BAA33	GVVLAAVVIITGCF--------------------------------------SYFQEAK-	139
Ver.XP_021506251.1	GFVLAAVVIITGCF--------------------------------------SYYQESK-	161
Ver.NP_074039.	GIVLSAVVIITGCF--------------------------------------SYYQEAK-	159
Ver.XP_021014708.1	GIVLSAVVVITGCF--------------------------------------SYYQEAK-	163
Ver.NP_038762.	GIVLSAVVIITGCF--------------------------------------SYYQEAK-	163
Ver.XP_020024800.1	GAVLAVVVIVTGCF--------------------------------------SYYQEAK-	156
Ver.XP_003795244.1	GCVLVLVVVITGCF--------------------------------------SYYQESK-	162
Ver.XP_004639995.1	GIVLAVVVAITGCF--------------------------------------SYYQEAK-	167
Ver.XP_004448484.1	GIVLSIVVIITGCF--------------------------------------SYYQEAK-	162
Ver.XP_023103614.1	GIVLSFVVVVTGCF--------------------------------------SYYQEAK-	163
Ver.XP_023496657.1	GIVLAVVVFITGCF--------------------------------------SYYQEAK-	168
Ver.XP_021537588.1	GIVLAVVVIVTGCF--------------------------------------SYYQEAK-	162
Ver.XP_006096963.1	GFVLAVVVIITGCF--------------------------------------SYYQEAK-	163
Ver.XP_006922963.1	GIVLAVVVVITGCF--------------------------------------SYSQEAK-	162
Ver.XP_011371380.1	GIVLAVVVVITGCF--------------------------------------SYSQEAK-	162
Ver.NP_0011375	GIVLTVVVIITGCF--------------------------------------SYYQEAK-	162
Ver.XP_020740848.1	GIVLTVVVVITGCF--------------------------------------SYYQEAK-	162
Ver.XP_010593170.2	GIVLSIVVIITGCF--------------------------------------SYYQEAK-	122

```
Ver.XP_021568356.1          GFVLSIVVVITGCF--------------------------------------SYYQEAK-  161
Ver.XP_012604632.1          GGILAIVVIITGCF--------------------------------------SYFQETK-  160
Ver.NP_653300.              SIVLSVVVIVTGCF--------------------------------------SYYQEAK-  161
Ver.XP_003892961.1          SIVLSVVVIITGCF--------------------------------------SYYQEAK-  161
Ver.XP_023069991.1          SIVLSVVVIITGCF--------------------------------------SYYQEAK-  161
Ver.UPI0001C650F7           GIVLAAVVIITGCF--------------------------------------SYYQEAK-  159
Ver.XP_013220247.1          GIVLSVVVIITGCF--------------------------------------SYYQEAK-  119
Art.ODM98254.1              GAVLAIVVIITGIF--------------------------------------SYYQESK-  135
Rinv.NP_0012967             GIVLSVVVIITGCF--------------------------------------SYYQESK-  159
Rinv.OQV17561.1(Group II)   GIVLAVVVIVTGCF--------------------------------------SYYQEAK-  199
Art.EFX71103.1              GIVLTAVVVVTGVF--------------------------------------SYLQERK-  132
Art.EFX71104.1              GIVLTTVVVVTGVF--------------------------------------SYLQERK-  132
Art.EFX71105.1              GIALTVVVVVTAMF--------------------------------------SYFQERK-  146
Art.XP_023221169.1          GLVLAIVVIVTSVF--------------------------------------SYYQESK-  52
Art.XP_022254094.1          GIVLAVVVIVTGCF--------------------------------------SYYQEAR-  190
Art.XP_023347331.1          GIVLTTVVVITGVF--------------------------------------SYYQESK-  135
Rinv.ALJ53300.1             GVALSVVVILTGVF--------------------------------------SYFQEAK-  153
Rinv.BAA32798.1             GVVLTAVVVITGCF--------------------------------------SYYQDAK-  152
Rinv.XP_018651572.1         GIVLLAVVVITGCF--------------------------------------SYYQESK-  147
Rinv.AAL09322.1             GIVLSVVVVVTGCF--------------------------------------SYYQESK-  137
Rinv.sp|Q6RWA9.1|           GIVLAAVVVITGCF--------------------------------------SYYQESK-  145
Rinv.CDS22215.1             GIVLAAVVVITGCF--------------------------------------SYYQESK-  156
Rinv.CDS36343.1             GIVLAAVVVITGCF--------------------------------------SYYQESK-  156
Rinv.AAX09623.1             GIVLTAVVVITGCF--------------------------------------SYYQEAK-  153
Rinv.ABO61333.1             GIVLTAVVIVTGIF--------------------------------------SYYQEAK-  158
Rinv.ABO61332.1             GIVLTAVVIVTGIF--------------------------------------SYYQEAK-  158
Rinv.EKC34610.1             GIVLTAVVLVTGIF--------------------------------------SYYQEAK-  197
Rinv.XP_022323941.1         GIVLTAVVLVTGIF--------------------------------------SYYQEAK-  163

Rinv.ELU12040.1             GIVLTAVVVVTGIF--------------------------------------AYYQEAK-  162
Rinv.XP_013405520.1         GIVLTAVVVVTGCF--------------------------------------SYYQEAK-  176
Rinv.NP_001116982.1         GIVLASVVIITGCF--------------------------------------SYYQEAK-  165
Nem.KHJ49479.1              GIVLAVVVIVTGIF--------------------------------------QYCQEAK-  24
Nem.CDW55413.1              GIVLAVVVIVTGIF--------------------------------------QYCQEAK-  170
Nem.KRY76685.1              GTVLAVVVIVTGCF--------------------------------------QYYQEAK-  197
Nem.KRZ73739.1              GTVLAVVVIVTGCF--------------------------------------QYYQEAK-  199
Nem.KRX35740.1              GTVLAVVVIVTGCF--------------------------------------QYYQEAK-  199
Nem.KRZ52541.1              GTVLAVVVIVTGCF--------------------------------------QYYQEAK-  201
Nem.UPI0006120BCD           GIVLATVVIITGCF--------------------------------------QYYQESK-  199
Nem.UPI0007A15AF4           GIVLAAVVIITGCF--------------------------------------QYYQESK-  106
Nem.UPI00020239EC           GIVLAAVVIITGCF--------------------------------------QYYQESK-  189
Nem.KHN72407.1              GIVLAAVVIITGCF--------------------------------------QYYQESK-  188
Nem.UPI0006036B9D           GIVLAGVVIITGCF--------------------------------------QYYQENK-  130
Nem.XP_024502753.1          GIVLMTVVVITGCF--------------------------------------QYYQENK-  113
Nem.UPI000605CA49           GIVLMTVVVITGCF--------------------------------------QYYQENK-  135
Nem.UPI000609E51C           GIVLMTVVVITGCF--------------------------------------QYYQESK-  89
Nem.UPI0007A24416           GIVLMTVVVITGCF--------------------------------------QYYQESK-  106
Nem.XP_003143231.1          GIVLMTVVVITGCF--------------------------------------QYYQESK-  130
Nem.UPI0007085A3B           GIVLMTVVVITGCF--------------------------------------QYYQESK-  142
Nem.OZC08885.1              GIVLMTVVVITGCF--------------------------------------QYYQESK-  130
Nem.UPI00060602BA           GIVLMTVVVITGCF--------------------------------------QYYQESK-  143
Nem.UPI000605F508           GIVLMTVVVITGCF--------------------------------------QYYQESK-  106
Nem.XP_001901816.1          GIVLMTVVVITGCF--------------------------------------QYYQESK-  130
Nem.UPI000818DDE2           GIVLMTVVVITGCF--------------------------------------QYYQESK-  148
Nem.UPI0007A17F26           GIVLMSVVIITGCF--------------------------------------QYYQESK-  130
Nem.UPI0007A19C37           GLVLMIVVIVTGCF--------------------------------------QYYQESK-  128
Nem.UPI000605DBB4           GLVLMIVVIVTGCF--------------------------------------QYYQESK-  128
Nem.KHN82508.1              GIVLMTVVVITGCF--------------------------------------QYYQESK-  130
Nem.ADY40856.1              GIVLMTVVVITGCF--------------------------------------QYYQESK-  130
Nem.ADY40930.1              GIVLMTVVVITGCF--------------------------------------QYYQESK-  139
Nem.UPI0006052FD1           GLVLMSVVIITGCF--------------------------------------QYYQESK-  130
Nem.UPI000BC5B284           GIVLMSVVIITGCF--------------------------------------QYFQERK-  89
Nem.UPI000BE6443D           GIVLMSVVIITGCF--------------------------------------QYYQESK-  143
Nem.UPI0001D4FB7A           GIVLMTVVIITGCF--------------------------------------QYYQESK-  131
Nem.UPI0001C851CE           GIVLMTVVVITGVF--------------------------------------QYYQESK-  131
Nem.P90735                  GIVLMTVVVITGVF--------------------------------------QYYQESK-  131
Nem.UPI000293EB87           GIVLMTVVVITGVF--------------------------------------QYYQESK-  131
Nem.UPI00060544A6           GIVLMTVVVITGCF--------------------------------------QYYQENK-  151
Nem.UPI0007A2DD20           GIVLMTVVVITGCF--------------------------------------QYYQESK-  132
Nem.UPI000342C523           GIVLMTVVVITGCF--------------------------------------QYYQESK-  196
Nem.U6PGW0                  GIVLMTVVVITGCF--------------------------------------QYYQESK-  132
Nem.UPI00060AD39A           GIVLMTVVVITGCF--------------------------------------QYYQESK-  132
```

```
Rinv.UPI0001782835(Group II)        GIVLASVVIITGCF--------------------------------------QYYQEAK-   122
Rinv.KXJ20422.1                     GVALAVVVILTGLF--------------------------------------SYYQEAK-   226
Rinv.XP_020601998.1                 GIVLSAVVLVTGIF--------------------------------------SYYQEAK-   191
Rinv.XP_020602001.1                 GIVLSAVVLVTGIF--------------------------------------SYYQEAK-   176
Art.XP_014232354.1                  GIVLTAVVIVTGIF--------------------------------------SYYQESK-   174
Art.XP_014205893.1                  GLVLAAVVIVTGIF--------------------------------------SYYQESK-   174
Art.XP_012269651.1                  GIVLAAVVIVTGIF--------------------------------------SYYQESK-   171
Art.XP_015585114.1                  GIVLAAVVIVTGIF--------------------------------------SYYQESK-   171
Art.KZC06498.1                      GIVLAAVVIVTGIF--------------------------------------SYYQESK-   137
Art.V9I6A9                          GIVLAAVVIVTGIF--------------------------------------SYYQESK-   144
Art.XP_012163873.1                  GIVLAAVVIVTGIF--------------------------------------SYYQESK-   171
Art.XP_012272094.1                  GIVLAAVVIVTGIF--------------------------------------SYYQESK-   171
Art.UPI0001FEE5BA                   GIVLAAVVIVTGIF--------------------------------------SYYQESK-   178
Art.EGI67709.1                      GIVLAAVVIVTGIF--------------------------------------SYYQESK-   137
Art.KYN38456.1                      GIVLAAVVIVTGIF--------------------------------------SYYQESK-   137
Art.UPI0005FA3D38                   GIVLAAVVIVTGIF--------------------------------------SYYQESK-   165
Art.XP_020294182.1                  GIVLAAVVIVTGIF--------------------------------------SYYQESK-   172
Art.EZA51212.1                      GIVLAAVVIVTGIF--------------------------------------SYYQESK-   171
Art.UPI0001E7C907                   GIVLAAVVIVTGIF--------------------------------------SYYQESK-   137
Art.XP_024883961.1                  GIVLAAVVIVTGIF--------------------------------------SYYQESK-   171
Art.UPI00091A11F9                   GIVLSAVVIVTGIF--------------------------------------SYYQESK-   131
Art.UPI00091201A3                   GIVLSAVVIVTGIF--------------------------------------SYYQESK-   169
Art.UPI000971E6B0                   GIVLSAVVIVTGIF--------------------------------------SYYQESK-   170
Art.T1E1Y4                          GIVLAAVVIVTGIF--------------------------------------SYYQESK-   130
Art.XP_021693479.1                  GIVLTAVVIVTGIF--------------------------------------SYYQESK-   170
Art.ETN62539.1                      GIVLAAVVIVTGIF--------------------------------------SYYQESK-   130

Art.UPI000CD7670C                   GIVLAAVVIVTGIF--------------------------------------SYYQESK-   165
Art.UPI0007D6117E                   GIVLAAVVIVTGIF--------------------------------------SYYQESK-   135
Art.UPI000153A0D9                   GIVLAAVVIVTGIF--------------------------------------SYYQESK-   130
Art.UPI0007D2379C                   GIVLAAVVIVTGIF--------------------------------------SYYQESK-   161
Art.UPI000957746B                   GIVLAAVVIVTGIF--------------------------------------SYYQESK-   154
Art.UPI000692F512                   GIVLAAVVIVTGIF--------------------------------------SYYQESK-   168
Art.T1PH35                          GIVLSAVVIVTGIF--------------------------------------SYYQESK-   132
Art.KNC28219.1                      GIVLSAVVIVTGIF--------------------------------------SYYQESK-   167
Art.UPI0005476AAF                   GIVLSAVVIVTGIF--------------------------------------SYYQESK-   168
Art.UPI000692F5BD                   GIVLSAVVIVTGIF--------------------------------------SYYQESK-   132
Art.XP_004536046.2                  GIVLSAVVIVTGIF--------------------------------------SYYQESK-   132
Art.A0A034W3G9                      GIVLSAVVIVTGIF--------------------------------------SYYQESK-   166
Art.UPI0006929A96                   GIVLSAVVIVTGIF--------------------------------------SYYQESK-   168
Art.XP_004536048.1                  GIVLSAVVIVTGIF--------------------------------------SYYQESK-   132
Art.UPI0005474384                   GIVLSAVVIVTGIF--------------------------------------SYYQESK-   132
Art.UPI0006ED933E                   GIVLSAVVIVTGIF--------------------------------------SYYQESK-   173
Art.UPI0006EDF4F9                   GIVLSAVVIVTGIF--------------------------------------SYYQESK-   169
Art.UPI0006BD3860                   GIVLSAVVIVTGIF--------------------------------------SYYQESK-   132
Art.XP_023176860.1                  GIVLSAVVIVTGIF--------------------------------------SYYQESK-   132
Art.UPI0006D327FB                   GIVLSAVVIVTGIF--------------------------------------SYYQESK-   171
Art.UPI00017C692D                   GIVLSAVVIVTGIF--------------------------------------SYYQESK-   172
Art.UPI0006EE4186                   GIVLSAVVIVTGIF--------------------------------------SYYQESK-   167
Art.XP_022210861.1                  GIVLSAVVIVTGIF--------------------------------------SYYQESK-   171
Art.UPI0007E80B92                   GIVLSAVVIVTGIF--------------------------------------SYYQESK-   171
Art.UPI0001781834                   GIVLSAVVIVTGIF--------------------------------------SYYQESK-   171
Art.NP_732572.                      GIVLSAVVIVTGIF--------------------------------------SYYQESK-   171
Art.UPI00017D261C                   GIVLSAVVIVTGIF--------------------------------------SYYQESK-   171
Art.XP_020799552.1                  GIVLSAVVIVTGIF--------------------------------------SYYQESK-   171
Art.XP_015032883.1                  GIVLSAVVIVTGIF--------------------------------------SYYQESK-   171
Art.AFU25675.1                      GIVLAAVVIVTGIF--------------------------------------SYYQESK-   139
Art.AFU25676.1                      GIVLAAVVIVTGIF--------------------------------------SYYQESK-   139
Art.BAS22117.1                      GIVLAAVVIVTGIF--------------------------------------SYYQESK-   139
Art.AFU25681.1                      GIVLAAVVIVTGIF--------------------------------------SYYQESK-   139
Art.XP_023954931.1                  GIVLAAVVIVTGIF--------------------------------------SYYQESK-   171
Art.XP_022114884.1                  GIVLAAVVIVTGIF--------------------------------------SYYQESK-   171
Art.AFU25678.1                      GIVLAAVVIVTGIF--------------------------------------SYYQESK-   139
Art.UPI00028A5EEF                   GIVLAAVVIVTGIF--------------------------------------SYYQESK-   166
Art.AFU25667.1                      GIVLAAVVIVTGIF--------------------------------------SYYQESK-   166
Art.AFU25673.1                      GIVLSAVVIVTGIF--------------------------------------SYYQESK-   139
Art.AFU25679.1                      GIVLAAVVIVTGIF--------------------------------------SYYQESK-   171
Art.UPI000B392785                   GIVLAAVVIVTGIF--------------------------------------SYYQESK-   139
Art.AFU25694.1                      GIVLAAVVIVTGIF--------------------------------------SYYQESK-   166
Art.XP_021196082.1                  GIVLAAVVIVTGIF--------------------------------------SYYQESK-   171
Art.XP_022817943.1                  GIVLAAVVIVTGIF--------------------------------------SYYQESK-   171
Art.AFU25670.1                      GLVLAGVVIITGIF--------------------------------------SYYQESK-   135
```

```
Art.XP_022903571.1             GIVLAAVVIVTGIF--------------------------------------SYYQESK-  166
Art.UPI00084EC8B0              GIVLAAVVIVTGIF--------------------------------------SYYQESK-  138
Art.U4UIT1                     GIVLAAVVIVTGIF--------------------------------------SYYQESK-  182
Art.XP_023016793.1             GIVLTAVVIVTGIF--------------------------------------SYYQESK-  172
Art.AFU25671.1                 GVVLAAVVIVTGIF--------------------------------------SYYQESK-  173
Art.AFU25692.1                 GIVLAAVVIVTGIF--------------------------------------SYYQESK-  174
Art.UPI000B551D17              GIVLAAVVIVTGIF--------------------------------------SYYQESK-  138
Art.AFU25686.1                 GVVLAAVVIVTGIF--------------------------------------SYYQESK-  141
Art.AFU25695.1                 GIVLSAVVIVTGIF--------------------------------------SYYQESK-  165
Art.XP_018562050.1             GIVLAAVVIITGIF--------------------------------------SYYQESK-  172
Art.UPI00028AEC41              GIVLAAVVIVTGIF--------------------------------------SYYQESK-  135
Art.AFU25691.1                 GVVLAAVVIVTGIF--------------------------------------SYYQESK-  138
Art.UPI000186D98E              GIVLAAVVIVTGIF--------------------------------------SYYQESK-  165
Art.XP_023711705.1             GIVLAAVVIVTGIF--------------------------------------SYYQESK-  191
Art.W5U4R1                     GIVLAAVVIITGIF--------------------------------------SYYQESK-  142
Art.AHH35009.1                 GVVLSAVVIVTGCF--------------------------------------SYYQEAK-  142
Art.AFU25683.1                 GLVLAGVVIITGIF--------------------------------------SYYQENK-  138
Art.XP_022184331.1             GIVLAAVVIVTGIF--------------------------------------SYYQENK-  169
Art.UPI00028BBB9D              GIVLATVVIITGIF--------------------------------------SYYQESK-  122
Art.UPI00028AA5CE              GIVLATVVIITGIF--------------------------------------SYYQESK-  124
Art.UPI0005464DB9              GIVLAAVVIVTGIF--------------------------------------SYYQESK-  143
Art.AFU25668.1                 GIVLAAVVIVTGIF--------------------------------------SYYQESK-  167
Art.XP_014271921.1             GIVLAAVVIVTGIF--------------------------------------SYYQESK-  140
Art.XP_014250371.1             GIVLAAVVIVTGIF--------------------------------------SYYQESK-  146

Art.UPI0007325ED7              GIVLAAVVIVTGIF--------------------------------------SYYQESK-  166
Art.XP_015929949.2             GVVLAAVVIVTGIF--------------------------------------SYYQEAK-  146
Art.XP_015930974.1             GIVLSVVVIVTGVF--------------------------------------SYYQEAK-  139
Art.XP_015907346.1             GIVLSVVVIVTGCF--------------------------------------SYYQEAR-  173
Art.XP_023221168.1             GIVLSTVVIVTGCFSYYQEARSSKIMESFKNMVPQLYLGIVLSTVVIVTGCFSYYQEAR-  214
Art.XP_015929951.2             GIVLATVVIVTGCF--------------------------------------SYYQEAR-  174
Art.XP_022253133.1             GVVLAAVVIITGCF--------------------------------------SYYQEAR-  170
Art.UPI0009F0198D              GIVLAVVVIVTGCF--------------------------------------SYYQEAR-  196
Art.XP_022668602.1             GIVLAVVVIVTGCF--------------------------------------SYYQEAR-  169
Art.UPI000B76A669              GAVLAIVVVVTGCF--------------------------------------SYYQEAR-  168
Art.UPI0007AA68F1              GAVLAIVVIVTGCF--------------------------------------SYYQEAR-  162
Art.AMK38059.1                 GAVLAIVVIVTGCF--------------------------------------SYYQEAR-  165
Art.UPI0007717391              GAVLAIVVIVTGCF--------------------------------------SYYQEAR-  163
Art.UPI00079F518A              GAVLAIVVIVTGCF--------------------------------------SYYQEAR-  164
Rinv.AOG19177.1                GIVLTAVVVVTGCF--------------------------------------SYYQEAK-  113
Rinv.AUG84438.1                GVVLTAVVVVTGCF--------------------------------------SYYQEAK-  156
Art.KYQ51534.1                 GIVLAAVVIVTGIF--------------------------------------SYYQESK-  137
Art.UPI000790B508              GIVLAAVVIVTGIF--------------------------------------SYYQESK-  172
Art.KPI92424.1                 GIVLAAVVIVTGIF--------------------------------------SYYQESK-  193
Art.AGZ13694.1(Group II)       GVVLSAVVIITGCF--------------------------------------SYYQESK-  173
Art.UPI000672CD46              GIVLTVVVVVTGVF--------------------------------------SYYQESK-  131
Art.XP_023337795.1             GVVLTAVVTVTGVF--------------------------------------SYYQESK-  135
Art.XP_023336146.1             GIVLSVVVIVTGIF--------------------------------------SYYQESK-  132
Art.XP_023323782.1             ------------------------------------------------------------  0
Art.AFU25682.1                 GLVLAGVVIITGVF--------------------------------------SYYQENK-  138
Art.AFU25689.1                 GLVLAGVVIITGVF--------------------------------------SYYQENK-  138
Art.EFX88073.1                 GIVLTTVVVVTGCF--------------------------------------SYYQESK-  134
Art.EFX88361.1                 GIVLTAVVVVTGVF--------------------------------------SYYQESK-  122
Art.XP_021944101.1             GAVLSVVVIVTGCF--------------------------------------SYYQESK-  131
Art.AIM43570.1                 GIVLTAVVIITGIF--------------------------------------SYYQESK-  143
Art.AFM54541.1                 GIVLTAVVIITGVF--------------------------------------SYYQESK-  139
Art.AEX07319.1                 GIVLTAVVIITGVF--------------------------------------SYYQESK-  141
Art.AIR93635.1                 GIVPTAVVIITGVF--------------------------------------SYYQESK-  168
Art.ADN83843.1                 GIVLTAVVIITGVF--------------------------------------SYYQESK-  168
Art.ABD59803.1                 GIVLTAVVIITGVF--------------------------------------SYYQESK-  168
Art.AAG47843.1                 GIVLTAVVIITGIF--------------------------------------SYYQESK-  169
Art.AGF90965.1                 GIVLTAVVIITGIF--------------------------------------SYYQESK-  169
Art.AGM39710.1                 GIVLTAVVIITGIF--------------------------------------SYYQESK-  167
Art.ABA02167.1                 GIVLTAVVIITGIF--------------------------------------SYYQESK-  140
Art.UPI0000085D74              GIVLTAVVIITGVF--------------------------------------SYYQENK-  168
Art.AJO70000.1                 GIVLTAVVIITGVF--------------------------------------SYYQENK-  168
Art.AJO70183.1                 GIVLTAVVIITGVF--------------------------------------SYYQENK-  168
Art.AKG50106.1                 GIVLTAVVIITGVF--------------------------------------SYYQENK-  168
Prt.CAI99406.1                 GVVLMLVVFITATF--------------------------------------SFLQEAK-  129
Prt.UPI0004A1BCA5              GVVLISVVIITATF--------------------------------------SYMQEAK-  135
Prt.UPI00014FFB3C              GVVLFTVVMITATF--------------------------------------SFLQEAK-  130
Prt.A0A090M1W3                 GVVLFTVVIITATF--------------------------------------SYLQEAK-  130
```

```
Prt.OUS42873.1                     GVVLFTVVIITATF--------------------------------------SYLQEAK-  130
Art.OWR44555.1(Group I)            GGIITATIIGTGLF--------------------------------------GFYQEAK-  157
Art.KPJ02649.1(Group I)            GCVITATILSTGIF--------------------------------------GFYQEAK-  156
Art.XP_004931505.2(Group I)        GCVLIAVDVICGLF--------------------------------------SFYQNYK-  91
Art.OWR53886.1(Group I)            GCVLIGVDVICGLF--------------------------------------SFFQNYKR  155
Art.KPJ07193.1(Group I)            GCVLIAVDIICGLF--------------------------------------SFFQNFK-  154
Art.KPI97351.1(Group I)            GCVLIAVDIICGLF--------------------------------------SFFQNFK-  154
Art.OXA57033.1                     GVSLLVVVLIAGSF--------------------------------------SYYQEFQ-  179
Art.ODM90865.1                     GIALVIVVFVSGMF--------------------------------------TFYQELK-  205
Art.ODM96221.1                     GCALIVVVHVSGAF--------------------------------------SFYQEYK-  106
Art.ODM99113.1                     FVSIVLVVHVSGTC--------------------------------------SFYQDFK-  155
Rinv.OQV20867.1                    GIVLIVVVFITGTF--------------------------------------SFMQQSK-  171
Art.K7IWP3(Group II)               GVIIVVICLISGVF--------------------------------------AYIQESK-  151
Art.EZA47803.1(Group II)           GIIIVVICITSGVF--------------------------------------AHIQETK-  142
Art.KYN03549.1(Group II)           GIIIVVICITSGVF--------------------------------------AYIQESK-  152
Art.KYQ48574.1(Group II)           GIIIVMICIISGVF--------------------------------------AYIQESK-  152
Art.KZC11054.1(Group II)           AFIITFICIISGVF--------------------------------------AYIQESK-  145
Art.KOX77187.1(Group II)           GIIIMIICLFSGFC--------------------------------------AYIQESK-  146
Art.OAD46911.1(Group II)           GVIITAICIFSGVF--------------------------------------AYIQESK-  147
Art.KOC67548.1(Group II)           GIIITLICLISGFS--------------------------------------AYIQESK-  149
Art.OXA54637.1(Group II)           GITVAAVVILLGMF--------------------------------------TFYQEYS-  201
Art.ODN05419.1(Group II)           GVVLIVLVIVLGLF--------------------------------------TFYQEYS-  183

Prt.EGD77429.1                     GIVLAAVVFITGCF--------------------------------------SYVQERR-  340
Nem.CEF64940.1(Group II)           GIVIAVVTLLTSIY--------------------------------------HYYQSRK-  138
Prt.EGD75712.1                     GVVLSGVVVITGVF--------------------------------------SYFQESK-  219
Rinv.OQV25202.1                    GIALLLVVTVSSTF--------------------------------------AYWQERK-  222
Art.XP_023321169.1(Group II)       GCALVVVVAISGIF--------------------------------------TYMQERQ-  121
Art.D6WB95(Group II)               GCVLVTVVVVTGCF--------------------------------------MYFQEHK-  146
Art.XP_018565491.1(Group II)       GCVLVVVVVVTGCF--------------------------------------MYFQEHK-  145
Art.AFU25672.1(Group II)           GCVLVVVVVVTGCF--------------------------------------MYFQEHK-  145
Art.KYN18319.1(Group II)           GLVLIVLIMITGVF--------------------------------------SYYQDSK-  137
Art.EFN88446.1(Group II)           GLVLIVLILVTGTF--------------------------------------SYYQDSK-  136
Art.A0A087ZR23(Group II)           SIVLVILILVTAMF--------------------------------------SHYQESK-  142
Art.XP_012279085.1(Group II)       GMVLILLILVTGMF--------------------------------------SYYQNSK-  140
Prt.BAA82752.2                     GIVLFAVVFVTGCF--------------------------------------SFFQNSK-  131
Prt.OQR99879.1                     AVALIAVILVSGLF--------------------------------------SYFQNKK-  175
Prt.OQR92998.1                     ASALFTVILVTGLF--------------------------------------SYFQNSK-  174
Prt.XP_008607481.1                 ATALFVTILVTGFF--------------------------------------SYFQNRK-  176
Prt.XP_008604114.1                 GIVLFCVVIITGLF--------------------------------------SYSQNRK-  155
Prt.OQS04799.1                     GVVLFGVVVITGLF--------------------------------------SYSQNRK-  151
Prt.OQR94535.1                     GIVLFCVVVITGLF--------------------------------------SYSQNRK-  151
Prt.CCA16430.1                     GIVLFLVVIITGTF--------------------------------------SYFQNRK-  156
Prt.POM62354.1                     GIVLFLVVIITGTF--------------------------------------SYFQNAK-  158
Prt.RAW38513.1                     GIVLFLVVVITGTF--------------------------------------SYFQNAK-  158
Prt.XP_024585310.1                 GIVLFLVVIITGTF--------------------------------------SYFQNAK-  158
Prt.XP_647420.2                    GIILWIVVFLTCTF--------------------------------------SYIQNSK-  249
Prt.XP_004352438.1                 GIILWAVVLFTTIF--------------------------------------TFLQESK-  300
Prt.tr|D3BBA2|                     GIILWAVVIITCTF--------------------------------------TFFQESK-  225
Prt.UPI00000795B1                  ACVLYAIVIFTCLL--------------------------------------TFSQERQ-  120
Prt.F1A2S2                         ACVLYAIVIFTCLL--------------------------------------TFSQERQ-  120
Prt.PXF41383.1                     GIVLYCVVVITAIF--------------------------------------TFLQEFK-  165
Prt.CAI99405.1                     GVVLYVVVVITALF--------------------------------------TFMQEFK-  172
Fun.SPPG_08470.2                   GCILISVAFINAGI--------------------------------------EFYELQK-  217
Fun.UniRef100_A0A4P9WG54           GAILIGVAFINAGI--------------------------------------EFYELQK-  132
Fun.AMAG_01211.1                   GPILLGVAHLNAFI--------------------------------------EWYQFHK-  129
Fun.AMAG_02439.1                   GSILLGVAFLNSFI--------------------------------------EFYQIQK-  131
Fun.UniRef100_A0A1Y2HI79           GPILLGVAFLNAFI--------------------------------------EFYMGAK-  127
Fun.UPI000006A49F                  GSILVAVAFLNAFI--------------------------------------EYYQVAK-  129
Fun.AAF20202.1                     GSILVAVAFLNAFI--------------------------------------EYYQVAK-  128
Fun.estExt_Genewise1.C_180045      GAILIAVAFLNAFI--------------------------------------EWFQGQK-  213
Fun.PGTG_03133.2                   GAILIIVAFLNAFI--------------------------------------EWYQGQK-  203
Fun.CC1G_09151.2                   GAILLFVAFLNAGI--------------------------------------DFYQIQK-  163
Fun.UniRef100_A0A067M739           GGILIAVAFLNAYI--------------------------------------EFYQMQK-  184
Fun.UPI0001643CDB                  GGILILVAFINASI--------------------------------------DFYQIQK-  155
Fun.UniRef100_A0A5E3X872           GGILIAVAFLNAFI--------------------------------------EFYQLQK-  209
Fun.fgenesh2_pm.C_sc               GGILIGAAFLNAFI--------------------------------------EFYQLQK-  154
Fun.UniRef100_A0A4Y9ZEZ1           GGILIAVAFLNAFI--------------------------------------EFYQLQK-  207
Fun.UniRef100_A0A067Q0Q4           GGILIAVAFLNAFI--------------------------------------EFYQLQK-  198
Fun.e_gwh2.1.49.1                  GGILIAVAFLNAFI--------------------------------------EFYQLQK-  155
Fun.UniRef100_A0A4S4MS29           GGILIAVAFLNAFI--------------------------------------EFYQLQK-  204
Fun.UniRef100_A0A1M2VF35           GGILIGVAFLNALI--------------------------------------EFYQLQK-  51
```

```
Fun.UniRef100_J4GT94            GGILIAVAFLNAFI--------------------------------------EFFQLQK-  203
Fun.UniRef100_A0A4Y9Y8I4        GGILIAVAFINAFI--------------------------------------EFFQLQK-  204
Fun.UniRef100_S8E4T6            GGILIAVAFINAFI--------------------------------------EFFQLQK-  153
Fun.UniRef100_A0A1Y1VYW4        GAILI-----------------------------------------------AWYQQRK-  150
Fun.UniRef100_A0A2G5BEZ7        GAILIGVAFMNAGV--------------------------------------EWYQQRK-  192
Fun.UniRef100_A0A507F1Y9        GAIMIGIGLMNAFI--------------------------------------EFYQQQK-  163
Fun.UniRef100_A0A1Y2BXZ1        -PILLGVALMNAAI--------------------------------------EFYQEQK-  89
Fun.UniRef100_A0A1Y2BYC1        GAILIGVGFMNAFI--------------------------------------EFYQQQK-  158
Fun.UniRef100_A0A4P9WWS6        GAILLVVALMNAFI--------------------------------------DFYQQQK-  163
Fun.UniRef100_A0A507BSP0        GAILIAVAFMNAGI--------------------------------------EFYQIQK-  173
Fun.BDEG_03368.1                GAILIIVAFMNASI--------------------------------------EFYQVQK-  142
Fun.BDEG_05936.1                GAILIIVAFMNASI--------------------------------------EFYQVQK-  146
Fun.UniRef100_A0A507E7U2        GGILILVSFLNATI--------------------------------------EFVQGQK-  191
Fun.SPPG_07476.2                GAILLAVAFLNAFI--------------------------------------EFFQGQK-  111
Fun.UniRef100_A0A507EKQ2        GAILIAVAFLNAFI--------------------------------------EFFQGQK-  233
Fun.UniRef100_A0A1X2ID33        GAIYIGVALLNAFI--------------------------------------EFYQEYK-  160
Fun.fgenesh1_pm.12_#_37         GAILIGVALLNAFI--------------------------------------EFYQEQK-  99
Fun.RO3G_04175.3                GAILIGVALLNAFI--------------------------------------EFYQEQK-  181
Fun.UniRef100_A0A0B7MNY4        GAILIGVALLNAFI--------------------------------------EFYQEQK-  153
Fun.UniRef100_A0A0C9MZH6        GAILIGVALLNAFI--------------------------------------EFYQEQK-  183

Fun.fgenesh1_pm.01_#_34         GAILIGVALLNAFI--------------------------------------EFYQEQK-  92
Fun.UniRef100_S2JVU7            GAILIGVALLNAFI--------------------------------------EFYQEQK-  183
Fun.UniRef100_A0A139AWN6        GGILIAVAFLNSFI--------------------------------------EYYQEAK-  148
Fun.UniRef100_A0A2Z6QEU7        GAILFGVAFLNAFL--------------------------------------QFIQLQK-  161
Fun.UniRef100_A0A2N0PA04        GAIFLGVAFLNAFI--------------------------------------EFYQLQK-  162
Fun.UniRef100_A0A397VHQ3        GAVLIGVAFLNAFI--------------------------------------EFYQLQK-  173
Fun.SPPG_01615.2                GAILLGVALLNAFI--------------------------------------EFYQLQK-  131
Fun.UniRef100_A0A194X4V3        GAILIAVAFINAFI--------------------------------------EFYQQQK-  165
Fun.UniRef100_A0A261Y8Y1        GAILIAVAFINAFI--------------------------------------EFYQQQK-  202
Fun.UniRef100_A0A0C3GKR9        GAILIAVAFINAFI--------------------------------------EFYQQQK-  195
Fun.UniRef100_A0A2J6SAB3        GAILILVAFINAFI--------------------------------------EFYQQQK-  217
Fun.UniRef100_A0A2J6SIQ3        GAILIAVAFINAFI--------------------------------------EFYQQQK-  200
Fun.fgenesh2_pm.7_#_193         GAILIAVANINAFI--------------------------------------EFYQQRK-  190
Fun.UniRef100_A0A090D7D5        GAILIIVANINAFI--------------------------------------EFYQQSK-  173
Fun.UniRef100_A0A447CBT6        GAILIIVANINAFI--------------------------------------EFYQQSK-  173
Fun.UniRef100_A0A507CSE4        GAILIAVAFLNAFI--------------------------------------EFYQLQK-  201
Fun.UniRef100_A0A4P9Y2Y3        GGILIGVAFLNAFI--------------------------------------EFYQSQK-  148
Fun.UniRef100_A0A1Y2G5J7        GAILIIVAFINAAI--------------------------------------EFYQAQK-  177
Fun.UniRef100_A0A4P9ZM46        GAILIAVAFLNAFI--------------------------------------EFYQLQK-  172
Fun.UniRef100_A0A4P9Z1Y8        GAILFVVAFANAFI--------------------------------------EFYQHQK-  178
Fun.UniRef100_A0A137NQA6        GGILIGVAVVNALI--------------------------------------EFVQLLK-  127
Fun.UniRef100_A0A1Y1YKS1        GAILIGVALVNAFI--------------------------------------EFYQLQK-  192
Fun.UniRef100_A0A1Y1YU47        GAILIVVALLNAFI--------------------------------------EFFQMLK-  188
Nem.KHN74191.1(Group I)         TIILIAIVFIMSFL--------------------------------------SFYQEKK-  140
Nem.NP_001122529.1(Group I)     AIILIAVVVFMGML--------------------------------------SYWQQAK-  181
Nem.tr|O16436|(Group I)         GIFIIVIIFVMCIV--------------------------------------SFFEEKK-  152
Nem.tr|O16331|(Group I)         GIFIVAIVFIMCVV--------------------------------------SFFEEKK-  152
Nem.KHN88767.1(Group I)         AIVLFLIVIVMCTV--------------------------------------TFFEEKK-  154
Nem.KHN88766.1(Group I)         AVVLILIVVVMCVV--------------------------------------TFFEEKK-  168
Prt.XP_024578788.1              AILLFAVVLGTCTA--------------------------------------TFLQERS-  156
Prt.GAX12878.1                  GLALLAVVFLTGYG--------------------------------------QYHEERK-  151
Prt.GAX20661.1                  GLALLAVVFLTGFG--------------------------------------QYHEERK-  150
Prt.UPI00015F4774               GVVLLVVVFISSTF--------------------------------------AYFQEAK-  134
Prt.UPI000D26B24D               GVVLILVVVLSATF--------------------------------------GYYQEAK-  171
Prt.XP_001427178.1              GIVIVVVNTLTGVI--------------------------------------TFFQNAK-  178
Prt.XP_001346890.1              AIILIAIILLTGSI--------------------------------------TYNQSAK-  210
Prt.UniRef100_Q23EX6            GIIIVIVIFMTGGI--------------------------------------TFMQNAK-  214
Prt.UniRef100_Q22XZ1            TIILAIVIFISAAI--------------------------------------NFQQHSS-  162
Prt.UniRef100_Q22LQ9            AIVILVVIFITTAI--------------------------------------TFQQNSK-  211
Prt.UniRef100_Q245Y8            AVVLIIVILITTAI--------------------------------------TFQQNAK-  221
Prt.UniRef100_Q23ZA6            AVVLIIVILITTAI--------------------------------------TFQQNAK-  208
Prt.UniRef100_I7M7N1            AIVLLIVIFVTAFI--------------------------------------TFQQNAK-  221
Prt.UniRef100_I7ME52            AIVLLIVIFITAFI--------------------------------------TYQQNAK-  230
Prt.UniRef100_I7MH18            GIVIIIVISITSGI--------------------------------------TFLQNAK-  214
Prt.UniRef100_I7MD85            GIVIIIVISITAVI--------------------------------------TFQQNAK-  209
Prt.UniRef100_Q23D88            GIVLILVIFLTGYI--------------------------------------TFQQTAK-  222
Prt.UniRef100_I7MHE1            GFVLIAVIFVTAQI--------------------------------------TYQQNKK-  216
Prt.UniRef100_I7M7R6            AIVLIIVIFITAII--------------------------------------TFMQNRK-  418
Prt.UniRef100_Q22P96            GIILIAVVLITAII--------------------------------------TYQQNKK-  230
Prt.UniRef100_Q22PA2            GIILIFVVLITAFI--------------------------------------TFQQNKK-  208
Prt.PXF41326.1                  GLFLYTVVFVTSLF--------------------------------------SFIQQHK-  147
```

```
Prk.YP_324582.1          GWAIWAVIWINAVF--------------------------------------SFWQEFQ-  103
Prk.NP_440621.1          GWAIWAVIWINGLF--------------------------------------SFSQEFR-  113
Prk.UPI0008639F5F        GFAIISVIVINAVF--------------------------------------SFWQEYE-  99
Prk.NP_276630.1          AVAIVMVIIINALF--------------------------------------SFWQEYE-  104
Prk.YP_502111.1          AAAVWMVNIINGLF--------------------------------------SFWQEFR-  106
Prk.tr|A0A347ZR85|       TIAIWLVNVINGAF--------------------------------------SFWQEFR-  117
Prk.UPI00032DDB86        GVAIWLVNIINGLF--------------------------------------SFWQQQR-  105
Prk.tr|A0A4R8A713|       GIAIWLVNVINGLF--------------------------------------SFWQEFQ-  104
Prk.YP_001963725.1       GLAIFIVVLINGIF--------------------------------------SFFQESK-  247
Prk.YP_391334.1          AYAVFAAVFLNATF--------------------------------------TFWQKFK-  105
Prk.tr|W5W8S0|           AVAILVVILLNALL--------------------------------------AFAQERQ-  89
Prk.tr|A0A1M7YB86|       GWALFGVALLNAMF--------------------------------------SFVQEYR-  111
Prk.YP_357688.1          GWALAGVALLNALF--------------------------------------SFIQEYR-  110
Prk.tr|A0A1G6WYT0|       GQALAGVALLNALF--------------------------------------SFGQEYR-  110
Prk.tr|A0A1M6NFZ1|       GWALAGVALLNALF--------------------------------------SFVQEYR-  110
Prk.tr|A0A1H7UXF5|       GFAIIGVIMINAVF--------------------------------------TFIQEYR-  106
Prk.tr|B5YJF3|           GFAIIGVIFINAIF--------------------------------------AFVQEYR-  106
Prk.tr|A0A317MXL5|       GVAIVGVIVVNGLF--------------------------------------SFWQEYR-  108

Prk.tr|A0A1H1W1D1|       GWAILGVIAINGSF--------------------------------------SFWQAYR-  106
Prk.UPI0002387025        GFAIVGVIIINGLF--------------------------------------AFWQSYR-  111
Prk.tr|A0A497XH49|       GLAIVGVIVVNGCF--------------------------------------SFWQAWR-  111
Prk.UPI0000E10E8B        GEALVFVVVLNAQV--------------------------------------SFYQNRK-  113
Prk.UPI000C2216DF        AWALLAVTLLNAIF--------------------------------------TFFQEQK-  127
Prk.UPI000C21D419        AWALLGVTLLNAIF--------------------------------------TFFQEQK-  127
Prk.UPI000B509329        GAALLVVMLLNGSF--------------------------------------SWYQQRK-  105
Prk.tr|K0C7I7|           GCALYGVVLLNAIF--------------------------------------TFIQAYQ-  107
Prk.UPI00057321B9        GIALLGVVILNGIF--------------------------------------SFFQEYR-  107
Prk.UPI000615B8D6        GLALGGVVVLNGTF--------------------------------------TFIQEYQ-  122
Prk.AKB42896.1           GIALGVVVILNGTF--------------------------------------TFVQEYQ-  118
Prk.UPI0006157E2A        GIALVGVVILNGTF--------------------------------------TFVQEYQ-  122
Prk.UPI00061563E3        GIALLGVVIINGTF--------------------------------------TFVQEYQ-  111
Prk.AKB83839.1           GIALLGVVIINGTF--------------------------------------TFVQEYQ-  111
Prk.UPI00003C6559        GIALAGVVIINGTF--------------------------------------TFIQEYQ-  122
Prk.UPI0006157AA6        GIALAGVVVINGTF--------------------------------------TFIQEYQ-  122
Prk.UPI0006157148        GIALVGVVFLNGTF--------------------------------------TFIQEYQ-  122
Prk.NP_633093.1          GIALVGVVFLNGTF--------------------------------------TFIQEYQ-  122
Prk.UPI000615BB49        GVALVGVVVLNGTF--------------------------------------TFVQEYQ-  122
Prk.UPI0000068661        GIALIGVVFLNGTF--------------------------------------TFVQEYQ-  122
Prk.UPI0006154E5D        GVALVGVVFLNGTF--------------------------------------TFVQEYQ-  122
Prk.YP_565169.1          AIALFGVVILNATF--------------------------------------TFIQEYQ-  85
Prk.UPI0008DEB8E8        AIALLGVVILNGTF--------------------------------------TFIQEYQ-  118
Prk.UPI000891AE6A        AIALLGVVILNGTF--------------------------------------TFIQEYQ-  118
Prk.UPI00079C7F01        AIALLGVVILNSTF--------------------------------------TFIQEYQ-  121
Prk.UPI00028B8ECD        AIALLGVVILNATF--------------------------------------TFIQEYQ-  118
```
