# Supplementary material for: Bioinformatics approaches for classification and investigation of the evolution of the Na/K-ATPase alpha-subunit
Source: BMC Ecol Evol. 2022 Oct 26;22:122. doi: 10.1186/s12862-022-02071-0 (PMC9609216; doi:10.1186/s12862-022-02071-0)
Supplement: Supplementary file 1 — Additional file 1. Supplementary figures and tables. [file 12862_2022_2071_MOESM1_ESM.zip › Additional file 1 Fig. S7.pdf]

```
Ver.NP_0010804              GGDRVPADLRIISSH-GCKV--DNSSLTGE-SEPQTRSPDCT-----H-DNP-------- 238
Ver.F7E0B8                  GGDRVPADLRIISSH-GCKV--DNSSLTGE-SEPQTRSPDCT-----H-ENP-------- 238
Ver.UPI000C736357           GGDRVPADLRIISAH-GCKV--DNSSLTGE-SEPQTRSPDCT-----H-DNP-------- 222
Ver.UPI0003CD047A           GGDRVPADLRIISAH-GCKV--DNSSLTGE-SEPQTRSPDCT-----H-DNP-------- 241
Ver.UPI000C73EFA7           GGDRVPADLRIISAH-GCKV--DNSSLTGE-SEPQTRSPDCT-----H-DNP-------- 262
Ver.UPI00049A9E19           GGDRVPADLRIISSH-GCKV--DNSSLTGE-SEPQTRSPDCT-----H-DNP-------- 245
Ver.XP_025028557.1          GGDRVPADLRIISAH-GCKV--DNSSLTGE-SEPQTRSPDCT-----H-DNP-------- 240
Ver.XP_020663591.1          GGDRVPADLRIISAH-GCKV--DNSSLTGE-SEPQTRSPDCT-----H-DNP-------- 301
Ver.UPI0000124FC2           GGDRVPADLRIISAH-GCKV--DNSSLTGE-SEPQTRSPDCT-----H-DNP-------- 223
Ver.UPI000BAD5294           GGDRVPADLRIISAH-GCKV--DNSSLTGE-SEPQTRSPDCT-----H-DNP-------- 222
Ver.XP_020948935.1          GGDRVPADLRIISAH-GCKV--DNSSLTGE-SEPQTRSPDCT-----H-DNP-------- 240
Ver.XP_012613923.1          GGDRVPADLRIISAH-GCKV--DNSSLTGE-SEPQTRSPDCT-----H-DNP-------- 226
Ver.UPI000C2D7C35           GGDRVPADLRIISAH-GCKV--DNSSLTGE-SEPQTRSPDCT-----H-DNP-------- 243
Ver.XP_006903931.1          GGDRVPADLRIISAH-GCKV--DNSSLTGE-SEPQTRSPDCT-----H-DNP-------- 196
Ver.XP_023380497.1          GGDRVPADLRIISAH-GCKV--DNSSLTGE-SEPQTRSPDCT-----H-DNP-------- 297
Ver.UPI0002B3612F           GGDRVPADLRIISAH-GCKV--DNSSLTGE-SEPQTRSPDCT-----H-DNP-------- 236
Ver.XP_020726792.1          GGDRVPADLRIISAH-GCKV--DNSSLTGE-SEPQTRSPDCT-----H-DNP-------- 191
Ver.ELK32312.1              GGDRVPADLRIISAH-GCKV--DNSSLTGE-SEPQTRSPDCT-----H-DNP-------- 265
Ver.UPI000226419C           GGDRVPADLRIISAH-GCKV--DNSSLTGE-SEPQTRSPDCT-----H-DNP-------- 241
Ver.UPI000C7286EF           GGDRVPADLRIISAH-GCKV--DNSSLTGE-SEPQTRSPDCT-----H-DNP-------- 226
Ver.UPI0007A6EC9C           GGDRVPADLRIISAH-GCKV--DNSSLTGE-SEPQTRSPDCT-----H-DNP-------- 226
Ver.UPI000C2DAA95           GGDRVPADLRIISAH-GCKV--DNSSLTGE-SEPQTRSPDCT-----H-DNP-------- 232
Ver.UPI000C740E55           GGDRVPADLRIISAH-GCKV--DNSSLTGE-SEPQTRSPDCT-----H-DNP-------- 237
Ver.UPI000CB4CAB6           GGDRVPADLRIISAH-GCKV--DNSSLTGE-SEPQTRSPDCT-----H-DNP-------- 237
Ver.PNJ19200.1              GGDRVPADLRIISAH-GCKV--DNSSLTGE-SEPQTRSPDCT-----H-DNP-------- 226
Ver.XP_021021704.1          GGDRVPADLRIISAH-GCKV--DNSSLTGE-SEPQTRSPDCT-----H-DNP-------- 239
Ver.UPI00035B05DE           GGDRVPADLRIISAH-GCKV--DNSSLTGE-SEPQTRSPDCT-----H-DNP-------- 261
Ver.XP_023600635.1          GGDRVPADLRIISAH-GCKV--DNSSLTGE-SEPQTRSPDCT-----H-DNP-------- 205
Ver.UPI000C732D5F           GGDRVPADLRIISAH-GCKV--DNSSLTGE-SEPQTRSPDCT-----H-DNP-------- 237
Ver.PNI95395.1              GGDRVPADLRIISAH-GCKV--DNSSLTGE-SEPQTRSPDCT-----H-DNP-------- 226
Ver.NP_036638.              GGDRVPADLRIISAH-GCKV--DNSSLTGE-SEPQTRSPDCT-----H-DNP-------- 226
Ver.XP_022441242.1          GGDRVPADLRIISAH-GCKV--DNSSLTGE-SEPQTRSPDCT-----H-DNP-------- 240
Ver.UPI000651771D           GGDRVPADLRIISAH-GCKV--DNSSLTGE-SEPQTRSPDCT-----H-DNP-------- 226
Ver.UPI000C7355ED           GGDRVPADLRIISAH-GCKV--DNSSLTGE-SEPQTRSPDCT-----H-DNP-------- 251
Ver.XP_021590883.1          GGDRVPADLRIISAH-GCKV--DNSSLTGE-SEPQTRSPDCT-----H-DNP-------- 263
Ver.XP_021106581.1          GGDRVPADLRIISAH-GCKV--DNSSLTGE-SEPQTRSPDCT-----H-DNP-------- 239
Ver.XP_024433413.1          GGDRVPADLRIISAH-GCKV--DNSSLTGE-SEPQTRSPDCT-----H-DNP-------- 226
Ver.UPI000C2EDFE7           GGDRVPADLRIISAH-GCKV--DNSSLTGE-SEPQTRSPDCT-----H-DNP-------- 237
Ver.UPI000C2E3154           GGDRVPADLRIISAH-GCKV--DNSSLTGE-SEPQTRSPDCT-----H-DNP-------- 239
Ver.XP_023507169.1          GGDRVPADLRIISAH-GCKV--DNSSLTGE-SEPQTRSPDCT-----H-DNP-------- 227
Ver.KFO33633.1              GGDRVPADLRIISAH-GCKV--DNSSLTGE-SEPQTRSPDCT-----H-DNP-------- 196

Ver.UPI000C71DF25           GGDRVPADLRIISAH-GCKV--DNSSLTGE-SEPQTRSPDCT-----H-DNP-------- 237
Ver.UPI0001914BDE           GGDRVPADLRIISAH-GCKV--DNSSLTGE-SEPQTRSPDCT-----H-DNP-------- 239
Ver.XP_003799510.1          GGDRVPADLRIISAH-GCKV--DNSSLTGE-SEPQTRSPDCT-----H-DNP-------- 239
Ver.XP_008065591.1          GGDRVPADLRIISAH-GCKV--DNSSLTGE-SEPQTRSPDCT-----H-DNP-------- 196
Ver.sp|P13637.3|            GGDRVPADLRIISAH-GCKV--DNSSLTGE-SEPQTRSPDCT-----H-DNP-------- 226
Ver.KPP65694.1              GGDRIPADLRIVSSH-GCKV--DNSSLTGE-SEPQTRSPDCT-----H-DNP-------- 200
Ver.XP_023665796.1          GGDRIPADLRITSSH-GCKV--DNSSLTGE-SEPQTRSPDCT-----H-DNP-------- 235
Ver.XP_015461719.2          GGDRIPADLRIISSH-GCKV--DNSSLTGE-SEPQTRSPDCT-----H-DNP-------- 237
Ver.BAB60722.1              GGDRIPADLRIVSSH-GCKV--DNSSLTGE-SEPQTRSPDCT-----H-DNP-------- 235
Ver.UPI000054C9F5           GGDRIPADLRIISAH-GCKV--DNSSLTGE-SEPQTRSPDCT-----H-DNP-------- 236
Ver.UPI0004E4D157           GGDRIPADLRIISSH-GCKV--DNSSLTGE-SEPQTRSPECT-----H-DNP-------- 235
Ver.UPI000293B6B0           GGDRIPADLRIISSH-GCKV--DNSSLTGE-SEPQTRSPECT-----H-DNP-------- 235
Ver.UPI0000E3AF2C           GGDRIPADLRIVSSH-GCKV--DNSSLTGE-SEPQTRSPDGT-----H-DNP-------- 235
Ver.XP_024920682.1          GGDRIPADLRIISSH-GCKV--DNSSLTGE-SEPQTRSPDCT-----H-DNP-------- 236
Ver.UPI0003B0448B           GGDRIPADLRIISSH-GCKV--DNSSLTGE-SEPQTRSPDCT-----H-DNP-------- 235
Ver.UPI00016E235F           GGDRIPADLRIISSH-GCKV--DNSSLTGE-SEPQTRSPDCT-----H-DNP-------- 238
Ver.UPI00032B6FE9           GGDRIPADLRIISSH-GCKV--DNSSLTGE-SEPQTRSPDCT-----H-DNP-------- 236
Ver.XP_004074116.1          GGDRIPADLRVISSH-GCKV--DNSSLTGE-SEPQTRSPDCT-----H-DNP-------- 235
Ver.KKF19362.1              GGDRIPADLRIISSH-GCKV--DNSSLTGE-SEPQTRSPDCT-----H-DNP-------- 249
Ver.XP_012711044.2          GGDRIPADLRIISSH-GCKV--DNSSLTGE-SEPQTRSPDCT-----H-DNP-------- 235
Ver.UPI00025F91A4           GGDRIPADLRIISSH-GCKV--DNSSLTGE-SEPQTRSPDCT-----H-DNP-------- 235
Ver.XP_020793662.1          GGDRIPADLRIISSH-GCKV--DNSSLTGE-SEPQTRSPDCT-----H-DNP-------- 235
Ver.XP_024153267.1          GGDRIPADLRIISSH-GCKV--DNSSLTGE-SEPQTRSPDCT-----H-DNP-------- 235
Ver.XP_023117914.1          GGDRIPADLRIISSH-GCKV--DNSSLTGE-SEPQTRSPDCT-----H-DNP-------- 235
Ver.XP_022053465.1          GGDRIPADLRIISSH-GCKV--DNSSLTGE-SEPQTRSPDCT-----H-DNP-------- 235
Ver.NP_571759.2             GGDRIPADLRIISAH-GCKV--DNSSLTGE-SEPQTRSPDCT-----H-DNP-------- 236
Ver.W5UML4                  GGDRIPADLRVISAH-GCKV--DNSSLTGE-SEPQTRSPDCT-----H-DNP-------- 237
Ver.W5L4G0                  GGDRIPADLRIISAH-GCKV--DNSSLTGE-SEPQTRSPDCT-----H-DNP-------- 235
Ver.UPI0005D90DB9           GGDRIPADLRIISAH-GCKV--DNSSLTGE-SEPQTRSPDCT-----H-DNP-------- 235
Ver.XP_021427657.1          GGDRIPADLRIVSAH-GCKV--DNSSLTGE-SEPQSRSPDCT-----H-DNP-------- 241
```

```
Ver.XP_024297426.1          GGDRIPADLRIVSAH-GCKV--DNSSLTGE-SEPQSRSPDCT-----H-DNP-------- 246
Ver.UPI00001DFF47           GGDRIPADLRVVSAH-GCKV--DNSSLTGE-SEPQSRSPDCT-----H-DNP-------- 224
Ver.UPI0006B7181A           GGDRIPADLRVVSAH-GCKV--DNSSLTGE-SEPQSRSPDCT-----H-DNP-------- 237
Ver.UPI00079DB5F5           GGDRIPADLRIISAH-GCKV--DNSSLTGE-SEPQTRTPDFS-----N-ENP-------- 222
Ver.UPI000050D2B6           GGDRIPADIRVVSAH-GCKV--DNSSLTGE-SEPQSRSPDNT-----H-DNP-------- 221
Ver.UPI0007F716B9           GGDRIPADIRVVSAH-GCKV--DNSSLTGE-SEPQSRSPDCT-----H-DNP-------- 222
Ver.UPI0000E9CD46           GGDRIPADIRIVSAH-GCKV--DNSSLTGE-SEPQNRSPDCT-----H-DNP-------- 234
Ver.UPI0007F7EA5B           GGDRIPADLRVISSH-GCKV--DNSSLTGE-SEPQTRSPDCT-----H-DNP-------- 235
Ver.UPI00079E2EF3           GGDRIPADIRVVSAH-GCKV--DNSSLTGE-SEPQSRSPDNT-----H-DNP-------- 222
Ver.UPI0004448FEC           GGDRIPADIRVISAH-GCKV--DNSSLTGE-SEPQSRSPDCT-----H-DNP-------- 222
Ver.XP_023187147.1          GGDRIPADIRVISAH-GCKV--DNSSLTGE-SEPQSRSPDCT-----H-DNP-------- 235
Ver.UPI0006B30A18           GGDRIPADIRVVSAH-GCKV--DNSSLTGE-SEPQSRSPDCT-----H-DNP-------- 237
Ver.XP_017270842.1          GGDRIPADIRVVSAH-GCKV--DNSSLTGE-SEPQSRSPDCT-----H-DNP-------- 237
Ver.T2B507                  GGDRIPADIRVVSAH-GCKV--DNSSLTGE-SEPQSRSPDCT-----H-DNP-------- 223
Ver.XP_020466584.1          GGDRIPADIRVVSAH-GCKV--DNSSLTGE-SEPQSRSPDCT-----H-DNP-------- 236
Ver.UPI0000124FC4           GGDRIPADIRVTSAH-GCKV--DNSSLTGE-SEPQSRSPDCT-----H-DNP-------- 223
Ver.XP_004550929.1          GGDRIPADIRVTSAH-GCKV--DNSSLTGE-SEPQSRSPDCT-----H-DNP-------- 236
Ver.UPI00025FB25F           GGDRIPADIRVTSAH-GCKV--DNSSLTGE-SEPQSRSPDCT-----H-DNP-------- 223
Ver.UPI0000E3A2FA           GGDRIPADIRVVSAH-GCKV--DNSSLTGE-SEPQSRSPDCT-----H-DNP-------- 236
Ver.UPI00003628C3           GGDRIPADIRVVSAH-GCKV--DNSSLTGE-SEPQSRSPDCT-----H-DNP-------- 233
Ver.AGR87394.1              GGDRIPADIRVVSAH-GCKV--DNSSLTGE-SEPQNRSPDCT-----H-DNP-------- 222
Ver.XP_020504733.1          GGDRIPADIRVVSAH-GCKV--DNSSLTGE-SEPQSRSPDCT-----H-DNP-------- 238
Ver.AHB86586.1              GGDRIPADIRVVSAH-GCKV--DNSSLTGE-SEPQSRSPDCT-----H-DNP-------- 223
Ver.UPI00032B9010           GGDRIPADIRVVSAH-GCKV--DNSSLTGE-SEPQSRSPDCT-----H-DNP-------- 223
Ver.XP_022612296.1          GGDRIPADIRVVSAH-GCKV--DNSSLTGE-SEPQSRSPDCT-----H-DNP-------- 223
Ver.XP_023285663.1          GGDRIPADIRVVSAH-GCKV--DNSSLTGE-SEPQSRSPDCT-----H-DNP-------- 236
Ver.NP_571763.1             GGDRIPADIRIVSSQ-GCKV--DNSSLTGE-SEPQTRAPEMS-----S-DNP-------- 237
Ver.UPI00025FADDE           GGDRIPADLRIISAH-GCKV--DNSSLTGE-SEPQTRAPDFS-----H-ENP-------- 236
Ver.UPI0000318264           GGDRIPADLRIISAH-GCKV--DNSSLTGE-SEPQTRTPDFS-----N-DNP-------- 236
Ver.ELK38498.1              GGDRIPADIRLISAQ-GCKV--DNSSLTGE-SNPQTRSPDFT-----H-ENP-------- 196
Ver.XP_545754.3             GGDRIPADLRLISTQ-GCKV--DNSSLTGE-SEPQTRSPDFT-----H-ENP-------- 246
Ver.ELK30843.1              GGDRIPADLRIISAN-GCKV--DNSSLTGE-SEPQTRSPDFT-----H-ENP-------- 326
Ver.XP_023390675.1          GGDRIPADLRIISAN-GCKV--DNSSLTGE-SEPQTRSPDFT-----N-ENP-------- 205
Ver.EMP33651.1              GGDRIPADLRIISAH-GCKV--DNSSLTGE-SEPQTRSPDFT-----N-ENP-------- 116
Ver.AHD24596.1              GGDRIPADLRIVSAH-GCKV--DNSSLTGE-SEPQTRSPECS-----S-DNP-------- 237
Ver.UPI00001261C4           GGDRIPADLRIISAC-SCKV--DNSSLTGE-SEPQSRSPEYS-----S-ENP-------- 236
Ver.Q98SL3                  GGDRILADVRFISCS-GCKV--DNSSLTGE-SEPQSRSPDFT-----H-ENP-------- 222
Ver.NP_0011179              GGDRIPADLRVVSAA-GCKV--DNSSLTGE-SEPQTRTPEFT-----H-ENP-------- 226
Ver.UPI0006B74D9B           GGDRIPADLRVVSAA-GCKV--DNSSLTGE-SEPQTRTPEFT-----H-ENP-------- 225

Ver.XP_020796901.1          GGDRIPADLRVISSS-GCKV--DNSSLTGE-SEPQTRSPDFT-----H-ENP-------- 268
Ver.NP_571758.1             GGDRVPADLRIISSS-GCKV--DNSSLTGE-SEPQTRSPEFT-----H-ENP-------- 231
Ver.XP_005796664.1          GGDRVPADLRVISSS-GCKV--DNSSLTGE-SEPQTRSPELT-----H-ENP-------- 224
Ver.UPI00000FE1CF           GGDRVPADLRVTSSS-GCKV--DNSSLTGE-SEPQTRSPELT-----H-ENP-------- 222
Ver.AHB86585.1              GGDRIPADLRVISSS-GCEV--DNSSLTGE-SEPQTRSPEFT-----H-DNP-------- 223
Ver.KKF24497.1              GGDRIPADLRVISSS-GCKV--DNSSLTGE-SEPQTRSPEFT-----H-ENP-------- 224
Ver.XP_020507674.1          GGDRIPADLRVISSS-GCKV--DNSSLTGE-SEPQTRSPEFT-----H-DNP-------- 79
Ver.XP_023135081.1          GGDRIPADLRVISSS-GCKV--DNSSLTGE-SEPQTRSPEFT-----H-DNP-------- 224
Ver.BAO02373.1              GGDRIPADLRVVSSS-GCKV--DNSSLTGE-SEPQTRSPEFT-----H-ENP-------- 224
Ver.UPI0003D8328F           GGDRIPADLRIISSN-SCKV--DNSSLTGE-SEPQTRSPEFT-----H-ENP-------- 232
Ver.NP_0010831              GGDRIPADLRITVAH-GCKV--DNSSLTGE-SEPQTRSPEFT-----H-ENP-------- 234
Ver.UPI0000F6BCEB           GGDRVPADIRIIIAH-GCKV--DNSSLTGE-SEPQTRSPEFT-----H-ENP-------- 234
Ver.ELK38499.1              GGDRVPADLRIISSH-GCKV--DNSSLTGE-SEPQTRSPEFT-----H-ENP-------- 234
Ver.EPQ02424.1              GGDRVPADLRIISSH-GCKV--DNSSLTGE-SEPQTRSPEFT-----H-ENP-------- 262
Ver.XP_023616468.1          GGDRVPADLRIISSH-GCKV--DNSSLTGE-SEPQTRSPEFT-----H-ENP-------- 234
Ver.XP_021054982.1          GGDRVPADLRIISSH-GCKV--DNSSLTGE-SEPQTRSPEFT-----H-ENP-------- 234
Ver.NP_036637.              GGDRVPADLRIISSH-GCKV--DNSSLTGE-SEPQTRSPEFT-----H-ENP-------- 234
Ver.XP_004858786.1          GGDRVPADLRIISSH-GCKV--DNSSLTGE-SEPQTRSPEFT-----H-ENP-------- 234
Ver.XP_003466610.1          GGDRVPADLRIISSH-GCKV--DNSSLTGE-SEPQTRSPEFT-----H-ENP-------- 234
Ver.XP_004639996.1          GGDRVPADLRIISSH-GCKV--DNSSLTGE-SEPQTRSPEFT-----H-ENP-------- 234
Ver.XP_005339432.1          GGDRVPADLRIISSH-GCKV--DNSSLTGE-SEPQTRSPEFT-----H-ENP-------- 234
Ver.XP_003795245.1          GGDRVPADLRIISSH-GCKV--DNSSLTGE-SEPQTRSPEFT-----H-ENP-------- 234
Ver.XP_008056914.2          GGDRVPADLRIISSH-GCKV--DNSSLTGE-SEPQTRSPEFT-----H-ENP-------- 234
Ver.XP_012604635.1          GGDRVPADLRIISSH-GCKV--DNSSLTGE-SEPQTRSPEFT-----H-ENP-------- 234
Ver.XP_004390257.1          GGDRVPADLRIISSH-GCKV--DNSSLTGE-SEPQTRSPEFT-----H-ENP-------- 234
Ver.XP_020024807.1          GGDRVPADLRIISSH-GCKV--DNSSLTGE-SEPQTRSPEFT-----H-ENP-------- 234
Ver.NP_0011253              GGDRVPADLRIISSH-GCKV--DNSSLTGE-SEPQTRSPEFT-----H-ENP-------- 234
Ver.XP_023069989.1          GGDRVPADLRIISSH-GCKV--DNSSLTGE-SEPQTRSPEFT-----H-ENP-------- 143
Ver.PNI19721.1              GGDRVPADLRIISSH-GCKV--DNSSLTGE-SEPQTRSPEFT-----H-ENP-------- 234
Ver.NP_000693.              GGDRVPADLRIISSH-GCKV--DNSSLTGE-SEPQTRSPEFT-----H-ENP-------- 234
Ver.NP_0012526              GGDRVPADLRIISSH-GCKV--DNSSLTGE-SEPQTRSPEFT-----H-ENP-------- 234
Ver.XP_021537506.1          GGDRVPADLRIISSH-GCKV--DNSSLTGE-SEPQTRSPEFT-----H-ENP-------- 295
```

```
Ver.UPI0002B2E326        GGDRVPADLRIISSH-GCKV--DNSSLTGE-SEPQTRSPEFT-----H-ENP-------- 227
Ver.XP_003415228.1       GGDRVPADLRIISSH-GCKV--DNSSLTGE-SEPQTRSPEFT-----H-ENP-------- 234
Ver.XP_004448489.1       GGDRVPADLRIISSH-GCKV--DNSSLTGE-SEPQTRSPEFT-----H-ENP-------- 234
Ver.XP_006922964.1       GGDRVPADLRIISSH-GCKV--DNSSLTGE-SEPQTRSPEFT-----H-ENP-------- 234
Ver.XP_545753.3          GGDRVPADLRIISSH-GCKV--DNSSLTGE-SEPQTRSPEFT-----H-ENP-------- 234
Ver.XP_019677883.4       GGDRVPADLRIISSH-GCKV--DNSSLTGE-SEPQTRSPEFT-----H-ENP-------- 234
Ver.NP_0011650           GGDRVPADLRIISSH-GCKV--DNSSLTGE-SEPQTRSPEFT-----H-ENP-------- 234
Ver.NP_0010749           GGDRVPADLRIISSH-GCKV--DNSSLTGE-SEPQTRSPEFT-----H-ENP-------- 234
Ver.XP_020740887.1       GGDRVPADLRIISSH-GCKV--DNSSLTGE-SEPQTRSPEFT-----H-ENP-------- 234
Ver.XP_022415031.1       GGDRVPADLRIISSH-GCKV--DNSSLTGE-SEPQTRSPEFT-----H-ENP-------- 234
Ver.XP_007129684.1       GGDQVPADLRIISSH-GCKV--DNSSLTGE-SEPQTRSPEFT-----H-ENP-------- 234
Ver.UPI000226F4AA        GGDRVPADLRIISSH-GCKV--DNSSLTGE-SEPQTRSPEFT-----H-ENP-------- 234
Ver.UPI00005E9366        GGDRVPADLRIISSH-GCKV--DNSSLTGE-SEPQTRSPEFT-----H-ENP-------- 234
Ver.XP_020835237.1       GGDRVPADLRIISSH-GCKV--DNSSLTGE-SEPQTRSPEFT-----H-ENP-------- 234
Ver.XP_020653823.1       GGDRVPADLRIISSH-GCKV--DNSSLTGE-SEPQTRSPEFT-----H-ENP-------- 234
Ver.XP_026576074.1       GGDRIPADLRVISSH-GCKV--DNSSLTGE-SEPQTRSPEFT-----H-ENP-------- 234
Ver.UPI0000124FC0        GGDRVPADMRIISSH-GCKV--DNSSLTGE-SEPQTRSPEFT-----H-ENP-------- 231
Ver.XP_005293820.1       GGDRVPADLRIISSH-GCKV--DNSSLTGE-SEPQTRSPEFT-----H-ENP-------- 234
Ver.KYO43368.1           GGDRVPADLRIISSH-GCKV--DNSSLTGE-SEPQTRSPEFT-----H-ENP-------- 240
Ver.XP_006038189.1       GGDRVPADLRIISSH-GCKV--DNSSLTGE-SEPQTRSPEFT-----H-ENP-------- 234
Ver.AGR45921.1           GGDRIPADLRIISAH-GCKV--DNSSLTGE-SEPQTRSPEFS-----N-DNP-------- 237
Ver.sp|P30714.2|         GGDRIPADLRIISAH-GCKV--DNSSLTGE-SEPQTRSPDFT-----N-ENP-------- 236
Ver.NP_0010840           GGDRIPADLRVISSH-GCKV--DNSSLTGE-SEPQTRSPDFT-----N-ENP-------- 238
Ver.NP_989407.1          GGDRIPADVRIISAH-GCKV--DNSSLTGE-SEPQTRSPDFT-----N-ENP-------- 236
Ver.XP_004853865.1       GGDRIPADLRIISAN-GCKV--DNSSLTGE-SEPQTRSPDFT-----N-DNP-------- 237
Ver.ACB20771.2           GGDRIPADLRIISAN-GCKV--DNSSLTGE-SEPQTRSPDFT-----N-ENP-------- 237
Ver.XP_023557491.1       GGDRIPADLRIISAN-GCKV--DNSSLTGE-SEPQTRSPDFT-----N-ENP-------- 206
Ver.ERE90024.1           GGDRIPADLRIISAN-GCKV--DNSSLTGE-SEPQTRSPDFT-----N-ENP-------- 256
Ver.XP_005076578.1       GGDRIPADLRIISAN-GCKV--DNSSLTGE-SEPQTRSPDFT-----N-ENP-------- 236
Ver.AAA41671.1           GGDRIPADLRIISAN-GCKV--DNSSLTGE-SEPQTRSPDFT-----N-ENP-------- 236
Ver.XP_021504168.1       GGDRIPADLRIISAN-GCKV--DNSSLTGE-SEPQTRSPDFT-----N-ENP-------- 236
Ver.XP_021051287.1       GGDRIPADLRIISAN-GCKV--DNSSLTGE-SEPQTRSPDFT-----N-ENP-------- 236
Ver.XP_021013125.1       GGDRIPADLRIISAN-GCKV--DNSSLTGE-SEPQTRSPDFT-----N-ENP-------- 236
Ver.NP_659149.           GGDRIPADLRIISAN-GCKV--DNSSLTGE-SEPQTRSPDFT-----N-ENP-------- 236
Ver.EPQ03777.1           GGDRIPADLRIISAN-GCKV--DNSSLTGE-SEPQTRSPDFT-----H-ENP-------- 270
Ver.UPI000C2F2801        GGDRIPADLRIISAN-GCKV--DNSSLTGE-SEPQTRSPDFT-----N-ENP-------- 240
Ver.XP_020858281.1       GGDRIPADLRIISAN-GCKV--DNSSLTGE-SEPQTRSPDFT-----N-ENP-------- 234

Ver.XP_004380410.1       GGDRIPADLRIISAN-GCKV--DNSSLTGE-SEPQTRSPDFT-----N-ENP-------- 234
Ver.AGY54951.1           GGDRIPADLRIISAN-GCNV--DKSSLTGE-SEPQTRSPDFT-----N-ENP-------- 234
Ver.UPI0001FB338F        GGDRIPADLRIISAN-GCKV--DNSSLTGE-SEPQTRSPDFT-----N-ENP-------- 234
Ver.NP_0011565           GGDRIPADLRIISAN-GCKV--DNSSLTGE-SEPQTRSPDFT-----N-ENP-------- 236
Ver.XP_010587900.1       GGDRIPADLRIISAN-GCKV--DNSSLTGE-SEPQTRSPDFT-----N-ENP-------- 205
Ver.UPI0002B3D77C        GGDRIPADLRIISAN-GCKV--DNSSLTGE-SEPQTRSPDFT-----N-ENP-------- 230
Ver.NP_0010702           GGDRIPADLRIISAN-GCKV--DNSSLTGE-SEPQTRSPDFT-----N-ENP-------- 234
Ver.OWK04910.1           GGDRIPADLRIISAN-GCKV--DNSSLTGE-SEPQTRSPDFT-----N-ENP-------- 205
Ver.UPI0000124FBE        GGDRIPADLRIISAN-GCKV--DNSSLTGE-SEPQTRSPDFT-----N-ENP-------- 234
Ver.NP_0010093           GGDRIPADLRIISAN-GCKV--DNSSLTGE-SEPQTRSPDFT-----N-ENP-------- 234
Ver.XP_020747989.1       GGDRIPADLRIISAN-GCKV--DNSSLTGE-SEPQTRSPDFT-----N-ENP-------- 234
Ver.XP_020012504.1       GGDRIPADLRIISAN-GCKV--DNSSLTGE-SEPQTRSPDFT-----N-ENP-------- 236
Ver.XP_005334975.1       GGDRIPADLRIISAN-GCKV--DNSSLTGE-SEPQTRSPDFT-----N-ENP-------- 236
Ver.NP_0010033           GGDRIPADLRIISAN-GCKV--DNSSLTGE-SEPQTRSPDFT-----N-ENP-------- 234
Ver.XP_022352592.1       GGDRIPADLRIISAN-GCKVQVDNSSLTGE-SEPQTRSPDFT-----N-ENP-------- 236
Ver.XP_011283388.1       GGDRIPADLRIISAN-GCKV--DNSSLTGE-SEPQTRSPDFT-----N-ENP-------- 234
Ver.XP_024426171.1       GGDRIPADLRIISAN-GCKV--DNSSLTGE-SEPQTRSPDFT-----N-ENP-------- 234
Ver.XP_023975434.1       GGDRIPADLRIISAN-GCKV--DNSSLTGE-SEPQTRSPDFT-----N-ENP-------- 234
Ver.XP_022439684.1       GGDRIPADLRIISAN-GCKV--DNSSLTGE-SEPQTRSPDFT-----N-ENP-------- 234
Ver.XP_024620662.1       GGDRIPADLRIISAN-GCKV--DNSSLTGE-SEPQTRSPDFT-----N-ENP-------- 234
Ver.XP_006919736.1       GGDRIPADLRIISAN-GCKV--DNSSLTGE-SEPQTRSPDFT-----N-ENP-------- 205
Ver.UPI000C746E0C        GGDRIPADLRIISAN-GCKV--DNSSLTGE-SEPQTRSPDFT-----N-ENP-------- 247
Ver.UPI000C2E744C        GGDRIPADLRIISAN-GCKV--DNSSLTGE-SEPQTRSPDFT-----N-ENP-------- 240
Ver.XP_020944376.1       GGDRIPADLRIISAN-GCKV--DNSSLTGE-SEPQTRSPDFT-----N-ENP-------- 234
Ver.XP_008071711.2       GGDRIPADLRIISAN-GCKV--DNSSLTGE-SEPQTRSPDFT-----N-ENP-------- 241
Ver.XP_012663099.1       GGDRIPADLRIISAN-GCKV--DNSSLTGE-SEPQTRSPDFT-----N-ENP-------- 236
Ver.UPI000C2E4C26        GGDRIPADLRIISAN-GCKV--DNSSLTGE-SEPQTRSPDFT-----N-ENP-------- 232
Ver.XP_012617266.1       GGDRIPADLRIISAN-GCKV--DNSSLTGE-SEPQTRSPDFT-----N-ENP-------- 236
Ver.XP_012314296.1       GGDRIPADLRIISAN-GCKV--DNSSLTGE-SEPQTRSPDFT-----N-ENP-------- 236
Ver.UPI0001C9F9BA        GGDRIPADLRIISAN-GCKV--DNSSLTGE-SEPQTRSPDFT-----N-ENP-------- 236
Ver.NP_000692.           GGDRIPADLRIISAN-GCKV--DNSSLTGE-SEPQTRSPDFT-----N-ENP-------- 236
Ver.XP_008971666.1       GGDRIPADLRIISAN-GCKV--DNSSLTGE-SEPQTRSPDFT-----N-ENP-------- 205
Ver.XP_016780478.1       GGDRIPADLRIISAN-GCKV--DNSSLTGE-SEPQTRSPDFT-----N-ENP-------- 236
Ver.XP_023078532.1       GGDRIPADLRIISAN-GCKV--DNSSLTGE-SEPQTRSPDFT-----N-ENP-------- 236
```

```
Ver.NP_0012536          GGDRIPADLRIISAN-GCKV--DNSSITGE-SEPQTRSPDFT-----N-ENP-------- 236
Ver.PNJ46178.1          GGDRIPADLRIISAN-GCKV--DNSSITGE-SEPQTRSPDFT-----N-ENP-------- 236
Ver.ETE67008.1          GGDRIPADLRIISAN-GCKV--DNSSITGE-SEPQTRSPDCT-----N-DNP-------- 235
Ver.XP_007435355.1      GGDRIPADLRIISAN-GCKV--DNSSITGE-SEPQTRSPDCT-----N-DNP-------- 205
Ver.XP_020645227.1      GGDRIPADLRIISAH-GCKV--DNSSITGE-SEPQTRSPDFT-----N-ENP-------- 205
Ver.XP_006132947.1      GGDRIPADLRIISAH-GCKV--DNSSITGE-SEPQTRSPDFT-----N-ENP-------- 205
Ver.XP_005292736.1      GGDRIPADLRIISAH-GCKV--DNSSITGE-SEPQTRSPDFT-----N-ENP-------- 237
Ver.XP_024064252.1      GGDRIPADLRIISAH-GCKV--DNSSITGE-SEPQTRSPDFT-----N-ENP-------- 237
Ver.XP_025067531.1      GGDRIPADLRIVSAH-GCKV--DNSSITGE-SEPQTRSPDFT-----N-ENP-------- 205
Ver.NP_990852.          GGDRIPADLRIISAH-GCKV--DNSSITGE-SEPQTRSPDFS-----N-ENP-------- 234
Ver.XP_021253236.1      GGDRIPADLRIISAH-GCKV--DNSSITGE-SEPQTRSPDFS-----N-ENP-------- 234
Ver.UPI00051ECCC0       GGDRIPADLRIISAH-GCKV--DNSSITGE-SEPQTRSPDFT-----N-ENP-------- 229
Ver.KFU90062.1          GGDRIPADLRIISAH-GCKV--DNSSITGE-SEPQTRSPEFS-----N-ENP-------- 231
Ver.XP_030317504.1      GGDRIPADLRIISAH-GCKV--DNSSITGE-SEPQTRSPDFS-----N-ENP-------- 231
Ver.KFP84941.1          GGDRIPADLRIISAH-GCKV--DNSSITGE-SEPQTRSPDFS-----N-ENP-------- 231
Ver.XP_030327328.1      GGDRIPADLRIISAH-GCKV--DNSSITGE-SEPQTRSPDFS-----N-ENP-------- 234
Ver.XP_023796730.1      GGDRIPADLRIISAH-GCKV--DNSSITGE-SEPQTRSPDFS-----H-ENP-------- 205
Ver.XP_021404823.1      GGDRIPADLRIISAH-GCKV--DNSSITGE-SEPQTRSPDFS-----H-ENP-------- 234
Ver.XP_025966334.1      GGDRIPADLRIISAH-GCKV--DNSSITGE-SEPQTRSPDFT-----N-ENP-------- 234
Ver.UPI0004FDA0CB       GGDRIPADLRIISAH-GCKV--DNSSITGE-SEPQTRSPDFS-----N-ENP-------- 232
Ver.KQK85052.1          GGDRIPADLRIISAH-GCKV--DNSSITGE-SEPQTRSPDFS-----H-ENP-------- 243
Ver.NP_0012973          GGDRIPADLRIISAH-GCKV--DNSSITGE-SEPQTRSPDFS-----N-ENP-------- 236
Ver.KFW61640.1          GGDRIPADLRIISAH-GCKV--DNSSITGE-SEPQTRSPDFS-----N-ENP-------- 231
Ver.XP_005511501.1      GGDRIPADLRIISAH-GCKV--DNSSITGE-SEPQTRSPDFS-----H-ENP-------- 205
Ver.OPJ66608.1          GGDRIPADLRIISAH-GCKV--DNSSITGE-SEPQTRSPDFS-----H-ENP-------- 237
Ver.UPI000056D0DB       GGDRIPADLRIISAH-GCKV--DNSSITGE-SEPQTRSPEFS-----S-ENP-------- 241
Ver.UPI0006B827F3       GGDRIPADLRIVSAS-GCKV--DNSSITGE-SEPQTRTPDFS-----N-DNP-------- 271
Ver.XP_004066575.1      GGDRVPADLRIVSSH-GCKV--DNSSITGE-SEPQTRSPEFS-----N-ENP-------- 235
Ver.XP_024144685.1      GGDRIPADLRIVSAH-GCKV--DNSSITGE-SEPQTRSPEFS-----N-ENP-------- 235
Ver.UPI0006B31231       GGDRIPADLRIISAH-GCKV--DNSSITGE-SEPQTRSPEFS-----N-ENP-------- 235
Ver.UPI0006B37238       GGDRIPADLRIISAH-GCKV--DNSSITGE-SEPQTRSPEFS-----N-ENP-------- 238
Ver.ADD60471.1          GGDRIPADLRIVSAQ-GCKV--DNSSITGE-SEPQTRTPDFS-----N-DNP-------- 235
Ver.UPI000157ACC7       GGDRIPADLRIISAH-GCKV--DNSSITGE-SEPQTRTPDFS-----N-DNP-------- 238
Ver.AAT48993.1          GGDRIPADLRIISAH-GCKV--DNSSITGE-SEPQTRTPDFS-----N-DNP-------- 236
Ver.XP_020476182.1      GGDRIPADLRIISAH-GCKV--DNSSITGE-SEPQTRTPEFT-----N-DNP-------- 239

Ver.UPI00079E81D7       GGDRIPADLRIISSH-GCKV--DNSSITGE-SEPQTRSPDFT-----N-DNP-------- 236
Ver.UPI000274DF5C       GGDRIPADLRIISAH-GCKV--DNSSITGE-SEPQTRTPDFS-----N-DNP-------- 229
Ver.NP_0012969          GGDRIPADLRIISSH-GCKV--DNSSITGE-SEPQTRSPDFT-----N-DNP-------- 236
Ver.UPI000443A733       GGDRIPADLRIISAH-GCKV--DNSSITGE-SEPQTRSPEFT-----N-DNP-------- 236
Ver.XP_023185631.1      GGDRIPADLRVISAQ-GCKV--DNSSITGE-SEPQTRSPDFT-----N-DNP-------- 236
Ver.ALA65287.2          GGDRIPADLRIISAH-GCKV--DNSSITGE-SEPQTRTPDFS-----N-DNP-------- 235
Ver.AKQ12834.1          GGDRIPADLRIISAS-GCKV--DNSSITGE-SEPQTRTPDFS-----N-DNP-------- 238
Ver.XP_023275950.1      GGDRIPADLRIISAH-GCKV--DNSSITGE-SEPQTRTPDFS-----N-DNP-------- 237
Ver.ADB03120.1          GGDRIPADLRIISAH-GCKV--DNSSITGE-SEPQTRSPDFS-----N-ENP-------- 236
Ver.UPI00022B0848       GGDRIPADLRIISAH-GCKV--DNSSITGE-SEPQTRSPDFS-----N-ENP-------- 236
Ver.AGZ87948.1          GGDRIPADLRIISAH-GCKV--DNSSITGE-SEPQTRSPDFS-----N-ENP-------- 236
Ver.sp|Q9YH26.2|        GGDRIPADLRIISAH-GCKV--DNSSITGE-SEPQTRSPDFS-----N-ENP-------- 236
Ver.AGO02179.1          GGDRIPADLRIISAH-GCKV--DNSSITGE-SEPQTRSPDFS-----N-ENP-------- 236
Ver.AHB86584.1          GGDRIPADLRIVSAH-GCKV--DNSSITGE-SEPQTRTPDFS-----N-DNP-------- 236
Ver.XP_023121557.1      GGDRIPADLRIVSAH-GCKV--DNSSITGE-SEPQTRTPDFS-----N-DNP-------- 236
Ver.XP_022078036.1      GGDRIPADLRIISAH-GCKV--DNSSITGE-SEPQTRTPDFS-----N-DNP-------- 236
Ver.XP_020792263.1      GGDRIPADLRIISAH-GCKV--DNSSITGE-SEPQTRTPDFS-----N-ENP-------- 237
Ver.AGR87393.1          GGDRIPADLRIISAH-GCKV--DNSSITGE-SEPQTRTPDFS-----N-ENP-------- 237
Ver.ABF58911.1          GGDRIPADLRIISAH-GCKV--DNSSITGE-SEPQTRSPDFS-----N-DNP-------- 237
Ver.Q90X33              GGDRIPADLRIISAH-GCKV--DNSSITGE-SEPQTRTPDFS-----N-DNP-------- 237
Ver.XP_022530668.1      GGDRIPADLRIISAH-GCKV--DNSSITGE-SEPQTRTPDFS-----N-DNP-------- 237
Ver.AJR20271.1          GGDRIPADLRIISAH-GCKV--DNSSITGE-SEPQTRTPDFS-----N-DNP-------- 253
Ver.XP_020328431.1      GGDRIPADLRIISAS-GCKV--DNSSITGE-SEPQTRTPDYS-----N-DNP-------- 238
Ver.XP_021426673.1      GGDRIPADLRIISAS-GCKV--DNSSITGE-SEPQTRTPDYS-----N-DNP-------- 238
Ver.XP_023653512.1      GGDRIPADLRIISAH-GCKV--DNSSITGE-SEPQTRSPDFS-----N-DNP-------- 237
Ver.sp|Q92030.1|        GGDRIPADLRVASAQ-GCKV--DNSSITGE-SEPQTRSPDFS-----N-ENP-------- 235
Ver.ALB35496.1          GGDRIPADLRVVSAQ-GCKV--DNSSITGE-SEPQTRSPDFS-----N-ENP-------- 235
Ver.NP_571761.1         GGDRIPADLRIISAH-GCKV--DNSSITGE-SEPQTRTPDFS-----N-DNP-------- 239
Ver.AJR20270.1          GGDRIPADLRIISAH-GCKV--DNSSITGE-SEPQTRTPDFS-----N-DNP-------- 239
Ver.XP_023690671.1      GGDRIPADLRIISAH-GCKV--DNSSITGE-SEPQTRSPDFS-----N-ENP-------- 237
Ver.XP_008322794.1      GGDRIPADLRIISAH-GCKV--DNSSITGE-SEPQTRTPDFS-----N-DNP-------- 268
Ver.XP_017282368.1      GGDRIPADLRIISAH-GCKV--DNSSITGE-SEPQTRTPDFS-----N-ENP-------- 241
Ver.XP_004066573.1      GGDRIPADLRIISAN-GCKV--DNSSITGE-SEPQTRTPDFS-----N-ENP-------- 237
Ver.XP_024144684.1      GGDRIPADLRIISAH-GCKV--DNSSITGE-SEPQTRTPDFS-----N-ENP-------- 237
Ver.XP_020497843.1      GGDRIPADLRIISAH-GCKV--DNSSITGE-SEPQTRTPDFS-----N-DNP-------- 249
Ver.UPI000027C768       GGDRIPADLRIISAH-GCKV--DNSSITGE-SEPQTRTPDFS-----N-ENP-------- 232
```

```
Ver.XP_004571307.1          GGDRIPADLRIISAH-GCKV--DNSSLTGE-SEPQTRTPDFS-----N-ENP-------- 237
Ver.XP_012714443.1          GGDRIPADLRIISAH-GCKV--DNSSLTGE-SEPQTRTPDFS-----N-ENP-------- 237
Ver.XP_023185013.1          GGDRIPADLRIISAH-GCKV--DNSSLTGE-SEPQTRTPDFS-----N-ENP-------- 237
Ver.UPI00066EFDEA           GGDRIPADLRIISAH-GCKV--DNSSLTGE-SEPQTRTPDFS-----N-ENP-------- 241
Ver.XP_022617258.1          GGDRIPADLRIISAH-GCKV--DNSSLTGE-SEPQTRTPDFS-----N-ENP-------- 237
Ver.BAN17691.1              GGDRIPADLRIISAH-GCKV--DNSSLTGE-SEPQTRTPDFS-----N-ENP-------- 237
Ver.sp|P25489.1|            GGDRIPADLRIISSH-GCKV--DNSSLTGE-SEPQTRSPDFS-----N-DNP-------- 239
Ver.XP_022536277.1          GGDRIPADLRIISAH-GCKV--DNSSLTGE-SEPQTRSPDFS-----N-DNP-------- 237
Ver.Q9DEU1                  GGDRIPADLRIIASH-GCKV--DNSSLTGE-SEPQTRSPDYS-----N-DNP-------- 237
Ver.ACB20770.2              GGDRIPADLRIISAN-GCKV--DNSSLTGE-SEPQTRSPDFT-----N-ENP-------- 224
Ver.NP_835200.1             GGDKIPADIRIVSSH-GCKV--DNSSLTGE-SEPQIRTPDMS-----S-ENP-------- 236
Ver.NP_571762.1             GGDRIPADLRIVYAQ-GCKV--DNSSLTGE-SEPQSRSPEFS-----H-ENP-------- 236
Ver.BAJ13363.1              GGDRIPADLRIVSAS-GCKV--DNSSLTGE-SEPQTRSPDFS-----N-DNH-------- 241
Ver.UPI00001DFF4A           GGDRIPADLRIVSAS-GCKV--DNSSLTGE-SEPQTRSPDFS-----N-DNP-------- 241
Ver.UPI00002BAA33           GGDRVPADLRVISSS-GCKV--DNSSLTGE-SEPQTRSPEPT-----H-DNP-------- 222
Ver.XP_021506251.1          GGDQIPADIRVIAAQ-GCKV--DNSSLTGE-SEPQSRCPDCT-----H-ENP-------- 244
Ver.NP_074039.              GGDQVPADIRVIAAQ-GCKV--DNSSLTGE-SEPQSRCPDCT-----H-ENP-------- 242
Ver.XP_021014708.1          GGDQIPADIRVISAQ-GCKV--DNSSLTGE-SEPQSRCPDCT-----H-ENP-------- 246
Ver.NP_038762.              GGDQIPADIRVISAQ-GCKV--DNSSLTGE-SEPQSRCPDCT-----H-ENP-------- 246
Ver.XP_020024800.1          GGDRIPADLRLIYAQ-RCKV--DNSSLTGE-SEPQSRSTDFT-----H-ENP-------- 239
Ver.XP_003795244.1          GGDRIPADLRIISSQ-GFKV--DNSSLTGE-SEPQTRSPEFT-----H-ENP-------- 245
Ver.XP_004639995.1          AGDRIPADIRLISAQ-GCKV--DNSSLTGE-SEPQSRSPNFT-----H-ENP-------- 250
Ver.XP_004448484.1          GGDRIPADLRLISAQ-GCKV--DNSSLTGE-SEPQTRTPEFT-----H-ENP-------- 245
Ver.XP_023103614.1          GGDRIPADLRLISAQ-GCKV--DNSSLTGE-SEPQSRSPDFS-----H-ENP-------- 246
Ver.XP_023496657.1          GGDRIPADIRLISAQ-GCKV--DNSSLTGE-SEPQSRFPHFT-----H-ENL-------- 251
Ver.XP_021537588.1          GGDRIPADLRLISSQ-GCKV--DNSSLTGE-SEPQTRSPEFT-----H-ENP-------- 245
Ver.XP_006096963.1          GGDRIPADIRLISAQ-GCKV--DNSSLTGE-SNPQTRSPDFT-----H-ENP-------- 246
Ver.XP_006922963.1          GGDRIPADLRLISAQ-GCKV--DNSSLTGE-SEPQSRSPDFT-----H-ENP-------- 245
Ver.XP_011371380.1          GGDRIPADLRLISAQ-GCKV--DNSSLTGE-SEPQTRSPDFT-----H-ENP-------- 245
Ver.NP_0011375              GGDRIPADIRLISSQ-GCKV--DNSSLTGE-SEPQSRSTEFT-----N-ENP-------- 245
Ver.XP_020740848.1          GGDRIPADIRLISSE-GCKV--DNSSLTGE-SEPQSRSPEFT-----N-ENP-------- 245
Ver.XP_010593170.2          GGDRIPADIRLISSQ-GCKV--DNSSLTGE-SEPQSRSPEFT-----H-ENP-------- 205
Ver.XP_021568356.1          GGDRIPADLRIISAQ-GCKV--DNSSLTGE-SEPQFRSPDFS-----H-ENP-------- 244

Ver.XP_012604632.1          GGDRIPADLRLISAQ-GCKV--DNSSLTGE-SEPQSRCPDFT-----N-ENP--------  243
Ver.NP_653300.              GGDRVPADLRLISAQ-GCKV--DNSSLTGE-SEPQSRSPDFT-----H-ENP--------  244
Ver.XP_003892961.1          GGDRVPADLRLISAQ-GCKV--DNSSLTGE-SEPQTRSPDFT-----H-ENP--------  244
Ver.XP_023069991.1          GGDRVPADLRLIFAQ-GCKV--DNSSLTGE-SEPQSRSPDFT-----H-ENP--------  244
Ver.UPI0001C650F7           GGDRIPADLRLISSQ-GCKV--DNSSLTGE-SEPQSRSPDFT-----H-ENP--------  242
Ver.XP_013220247.1          GGDRIPADLRLISSQ-GCKV--DNSSLTGE-SEPQSRSPDFT-----H-ENP--------  202
Art.EFX69525.1(Group II)    FGDRLPADILILSCS-NFKV--DNSSLTGE-SEPQTRSPECT-----H-NNP--------  217
Rinv.CRX73232.1(Group II)   SGNRIPADVRIIESR-GMKV--DNSSLTGE-SEPQSRNIECT-----S-DNP--------  205
Prt.XP_001742517.1          SGDRVPADLRIIECA-DLKV--DNSSLTGE-SEPQKRGTECT-----D-ENP--------  243
Prt.EGD73524.1              SGDRIPADLRLIEVQ-GLKV--DNSSLTGE-SEPQKRSPECT-----D-VNP--------  338
Rinv.PIS80793.1             YGDRIPADIRLIYSS-SLKV--DNSSLTGE-SEPQSRSANCT-----D-PNP--------  248
Art.AAF17586.1              FGDRVPADIRILEAH-GLKV--DNSSLTGE-SEPQVRSTEFT-----H-ENP--------  225
Art.AFU25666.1(Group II)    FGDRVPADVRIIEAH-NFKV--DNSSLTGE-TEPQPRDSAVS-----K-VQV--------  220
Art.AFU25665.1              FGDRIPADLRIIESH-SFKV--DNSSLTGE-SEPQSRTPEFT-----H-DDP--------  261
Art.XP_021915175.1          FGDRIPADVRIIESR-GFKV--DNSSLTGE-SEPQSRGPEFT-----H-ENP--------  256
Art.EFN85240.1              FGDRIPADIRIIEAR-GFKV--DNSSLTGE-SEPQSRSPEFT-----N-ENP--------  293
Art.OXA63786.1(Group II)    LGDRIPADIRIVEAK-SFKV--DNSSLTGE-R----------------------------  202
Art.ODN01960.1(Group II)    FGDRIPADIRIIESK-GLKV--DNSSLTGE-SEPQRREPKCT-----N-ENP--------  222
Rinv.XP_020602016.1         FGDRVPADIRVLEAR-GFKV--DNSSLTGE-SEPQARTPEFS-----H-DNP--------  50
Art.ODM98837.1              FGDRIPADIRVLESH-GFKV--DNSSLTGE-SEPQTRSPECT-----H-DNP--------  215
Art.AGZ13696.1-             FGDRVPADIRILQAH-AMKV--DNSSLTGE-SEPQPRSPEAT-----N-DNP--------  232
Rinv.G4VGA0                 CGDRVPADIRIISAS-SFKV--DNSALTGE-SEPQSRTSEYT-----N-ENP--------  232
Rinv.KXJ20388.1             IGDRVPADIRILESK-GFKV--DNSSLTGE-SEPQARTPEFT-----H-ENP--------  242
Nem.CDW54807.1              GGDRIPADIRLIVAH-GLKV--DNSSLTGE-SDPQSRTTECT-----N-SNP--------  191
Nem.KRZ50957.1              GGDRIPADIRLIVSH-GLKV--DNSSLTGE-SDPQSRVAECT-----N-KNP--------  301
Nem.KRX38989.1              GGDRIPADIRLIVSH-GLKV--DNSSLTGE-SDPQSRVAECT-----N-KNP--------  301
Nem.KRY10799.1              GGDRIPADIRLIVSH-GLKV--DNSSLTGE-SDPQSRVAECT-----N-KNP--------  330
Nem.KRY70033.1              GGDRIPADIRLIVSH-GLKV--DNSSLTGE-SDPQSRVAECT-----N-KNP--------  244
Nem.KRZ74907.1              GGDRIPADIRLIVSH-GLKV--DNSSLTGE-SDPQSRVTECT-----N-KNP--------  244
Nem.KRZ04306.1              GGDRIPADIRLIVSH-GLKV--DNSSLTGE-SDPQSRVTECT-----N-KNP--------  244
Nem.KRX24043.1              GGDRIPADIRLIVSH-GLKV--DNSSLTGE-SDPQSRVAECT-----N-KNP--------  301
Nem.KRY48181.1              GGDRIPADIRLIVSH-GLKV--DNSSLTGE-SDPQSRVAECT-----N-KNP--------  244
Nem.KRY33278.1              GGDRIPADIRLIVSH-GLKV--DNSSLTGE-SDPQSRVAECT-----N-KNP--------  277
Rinv.OQV18895.1(Group II)   GGDRIPADLRMIEVK-GMKV--DNSSLTGE-SEPQSRSLESH-------PNP--------  229
Nem.XP_003369418.1          GGDRVPADIRIIAAQ-SFKV--DNSSLTGE-SEPQSRSAECT-----NVENP--------  281
Nem.UPI00060630B9           GGDRIPADVRIIYAM-GLKV--DNASLTGE-SEPQSRSTECT-----S-ERP--------  269
Nem.UPI000609E432           SGDRVPADIRILHAT-GFKV--DNSSLTGE-SEPQSRSPECT-----N-ENP--------  213
Nem.UPI00060EAC2D           SGDRVPADIRIIEAS-SFKV--DNASLTGE-SEPQSRSPECT-----N-PNP--------  212
```

```
Nem.UPI000601D02E              GGDRIPADMRIISSF-GLKV--DNSSLTGE-SEPQSRSPICT-----S-ENP-------- 213
Nem.UPI00060CD0E5              GGDRIPADIRIITSF-GLKV--DNSSLTGE-SEPQSRSPICT-----S-ENP-------- 213
Nem.UPI000183E9C3              GGDRVPADIRVISAF-GFKV--DNSSLTGE-SEPQSRSPDCT-----N-ENP-------- 235
Nem.UPI0007A1BF54              GGDRVPADIRIISAF-GFKV--DNSSLTGE-SEPQSRSPECT-----N-ENP-------- 246
Rinv.NP_0012967                FGDRIPADIRIVECK-GLKV--DNSSLTGE-SEPQSRAVDFT-----H-ENP-------- 242
Rinv.OQV17561.1(Group II)      GGDRIPADLRIVECR-GMKV--DNSSLTGE-SEPQSRSAQNT-----H-SNP-------- 282
Art.EFX71103.1                 AGNRIPADIRILGAH-QFKV--DNSSLTGE-SEPQSRGPEYT-----S-DNP-------- 215
Art.EFX71104.1                 FGDRIPADIRVLEAR-QFKV--DNSSLTGE-SEPQSRSPEFT-----N-DNP-------- 215
Art.EFX71105.1                 FGDRVPADIRILEAR-QFKV--NNSSLTGE-SEPQSRTPEFT-----N-NNP-------- 229
Art.ODM98254.1                 FGDRIPADIRIVESR-GFKV--DNSSLTGE-SEPQSRGADFT-----H-DNP-------- 218
Art.XP_023347331.1             FGDRVPADIRVLEAR-SFKV--DNSSLTGE-SEPQARSPEFT-----H-ENP-------- 218
Art.XP_015929949.2             GGDRIPADIRIISAS-GFKV--DNSSLTGE-SEPQTRSPDMT-----N-ENP-------- 229
Art.XP_023221169.1             GGDRIPADIRIVCAQ-SFKV--DNSSLTGE-SEPQTRNSECT-----H-ENP-------- 135
Art.XP_023221168.1             GGDRIPADIRVISSQ-SCKV--DNSSLTGE-SEPQTRSPELT-----N-ENP-------- 297
Art.XP_022253133.1             GGDRIPADIRVISSH-SFKV--DNSSLTGE-SEAQTRSPELT-----N-DNP-------- 253
Art.XP_022254094.1             GGDRIPADIRVLVSR-SFKV--DNSSLTGE-SEPQTRSPEMT-----H-ENP-------- 273
Rinv.ALJ53300.1                FGDRVPADIRVVQAH-GFKV--DNSSLTGE-SEPQARGPDFT-----N-ENP-------- 236
Rinv.BAA32798.1                FGDRVPADIRVIQAS-SFKV--DNSALTGE-SEAQVRTPEFT-----N-DNP-------- 235
Rinv.XP_018651572.1            FGDRIPADIRIITAS-SFKV--DNSALTGE-SEPQSRSTEFS-----N-ENP-------- 230
Rinv.AAL09322.1                CGDRVPADIRIISAS-SFKV--DNSALTGE-SEPQSRTSEYT-----N-ENP-------- 220
Rinv.sp|Q6RWA9.1|              FGDRVPADIRVIKAS-SFKV--DNSALTGE-SEPQTRTAEYT-----N-ENP-------- 228
Rinv.CDS22215.1                FGDRVPADIRVIKAS-SFKV--DNSALTGE-SEPQTRTAEYT-----N-ENP-------- 239
Rinv.CDS36343.1                FGDRVPADIRVIKAS-SFKV--DNSALTGE-SEPQTRTAEYT-----N-ENP-------- 239
Rinv.AAX09623.1                FGDRIPADIRVISSH-GFKV--DNSSLTGE-SEPQSRTPEFS-----N-ENP-------- 236
Rinv.ABO61333.1                FGDRIPSDIRVISAH-SFKV--DNSSLTGE-SEPQSRSAEFT-----N-ENP-------- 241
Rinv.ABO61332.1                FGDRIPADIRVVAAH-SFKV--DNSSLTGE-SEPQSRSAEFT-----N-ENP-------- 241
Rinv.EKC34610.1                FGDRVPADVRVITAH-GFKV--DNSSLTGE-SEPQTRTADFT-----N-DNP-------- 280
Rinv.XP_022323941.1            FGDRVPADVRVITAH-GFKV--DNSSLTGE-SEPQTRTAEFT-----N-DNP-------- 246
Rinv.XP_013405520.1            FGDRVPADIRVIKAH-GFKV--DNSSLTGE-SEPQTRLPEFT-----S-ENP-------- 259
Rinv.ELU12040.1                FGDRVPADVRVISAH-GFKV--DNSSLTGE-SEPQSRSSDFT-----H-ENP-------- 245
Rinv.AUG84438.1                FGDRIPADIRVVYAN-SFKV--DNSSLTGE-SEPQTRTAEFT-----N-DNP-------- 239

Rinv.NP_001116982.1            GGDRIPADVRVVESK-SFKV--DNSSLTGE-SEPQSRSPEFT-----S-DNP-------- 248
Nem.KHJ49479.1                 GGDRVPADVRIIASH-GFKV--DNSSLTGE-SEAQSRGVECT-----NPDNP-------- 108
Nem.CDW55413.1                 GGDRVPADVRIIASH-GFKV--DNSSLTGE-SEAQSRGVECT-----NPDNP-------- 254
Nem.KRY76685.1                 GGDRVPADIRIIAAQ-SFKV--DNSSLTGE-SEPQSRSAECT-----NIENP-------- 281
Nem.KRZ73739.1                 GGDRVPADIRIIAAQ-SFKV--DNSSLTGE-SEPQSRSAECT-----NMENP-------- 283
Nem.KRX35740.1                 GGDRVPADIRIIAAQ-SFKV--DNSSLTGE-SEPQSRSAECT-----NVENP-------- 283
Nem.KRZ52541.1                 GGDRVPADIRIIAAQ-SFKV--DNSSLTGE-SEPQSRSAECT-----NVENP-------- 285
Nem.UPI0006120BCD              GGDRIPADIRVISCH-GFKV--DNSSLTGE-SEPHSLSAECT-----N-ENP-------- 282
Nem.UPI0007A15AF4              GGDRVPADVRVISAH-GFKV--DNSSLTGE-SEPQSRSAEFT-----N-DNP-------- 189
Nem.UPI00020239EC              GGDRVPADIRIISAH-GFKV--DNSSLTGE-SEPQSRSQECT-----S-ENP-------- 272
Nem.KHN72407.1                 GGDRVPADIRIISAH-GFKV--DNSSLTGE-SEPQSRNHECT-----N-ENP-------- 271
Nem.UPI0006052FD1              GGDRVPADIRVIYAM-GFKV--DNASLTGE-SEPQSRSPECT-----N-ENP-------- 213
Nem.UPI000609F1C5              SGDRVPADIRVISAS-GFKV--DNASLTGE-SEPQSRSPECT-----N-ENP-------- 213
Nem.UPI0006036B9D              GGDRVPADIRIISSF-GFKV--DNSSLTGE-SEPQSRSPECT-----N-ENP-------- 213
Nem.XP_024502753.1             GGDRVPADMRIISSF-GFKV--DNSSLTGE-SEPQSRSPECT-----N-ENP-------- 196
Nem.UPI000605CA49              GGDRVPADLRIISSF-GFKV--DNSSLTGE-SEPQSRSNECT-----H-ENP-------- 218
Nem.UPI000609E51C              GGDRVPADLRVISAS-GFKV--DNSSLTGE-SEPQARSSECT-----N-ENP-------- 172
Nem.UPI0007A24416              GGDRVPADLRIISAS-GFKV--DNSSLTGE-SEPQARSPECT-----N-ENP-------- 189
Nem.XP_003143231.1             GGDRVPADLRIISAS-GFKV--DNSSLTGE-SEPQARSPECT-----N-ENP-------- 213
Nem.UPI0007085A3B              GGDRVPADLRIISAS-GFKV--DNSSLTGE-SEPQARSPECT-----N-ENP-------- 225
Nem.OZC08885.1                 GGDRVPADLRIISAS-GFKV--DNSSLTGE-SEPQARSPECT-----N-ENP-------- 213
Nem.UPI00060602BA              GGDRVPADLRIISAS-GFKV--DNSSLTGE-SEPQARSPECT-----N-ENP-------- 226
Nem.UPI000605F508              GGDRVPADLRIISAS-GFKV--DNSSLTGE-SEPQARSPECT-----N-ENP-------- 189
Nem.XP_001901816.1             GGDRVPADLRIISAS-GFKV--DNSSLTGE-SEPQARSPECT-----N-ENP-------- 213
Nem.UPI000818DDE2              GGDRVPADLRIISSS-GFKV--DNSSLTGE-SEPQTRSPECT-----N-ENP-------- 231
Nem.UPI0007A17F26              GGDRVPADLRIITAF-GFKV--DNSSLTGE-SEPQSRSPECT-----N-ENP-------- 213
Nem.UPI0007A19C37              GGDRVPADIRVISAF-GFKV--DNSSLTGE-SEPQTRSPECT-----N-ENP-------- 211
Nem.UPI000605DBB4              GGDRVPADIRVISAF-GFKV--DNSSLTGE-SEPQSRSPECT-----N-ENP-------- 211
Nem.KHN82508.1                 GGDRVPADIRVISSF-GFKV--DNSSLTGE-SEPQSRSPECT-----N-ENP-------- 213
Nem.ADY40856.1                 GGDRVPADIRIISAF-GFKV--DNSSLTGE-SEPQSRSPECT-----N-ENP-------- 213
Nem.ADY40930.1                 GGDRVPADIRVVSAF-GFKV--DNSSLTGE-SEPQSRSPECT-----N-ENP-------- 222
Nem.UPI000BC5B284              GGDRVPADIRIISAF-GFKV--DNSSLTGE-SEPQSRSPDCT-----S-DNP-------- 172
Nem.UPI000BE6443D              GGDRVPADIRVISAF-GFKV--DNSSLTGE-SEPQSRNAECT-----N-ENP-------- 226
Nem.UPI0001D4FB7A              GGDRVPADIRVISAF-GFKV--DNSSLTGE-SEPQSRNAECT-----N-ENP-------- 214
Nem.UPI0006101D56              GGDRVPADIRESVHI-FHEV--DNSSLTGE-SEPQSRTPECT-----N-ENP-------- 184
Nem.UPI0001C851CE              GGDRVPADIRIVSAF-GFKV--DNSSLTGE-SEPQSRSPDCT-----N-ENP-------- 214
Nem.P90735                     GGDRVPADLRVVSAF-GFKV--DNSSLTGE-SEPQSRSPDCT-----N-ENP-------- 214
Nem.UPI000293EB87              GGDRVPADIRVISAF-GFKV--DNSSLTGE-SEPQSRSPDCT-----N-ENP-------- 214
Nem.UPI00060544A6              GGDRVPADIRVISAF-GFKV--DNSSLTGE-SEPQTRTPECT-----N-ENP-------- 234
Nem.UPI0007A2DD20              GGDRVPADIRVISAF-GFKV--DNSSLTGE-SEPQSRSPECT-----N-ENP-------- 215
```

```
Nem.UPI000342C523               GGDRVPADIRIISAF-GFKV--DNSSLTGE-SEPQSRSPECT-----N-ENP-------- 279
Nem.U6PGW0                      GGDRVPADIRVISAF-GFKV--DNSSLTGE-SEPQTRTPECT-----N-ENP-------- 215
Nem.UPI00060AD39A               GGDRVPADIRVISAF-GFKV--DNSSLTGE-SEPQTRTPECT-----N-ENP-------- 215
Rinv.UPI0001782835(Group II)    SGDRIPADIRIIEAR-SMKV--DNSSLTGE-SEPQVRLPECT-----N-DNP-------- 205
Rinv.KXJ20422.1                 GGDRLPGDLRVMEAK-SFKV--DNSSLTGE-SEPQSRSPDCT-----S-DNP-------- 309
Rinv.XP_020601998.1             GGDRVPADLRVVEAR-GFKV--DNSSLTGE-SEPQSRGPDCT-----N-ENP-------- 274
Rinv.XP_020602001.1             GGDRVPADLRVVEAR-GFKV--DNSSLTGE-SEPQSRGPDCT-----N-ENP-------- 259
Rinv.AOG19177.1                 FGDRVPADIRIISAH-GFKV--DNSSLTGE-SEPQVRTAECT-----S-ENP-------- 196
Art.XP_015930974.1              GGDRIPADMRIINSS-GCKV--DNSSLTGE-SEPQTRTPEAS-----N-DNP-------- 222
Art.XP_015907346.1              GGDRIPADIRIISAS-SCKV--DNSSLTGE-SEPQSRSPEMT-----N-ENP-------- 256
Art.XP_015929951.2              GGDRVPADIRVISAQ-GFKV--DNSSLTGE-SEPQTRSPEMT-----N-ENP-------- 257
Art.UPI0009F0198D               GGDRVPADMRVIQAQ-GFKV--DNSSLTGE-SEPQTRSPEMT-----N-DNP-------- 279
Art.XP_022668602.1              GGDRVPADMRVIQAQ-GFKV--DNSSLTGE-SEPQTRSPEMT-----N-ENP-------- 252
Art.UPI000B76A669               GGDRIPADMRVIQSQ-GFKV--DNSSLTGE-SEPQTRSPELT-----N-ENP-------- 251
Art.UPI0007AA68F1               GGDRIPADMRVIQAQ-GFKV--DNSSLTGE-SEPQSRSPEMT-----N-ENP-------- 245
Art.AMK38059.1                  GGDRIPADMRVIQAQ-GFKV--DNSSLTGE-SEPQTRSPEMT-----N-ENP-------- 248
Art.UPI0007717391               GGDRIPADMRVIQAQ-GFKV--DNSSLTGE-SEPQTRSPELT-----N-ENP-------- 246
Art.UPI00079F518A               GGDRIPADMRVIQGQ-GFKV--DNSSLTGE-SEPQTRSPELT-----N-ENP-------- 247
Art.UPI000790B508               FGDRIPADIRIIESR-GFKV--DNSSLTGE-SEPQSRSPEFT-----H-ENP-------- 255
Art.KPI92424.1                  FGDRIPADIRIIEAR-GFKV--DNSSLTGE-SEPQSRGAEFT-----N-ENP-------- 276
Art.AGZ13694.1(Group II)        FGDRVPADVRIIEAR-NMKV--DNSSLTGE-SEPQSRSPEFT-----A-ENP-------- 256
Art.UPI000672CD46               FGDRVPADMRLLESR-GFKV--DNSSLTGE-SEPQSRSTEFT-----N-DNP-------- 214
Art.XP_023337795.1              FGDRLPADVRVLEAR-GFKV--DNSSLTGE-SEPCSRSPEFT-----H-ENP-------- 218
Art.XP_023336146.1              FGDRIPADLRVLESK-GFKV--DNSSLTGE-SEPQSRGPEFT-----H-ENP-------- 215
Art.XP_023323782.1              FGDRLPADIRVVEAR-GFKV--DNSSLTGE-SEPQSRGPEYT-----H-ENP-------- 79
Art.AFU25682.1                  FGDRIPADIRIIESR-GFKV--DNSSLTGE-SEPQSRGIEMT-----N-DNP-------- 221
Art.AFU25689.1                  FGDRIPADIRIIESR-GFKV--DNSSLTGE-SEPQSRGIEMT-----H-DNP-------- 221
Art.EFX88073.1                  FGDRIPADIRVIEAR-QFKV--DNSSLTGE-SEPQSRSPEFT-----N-DNP-------- 217
Art.EFX88361.1                  FGDRIPADIRIIEAR-SFKV--DNSSLTGE-SEPQSRSSEFT-----N-ENP-------- 205

Art.XP_021944101.1              FGDRIPADIRVIESR-GFKV--DNSSLTGE-SEPQSRGSDFT-----H-DNP-------- 214
Art.AIM43570.1                  FGDRIPADIRVIEAR-GFKV--DNSSLTGE-SEPQSRSPEFT-----S-DNP-------- 226
Art.AFM54541.1                  FGDRIPADIRVIEAR-GFKV--DNSSLTGE-SEPQSRSPEFT-----S-ENP-------- 222
Art.AEX07319.1                  FGDRIPADIRVIESR-GFKV--DNSSLTGE-SEPQSRSPEYT-----S-ENP-------- 224
Art.AIR93635.1                  FGDRIPADIRVIESR-GFKV--DNSSLTGE-SEPQSRSPEYT-----S-ENP-------- 251
Art.ADN83843.1                  FGDRIPADIRVIESR-GFKV--DNSSLTGE-SEPQSRSPEYT-----S-ENP-------- 251
Art.ABD59803.1                  FGDRIPADIRVIESR-GFKV--DNSSLTGE-SEPQSRSSEYT-----S-ENP-------- 251
Art.AAG47843.1                  FGDRIPADMRVIEAR-GFKV--DNSSLTGE-SEPQSRSPEFT-----S-ENP-------- 252
Art.AGF90965.1                  FGDRIPADMRVIEAR-GFKV--DNSSLTGE-SEPQSRSPEFT-----S-ENP-------- 252
Art.AGM39710.1                  FGDRIPADVRVIEAR-GFKV--DNSSLTGE-SEPQSRSSEFT-----S-ENP-------- 250
Art.ABA02167.1                  FGDRIPADVRVTEAR-GFKV--DNSSLTGE-SEPQSRSAEFT-----S-ENP-------- 223
Art.UPI0000085D74               FGDRIPADVRVIEAR-GFKV--DNSSLTGE-SEPQSRSPEFT-----S-ENP-------- 251
Art.AJO70000.1                  FGDRIPADVRVIEAR-GFKV--DNSSLTGE-SEPQSRSPEFT-----S-ENP-------- 251
Art.AJO70183.1                  FGDRIPADVRVIEAR-GFKV--DNSSLTGE-SEPQSRSPEFT-----S-ENP-------- 251
Art.AKG50106.1                  FGDRIPADVRVIEAR-GFKV--DNSSLTGE-SEPQSRSPEFT-----S-ENP-------- 251
Art.KZC06498.1                  FGDRIPADIRIIESR-GFKV--DNSSLTGE-SEPQSRSPEFT-----N-ENP-------- 220
Art.KYN38456.1                  FGDRIPADIRIIEAR-GFKV--DNSSLTGE-SEPQSRSPEFT-----N-ENP-------- 220
Art.EGI67709.1                  FGDRIPADIRIIEAR-GFKV--DNSSLTGE-SEPQSRSPEFT-----N-ENP-------- 220
Art.KYQ51534.1                  FGDRIPADIRIIEAR-GFKV--DNSSLTGE-SEPQSRSPEFT-----N-ENP-------- 220
Art.XP_014232354.1              FGDRIPADLRIIESR-GFKV--DNSSLTGE-SEPQSRSPDFT-----H-ENP-------- 257
Art.XP_014205893.1              FGDRIPADLRIIESR-GFKV--DNSSLTGE-SEPQSRSPEFT-----N-ENP-------- 257
Art.XP_012269651.1              FGDRIPADIRIIESR-GFKV--DNSSLTGE-SEPQSRSPEFT-----N-ENP-------- 254
Art.XP_015585114.1              FGDRIPADIRIIESR-GFKV--DNSSLTGE-SEPQSRSPEFT-----N-ENP-------- 254
Art.V9I6A9                      FGDRIPADIRIIESR-GFKV--DNSSLTGE-SEPQSRSPEFT-----N-ENP-------- 227
Art.XP_012163873.1              FGDRIPADIRIIESR-GFKV--DNSSLTGE-SEPQSRSPEFT-----N-ENP-------- 254
Art.XP_012272094.1              FGDRIPADIRIIESQ-GFKV--DNSSLTGE-SEPQSRSPEFT-----N-ENP-------- 254
Art.UPI0001FEE5BA               FGDRIPADIRIIEAR-GFKV--DNSSLTGE-SEPQSRSPEFT-----N-ENP-------- 261
Art.UPI0005FA3D38               FGDRIPADIRIIEAR-GFKV--DNSSLTGE-SEPQSRSPEFT-----N-ENP-------- 248
Art.XP_020294182.1              FGDRIPADIRIIESR-GFKV--DNSSLTGE-SEPQSRSPEFT-----N-ENP-------- 255
Art.EZA51212.1                  FGDRIPADIRIIEAR-GFKV--DNSSLTGE-SEPQSRSPEFT-----N-ENP-------- 254
Art.UPI0001E7C907               FGDRIPADIRIIESR-GFKV--DNSSLTGE-SEPQSRSPEFT-----N-ENP-------- 220
Art.XP_024883961.1              FGDRIPADIRIIEAR-GFKV--DNSSLTGE-SEPQSRSPEFT-----N-ENP-------- 254
Art.UPI00091A11F9               FGDRIPADIRIIESR-NFKV--DNSSLTGE-SEPQSRGPEFT-----H-ENP-------- 214
Art.UPI00091201A3               FGDRIPADIRIIESR-NFKV--DNSSLTGE-SEPQSRGPEFT-----H-ENP-------- 252
Art.UPI000971E6B0               FGDRIPADIRIIEAR-SFKV--DNSSLTGE-SEPQSRGPDFT-----H-ENP-------- 253
Art.T1E1Y4                      FGDRIPADIRIIEAR-NFKV--DNSSLTGE-SEPQSRSPEFT-----H-ENP-------- 213
Art.XP_021693479.1              FGDRLPADIRIIEAR-NFKV--DNSSLTGE-SEPQSRGPDFT-----H-ENP-------- 253
Art.ETN62539.1                  FGDRIPADIRIIEAR-NFKV--DNSSLTGE-SEPQSRGPDFT-----H-ENP-------- 213
Art.UPI000CD7670C               FGDRIPADIRIIEAR-NFKV--DNSSLTGE-SEPQSRGPDFT-----H-ENP-------- 248
Art.UPI0007D6117E               FGDRIPADVRIIEAR-NFKV--DNSSLTGE-SEPQSRGPDFT-----H-ENP-------- 218
Art.UPI000153A0D9               FGDRIPADIRIIEAR-NFKV--DNSSLTGE-SEPQSRGPDFT-----H-ENP-------- 213
Art.UPI0007D2379C               FGDRIPADVRIIEAR-NFKV--DNSSLTGE-SEPQSRGPDFT-----H-ENP-------- 244
```

```
Art.UPI000957746B                  FGDRIPADIRIIEAR-NFKV--DNSSLTGE-SEPQSRGFEFT-----H-ENP-------- 237
Art.UPI000692F512                  FGDRIPADIRIIEAR-NFKV--DNSSLTGE-SEPQSRGPEFT-----H-ENP-------- 251
Art.T1PH35                         FGDRIPADIRIIEAR-TFKV--DNSSLTGE-SEPQSRGPEYT-----H-ENP-------- 215
Art.KNC28219.1                     FGDRIPADIRIIEAR-NFKV--DNSSLTGE-SEPQSRGPEFT-----H-ENP-------- 250
Art.UPI0005476AAF                  FGDRIPADIRIIEAR-NFKV--DNSSLTGE-SEPQSRGPEFT-----H-ENP-------- 251
Art.UPI000692F5BD                  FGDRIPADIRIIEAR-NFKV--DNSSLTGE-SEPQSRGPEFT-----H-ENP-------- 215
Art.XP_004536046.2                 FGDRIPADVRIIEAR-NFKV--DNSSLTGE-SEPQSRGAEFT-----H-ENP-------- 215
Art.A0A034W3G9                     FGDRIPADIRIIEAR-NFKV--DNSSLTGE-SEPQSRGPEFT-----H-ENP-------- 249
Art.UPI0006929A96                  FGDRIPADIRIIEAR-NFKV--DNSSLTGE-SEPQSRGPEFT-----H-ENP-------- 251
Art.XP_004536048.1                 FGDRIPADVRIIEAR-NFKV--DNSSLTGE-SEPQSRGAEFT-----H-ENP-------- 215
Art.UPI0005474384                  FGDRIPADIRIIEAR-NFKV--DNSSLTGE-SEPQSRGPEFT-----H-ENP-------- 215
Art.UPI0006ED933E                  FGDRIPADIRIIEAR-NFKV--DNSSLTGE-SEPQSRGSEFT-----H-ENP-------- 256
Art.UPI0006EDF4F9                  FGDRIPADIRIIEAR-NFKV--DNSSLTGE-SEPQSRGSEFT-----H-ENP-------- 252
Art.UPI0006BD3860                  FGDRIPADIRIIEAR-NFKV--DNSSLTGE-SEPQSRGAEFT-----H-ENP-------- 215
Art.XP_023176860.1                 FGDRIPADIRIIEAR-NFKV--DNSSLTGE-SEPQSRGSEFT-----H-ENP-------- 215
Art.UPI0006D327FB                  FGDRIPADIRIIEAR-NFKV--DNSSLTGE-SEPQSRGAEFT-----H-ENP-------- 254
Art.UPI00017C692D                  FGDRIPADIRIIEAR-TFKV--DNSSLTGE-SEPQSRGAEFT-----H-ENP-------- 255
Art.UPI0006EE4186                  FGDRIPADIRIIEAR-NFKV--DNSSLTGE-SEPQSRGAEFT-----H-ENP-------- 250
Art.XP_022210861.1                 FGDRIPADIRIIEAR-TFKV--DNSSLTGE-SEPQSRNAEFT-----H-ENP-------- 254
Art.UPI0007E80B92                  FGDRIPADIRIIEAR-TFKV--DNSSLTGE-SEPQSRGAEFT-----H-ENP-------- 254
Art.UPI0001781834                  FGDRIPADIRIIEAR-NFKV--DNSSLTGE-SEPQSRGAEFT-----H-ENP-------- 254
Art.NP_732572.                     FGDRIPADIRIIEAR-NFKV--DNSSLTGE-SEPQSRGAEFT-----H-ENP-------- 254
Art.UPI00017D261C                  FGDRIPADIRIIEAR-NFKV--DNSSLTGE-SEPQSRGAEFT-----H-ENP-------- 254
Art.XP_020799552.1                 FGDRIPADIRIIEAR-TFKV--DNSSLTGE-SEPQSRGAEFT-----H-ENP-------- 254
Art.XP_015032883.1                 FGDRIPADIRIIEAR-TFKV--DNSSLTGE-SEPQSRGAEFT-----H-ENP-------- 254
Art.AFU25675.1                     FGDRIPADIRIIEAR-GFKV--DNSSLTGE-SEPQSRGPEFT-----N-ENP-------- 222
Art.AFU25676.1                     FGDRIPADIRIIEAR-GFKV--DNSSLTGE-SEPQSRGPEFT-----N-ENP-------- 222

Art.BAS22117.1                     FGDRIPADIRIIEAR-GFKV--DNSSLTGE-SEPQSRGAEFT-----N-ENP-------- 222
Art.AFU25681.1                     FGDRIPADIRIIEAR-GFKV--DNSSLTGE-SEPQSRGPEFT-----N-ENP-------- 222
Art.XP_023954931.1                 FGDRIPADVRIIEAR-GFKV--DNSSLTGE-SEPQSRGPEFT-----N-ENP-------- 254
Art.XP_022114884.1                 FGDRIPADVRIIESR-GFKV--DNSSLTGE-SEPQSRGAEFT-----N-ENP-------- 254
Art.AFU25678.1                     FGDRIPADVRIIESR-GFKV--DNSSLTGE-SEPQSRGPEFT-----N-ENP-------- 222
Art.UPI00028A5EEF                  FGDRIPADIRIIESR-GFKV--DNSSLTGE-SEPQSRGAEFT-----N-ENP-------- 249
Art.AFU25667.1                     FGDRIPADIRIIESR-GFKV--DNSSLTGE-SEPQSRGAEFT-----N-ENP-------- 249
Art.AFU25673.1                     FGDRIPADIRIIEAR-GFKV--DNSSLTGE-SEPQSRGAEFT-----N-ENP-------- 222
Art.AFU25679.1                     FGDRIPADIRIIEAR-GFKV--DNSSLTGE-SEPQSRGADFT-----N-ENP-------- 254
Art.UPI000B392785                  FGDRIPADIRIIEAR-GFKV--DNSSLTGE-SEPQSRGSEFT-----H-ENP-------- 222
Art.AFU25694.1                     FGDRIPADIRIIEAR-GFKV--DNSSLTGE-SEPQSRGAEFT-----H-ENP-------- 249
Art.XP_021196082.1                 FGDRIPADIRIIEAR-GFKV--DNSSLTGE-SEPQSRGSEFT-----H-ENP-------- 254
Art.XP_022817943.1                 FGDRIPADIRIIEAR-GFKV--DNSSLTGE-SEPQSRGAEFT-----H-ENP-------- 254
Art.AFU25670.1                     FGDRIPADIRIIESR-GFKV--DNSSLTGE-SEPQSRSPEFT-----N-ENP-------- 218
Art.XP_022903571.1                 FGDRIPADIRIIESR-GFKV--DNSSLTGE-SEPQSRSPEFT-----H-ENP-------- 249
Art.UPI00084EC8B0                  FGDRIPADIRIIESR-GFKV--DNSSLTGE-SEPQSRSSEFT-----H-ENP-------- 221
Art.U4UIT1                         FGDRIPADIRIIETR-GFKV--DNSSLTGE-SEPQSRSPEFT-----H-ENP-------- 265
Art.XP_023016793.1                 FGDRIPADIRIIESR-GFKV--DNSSLTGE-SEPQSRSPEFT-----H-ENP-------- 255
Art.AFU25671.1                     FGDRIPADVRIIESR-GFKV--DNSSLTGE-SEPQSRSPEFT-----H-ENP-------- 256
Art.AFU25692.1                     FGDRIPADIRIIESR-GFKV--DNSSLTGE-SEPQSRSPEFT-----H-ENP-------- 257
Art.UPI000B551D17                  FGDRIPADIRIIESR-GFKV--DNSSLTGE-SEPQSRSPEFT-----H-ENP-------- 221
Art.AFU25686.1                     FGDRIPADIRIIESR-GFKV--DNSSLTGE-SEPQSRSPEFT-----H-ENP-------- 224
Art.AFU25695.1                     FGDRIPADIRIIESR-GFKV--DNSSLTGE-SEPQSRSPEFT-----H-ENP-------- 248
Art.XP_018562050.1                 FGDRIPADIRIIESR-GFKV--DNSSLTGE-SEPQSRSPEFT-----H-ENP-------- 255
Art.UPI00028AEC41                  FGDRIPADIRIIESR-GFKV--DNSSLTGE-SEPQSRSPEFT-----H-ENP-------- 218
Art.AFU25691.1                     FGDRIPADIRIIESR-GFKV--DNSSLTGE-SEPQSRSPEFT-----H-ENP-------- 221
Art.UPI000186D98E                  FGDRIPADIRIFESR-GFKV--DNSSLTGE-SEPQSRGVEFT-----H-ENP-------- 248
Art.XP_023711705.1                 FGDRIPADIRIIESR-GFKV--DNSSLTGE-SEPQSRGPEFT-----H-ENP-------- 274
Art.W5U4R1                         FGDRIPADIRIIESR-GFKV--DNSSLTGE-SEPQSRSPEFT-----H-ENP-------- 225
Art.AHH35009.1                     FGDRIPADIRIIESR-GFKV--DNSSLTGE-SEPQSRSPEFT-----H-ENP-------- 225
Art.AFU25683.1                     FGDRIPADIRIIEAR-GFKV--DNSSLTGE-SEPQSRGIEMT-----H-ENP-------- 221
Art.XP_022184331.1                 FGDRIPADIRIIEAR-GFKV--DNSSLTGE-SEPQSRGIDFT-----H-ENP-------- 252
Art.UPI00028BBB9D                  FGDRIPADIRIIEAR-GFKV--DNSSLTGE-SEPQSRGIENT-----H-ENP-------- 205
Art.UPI00028AA5CE                  FGDRIPADIRIIEAR-GFKV--DNSSLTGE-SEPQSRGVENT-----H-ENP-------- 207
Art.UPI0005464DB9                  FGDRIPADIRIIEAR-GFKV--DNSSLTGE-SEPQSRGVDLT-----N-ENP-------- 226
Art.AFU25668.1                     FGDRIPADIRIIEAR-GFKV--DNSSLTGE-SEPQSRGVELT-----N-DNP-------- 250
Art.XP_014271921.1                 FGDRIPADIRIIEAR-GFKV--DNSSLTGE-SEPQSRGVELT-----N-ENP-------- 223
Art.XP_014250371.1                 FGDRIPADIRIIEAR-GFKV--DNSSLTGE-SEPQSRGVELT-----N-DNP-------- 229
Art.UPI0007325ED7                  FGDRIPADIRIIEAR-GFKV--DNSSLTGE-SEPQSRGVELT-----N-DNP-------- 249
Prt.CAI99406.1                     DGDQVPADIRVIAAT-DLKV--DNSSLTGE-SEPQTRVPDVE-----HGTDENGNPKFIP 221
Prt.UPI0004A1BCA5                  DGDQVPADICVKKST-DLKV--DNSSLTGE-SEPQDRCNELM-----KDS--SG-QPITQ 224
Prt.UPI00014FFB3C                  DGDQVPADIRVMRSN-ELKV--DNSSLTGE-SEPQDRTPELA-----VDS--NG-NIVTQ 219
Prt.A0A090M1W3                     DGDQVPADIRVMKSN-ELKV--DNSSLTGE-SEPQDRSPELA-----RTA--TG-ELVTQ 219
Prt.OUS42873.1                     DGDQVPADIRVMKSN-ELKV--DNSSLTGE-SEPQDRSPELA-----RTA--TG-ELVTQ 219
```

```
Art.OWR44555.1(Group I)           GGEVVPADVRILSCS-NFKT--DMSSLTGE-SEPIVHRPEYT------NANP-------- 240
Art.KPJ02649.1(Group I)           SGEIVPADIRVLESN-NFKT--DMSSLTGE-SKPVKHTPKCT------NKNP-------- 239
Art.XP_004931505.2(Group I)       AGDIVPADIRIIESK-GFKV--DNSSLTGE-SIAVLRANVEG------TSNI-------- 175
Art.OWR53886.1(Group I)           IGDVLPADIRIIDSK-GFKV--DNSSLTGE-SVALPRSNTEG------TENI-------- 242
Art.KPJ07193.1(Group I)           AGDVIPADIRIIESK-GFKV--DISSLTGE-SRAVTRSNTEG------TTNI-------- 238
Art.KPI97351.1(Group I)           AGDVIPADIRIIDSK-GFKV--DNSSLTGE-SRAVTRSNTEG------TTNI-------- 238
Art.OXA57033.1                    YGDKLPADVRIIECQ-SFKV--DNSSITGE-SDPQSRTAICT------DKDADG--RKVE 268
Art.ODM90865.1                    LGDLIPADIRIIDCQ-NFKV--DNSSLTGE-SEAQKRSRECT------SSDP-------- 288
Art.ODM96221.1                    YGDLVPADIRILECQ-NFTV--DNSALTGE-AEPQKRLSTCT------DDNP-------- 189
Art.ODM99113.1                    FGDLIPADIRILESH-DFKV--DNSSLTGE-SEPQKRSPECT------HDEP-------- 238
Rinv.OQV20867.1                   AGDKIPADIRIVENR-GLKV--DMASLTGE-SEPLSRTVEGT------NENP-------- 254
Art.K7IWP3(Group II)              LGDKIPADIRIIECH-GLRV--ENSSITGE-SEPTTRTDYPT------DNNP-------- 234
Art.EZA47803.1(Group II)          MGDKIPADIRIIECS-GLRV--ENSSITGE-SEPVVRTDYPT------DPNP-------- 225
Art.KYN03549.1(Group II)          MGDKIPADIRIIECR-GLRV--ENSSITGE-SEPVARTDYPT------DRNP-------- 235
Art.KYQ48574.1(Group II)          MGDKIPADVRIIECH-GLRV--ENSSITGE-SEPVARTNYPT------DRNP-------- 235
Art.KZC11054.1(Group II)          FGDKIPADIRITECR-GLRV--ENSSITGE-SEAVVRTNYPT------DDNP-------- 228
Art.KOX77187.1(Group II)          TGDKIPADIRIIECQ-GLRV--ENSSITGE-SEPVVRTNYPT------DENP-------- 229
Art.OAD46911.1(Group II)          TGDKIPADIRIIQCR-GLRV--ENSSITGE-SEPVPRTDYPS------DENP-------- 230
Art.KOC67548.1(Group II)          NGDKIPADIRIIECR-ELRV--ENSSITGE-SEPVTRTNYPT------DENP-------- 232
Art.OXA54637.1(Group II)          FGDSIPADIRIIECQ-GLKV--DNSSLTGE-SEPQSRSPLCS------DDNP-------- 284
Art.ODN05419.1(Group II)          FGDRIPADIRIIECS-GFKV--DNSSLTGE-SEPQSRSPQCT------DENP-------- 266
Prt.EGD77429.1                    AGDRIPADLRIIDEH-GLKV--DNSSLTGE-SEPQKRSAECT------HKNP-------- 423
Nem.CEF64940.1(Group II)          SGDRVAGDIRIVKSA-GLRV--DNSSLTGE-SEPQSRSIEFS------NDNP-------- 221
Prt.EGD75712.1                    YGNKVPADIRILEAS-NLKV--DNSSLTGE-SEPQKRSPECT------HEDF-------- 303
Rinv.OQV25202.1                   MGDRMPADIVILEAQ-AFKV--DNSSITGE-SEPVSRNGKFT------HENP-------- 305

Art.XP_023321169.1(Group II)      GGDKVPADLRIFESN-AVKV--DNSSLTGE-SIAVSLKPDLS------EKNR-------- 204
Art.D6WB95(Group II)              FGDRIPADIRIIHSQ-GFKV--DNSALTGE-SEPQFRGSECT------SDNI-------- 229
Art.XP_018565491.1(Group II)      FGDRIPADIRIIQSQ-GFKV--DNSALTGE-SEPQFRTIECS------SDNI-------- 228
Art.AFU25672.1(Group II)          FGDRIPADIRIIHSQ-GFKV--DNSALTGE-SEPQPRGTDCT------SDNL-------- 228
Art.KYN18319.1(Group II)          TGDRVPADIRILECQ-GLKV--DNASVTGE-SIPLIRTANIP-----QTGSV-------- 221
Art.EFN88446.1(Group II)          TGDRVSADIRILECQ-GLKV--DNASFTGESSIPLIRTANIP-----QTGSV-------- 221
Art.A0A087ZR23(Group II)          TGERVPADIRILECQ-GLKV--DHASITGE-SIPLLRTANII-----PTGDV-------- 226
Art.XP_012279085.1(Group II)      TGDRVPADIRLLECQ-GLKV--DNASITGE-SMPLLRSTKPS------PGSF-------- 223
Prt.BAA82752.2                    GGELVPCDVRIITCTDNCVV--DNASLTGE-AEPQKRKNEAT------HDEP-------- 215
Prt.OQR99879.1                    GGDKVPADIRVVDCSDDFLV--DNSCLTGE-SEPLKRVPHCT------DENP-------- 259
Prt.OQR92998.1                    GGDKVPADVRVVECSDDFMV--DNSCLTGE-SEPLKRVPHCT------DENP-------- 258
Prt.XP_008607481.1                GGDKVPADIRVVECSDDFLV--DNSCLTGE-SEPIKRVPHCT------DENP-------- 260
Prt.XP_008604114.1                GGDKVPADIRIIEGSDDLAV--DNSSLTGE-PEPLKRIPDCT------HDNP-------- 239
Prt.OQS04799.1                    GGDKVPADIRIVECSDDLAV--DNSSLTGE-PEPLKRIPDCT------HENP-------- 235
Prt.OQR94535.1                    GGDKVPADIRIVECSDDLAV--DNSSLTGE-PEPLKRIPDCT------HDNP-------- 235
Prt.CCA16430.1                    GGDKVPADIRIIECSDDLAV--DNSCLTGE-PEPLKRIPDCT------DENP-------- 240
Prt.POM62354.1                    GGDKVPADIRVLECSDDLTV--DNSCLTGE-PEPLKRIPDCT------DENP-------- 242
Prt.RAW38513.1                    GGDKVPADIRVLECSDDLTV--DNSCLTGE-PEPLKRVPDCT------DENP-------- 242
Prt.XP_024585310.1                GGDKVPADIRVLECSDDLTV--DNSCLTGE-PEPLKRVPDCT------DENP-------- 242
Prt.XP_647420.2                   AGDKVPADLRVIASH-HFKV--DNASLTGE-TEPQTRSPNCT------DENP-------- 332
Prt.XP_004352438.1                AGDRIPADIRIIYSH-HLKV--DNASLTGE-SEPQSRTAECT------DENA-------- 383
Prt.tr|D3BBA2|                    MGDKVPADVRVLFAH-HFKV--DNSSLTGE-SEPQTRTPECT------DQNA-------- 308
Prt.UPI00000795B1                 AGDKVPADIRIITCN-GMKV--DNSSLTGE-SDAQSCTVTCT------DDNP-------- 203
Prt.F1A2S2                        AGDKVPGDIRIITCN-GMKV--DNSSLTGE-SEAQSCTVTCT------DDNP-------- 203
Prt.PXF41383.1                    LGDKIPADIRLVSNQ-KLKV--DNSPLTGE-SEPIGRTVDCT------DDNP-------- 248
Prt.CAI99405.1                    LGDKIPADIRLVENA-KLKV--DNSSLTGE-SEPQKRTVECT------DENP-------- 255
Fun.SPPG_08470.2                  AGAKVPADMVLFNST-DCKV--DMSSLTGE-SDPLGRTALENG--APPDVEA-------- 304
Fun.UniRef100_A0A4P9WG54          AGSKISADLVLFAAA-DCQV--DMSSLTGE-AEPVRRVAVEKG--VGADVPA-------- 219
Fun.AMAG_01211.1                  SGDKVPADLWLFAAS-DLKV--DNSSLTGE-SEPQDRTP-KNT--Q---KAA-------- 212
Fun.AMAG_02439.1                  MGDKVPADVWLFKTT-DMKV--DNSSLTGE-AEPQERKP-IKT--H---DNP-------- 214
Fun.UniRef100_A0A1Y2HI79          MGDKTPADVRLFKTQ-DMKV--DNSSLTGE-SEPQDRVP-TNT--H---DTA-------- 210
Fun.UPI000006A49F                 MGDKMPADVFLFKTA-DMKV--DNASLTGE-SEPQDRSP-VNT--H---EAA-------- 212
Fun.AAF20202.1                    MGDKVPGDVFLFKTA-DMKV--DNASLTGE-SEPQDRSP-VNT--H---EAA-------- 211
Fun.estExt_Genewise1.C_180045     MGDKVPADLVLFAAT-EVKV--DNSSLTGE-SEPQERQLCPEG--DP--VRA-------- 298
Fun.PGTG_03133.2                  MGDKVPADLVLFSAS-DVKV--DNSSLTGE-SEPQERSVFAEG--DP--VRP-------- 288
Fun.CC1G_09151.2                  TGDKTPADMILFSAN-DLKV--DNSSLTGE-SEPQERRPLVEG--SP--HRA-------- 235
Fun.UniRef100_A0A067M739          MGDKTPADLIMFAAT-DLKV--DNSSLTGE-SEPQERGPVHEG--SK--GRA-------- 269
Fun.UniRef100_A0A5E3X872          VGDKTPADCVIISAT-DLKV--DNSSLTGE-SEPQERMAKPNG--MS--CRA-------- 294
Fun.fgenesh2_pm.C_sc              VGDKTPADLLLFAAT-DLKV--DNSSLTGE-SEPQERMPKIDG--SSY-PRA-------- 240
Fun.UniRef100_A0A4Y9ZEZ1          VGDKTPADLVLFAAT-DLKV--DNSSLTGE-SEPQERLPNLEG--SS--ARP-------- 290
Fun.UPI0001643CDB                 SGDKTPADLILFSAT-ELKV--DNSSLTGE-SEPQARIPLPNG--SK--QRP-------- 240
Fun.UniRef100_A0A067Q0Q4          TGDKTPADLILFGAT-DLRV--DNSNLTGE-SEPQERKPLPAG--SK--VRP-------- 283
Fun.e_gwh2.1.49.1                 VGDKTPADLVLFAAT-DLKV--DNSSLTGE-SEPQERCAVPDG--SK--ARP-------- 240
Fun.UniRef100_A0A4S4MS29          VGDKTPADLVLFAAT-DLKI--DNSSLTGE-SEPQERFPKPNG--HP--VRP-------- 289
Fun.UniRef100_A0A1M2VF35          SGDKTPADLIVFSAS-DLKI--DNSSLTGE-SEPQERFAKPDG--VQ--TRP-------- 136
Fun.UniRef100_J4GT94              TGDKTPADLVIFAAT-DMKV--DNSNLTGE-SEAQERISLPDG--SR--ARP-------- 288
```

```
Fun.UniRef100_A0A4Y9Y8I4           TGDKTPADLILFSAT-DLKV--DNSNLTGE-SEAQERFGKPEG--SS--HRP-------- 289
Fun.UniRef100_S8E4T6               TGDKTPADLIIFAAT-DLKV--DNSNLTGE-SEAQERFGVPEG--SK--HRP-------- 238
Fun.UniRef100_A0A1Y1VYW4           MGDKIPADCYVFSGS-DLKV--DNSSLTGE-SDPQERGA-GNK--N---NSP-------- 233
Fun.UniRef100_A0A2G5BEZ7           MGDKVPADCYIFAAN-ELKV--DNSSLTGE-SEPQERAA-GNS--M---RNP-------- 275
Fun.UniRef100_A0A507F1Y9           TGDKIPADLLVIGAT-ELKV--DNSSLTGE-ADPQERGK-KNS--H---TNP-------- 246
Fun.UniRef100_A0A1Y2BXZ1           SGDKIPADLLVIGST-ELKV--DNSSLTGE-ADPQERQK-RNT--F---ENP-------- 172
Fun.UniRef100_A0A1Y2BYC1           SGDKIPADLLVIGST-ELKV--DNSSLTGE-ADPQERSK-RNS--H---HNP-------- 241
Fun.UniRef100_A0A4P9WWS6           MGDKIPADLWIFQAT-DFKV--DNSSLTGE-TEPQERMR-KNV--H---TNP-------- 246
Fun.UniRef100_A0A507BSP0           MGDKVPADVILFAST-ELKV--DNSSLTGE-AEPQERNK-NNT--M---TSP-------- 256
Fun.BDEG_03368.1                   SGDKIPSDLRIFWSN-ELKV--DNSSLTGE-AEPQERNA-NNV--M---TSP-------- 225
Fun.BDEG_05936.1                   SGDKIPSDLRIFWSN-ELKV--DNSSLTGE-AEPQERNA-NNV--M---TSP-------- 229
Fun.UniRef100_A0A507E7U2           SGDKVPADLYVFAAN-EFKV--DNSSLTGE-ADPQERVP-RNT--Y---KSP-------- 274
Fun.SPPG_07476.2                   SGDKLPADLYIFSAN-ELKV--DNSSLTGE-ADPQERGP-RNR--Y---QNP-------- 194
Fun.UniRef100_A0A507EKQ2           SGDKLPADLYIFSAN-ELKV--DNSSLTGE-ADPQERVP-RNR--H---QNP-------- 316
Fun.UniRef100_A0A1X2ID33           MGDKVPADVYLFAG--EIKV--DNSSLTGE-SDPQVRGN-HNT--Q---KNP-------- 242
Fun.fgenesh1_pm.12_#_37            MGDKVPADLFIFAGS-ELKV--DNSSLTGE-SDPQERGP-INT--Q---QSL-------- 182
Fun.RO3G_04175.3                   MGDKVPADLFLFQGS-DLKV--DNSPLTGE-SDPQERGP-FNS--Q---QNV-------- 264
Fun.UniRef100_A0A0B7MNY4           MGDKIPADLLVFSAS-DLKV--DNSPLTGE-SDPQERGA-ENT--Q---KNV-------- 236
Fun.UniRef100_A0A0C9MZH6           MGDKVPADLFVFSAS-DLKV--DNSPLTGE-SDPQERGG-ANT--Q---KNV-------- 266
Fun.fgenesh1_pm.01_#_34            MGDKVPADLFIFSAS-DLKV--DNSPLTGE-SDPQERGG-TNT--Q---KNV-------- 175
Fun.UniRef100_S2JVU7               MGDKVPADLFIFSAS-DLKV--DNSPLTGE-SDPQERGA-TNT--Q---KNV-------- 266
Fun.UniRef100_A0A139AWN6           LGDKVPADCVIVAAT-DLKV--DNSSLTGE-SEPQERTK-RNT--N---ENP-------- 231
Fun.UniRef100_A0A2Z6QEU7           MGDKVPADLFMFATN-DMKV--DNSSLTGE-SEPQERSKAMST--E---KNP-------- 245

Fun.UniRef100_A0A2N0PA04           MGDKVPADLFVFSAT-DMKV--DNSSLTGE-SEPQERLKT-NT--Y---SNP-------- 245
Fun.UniRef100_A0A397VHQ3           MGDKIPADLLIFAAT-DLKV--DNSSLTGE-SEPQERNKK-NT--F---ENP-------- 256
Fun.SPPG_01615.2                   QGDKIPADIYVFHSS-ELKV--DNSSLTGE-SDPQERTA-HNT--H---KNP-------- 214
Fun.UniRef100_A0A194X4V3           MGDKTPADILVFSAS-DCKV--DNSSLTGE-SEPQERMK-DND--M---RNP-------- 248
Fun.UniRef100_A0A261Y8Y1           MGDKTPADIIVFASS-DCKV--DNSSLTGE-SEPQERGK-DND--M---KNP-------- 285
Fun.UniRef100_A0A0C3GKR9           MGDKTPADFLVFSAS-DCKV--DNSSLTGE-SEPQERTK-DND--M---RNP-------- 278
Fun.UniRef100_A0A2J6SAB3           MGDKTAADLLVFSAS-DCKV--DNSSLTGE-SEPQERTK-END--M---TNP-------- 300
Fun.UniRef100_A0A2J6SIQ3           MGDKTAADLLVFSAS-DCKV--DNSSLTGE-SEPQERTK-DND--M---MNP-------- 283
Fun.fgenesh2_pm.7_#_193            MGDKTPADVLVFWAS-DCKV--DNSSLTGE-SEPQERTR-END--M---QNP-------- 273
Fun.UniRef100_A0A090D7D5           MGDKTPADILVFSAS-DCKV--DNSSLTGE-SEPQDRTT-DND--M---KNP-------- 256
Fun.UniRef100_A0A447CBT6           MGDKTPADILVFSAS-DCKV--DNSSLTGE-SEPQDRTA-DND--M---KNP-------- 256
Fun.UniRef100_A0A507CSE4           IGDKVPADFYVFHGS-DFKV--DNSSLTGE-SEPQDRVA-SNT--Q---PNP-------- 284
Fun.UniRef100_A0A4P9Y2Y3           MGDKIPADLRIISAS-DMKV--DNSSLTGE-SDPQPRSSSVDT--Q---KNP-------- 232
Fun.UniRef100_A0A1Y2G5J7           MGDKIPADIMIIGCT-DMKV--DNSSLTGE-TDPQDRSP-IND--Q---KNP-------- 260
Fun.UniRef100_A0A4P9ZM46           QGDKVPADLYIFASN-ELKV--DNSSLTGE-SEPQSRFP-GNE--A---SNC-------- 255
Fun.UniRef100_A0A4P9Z1Y8           MGDKVPADLRIFASS-DLKV--DNSSLTGE-SEPQARTK-DND--H---KNP-------- 261
Fun.UniRef100_A0A137NQA6           MGDKVPADLFLFFVS-DMKV--DNSSLTGE-SEPQERIV-QND--Q---KNF-------- 210
Fun.UniRef100_A0A1Y1YKS1           MGDKIPADLLIFASS-DMKV--DNSSLTGE-SEPQERTK-ANT--H---KNP-------- 275
Fun.UniRef100_A0A1Y1YU47           MGDKIPADLLIFHSS-DMKV--DNSSLTGE-SEPQARVP-FND--Q---RSP-------- 271
Nem.KHN74191.1(Group I)            AGARIPADLRILQSN-GLKL--ETSAITGE-GQPIDYTHEAAA----PHISM-------- 225
Nem.NP_001122529.1(Group I)        AGYKIPADMRILQAN-CLMI--ESHDVTGH-RMPQEYKSDPVQ----ANVSV-------- 266
Nem.tr|O16436|(Group I)            SGCKVPADIRVIACT-DFYL--ETSSITGE-AEPLEFNSAMAD----QKTSI-------- 237
Nem.tr|O16331|(Group I)            SGCKVPADMRIIACT-DFFL--ETSSITGE-AEPLEFHSKTAD----AKTSI-------- 237
Nem.KHN88767.1(Group I)            SGSRVPADLRIIQST-GLKV--EASSITGE-AEPIEVHAESVA----EHIGV-------- 239
Nem.KHN88766.1(Group I)            CGARVPADVRILHSV-NLKI--ESSSVTGE-SEPIDFQSEAVA----AHIDV-------- 253
Prt.XP_024578788.1                 NGDKVPADIRILQCN-NLKV--ENSSLTGE-SELITLTSDVQD----HSVAH-------- 241
Prt.GAX12878.1                     DGEKVPADAVLLLCR-GLKA--ECASLTGE-SEPIPCSDQVSA----KGTRL-------- 236
Prt.GAX20661.1                     DGEKVPADAVLLLCR-ALKT--ECASLTGE-SEPISCSDQVSV----KGTQL-------- 235
Prt.PXF41326.1                     LGDKVPADVRLLTSN-RFTV--DNSSLTGE-SEPVELTPEV------SHSNP-------- 231
Prk.YP_001963725.1                 EGDIVPADCRIIESE-DVEV--DNSSLTGE-STSARRYKSENQIVLEGKFLW-------- 336
Prk.YP_324582.1                    EGDHVSADARLVKSE-SLYL--DVSVLTGE-SLPVARNAY-----------E-------- 181
Prk.NP_440621.1                    EGDRVSADLRLVSAD-SLYV--DVSVMTGE-SLPVARFAE------QDPVKA-------- 196
Prk.UPI0008639F5F                  EGDNVSADARLVEAF-QMKV--DTSTLTGE-SKPIRKVAEPIM---KDNESF-------- 185
Prk.NP_276630.1                    EGDTVPADARILESH-NLRV--DASALTGE-SKPVRKVSHPVR----EADNY-------- 189
Prk.YP_502111.1                    EGDHISADGRLVEAS-ELRI--DQSTLTGE-SHPVKKTADPSY---ETDISK-------- 192
Prk.tr|A0A347ZR85|                 EGEKISADCRLVDQA-ALRT--DQATLTGE-SRPVNKTAEAVL---QDKITY-------- 203
Prk.UPI00032DDB86                  SGDNIPADGRLFVCD-SLQV--NQSSLTGE-AVPVSKEDASQSQAAIQAAGR-------- 194
Prk.tr|A0A4R8A713|                 EGDKISADARLVETS-DFQA--NQSALTGE-SNPVHKQSDAVL---KDDLTR-------- 190
Prk.YP_391334.1                    EGEKIAADAILLSAN-DLYL--NLSVLNGE-STPSVRSLNP-----GDAQRE-------- 189
Prk.tr|W5W8S0|                     EGERVSADALLVEG--TVEV--DNSALTGE-SVPVVRSADP-A---AAQTRS-------- 173
Prk.tr|A0A1M7YB86|                 EGDKVPADLRLVESH-DLQV--DNSPLTGE-SMPCDLTSRE------SDTQA-------- 194
Prk.YP_357688.1                    EGDRIPADARLVECQ-DLVV--NNAPLTGE-AKPVALTAVV------EDARL-------- 193
Prk.tr|A0A1G6WYT0|                 EGDRVPADVRLVRVE-GLVV--NNAPLTGE-ANPLALTAAP------VDSPL-------- 193
Prk.tr|A0A1M6NFZ1|                 EGDRVPADARLVAAE-GLMA--NNAPLTGE-AQPVALSSAV------CESRL-------- 193
Prk.tr|A0A1H7UXF5|                 EGDRIPADARLIEGA-MLKV--NNASLTGE-SEASLRNSLP------VQGEL-------- 189
Prk.tr|B5YJF3|                     EGDKVPADARVIESN-SLTV--NNAPLTGE-SVPVVLTHES------ESGDL-------- 189
Prk.tr|A0A317MXL5|                 AGDRVPADCRLLEAH-GVRV--DTATISGE-SVPMARSAA--P---CASIHP-------- 192
Prk.tr|A0A1H1W1D1|                 EGDSVPADCRLLEAD-GLRL--SLATLTGE-SLPQSRTAE--A---SGSSDP-------- 190
```

```
Prk.UPI0002387025            EGARVPADCRIIEAW-GLRV--DLATLTGE-SFPKARNEK--A---DAGDDA-------- 195
Prk.tr|A0A497XH49|           EGAKVPADCRLIESW-GLRV--NLATLTGE-SRPKARSADAIS---DAIPDP-------- 197
Prk.UPI0000E10E8B            EGDKIPADGVILEMN-QLLV--DESILTGE-SEPLMKSAL---------DMV-------- 193
Prk.UPI000C2216DF            EGDRVTADARLIEQS-MLKV--DHSALTGE-SEPQLRSLKA------TSGNV-------- 210
Prk.UPI000C21D419            EGDRITADARLIEQS-MLKV--DHSALTGE-SEPQLRSLNA------TSGNV-------- 210
Prk.UPI000B509329            EGEQVPADARLFDVA-GLKV--DNASLTGE-SEPQLRTTYP------TDRHL-------- 188
Prk.tr|K0C7I7|               EGDKVSADGRLIRVN-QLQV--DMSSLTGE-STPETLQLEA------DSENP-------- 190
Prk.UPI00057321B9            EGDKVPADARLIEEN-TLKV--DHSSITGE-SEPQLRSLEC------THSNI-------- 190
Prk.UPI000615B8D6            EGDKVPADGRLIEVN-SLKV--DNSALTGE-AEPQLRSLNC------THPEM-------- 205
Prk.AKB42896.1               EGDKVPADGRLIETN-SLKV--DHSTITGE-AEPQLRSLEC------TNSNI-------- 201
Prk.UPI0006157E2A            EGDKVPADGRLIETN-VLKV--DNSAITGE-SEPQLRSLEC------THPNM-------- 205
Prk.UPI00061563E3            EGDKVPADGRLIETN-ALKV--DNSAITGE-SEPQLRSLEC------THPNM-------- 194
Prk.AKB83839.1               EGDKVPADGRLIETN-ALKV--DNSAITGE-SEPQLRSLEC------THPNM-------- 194
Prk.UPI00003C6559            EGDKVPADGRLVEIN-TLKV--DNSAITGE-SEPQLRSLEC------THPNM-------- 205
Prk.UPI0006157AA6            EGDKVPADGRLIEIN-TLKV--DNSAITGE-SEPQLRSLEC------THPNM-------- 205
Prk.UPI0006157148            EGDKVPADGRLIEIN-SLKV--DNSALTGE-SEPQLRSLEC------THSNL-------- 205
Prk.NP_633093.1              EGDKVPADGRLIEIN-SLKV--DNSALTGE-SEPQLRSLEC------THSNL-------- 205
Prk.UPI000615BB49            EGDKVPADGRLIETN-SLKV--DNSTLTGE-SEPQLRSLEC------THSNL-------- 205
Prk.UPI0000068661            EGDKVPADGRLIETN-ALKV--DNSALTGE-SEPQLRSIEC------THENM-------- 205
Prk.UPI0006154E5D            EGDKVPADGRLIETN-ALKV--DNSALTGE-SEPQLRSIEC------THEDM-------- 205
Prk.YP_565169.1              EGDKVPADGRLIEEN-TLKV--DNSSLTGE-AEPQLRSLEC------THPNI-------- 168

Prk.UPI0008DEB8E8             EGDKIPADGRLIEEN-SLKV--DNSPITGE-AEPQLRSLEC------THPNI------- 201
Prk.UPI000891AE6A             EGDKVPADGRLIEEN-SLKV--DNSPITGE-AEPQLRSLDC------THPNI------- 201
Prk.UPI00079C7F01             EGDKVPADARLIEQN-TLKV--DNSPITGE-AEPQLRALEC------THEDM------- 204
Prk.UPI00028B8ECD             EGDKIPADGRIIVQN-SLKV--DNSPITGE-SEPQLRSLEC------THDDM------- 201
Prt.XP_001427178.1            NGKRIPADIRILESN-EMKV--DNSSLTGE-SLLLMRSLEC-----TNPANP------- 262
Prt.XP_001346890.1            MGDKIPADVRIIQSR-EMKV--DNSALTGE-CDPLLRVTEL-----T-SENP------- 293
Prt.UniRef100_Q23EX6          SGDKVPADIRILTSN-EMKV--DNSPFTGE-TEPLLRTTEC-----SNL-NP------- 297
Prt.UniRef100_Q22XZ1          YGQRIPADIRILSSN-GMMV--DNYQLTGE-SEPQYRTVEC-----SHPESF------- 247
Prt.UniRef100_Q22LQ9          LGEKIPADIRILESN-EMKV--DNSPLTGE-CEPLLRTVEC-----SHPESY------- 295
Prt.UniRef100_Q245Y8          LGEKIPADIRILESN-EMKV--DNSPLTGE-SEPLLRTIEC-----SHPESY------- 305
Prt.UniRef100_Q23ZA6          IGEKIPADIRILESN-EMKV--DNSPLTGE-SEPLLRTTEC-----SHPESY------- 292
Prt.UniRef100_I7M7N1          AGEKIPADIRILESS-EMKV--DNSPLTGE-SEPQLRTAEC-----SHPENY------- 305
Prt.UniRef100_I7ME52          AGEKIPADIRILESS-EMKV--DNSALTGE-SEPQLRTVDC-----SHPENY------- 314
Prt.UniRef100_I7MH18          AGEKIPADMRIVESN-EMKV--DNSPLTGE-SEALLRTVEC-----THPENY------- 298
Prt.UniRef100_I7MD85          AGEKIPADIRIIESN-EMKV--DNSPLTGE-CEPLLRTVEC-----SHPDSY------- 293
Prt.UniRef100_Q23D88          AGEKIPADIRILMSN-EMKV--DNSPLTGE-SEPLLRTTEC-----THPENP------- 306
Prt.UniRef100_I7MHE1          AGERIPADIRIIQSN-EMKV--DNSPLTGE-SEPQLRTPIC-----THPDSP------- 300
Prt.UniRef100_I7M7R6          AGEKIPADIRMIQVN-EMKV--DNSALTGE-SEPQLRTTIC-----SHPESL------- 322
Prt.UniRef100_Q22P96          AGEKIPADIRLIQVN-EMKV--DNSALTGE-SEPQIRSTIC-----SHPESL------- 314
Prt.UniRef100_Q22PA2          AGEKIPADIRLIRVN-EMKV--DNSALTGE-SESQIRSTFC-----SHPESL------- 292
Prt.UPI00015F4774             EGDQVPADIRVIDSY-NLKV--DNASLTAC-ERSAVLIAPS-----RAKVPA------- 218
Prt.UPI000D26B24D             DGDQVPADIRILSCN-DMQV--DNSSLTGE-SEPQ----EG-----KLITVP------- 251
```
