# Supplementary material for: Bioinformatics approaches for classification and investigation of the evolution of the Na/K-ATPase alpha-subunit
Source: BMC Ecol Evol. 2022 Oct 26;22:122. doi: 10.1186/s12862-022-02071-0 (PMC9609216; doi:10.1186/s12862-022-02071-0)
Supplement: Supplementary file 1 — Additional file 1. Supplementary figures and tables. [file 12862_2022_2071_MOESM1_ESM.zip › Additional file 1 Fig. S8.pdf]

```
Ver.NP_0010804          GCQ-RQGAIVAVTG-----------------------------DGVNDSPALKKADIG--  733
Ver.F7E0B8              GCQ-RQGAIVAVTG-----------------------------DGVNDSPALKKADIG--  733
Ver.UPI000C736357       PLS-LQGAIVAVTG-----------------------------DGVNDSPALKKADIG--  687
Ver.UPI0003CD047A       GCQ-RQGAIVAVTG-----------------------------DGVNDSPALKKADIG--  736
Ver.UPI000C73EFA7       SLS-LQGAIVAVTG-----------------------------DGVNDSPALKKADIG--  738
Ver.UPI00049A9E19       GCQ-RQGAIVAVTG-----------------------------DGVNDSPALKKADIG--  740
Ver.XP_025028557.1      GCQ-RQGAIVAVTG-----------------------------DGVNDSPALKKADIG--  735
Ver.XP_020663591.1      GCQ-RQGAIVAVTG-----------------------------DGVNDSPALKKADIG--  796
Ver.UPI0000124FC2       GCQ-RQGAIVAVTG-----------------------------DGVNDSPALKKADIG--  718
Ver.UPI000BAD5294       GCQ-RQGAIVAVTG-----------------------------DGVNDSPALKKADIG--  717
Ver.XP_020948935.1      GCQ-RQGAIVAVTG-----------------------------DGVNDSPALKKADIG--  735
Ver.XP_012613923.1      GCQ-RQGAIVAVTG-----------------------------DGVNDSPALKKADIG--  721
Ver.UPI000C2D7C35       GCQ-RQGAIVAVTG-----------------------------DGVNDSPALKKADIG--  738
Ver.XP_006903931.1      GCQ-RQGAIVAVTG-----------------------------DGVNDSPALKKADIG--  691
Ver.XP_023380497.1      GCQ-RQGAIVAVTG-----------------------------DGVNDSPALKKADIG--  792
Ver.UPI0002B3612F       GCQ-RQGAIVAVTG-----------------------------DGVNDSPALKKADIG--  731
Ver.XP_020726792.1      GCQ-RQGAIVAVTG-----------------------------DGVNDSPALKKADIG--  686
Ver.ELK32312.1          GCQ-RQGAIVAVTG-----------------------------DGVNDSPALKKADIG--  760
Ver.UPI000226419C       GCQ-RQGAIVAVTG-----------------------------DGVNDSPALKKADIG--  736
Ver.UPI000C7286EF       GCQ-RQGAIVAVTG-----------------------------DGVNDSPALKKADIG--  721
Ver.UPI0007A6EC9C       GCQ-RQGAIVAVTG-----------------------------DGVNDSPALKKADIG--  721
Ver.UPI000C2DAA95       GCQ-RQGAIVAVTG-----------------------------DGVNDSPALKKADIG--  727
Ver.UPI000C740E55       GCQ-RQGAIVAVTG-----------------------------DGVNDSPALKKADIG--  732
Ver.UPI000CB4CAB6       GCQ-RQGAIVAVTG-----------------------------DGVNDSPALKKADIG--  732
Ver.PNJ19200.1          GCQ-RQGAIVAVTG-----------------------------DGVNDSPALKKADIG--  721
Ver.XP_021021704.1      GCQ-RQGAIVAVTG-----------------------------DGVNDSPALKKADIG--  734
Ver.UPI00035B05DE       GCQ-RQGAIVAVTG-----------------------------DGVNDSPALKKADIG--  756
Ver.XP_023600635.1      GCQ-RQGAIVAVTG-----------------------------DGVNDSPALKKADIG--  700
Ver.UPI000C732D5F       GCQ-RQGAIVAVTG-----------------------------DGVNDSPALKKADIG--  732
Ver.PNI95395.1          GCQ-RQGAIVAVTG-----------------------------DGVNDSPALKKADIG--  688
Ver.NP_036638.          GCQ-RQGAIVAVTG-----------------------------DGVNDSPALKKADIG--  721
Ver.XP_022441242.1      GCQ-RQGAIVAVTG-----------------------------DGVNDSPALKKADIG--  735
Ver.UPI000651771D       GCQ-RQGAIVAVTG-----------------------------DGVNDSPALKKADIG--  721
Ver.UPI000C7355ED       GCQ-RQGAIVAVTG-----------------------------DGVNDSPALKKADIG--  746
Ver.XP_021590883.1      GCQ-RQGAIVAVTG-----------------------------DGVNDSPALKKADIG--  758
Ver.XP_021106581.1      GCQ-RQGAIVAVTG-----------------------------DGVNDSPALKKADIG--  734
Ver.XP_024433413.1      GCQ-RQGAIVAVTG-----------------------------DGVNDSPALKKADIG--  721
Ver.UPI000C2EDFE7       GCQ-RQGAIVAVTG-----------------------------DGVNDSPALKKADIG--  732
Ver.UPI000C2E3154       GCQ-RQGAIVAVTG-----------------------------DGVNDSPALKKADIG--  734
Ver.XP_023507169.1      GCQ-RQGAIVAVTG-----------------------------DGVNDSPALKKADIG--  722
Ver.KFO33633.1          GCQ-RQGAIVAVTG-----------------------------DGVNDSPALKKADIG--  691
Ver.UPI000C71DF25       GCQ-RQGAIVAVTG-----------------------------DGVNDSPALKKADIG--  732
Ver.UPI0001914BDE       GCQ-RQGAIVAVTG-----------------------------DGVNDSPALKKADIG--  734
Ver.XP_003799510.1      GCQ-RQGAIVAVTG-----------------------------DGVNDSPALKKADIG--  734

Ver.XP_008065591.1      GCQ-RQGAIVAVTG-----------------------------DGVNDSPALKKADIG--  691
Ver.sp|P13637.3|        GCQ-RQGAIVAVTG-----------------------------DGVNDSPALKKADIG--  721
Ver.KPP65694.1          GCQ-RQGAIVAVTG-----------------------------DGVNDSPALKKADIG--  665
Ver.XP_023665796.1      GCQ-RQGAIVAVTG-----------------------------DGVNDSPALKKADIG--  730
Ver.XP_015461719.2      GCQ-RQGAIVAVTG-----------------------------DGVNDSPALKKADIG--  732
Ver.BAB60722.1          GCQ-RQGAIVAVTG-----------------------------DGVNDSPALKKADIG--  730
Ver.UPI000054C9F5       GCQ-RQGAIVAVTG-----------------------------DGVNDSPALKKADIG--  731
Ver.UPI0004E4D157       GCQ-RQGAIVAVTG-----------------------------DGVNDSPALKKADIG--  730
Ver.UPI000293B6B0       GCQ-RQGAIVAVTG-----------------------------DGVNDSPALKKADIG--  730
Ver.UPI0000E3AF2C       GCQ-RQGAIVAVTG-----------------------------DGVNDSPALKKADIG--  730
Ver.XP_024920682.1      GCQ-RQGAIVAVTG-----------------------------DGVNDSPALKKADIG--  731
Ver.UPI0003B0448B       GCQ-RQGAIVAVTG-----------------------------DGVNDSPALKKADIG--  730
Ver.UPI00016E235F       GCQ-RQGAIVAVTG-----------------------------DGVNDSPALKKADIG--  733
Ver.UPI00032B6FE9       GCQ-RQGAIVAVTG-----------------------------DGVNDSPALKKADIG--  731
Ver.XP_004074116.1      GCQ-RQGAIVAVTG-----------------------------DGVNDSPALKKADIG--  730
Ver.KKF19362.1          GCQ-RQGAIVAVTG-----------------------------DGVNDSPALKKADIG--  744
Ver.XP_012711044.2      GCQ-RQGAIVAVTG-----------------------------DGVNDSPALKKADIG--  730
Ver.UPI00025F91A4       GCQ-RQGAIVAVTG-----------------------------DGVNDSPALKKADIG--  730
Ver.XP_020793662.1      GCQ-RQGAIVAVTG-----------------------------DGVNDSPALKKADIG--  730
Ver.XP_024153267.1      GCQ-RQGAIVAVTG-----------------------------DGVNDSPALKKADIG--  730
Ver.XP_023117914.1      GCQ-RQGAIVAVTG-----------------------------DGVNDSPALKKADIG--  730
Ver.XP_022053465.1      GCQ-RQGAIVAVTG-----------------------------DGVNDSPALKKADIG--  730
Ver.NP_571759.2         GCQ-RQGAIVAVTG-----------------------------DGVNDSPALKKADIG--  731
Ver.W5UML4              GCQ-RQGAIVAVTG-----------------------------DGVNDSPALKKADIG--  732
Ver.W5L4G0              GCQ-RQGAIVAVTG-----------------------------DGVNDSPALKKADIG--  730
```

```
Ver.UPI0005D90DB9      GCQ-RQGAIVAVTG-----------------------------DGVNDSPALKKADIG--  730
Ver.XP_021427657.1     GCQ-RQGAIVAVTG-----------------------------DGVNDSPALKKADIG--  736
Ver.XP_024297426.1     GCQ-RQGAIVAVTG-----------------------------DGVNDSPALKKADIG--  741
Ver.UPI00001DFF47      GCQ-RLGAIVAVTG-----------------------------DGVNDSPALKKADIG--  719
Ver.UPI0006B7181A      GCQ-RLGAIVAVTG-----------------------------DGVNDSPALKKADIG--  732
Ver.UPI00079DB5F5      GCQ-RRGAIVAVTG-----------------------------DGVNDSPALKKADIG--  717
Ver.UPI000050D2B6      GCQ-RLGAIVAVTG-----------------------------DGVNDSPALKKADIG--  716
Ver.UPI0007F716B9      GCQ-RQGAIVAVTG-----------------------------DGVNDSPALKKADIG--  718
Ver.UPI0000E9CD46      GCQ-RQGAIVAVTG-----------------------------DGVNDSPALKMADIG--  729
Ver.UPI0007F7EA5B      GCQ-RQGAIVAVTG-----------------------------DGVNDSPALKKADIG--  730
Ver.UPI00079E2EF3      GCQ-RQGAIVAVTG-----------------------------DGVNDSPALKKADIG--  750
Ver.UPI0004448FEC      GCQ-RQGAIVAVTG-----------------------------DGVNDSPALKKADIG--  717
Ver.XP_023187147.1     GCQ-RQGAIVAVTG-----------------------------DGVNDSPALKKADIG--  730
Ver.UPI0006B30A18      GCQ-RQGAIVAVTG-----------------------------DGVNDSPALKKADIG--  732
Ver.XP_017270842.1     GCQ-RQGAIVAVTG-----------------------------DGVNDSPALKKADIG--  732
Ver.T2B507             GCQ-RQGAIVAVTG-----------------------------DGVNDSPALKKADIG--  718
Ver.XP_020466584.1     GCQ-RQGAIVAVTG-----------------------------DGVNDSPALKKADIG--  731
Ver.UPI0000124FC4      GCQ-RLGAIVAVTG-----------------------------DGVNDSPALKKADIG--  718
Ver.XP_004550929.1     GCQ-RLGAIVAVTG-----------------------------DGVNDSPALKKADIG--  731
Ver.UPI00025FB25F      GCQ-RLGAIVAVTG-----------------------------DGVNDSPALKKADIG--  718
Ver.UPI0000E3A2FA      GCQ-RLGAIVAVTG-----------------------------DGVNDSPALKKADIG--  731
Ver.UPI00003628C3      GCQ-RLGAIVAVTG-----------------------------DGVNDSPALKKADIG--  728
Ver.AGR87394.1         GCQ-RQGAIVAVTG-----------------------------DGVNDSPALKKADIG--  717
Ver.XP_020504733.1     GCQ-RLGAIVAVTG-----------------------------DGVNDSPALKKADIG--  733
Ver.AHB86586.1         GCQ-RLGAIVAVTG-----------------------------DGVNDSPALKKADIG--  718
Ver.UPI00032B9010      GCQ-RQGAIVAVTG-----------------------------DGVNDSPALKKADIG--  718
Ver.XP_022612296.1     GCQ-RQGAIVAVTG-----------------------------DGVNDSPALKKADIG--  718
Ver.XP_023285663.1     GCQ-RQGAIVAVTG-----------------------------DGVNDSPALKKADIG--  731
Ver.ELK38498.1         GCQ-RLGAIVAVTG-----------------------------DGVNDSPALKKADIG--  689
Ver.XP_545754.3        GCQ-RQGAVVAVTG-----------------------------DGVNDSPALKKADIG--  739
Ver.Q98SL3             GCQ-RQGAIVAVTG-----------------------------DGVNDSPALKRADIG--  717
Ver.NP_0011179         GCQ-RTGAIVAVTG-----------------------------DGVNDSPALKKADIG--  720
Ver.UPI0006B74D9B      GCQ-RTGAIVAVTG-----------------------------DGVNDSPALKKADIG--  719
Ver.XP_020796901.1     GCQ-RQGAIVAVTG-----------------------------DGVNDSPALKKADIG--  761
Ver.NP_571758.1        GCQ-RQGAIVAVTG-----------------------------DGVNDSPALKKADIG--  725
Ver.XP_005796664.1     GCQ-RQGAIVAVTG-----------------------------DGVNDSPALKKADIG--  718
Ver.UPI00000FE1CF      GCQ-RQGAIVAVTG-----------------------------DGVNDSPALKKADIG--  716
Ver.AHB86585.1         GCQ-RQGAIVAVTG-----------------------------DGVNDSPALKKADIG--  717
Ver.KKF24497.1         GCQ-RQGAIVAVTG-----------------------------DGVNDSPALKKADIG--  673
Ver.XP_020507674.1     GCQ-RQGAIVAVTG-----------------------------DGVNDSPALKKADIG--  573
Ver.XP_023135081.1     GCQ-RQGAIVAVTG-----------------------------DGVNDSPALKKADIG--  718
Ver.BAO02373.1         GCQ-RQGAIVAVTG-----------------------------DGVNDSPALKKADIG--  718
Ver.UPI0003D8328F      GCQ-RQGAIVAVTG-----------------------------DGVNDSPALKKADIG--  727
Ver.NP_0010831         GCQ-RQGAIVAVTG-----------------------------DGVNDSPALKKADIG--  728

Ver.UPI0000F6BCEB      GCQ-RQGAIVAVTG-----------------------------DGVNDSPALKKADIG--  728
Ver.ELK38499.1         GCQ-RQGAIVAVTG-----------------------------DGGNDSPALKKADIG--  728
Ver.EPQ02424.1         GCQ-RQGAIVAVTG-----------------------------DGVNDSPALKKADIG--  756
Ver.XP_023616468.1     ------------------------------------------------------------  655
Ver.XP_021054982.1     GCQ-RQGAIVAVTG-----------------------------DGVNDSPALKKADIG--  728
Ver.NP_036637.         GCQ-RQGAIVAVTG-----------------------------DGVNDSPALKKADIG--  728
Ver.XP_004858786.1     GCQ-RQGAIVAVTG-----------------------------DGVNDSPALKKADIG--  728
Ver.XP_003466610.1     GCQ-RQGAIVAVTG-----------------------------DGVNDSPALKKADIG--  728
Ver.XP_004639996.1     GCQ-RQGAIVAVTG-----------------------------DGVNDSPALKKADIG--  728
Ver.XP_005339432.1     GCQ-RQGAIVAVTG-----------------------------DGVNDSPALKKADIG--  728
Ver.XP_003795245.1     GCQ-RQGAIVAVTG-----------------------------DGVNDSPALKKADIG--  728
Ver.XP_008056914.2     GCQ-RQGAIVAVTG-----------------------------DGVNDSPALKKADIG--  728
Ver.XP_012604635.1     GCQ-RQGAIVAVTG-----------------------------DGVNDSPALKKADIG--  728
Ver.XP_004390257.1     GCQ-RQGAIVAVTG-----------------------------DGVNDSPALKKADIG--  728
Ver.XP_020024807.1     GCQ-RQGAIVAVTG-----------------------------DGVNDSPALKKADIG--  728
Ver.NP_0011253         GCQ-RQGAIVAVTG-----------------------------DGVNDSPALKKADIG--  728
Ver.XP_023069989.1     GCQ-RQGAIVAVTG-----------------------------DGVNDSPALKKADIG--  637
Ver.PNI19721.1         GCQ-RQGAIVAVTG-----------------------------DGVNDSPALKKADIG--  728
Ver.NP_000693.         GCQ-RQGAIVAVTG-----------------------------DGVNDSPALKKADIG--  728
Ver.NP_0012526         GCQ-RQGAIVAVTG-----------------------------DGVNDSPALKKADIG--  728
Ver.XP_021537506.1     GCQ-RQGAIVAVTG-----------------------------DGVNDSPALKKADIG--  789
Ver.UPI0002B2E326      GCQ-RQGAIVAVTG-----------------------------DGVNDSPALKKADIG--  731
Ver.XP_003415228.1     GCQ-RQGAIVAVTG-----------------------------DGVNDSPALKKADIG--  728
Ver.XP_004448489.1     GCQ-RQGAIVAVTG-----------------------------DGVNDSPALKKADIG--  728
Ver.XP_006922964.1     GCQ-RQGAIVAVTG-----------------------------DGVNDSPALKKADIG--  728
Ver.XP_545753.3        GCQ-RQGAIVAVTG-----------------------------DGVNDSPALKKADIG--  728
```

```
Ver.NP_0011650             GCQ-RQGAIVAVTG-----------------------------DGVNDSPALKKADIG--  728
Ver.NP_0010749             GCQ-RQGAIVAVTG-----------------------------DGVNDSPALKKADIG--  728
Ver.XP_020740887.1         GCQ-RQGAIVAVTG-----------------------------DGVNDSPALKKADIG--  728
Ver.XP_022415031.1         GCQ-RQGAIVAVTG-----------------------------DGVNDSPALKKADIG--  728
Ver.XP_007129684.1         GCQ-RQGAIVAVTG-----------------------------DGVNDSPALKKADIG--  728
Ver.UPI000226F4AA          GCQ-RQGAIVAVTG-----------------------------DGVNDSPALKKADIG--  733
Ver.UPI00005E9366          GCQ-RQGAIVAVTG-----------------------------DGVNDSPALKKADIG--  728
Ver.XP_020835237.1         GCQ-RQGAIVAVTG-----------------------------DGVNDSPALKKADIG--  728
Ver.XP_020653823.1         GCQ-RQGAIVAVTG-----------------------------DGVNDSPALKKADIG--  728
Ver.XP_026576074.1         GCQ-RQGAIVAVTG-----------------------------DGVNDSPALKKADIG--  728
Ver.UPI0000124FC0          GCQ-RQGAIVAVTG-----------------------------DGVNDSPALKKADIG--  725
Ver.XP_005293820.1         GCQ-RQGAIVAVTG-----------------------------DGVNDSPALKKADIG--  728
Ver.KYO43368.1             GCQ-RQGAIVAVTG-----------------------------DGVNDSPALKKADIG--  734
Ver.XP_006038189.1         GCQ-RQGAIVAVTG-----------------------------DGVNDSPALKKADIG--  728
Ver.XP_023390675.1         GCQ-RQVRGR----------------------------------QAGCLQALLPSGTG--  695
Ver.ELK30843.1             GCQ-RQGAIVAVTG-----------------------------DGVNDSPALKKADIG--  751
Ver.EMP33651.1             GCQ-RQGAIVAVTG-----------------------------DGVNDSPALKKADIG--  611
Ver.UPI000056D0DB          GCQ-RQGAIVAVTG-----------------------------DGVNDSPALKKADIG--  736
Ver.AHD24596.1             GCQ-RRGAIVAVTG-----------------------------DGVNDSPALKKADIG--  731
Ver.UPI00001261C4          GCQ-RQGAIVAVTG-----------------------------DGVNDSPALKKADIG--  730
Ver.UPI000C2F2801          GCQ-RQGAIVAVTG-----------------------------DGVNDSPALKKADIG--  719
Ver.NP_571763.1            GCQ-RQGAIVAVTG-----------------------------DGVNDSPALKKADIG--  732
Ver.UPI00025FADDE          GCQ-RQGAIVAVTG-----------------------------DGVNDSPALKKADIG--  731
Ver.UPI0000318264          GCQ-RQGAIVAVTG-----------------------------DGVNDSPALKKADIG--  733
Ver.UPI0006B827F3          GCQ-RQGAIVAVTG-----------------------------DGVNDSPALKKADIG--  766
Ver.XP_004066575.1         GCQ-RQGAIVAVTG-----------------------------DGVNDSPALKKADIG--  730
Ver.XP_024144685.1         GCQ-RQGAIVAVTG-----------------------------DGVNDSPALKKADIG--  730
Ver.UPI0006B31231          GCQ-RQGAIVAVTG-----------------------------DGVNDSPALKKADIG--  730
Ver.UPI0006B37238          GCQ-RQGAIVAVTG-----------------------------DGVNDSPALKKADIG--  733
Ver.ADD60471.1             GCQ-RQGAIVAVTG-----------------------------DGVNDSPALKKADIG--  730
Ver.UPI000157ACC7          GCQ-RQGAIVAVTG-----------------------------DGVNDSPALKKADIG--  733
Ver.AAT48993.1             GCQ-RQGAIVAVTG-----------------------------DGVNDSPALKKADIG--  731
Ver.XP_020476182.1         GCQ-RQGAIVAVTG-----------------------------DGVNDSPALKKADIG--  734
Ver.UPI00079E81D7          GCQ-RQGAIVAVTG-----------------------------DGVNDSPALKKADIG--  731
Ver.UPI000274DF5C          GCQ-RQGAIVAVTG-----------------------------DGVNDPPALKKADIG--  724
Ver.NP_0012969             GCQ-RQGAIVAVTG-----------------------------DGVNDSPALKKADIG--  731
Ver.UPI000443A733          GCQ-RQGAIVAVTG-----------------------------DGVNDSPALKKADIG--  731
Ver.XP_023185631.1         GCQ-RQGAIVAVTG-----------------------------DGVNDSPALKKADIG--  731
Ver.ALA65287.2             GCQ-RQGAIVAVTG-----------------------------DGVNDSPALKKADIG--  730
Ver.AKQ12834.1             GCQ-RQGAIVAVTG-----------------------------DGVNDSPALKKADIG--  733
Ver.XP_023275950.1         GCQ-RQGAIVAVTG-----------------------------DGVNDSPALKKADIG--  732
Ver.ADB03120.1             GCQ-RQGAIVAVTG-----------------------------DGVNDSPALKKADIG--  731

Ver.UPI00022B0848          GCQ-RQGAIVAVTG-----------------------------DGVNDSPALKKADIG--  731
Ver.AGZ87948.1             GCQ-RQGAIVAVTG-----------------------------DGVNDSPALKKADIG--  731
Ver.sp|Q9YH26.2|           GCQ-RQGAIVAVTG-----------------------------DGVNDSPALKKADIG--  731
Ver.AGO02179.1             GCQ-RQGAIVAVTG-----------------------------DGVNDSPALKKADIG--  731
Ver.AHB86584.1             GCQ-RQGAIVAVTG-----------------------------DGVNDSPALKKADIG--  731
Ver.XP_023121557.1         GCQ-RQGAIVAVTG-----------------------------DGVNDSPALKKADIG--  731
Ver.XP_022078036.1         GCQ-RQGAIVAVTG-----------------------------DGVNDSPALKKADIG--  731
Ver.XP_020792263.1         GCQ-RQGAIVAVTG-----------------------------DGVNDSPALKKADIG--  732
Ver.AGR87393.1             GCQ-RQGAIVAVTG-----------------------------DGVNDSPALKKADIG--  732
Ver.ABF58911.1             GCQ-RQGAIVAVTG-----------------------------DGVNDSPALKKADIG--  732
Ver.Q90X33                 GCQ-RQGAIVAVTG-----------------------------DGVNDSPALKKADIG--  732
Ver.XP_022530668.1         GCQ-RQGAIVAVTG-----------------------------DGVNDSPALKKADIG--  732
Ver.AJR20271.1             GCQ-RQGAIVAVTG-----------------------------DGVNDSPALKKADIG--  748
Ver.XP_020328431.1         GCQ-RQGAIVAVTG-----------------------------DGVNDSPALKKADIG--  738
Ver.XP_021426673.1         GCQ-RQGAIVAVTG-----------------------------DGVNDSPALKKADIG--  733
Ver.XP_023653512.1         GCQ-RQGAIVAVTG-----------------------------DGVNDSPALKKADIG--  732
Ver.sp|Q92030.1|           GCQ-RQGAIVAVTG-----------------------------DGVNDSPALKKADIG--  730
Ver.ALB35496.1             GCQ-RQGAIVAVTG-----------------------------DGVNDSPALKKADIG--  730
Ver.NP_571761.1            GCQ-RQGAIVAVTG-----------------------------DGVNDSPALKKADIG--  736
Ver.AJR20270.1             GCQ-RQGAIVAVTG-----------------------------DGVNDSPALKKADIG--  734
Ver.XP_023690671.1         GCQ-RQGAIVAVTG-----------------------------DGVNDSPALKKADIG--  732
Ver.XP_008322794.1         GCQ-RQGAIVAVTG-----------------------------DGVNDSPALKKADIG--  764
Ver.XP_017282368.1         GCQ-RQGAIVAVTG-----------------------------DGVNDSPALKKADIG--  737
Ver.XP_004066573.1         GCQ-RQGAIVAVTG-----------------------------DGVNDSPALKKADIG--  733
Ver.XP_024144684.1         GCQ-RQGAIVAVTG-----------------------------DGVNDSPALKKADIG--  733
Ver.XP_020497843.1         GCQ-RQGAIVAVTG-----------------------------DGVNDSPALKKADIG--  745
Ver.UPI000027C768          GCQ-RQGAIVAVTG-----------------------------DGVNDSPALKKADIG--  727
Ver.XP_004571307.1         GCQ-RQGAIVAVTG-----------------------------DGVNDSPALKKADIG--  732
Ver.XP_012714443.1         GCQ-RRGAIVAVTG-----------------------------DGVNDSPALKKADIG--  733
```

```
Ver.UPI00066EFDEA       GCQ-RQGAIVAVTG---------------------------DGVNDSPALKKADIG--  737
Ver.XP_022617258.1      GCQ-RQGAIVAVTG---------------------------DGVNDSPALKKADIG--  733
Ver.BAN17691.1          GCQ-RQGAIVAVTG---------------------------DGVNDSPALKKADIG--  733
Ver.sp|P25489.1|        GCQ-RTGAIVAVTG---------------------------DGVNDSPALKKADIG--  735
Ver.XP_022536277.1      GCQ-RQGAIVAVTG---------------------------DGVNDSPALKKADIG--  733
Ver.Q9DEU1              GCQ-RQGAIVAVTG---------------------------DGVNDSPALKKADIG--  733
Ver.AGR45921.1          GCQ-RQGAIVAVTG---------------------------DGVNDSPALKKADIG--  732
Ver.sp|P30714.2|        GCQ-RQGAIVAVTG---------------------------DGVNDSPALKKADIG--  731
Ver.NP_0010840          GCQ-RQGAIVAVTG---------------------------DGVNDSPALKKADIG--  733
Ver.NP_989407.1         GCQ-RQGAIVAVTG---------------------------DGVNDSPALKKADIG--  731
Ver.XP_004853865.1      GCQ-RQGAIVAVTG---------------------------DGVNDSPALKKADIG--  732
Ver.ACB20771.2          GCQ-RQGAIVAVTG---------------------------DGVNDSPALKKADIG--  732
Ver.XP_023557491.1      GCQ-RQGAIVAVTG---------------------------DGVNDSPALKKADIG--  701
Ver.ERE90024.1          GCQ-RQGAIVAVTG---------------------------DGVNDSPALKKADIG--  751
Ver.XP_005076578.1      GCQ-RQGAIVAVTG---------------------------DGVNDSPALKKADIG--  731
Ver.AAA41671.1          GCQ-RQGAIVAVTG---------------------------DGVNDSPALKKADIG--  731
Ver.XP_021504168.1      GCQ-RQGAIVAVTG---------------------------DGVNDSPALKKADIG--  731
Ver.XP_021051287.1      GCQ-RQGAIVAVTG---------------------------DGVNDSPALKKADIG--  731
Ver.XP_021013125.1      GCQ-RQGAIVAVTG---------------------------DGVNDSPALKKADIG--  731
Ver.NP_659149.          GCQ-RQGAIVAVTG---------------------------DGVNDSPALKKADIG--  731
Ver.EPQ03777.1          GCQ-RQGAIVAVTG---------------------------DGVNDSPALKKADIG--  760
Ver.XP_020858281.1      GCQ-RQGAIVAVTG---------------------------DGVNDSPALKKADIG--  729
Ver.XP_004380410.1      GCQ-RQGAIVAVTG---------------------------DGVNDSPALKKADIG--  729
Ver.AGY54951.1          GCQ-RQGAIVAVTG---------------------------DGVNDSPALKKADIG--  729
Ver.UPI0001FB338F       GCQ-RQGAIVAVTG---------------------------DGVNDSPALKKADIG--  729
Ver.NP_0011565          GCQ-RQGAIVAVTG---------------------------DGVNDSPALKKADIG--  731
Ver.XP_010587900.1      GCQ-RQGAIVAVTG---------------------------DGVNDSPALKKADIG--  700
Ver.UPI0002B3D77C       GCQ-RQGAIVAVTG---------------------------DGVNDSPALKKADIG--  744
Ver.NP_0010702          GCQ-RQGAIVAVTG---------------------------DGVNDSPALKKADIG--  729
Ver.OWK04910.1          GCQ-RQGAIVAVTG---------------------------DGVNDSPALKKADIG--  700
Ver.UPI0000124FBE       GCQ-RQGAIVAVTG---------------------------DGVNDSPALKKADIG--  729
Ver.NP_0010093          GCQ-RQGAIVAVTG---------------------------DGVNDSPALKKADIG--  729
Ver.XP_020747989.1      GCQ-RQGAIVAVTG---------------------------DGVNDSPALKKADIG--  729
Ver.XP_020012504.1      GCQ-RQGAIVAVTG---------------------------DGVNDSPALKKADIG--  731
Ver.XP_005334975.1      GCQ-RQGAIVAVTG---------------------------DGVNDSPALKKADIG--  731
Ver.NP_0010033          GCQ-RQGAIVAVTG---------------------------DGVNDSPALKKADIG--  729
Ver.XP_022352592.1      GCQ-RQGAIVAVTG---------------------------DGVNDSPALKKADIG--  759
Ver.XP_011283388.1      GCQ-RQGAIVAVTG---------------------------DGVNDSPALKKADIG--  729
Ver.XP_024426171.1      GCQ-RQGAIVAVTG---------------------------DGVNDSPALKKADIG--  729

Ver.XP_023975434.1      GCQ-RQGAIVAVTG---------------------------DGVNDSPALKKADIG--  729
Ver.XP_022439684.1      GCQ-RQGAIVAVTG---------------------------DGVNDSPALKKADIG--  729
Ver.XP_024620662.1      GCQ-RQGAIVAVTG---------------------------DGVNDSPALKKADIG--  729
Ver.XP_006919736.1      GCQ-RQGAIVAVTG---------------------------DGVNDSPALKKADIG--  700
Ver.UPI000C746E0C       GCQ-RQGAIVAVTG---------------------------DGVNDSPALKKADIG--  742
Ver.UPI000C2E744C       GCQ-RQGAIVAVTG---------------------------DGVNDSPALKKADIG--  735
Ver.XP_020944376.1      GCQ-RQGAIVAVTG---------------------------DGVNDSPALKKADIG--  729
Ver.XP_008071711.2      GCQ-RQGAIVAVTG---------------------------DGVNDSPALKKADIG--  736
Ver.XP_012663099.1      GCQ-RQGAIVAVTG---------------------------DGVNDSPALKKADIG--  731
Ver.UPI000C2E4C26       GCQ-RQGAIVAVTG---------------------------DGVNDSPALKKADIG--  727
Ver.XP_012617266.1      GCQ-RQGAIVAVTG---------------------------DGVNDSPALKKADIG--  731
Ver.XP_012314296.1      GCQ-RQGAIVAVTG---------------------------DGVNDSPALKKADIG--  731
Ver.UPI0001C9F9BA       GCQ-RQGAIVAVTG---------------------------DGVNDSPALKKADIG--  731
Ver.NP_000692.          GCQ-RQGAIVAVTG---------------------------DGVNDSPALKKADIG--  731
Ver.XP_008971666.1      GCQ-RQGAIVAVTG---------------------------DGVNDSPALKKADIG--  700
Ver.XP_016780478.1      GCQ-RQGAIVAVTG---------------------------DGVNDSPALKKADIG--  731
Ver.XP_023078532.1      GCQ-RQGAIVAVTG---------------------------DGVNDSPALKKADIG--  731
Ver.NP_0012536          GCQ-RQGAIVAVTG---------------------------DGVNDSPALKKADIG--  731
Ver.PNJ46178.1          GCQ-RQGAIVAVTG---------------------------DGVNDSPALKKADIG--  731
Ver.ETE67008.1          GCQ-RQGAIVAVTG---------------------------DGVNDSPALKKADIG--  730
Ver.XP_007435355.1      GCQ-RQGAIVAVTG---------------------------DGVNDSPALKKADIG--  700
Ver.XP_020645227.1      GCQ-RQGAIVAVTG---------------------------DGVNDSPALKKADIG--  700
Ver.XP_006132947.1      GCQ-RQGAIVAVTG---------------------------DGVNDSPALKKADIG--  700
Ver.XP_005292736.1      GCQ-RQGAIVAVTG---------------------------DGVNDSPALKKADIG--  732
Ver.XP_024064252.1      GCQ-RQGAIVAVTG---------------------------DGVNDSPALKKADIG--  732
Ver.XP_025067531.1      GCQ-RQGAIVAVTG---------------------------DGVNDSPALKKADIG--  700
Ver.NP_990852.          GCQ-RQGAIVAVTG---------------------------DGVNDSPALKKADIG--  729
Ver.XP_021253236.1      GCQ-RQGAIVAVTG---------------------------DGVNDSPALKKADIG--  729
Ver.UPI00051ECCC0       GCQ-RQGAIVAVTG---------------------------DGVNDSPALKKADIG--  724
Ver.KFU90062.1          GCQ-RQGAIVAVTG---------------------------DGVNDSPALKKADIG--  726
```

```
Ver.KFP84941.1          GCQ-RQGAIVAVTG-----------------------------DGVNDSPALKKADIG--  726
Ver.XP_030327328.1      GCQ-RQGAIVAVTG-----------------------------DGVNDSPALKKADIG--  729
Ver.XP_023796730.1      GCQ-RQGAIVAVTG-----------------------------DGVNDSPALKKADIG--  700
Ver.XP_021404823.1      GCQ-RQGAIVAVTG-----------------------------DGVNDSPALKKADIG--  729
Ver.XP_025966334.1      GCQ-RQGAIVAVTG-----------------------------DGVNDSPALKKADIG--  729
Ver.UPI0004FDA0CB       GCQ-RQGAIVAVTG-----------------------------DGVNDSPALKKADIG--  727
Ver.KQK85052.1          GCQ-RQGAIVAVTG-----------------------------DGVNDSPALKKADIG--  738
Ver.NP_0012973          GCQ-RQGAIVAVTG-----------------------------DGVNDSPALKKADIG--  731
Ver.KFW61640.1          GCQ-RQGAIVAVTG-----------------------------DGVNDSPALKKADIG--  726
Ver.XP_005511501.1      GCQ-RQGAIVAVTG-----------------------------DGVNDSPALKKADIG--  700
Ver.OPJ66608.1          GCQ-RQGAIVAVTG-----------------------------DGVNDSPALKKADIG--  732
Art.EFX69525.1          GFQ-RLGSIVAVTG-----------------------------DGVNDSPALKKADIG--  719
Rinv.CRX73232.1-        GCQ-RQGAIVAVTG-----------------------------DGVNDSPALKKADIG--  704
Prt.XP_001742517.1      GCQ-RANQIVAVTG-----------------------------DGVNDSPALKKADIG--  750
Prt.EGD73524.1          GCQ-RAGQIVAVTG-----------------------------DGVNDSPALKKADIG--  848
Rinv.PIS80793.1         GFQ-RQGRFVAVTG-----------------------------DGVNDSPALKKADIG--  739
Ver.ACB20770.2          GCH-SQGAMEAVTG-----------------------------DGVNDSAIFKKADIG--  674
Art.AAF17586.1          ACQ-RRGEIVAVSG-----------------------------DGVNDSPALKRADIG--  717
Art.AFU25666.1          GCQ-NLGAIVAVTG-----------------------------DGVNDSPALKKADIG--  713
Art.AFU25665.1          GCQ-RIGGVVAVTG-----------------------------DGVNDSPALKKADIG--  759
Art.XP_021915175.1      GCQ-RMGAIVAVTG-----------------------------DGVNDSPALKKADIG--  643
Art.EFN85240.1          GCQ-RMGAIVAVTG-----------------------------DGVNDSPALKKADIG--  689
Art.OXA63786.1          ALQ-KQGAIVAVTG-----------------------------DGVNDSPALKKADIG--  684
Art.ODN01960.1          ALQ-RQNEIVAVTG-----------------------------DGVNDSPALKKADIG--  713
Rinv.XP_020602016.1     GCQ-RQGAIVAVTG-----------------------------DGVNDSPALKKADIG--  545
Rinv.G4VGA0             GCQ-RQGAIVAVTG-----------------------------DGVNDSPALKQADIA--  713
Art.ODM98837.1          GCQ-RNGAVVAVTG-----------------------------DGVNDSPALKKADIG--  708
Art.AGZ13696.1-         GCQ-RAGAIVAVTG-----------------------------DGVNDSPALKRADIG--  733
Rinv.KXJ20388.1         GCQ-RNGAIVAVTG-----------------------------DGVNDSPALKKADIG--  740
Nem.CDW54807.1          SCQ-RNKNIVAVTG-----------------------------DGVNDSPALKQADIG--  686
Nem.KRZ50957.1          SCQ-RNGAIVAVTG-----------------------------DGVNDSPALKQADIG--  801
Nem.KRX38989.1          SCQ-RNGAIVAVTG-----------------------------DGVNDSPALKQADIG--  801
Nem.KRY10799.1          SCQ-RNGAIVAVTG-----------------------------DGVNDSPALKQADIG--  830
Nem.KRY70033.1          SCQ-RNGAIVAVTG-----------------------------DGVNDSPALKQADIG--  739
Nem.KRZ74907.1          SCQ-RNGAIVAVTG-----------------------------DGVNDSPALKQADIG--  739
Nem.KRZ04306.1          SCQ-RNGAIVAVTG-----------------------------DGVNDSPALKQADIG--  739
Nem.KRX24043.1          SCQ-RNGAIVAVTG-----------------------------DGVNDSPALKQADIG--  800
Nem.KRY48181.1          SCQ-RNGAIVAVTG-----------------------------DGVNDSPALKQADIG--  739

Nem.KRY33278.1          SCQ-RNGAIVAVTG-----------------------------DGVNDSPALKQADIG--  772
Rinv.OQV18895.1         ACQ-RQGAIVAVTG-----------------------------DGVNDSPALKKADIG--  725
Nem.XP_003369418.1      GCQ-RQGAIVAVTG-----------------------------DGVNDSPALKKADIG--  780
Nem.UPI0007A1BF54       GFQ-RQGKIVAVTG-----------------------------DGVNDSPALKRADIG--  742
Nem.UPI000183E9C3       GFQ-KQGQIVAVTG-----------------------------DGVNDSPALKKADIG--  744
Nem.UPI0006101D56       GFQ-KQGQIVAVTG-----------------------------DGVNDSPALKKADIG--  676
Nem.UPI000601D02E       AFQ-HEGYIVAVTG-----------------------------DGVNDSPALKKADIG--  705
Nem.UPI00060CD0E5       AFQ-HEGYIVAVTG-----------------------------DGVNDSPALKKADIG--  705
Nem.UPI00060630B9       AFQ-KQGQIVAVTG-----------------------------DGVNDSPALKKADIG--  762
Nem.UPI00060EAC2D       GFQ-RQGQIVAVTG-----------------------------DGVNDSPALKKADIG--  704
Nem.UPI000609E432       GFQ-RQGQIVAVTG-----------------------------DGVNDSPALKKADIG--  713
Nem.UPI000609F1C5       GFQ-RQGQIVAVTG-----------------------------DGVNDSPALKKADIG--  701
Ver.NP_835200.1         GCQ-RQGAIVAVTG-----------------------------DGVNDSPALKKADIG--  731
Ver.NP_571762.1         GCQ-RQGAIVAVTG-----------------------------DGVNDSPALKKADIG--  731
Ver.BAJ13363.1          GCQ-RQGAIVAVTG-----------------------------EGVNDSPALRKADIG--  736
Ver.UPI00001DFF4A       GCQ-RQGAIVAVTG-----------------------------DGVNDSPALRKADIG--  736
Ver.UPI00002BAA33       GCQ-RQGAIVAVTG-----------------------------DGVNDSPALKKADIG--  749
Ver.XP_021506251.1      GCQ-RLGAIVAVTG-----------------------------DGVNDSPALKKADIG--  737
Ver.NP_074039.          GCQ-RLGAIVAVTG-----------------------------DGVNDSPALKKADIG--  736
Ver.XP_021014708.1      GCQ-RLGAIVAVTG-----------------------------DGVNDSPALKKADIG--  740
Ver.NP_038762.          GCQ-RLGAIVAVTG-----------------------------DGVNDSPALKKADIG--  740
Ver.XP_020024800.1      GCQ-RLGAIVAVTG-----------------------------DGVNDSPALKKADIG--  737
Ver.XP_003795244.1      GCQ-RLGAIVAVTG-----------------------------DGVNDSPALKKADIG--  739
Ver.XP_004639995.1      GCQ-NLGAIVAVTG-----------------------------DGVNDSPALKKADIG--  743
Ver.XP_004448484.1      GCQ-RQGAIVAVTG-----------------------------DGVNDSPALKKADIG--  737
Ver.XP_023103614.1      GCQ-RQGAIVAVTG-----------------------------DGVNDSPALKKADIG--  740
Ver.XP_023496657.1      GCQ-RQGNVVAATG-----------------------------DGVNDSPALKKADIG--  744
Ver.XP_021537588.1      GCQ-RQGAIVAVTG-----------------------------DGVNDSPALRKADIG--  738
Ver.XP_006096963.1      GCQ-RLGAIVAVTG-----------------------------DGVNDSPALKKADIG--  739
Ver.XP_006922963.1      GCQ-RLGAIVAVTG-----------------------------DGVNDSPALKKADIG--  738
Ver.XP_011371380.1      GCQ-RLGAIVAVTG-----------------------------DGVNDSPALKKADIG--  738
Ver.NP_0011375          GCQ-RLGAIVAVTG-----------------------------DGVNDSPALKKADIG--  738
Ver.XP_020740848.1      GCQ-RLGAIVAVTG-----------------------------DGVNDSPALKKADIG--  738
```

```
Ver.XP_021568356.1        GCQ-RLGAIVAVTG-----------------------------DGVNDSPALKKADIG--  737
Ver.XP_012604632.1        GCQ-RLGAIVAVTG-----------------------------DGVNDSPALKKADIG--  736
Ver.NP_653300.            GCQ-RLGAVVAVTG-----------------------------DGVNDSPALKKADIG--  737
Ver.XP_003892961.1        GCQ-RLGAIVAVTG-----------------------------DGVNDSPALKKADIG--  737
Ver.XP_023069991.1        GCQ-RLGAIVAVTG-----------------------------DGVNDSPALKKADIG--  737
Ver.UPI0001C650F7         GCQ-RLGAIVAVTG-----------------------------DGVNDSPALKKADIG--  735
Ver.XP_013220247.1        GCQ-RLGAIVAVTG-----------------------------DGVNDSPALKKADIG--  695
Art.ODM98254.1            GCQ-RQGAIVAVTG-----------------------------DGVNDSPALKKADIGLV  696
Rinv.NP_0012967           GCQ-RQGAIVAVTG-----------------------------DGVNDSPALKKADIG--  739
Rinv.OQV17561.1           GCQ-RQGAIVAVTG-----------------------------DGVNDSPALKKADIG--  779
Art.EFX71103.1            GCQ-RAGAIVAVTG-----------------------------DGVNDSPALKKADIGKE  713
Art.EFX71104.1            GCQ-RAGAIVAVTG-----------------------------DGVNDSPALKKADIG--  710
Art.EFX71105.1            GCQ-RAGAIVAVTG-----------------------------DGVNDSPALKKADIG--  724
Art.XP_023221169.1        SCQ-RLGAIVAVTG-----------------------------DGVNDSPALKKADIG--  632
Art.XP_022254094.1        GCQ-RLGAIVAVTG-----------------------------DGVNDSPALKKADIG--  768
Art.XP_023347331.1        GCQ-RLGAIVAVTG-----------------------------DGVNDSPALKKADIG--  712
Rinv.ALJ53300.1           GCQ-RQGAIVAVTG-----------------------------DGVNDSPALKKADIG--  730
Rinv.BAA32798.1           GCQ-RQGAIVAVTG-----------------------------DGVNDSPALKKADIG--  729
Rinv.XP_018651572.1       GCQ-RQGAIVAVTG-----------------------------DGVNDSPALKQADIG--  724
Rinv.AAL09322.1           GCQ-RQGAIVAVTG-----------------------------DGVNDSPALKQADIG--  714
Rinv.sp|Q6RWA9.1|         GIQ-RMGAIVAVTG-----------------------------DGVNDSPALKKADIG--  722
Rinv.CDS22215.1           GFQ-RMGAIVAVTG-----------------------------DGVNDSPALKKADIG--  733
Rinv.CDS36343.1           GFQ-RMGAIVAVTG-----------------------------DGVNDSPALKKADIG--  733
Rinv.AAX09623.1           GCQ-RQGAIVAVTG-----------------------------DGVNDSPALKKADIG--  730
Rinv.ABO61333.1           GCQ-RQGHIVAVTG-----------------------------DGVNDSPALKKADIG--  736
Rinv.ABO61332.1           GCQ-RQGHIVAVTG-----------------------------DGVNDSPALKKADIG--  736
Rinv.EKC34610.1           GCQ-RQGAIVAVTG-----------------------------DGVNDSPALKKADIG--  758
Rinv.XP_022323941.1       GCQ-RQGAIVAVTG-----------------------------DGVNDSPALKKADIG--  742
Rinv.ELU12040.1           GCQ-RQGQIVAVTG-----------------------------DGVNDSPALKKADIG--  740
Rinv.XP_013405520.1       GCQ-RQGAIVAVTG-----------------------------DGVNDSPALKKADIG--  754
Rinv.NP_001116982.1       GCQ-RAGAIVAVTG-----------------------------DGVNDSPALKKADIG--  741
Nem.KHJ49479.1            GCQ-RQGAIVAVTG-----------------------------DGVNDSPALKKADIG--  603
Nem.CDW55413.1            GCQ-RQGAIVAVTG-----------------------------DGVNDSPALKKADIG--  768
Nem.KRY76685.1            GCQ-RQGAIVAVTG-----------------------------DGVNDSPALKKADIG--  776
Nem.KRZ73739.1            GCQ-RQGAIVAVTG-----------------------------DGVNDSPALKKADIG--  781

Nem.KRX35740.1            GCQ-RQGAIVAVTG-----------------------------DGVNDSPALKKADIG--  778
Nem.KRZ52541.1            GCQ-RQGAIVAVTG-----------------------------DGVNDSPALKKADIG--  804
Nem.UPI0006120BCD         GFQ-RQGQIVAVTG-----------------------------DGVNDSPALKKADIG--  775
Nem.UPI0007A15AF4         GFQ-RQGQIVAVTG-----------------------------DGVNDSPALKKADIG--  684
Nem.UPI00020239EC         GFQ-RQGQIVAVTG-----------------------------DGVNDSPALKKADIG--  767
Nem.KHN72407.1            GFQ-RQGQIVAVTG-----------------------------DGVNDSPALKRADIG--  766
Nem.UPI0006036B9D         AFQ-REGHVVAVTG-----------------------------DGVNDSPALKKADIG--  705
Nem.XP_024502753.1        GFQ-KQGQIVAVTG-----------------------------DGVNDSPALKKADIG--  688
Nem.UPI000605CA49         GFQ-KQGQIVAVTG-----------------------------DGVNDSPALKKADIG--  710
Nem.UPI000609E51C         GFQ-RQGQIVAVTG-----------------------------DGVNDSPALKRADIG--  667
Nem.UPI0007A24416         GFQ-RQGQIVAVTG-----------------------------DGVNDSPALKRADIG--  684
Nem.XP_003143231.1        GFQ-RQGQIVAVTG-----------------------------DGVNDSPALKRADIG--  708
Nem.UPI0007085A3B         GFQ-RQGQIVAVTG-----------------------------DGVNDSPALKRADIG--  720
Nem.OZC08885.1            GFQ-RQGQIVAVTG-----------------------------DGVNDSPALKRADIG--  708
Nem.UPI00060602BA         GFQ-RQGQIVAVTG-----------------------------DGVNDSPALKRADIG--  721
Nem.UPI000605F508         GFQ-RQGQIVAVTG-----------------------------DGVNDSPALKRADIG--  684
Nem.XP_001901816.1        GFQ-RQGQIVAVTG-----------------------------DGVNDSPALKRADIG--  708
Nem.UPI000818DDE2         GFQ-RQGQIVAVTG-----------------------------DGVNDSPALKRADIG--  738
Nem.UPI0007A17F26         GFQ-RQGQIVAVTG-----------------------------DGVNDSPALKRADIG--  708
Nem.UPI0007A19C37         GFQ-RQGQIVAVTG-----------------------------DGVNDSPALKRADIG--  706
Nem.UPI000605DBB4         GFQ-RQGQIVAVTG-----------------------------DGVNDSPALKRADIG--  706
Nem.KHN82508.1            GFQ-RQGQIVAVTG-----------------------------DGVNDSPALKRADIG--  710
Nem.ADY40856.1            GFQ-RQGQIVAVTG-----------------------------DGVNDSPALKRADIG--  709
Nem.ADY40930.1            GFQ-RQGQIVAVTG-----------------------------DGVNDSPALKRADIG--  718
Nem.UPI0006052FD1         GFQ-RQGQIVAVTG-----------------------------DGVNDSPALKRADIG--  707
Nem.UPI000BC5B284         GFQ-KQGHIVAVTG-----------------------------DGVNDSPALKKADIG--  667
Nem.UPI000BE6443D         GFQ-KQGQIVAVTG-----------------------------DGVNDSPALKRADIG--  723
Nem.UPI0001D4FB7A         GFQ-KQGQIVAVTG-----------------------------DGVNDSPALKRADIG--  711
Nem.UPI0001C851CE         GFQ-KQGQIVAVTG-----------------------------DGVNDSPALKKADIG--  704
Nem.P90735                GFQ-KQGQIVAVTG-----------------------------DGVNDSPALKKADIG--  704
Nem.UPI000293EB87         GFQ-KQGQIVAVTG-----------------------------DGVNDSPALKKADIG--  704
Nem.UPI00060544A6         GFQ-KQGQIVAVTG-----------------------------DGVNDSPALKKADIG--  727
Nem.UPI0007A2DD20         GFQ-KQGQIVAVTG-----------------------------DGVNDSPALKKADIG--  707
Nem.UPI000342C523         GFQ-KQGQIVAVTG-----------------------------DGVNDSPALKKADIG--  772
Nem.U6PGW0                GFQ-KQGQIVAVTG-----------------------------DGVNDSPALKKADIG--  708
```

```
Nem.UPI00060AD39A          GFQ-KQGQIVAVTG-----------------------------DGVNDSPALKKADIG--  695
Rinv.UPI0001782835         GCQ-RQGQIVAVTG-----------------------------DGVNDSPALKRADIG--  698
Rinv.KXJ20422.1            GCQ-RNGAIVAVTG-----------------------------DGVNDSPALKKADIG--  818
Rinv.XP_020601998.1        GCQ-RQGAIVAVTG-----------------------------DGVNDSPALKRADIG--  770
Rinv.XP_020602001.1        GCQ-RQGAIVAVTG-----------------------------DGVNDSPALKRADIG--  755
Art.XP_014232354.1         GCQ-RMGAIVAVTG-----------------------------DGVNDSPALKKADIG--  752
Art.XP_014205893.1         GCQ-RQGAIVAVTG-----------------------------DGVNDSPALKKADIG--  752
Art.XP_012269651.1         GCQ-RMGAIVAVTG-----------------------------DGVNDSPALKKADIG--  749
Art.XP_015585114.1         GCQ-RMGAIVAVTG-----------------------------DGVNDSPALKKADIG--  749
Art.KZC06498.1             GCQ-RMGAIVAVTG-----------------------------DGVNDSPALKKADIG--  715
Art.V9I6A9                 GCQ-RMGAIVAVTG-----------------------------DGVNDSPALKKADIG--  722
Art.XP_012163873.1         GCQ-RMGAIVAVTG-----------------------------DGVNDSPALKKADIG--  749
Art.XP_012272094.1         GCQ-RMGAIVAVTG-----------------------------DGVNDSPALKKADIG--  749
Art.UPI0001FEE5BA          GCQ-RMGAIVAVTG-----------------------------DGVNDSPALKKADIG--  756
Art.EGI67709.1             GCQ-RMGAIVAVTG-----------------------------DGVNDSPALKKADIG--  715
Art.KYN38456.1             GCQ-RMGAIVAVTG-----------------------------DGVNDSPALKKADIG--  715
Art.UPI0005FA3D38          GCQ-RMGAIVAVTG-----------------------------DGVNDSPALKKADIG--  743
Art.XP_020294182.1         GCQ-RMGAIVAVTG-----------------------------DGVNDSPALKKADIG--  750
Art.EZA51212.1             GCQ-RMGAIVAVTG-----------------------------DGVNDSPALKKADIG--  749
Art.UPI0001E7C907          GCQ-RMGAIVAVTG-----------------------------DGVNDSPALKKADIG--  715
Art.XP_024883961.1         GCQ-RMGAIVAVTG-----------------------------DGVNDSPALKKADIG--  749
Art.UPI00091A11F9          GCQ-RMGAIVAVTG-----------------------------DGVNDSPALKKADIG--  709
Art.UPI00091201A3          GCQ-RMGAIVAVTG-----------------------------DGVNDSPALKKADIG--  747
Art.UPI000971E6B0          GCQ-RMGAIVAVTG-----------------------------DGVNDSPALKKADIG--  748
Art.T1E1Y4                 GCQ-RMGAIVAVTG-----------------------------DGVNDSPALKKADIG--  708
Art.XP_021693479.1         GCQ-RMGAIVAVTG-----------------------------DGVNDSPALKKADIG--  748
Art.ETN62539.1             GCQ-RMGAIVAVTG-----------------------------DGVNDSPALKKADIG--  708
Art.UPI000CD7670C          GCQ-RMGAIVAVTG-----------------------------DGVNDSPALKKADIG--  743
Art.UPI0007D6117E          GCQ-RMGAIVAVTG-----------------------------DGVNDSPALKKADIG--  713
Art.UPI000153A0D9          GCQ-RMGAIVAVTG-----------------------------DGVNDSPALKKADIG--  708
Art.UPI0007D2379C          GCQ-RMGAIVAVTG-----------------------------DGVNDSPALKKADIG--  739
Art.UPI000957746B          GCQ-RMGAIVAVTG-----------------------------DGVNDSPALKKADIG--  732
Art.UPI000692F512          GCQ-RMGAIVAVTG-----------------------------DGVNDSPALKKADIG--  746
Art.T1PH35                 GCQ-RMGAIVAVTG-----------------------------DGVNDSPALKKADIG--  710

Art.KNC28219.1             GCQ-RMGAIVAVTG-----------------------------DGVNDSPALKKADIG--  745
Art.UPI0005476AAF          GCQ-RMGAIVAVTG-----------------------------DGVNDSPALKKADIG--  746
Art.UPI000692F5BD          GCQ-RMGAIVAVTG-----------------------------DGVNDSPALKKADIG--  710
Art.XP_004536046.2         GCQ-RMGAIVAVTG-----------------------------DGVNDSPALKKADIG--  710
Art.A0A034W3G9             GCQ-RMGAIVAVTG-----------------------------DGVNDSPALKKADIG--  744
Art.UPI0006929A96          GCQ-RMGAIVAVTG-----------------------------DGVNDSPALKKADIG--  746
Art.XP_004536048.1         GCQ-RMGAIVAVTG-----------------------------DGVNDSPALKKADIG--  710
Art.UPI0005474384          GCQ-RMGAIVAVTG-----------------------------DGVNDSPALKKADIG--  710
Art.UPI0006ED933E          GCQ-RMGAIVAVTG-----------------------------DGVNDSPALKKADIG--  751
Art.UPI0006EDF4F9          GCQ-RMGAIVAVTG-----------------------------DGVNDSPALKKADIG--  747
Art.UPI0006BD3860          GCQ-RMGAIVAVTG-----------------------------DGVNDSPALKKADIG--  710
Art.XP_023176860.1         GCQ-RMGAIVAVTG-----------------------------DGVNDSPALKKADIG--  710
Art.UPI0006D327FB          GCQ-RMGAIVAVTG-----------------------------DGVNDSPALKKADIG--  749
Art.UPI00017C692D          GCQ-RMGAIVAVTG-----------------------------DGVNDSPALKKADIG--  750
Art.UPI0006EE4186          GCQ-RMGAIVAVTG-----------------------------DGVNDSPALKKADIG--  745
Art.XP_022210861.1         GCQ-RMGAIVAVTG-----------------------------DGVNDSPALKKADIG--  749
Art.UPI0007E80B92          GCQ-RMGAIVAVTG-----------------------------DGVNDSPALKKADIG--  749
Art.UPI0001781834          GCQ-RMGAIVAVTG-----------------------------DGVNDSPALKKADIG--  749
Art.NP_732572.             GCQ-RMGAIVAVTG-----------------------------DGVNDSPALKKADIG--  749
Art.UPI00017D261C          GCQ-RMGAIVAVTG-----------------------------DGVNDSPALKKADIG--  749
Art.XP_020799552.1         GCQ-RMGAIVAVTG-----------------------------DGVNDSPALKKADIG--  749
Art.XP_015032883.1         GCQ-RMGAIVAVTG-----------------------------DGVNDSPALKKADIG--  749
Art.AFU25675.1             GCQ-RLGAIVAVTG-----------------------------DGVNDSPALKKADIG--  717
Art.AFU25676.1             GCQ-RLGAIVAVTG-----------------------------DGVNDSPALKKADIG--  717
Art.BAS22117.1             GCQ-RLGAIVAVTG-----------------------------DGVNDSPALKKADIG--  717
Art.AFU25681.1             GCQ-RLGAIVAVTG-----------------------------DGVNDSPALKKADIG--  717
Art.XP_023954931.1         GCQ-RLGAIVAVTG-----------------------------DGVNDSPALKKADIG--  749
Art.XP_022114884.1         GCQ-RLGAIVAVTG-----------------------------DGVNDSPALKKADIG--  749
Art.AFU25678.1             GCQ-RLGAIVAVTG-----------------------------DGVNDSPALKKADIG--  717
Art.UPI00028A5EEF          GCQ-RLGAIVAVTG-----------------------------DGVNDSPALKKADIG--  744
Art.AFU25667.1             GCQ-RLGAIVAVTG-----------------------------DGVNDSPALKKADIG--  744
Art.AFU25673.1             GCQ-RLGAIVAVTG-----------------------------DGVNDSPALKKADIG--  717
Art.AFU25679.1             GCQ-RLGAIVAVTG-----------------------------DGVNDSPALKKADIG--  749
Art.UPI000B392785          GCQ-RLGAIVAVTG-----------------------------DGVNDSPALKKADIG--  717
Art.AFU25694.1             GCQ-RLGAIVAVTG-----------------------------DGVNDSPALKKADIG--  744
Art.XP_021196082.1         GCQ-RLGAIVAVTG-----------------------------DGVNDSPALKKADIG--  749
Art.XP_022817943.1         GCQ-RLGAIVAVTG-----------------------------DGVNDSPALKKADIG--  749
```

```
Art.AFU25670.1          GCQ-RMGAIVAVTG----------------------------DGVNDSPALKKADIG--  713
Art.XP_022903571.1      GCQ-RMGAIVAVTG----------------------------DGVNDSPALKKADIG--  744
Art.UPI00084EC8B0       GCQ-RMGAIVAVTG----------------------------DGVNDSPALKKADIG--  716
Art.U4UIT1              GCQ-RMGAIVAVTG----------------------------DGVNDSPALKKADIG--  760
Art.XP_023016793.1      GCQ-RMGAIVAVTG----------------------------DGVNDSPALKKADIG--  750
Art.AFU25671.1          GCQ-RMGAIVAVTG----------------------------DGVNDSPALKKADIG--  751
Art.AFU25692.1          GCQ-RMGAIVAVTG----------------------------DGVNDSPALKKADIG--  752
Art.UPI000B551D17       GCQ-RMGAIVAVTG----------------------------DGVNDSPALKKADIG--  716
Art.AFU25686.1          GCQ-RMGAIVAVTG----------------------------DGVNDSPALKKADIG--  719
Art.AFU25695.1          GCQ-RMGAIVAVTG----------------------------DGVNDSPALKKADIG--  743
Art.XP_018562050.1      GCQ-RMGAIVAVTG----------------------------DGVNDSPALKKADIG--  750
Art.UPI00028AEC41       GCQ-RMGAIVAVTG----------------------------DGVNDSPALKKADIG--  713
Art.AFU25691.1          GCQ-RMGAIVAVTG----------------------------DGVNDSPALKKADIG--  716
Art.UPI000186D98E       GCQ-RMGAIVAVTG----------------------------DGVNDSPALKKADIG--  743
Art.XP_023711705.1      GCQ-RMGAIVAVTG----------------------------DGVNDSPALKKADIG--  769
Art.W5U4R1              GCQ-RMGAIVAVTG----------------------------DGVNDSPALKKADIG--  720
Art.AHH35009.1          GCQ-RMGAIVAVTG----------------------------DGVNDSPALKKADIG--  720
Art.AFU25683.1          GCQ-RMGAIVAVTG----------------------------DGVNDSPALKKADIG--  716
Art.XP_022184331.1      GCQ-RRGAIVAVTG----------------------------DGVNDSPALKKADIG--  747
Art.UPI00028BBB9D       GCQ-RMGAIVAVTG----------------------------DGVNDSPALKKADIG--  700
Art.UPI00028AA5CE       GCQ-RMGAIVAVTG----------------------------DGVNDSPALKKADIG--  702
Art.UPI0005464DB9       GCQ-RMGAIVAVTG----------------------------DGVNDSPALKKADIG--  721
Art.AFU25668.1          GCQ-RMGAIVAVTG----------------------------DGVNDSPALKKADIG--  745
Art.XP_014271921.1      GCQ-RMGAIVAVTG----------------------------DGVNDSPALKKADIG--  718
Art.XP_014250371.1      GCQ-RMGAIVAVTG----------------------------DGVNDSPALKKADIG--  724
Art.UPI0007325ED7       GCQ-RMGAIVAVTG----------------------------DGVNDSPALKKADIG--  744
Art.XP_015929949.2      GCQ-RQGQIVAVTG----------------------------DGVNDSPALKKADIG--  722
Art.XP_015930974.1      GCQ-RQGAIVAVTG----------------------------DGVNDSPALKKADIG--  714
Art.XP_015907346.1      GCQ-RQGAIVAVTG----------------------------DGVNDSPALKKADIG--  751
Art.XP_023221168.1      GCQ-RMGAIVAVTG----------------------------DGVNDSPALKKADIG--  792
Art.XP_015929951.2      GCQ-RQGAIVAVTG----------------------------DGVNDSPALKKADIG--  752
Art.XP_022253133.1      GCQ-RLGAIVAVTG----------------------------DGVNDSPALKKADIG--  748

Art.UPI0009F0198D       GCQ-RLGAIVAVTG----------------------------DGVNDSPALKRADIG--  774
Art.XP_022668602.1      GCQ-RLGAIVAVTG----------------------------DGVNDSPALKKADIG--  747
Art.UPI000B76A669       GCQ-RLGAIVAVTG----------------------------DGVNDSPALKKADIG--  746
Art.UPI0007AA68F1       GCQ-RLGAIVAVTG----------------------------DGVNDSPALKKADIG--  740
Art.AMK38059.1          GCQ-RLGAIVAVTG----------------------------DGVNDSPALKKADIG--  743
Art.UPI0007717391       GCQ-RLGAIVAVTG----------------------------DGVNDSPALKKADIG--  741
Art.UPI00079F518A       GCQ-RLGAIVAVTG----------------------------DGVNDSPALKKADIG--  742
Rinv.AOG19177.1         GCQ-RQGAIVAVTG----------------------------DGVNDSPALKKADIG--  691
Rinv.AUG84438.1         GCQ-RQGAIVAVTG----------------------------DGVNDSPALKKADIG--  736
Art.KYQ51534.1          GCQ-RMGAIVAVTG----------------------------DGVNDSPALKKADIG--  715
Art.UPI000790B508       GCQ-RMGAIVAVTG----------------------------DGVNDSPALKKADIG--  750
Art.KPI92424.1          GCQ-RLGAIVAVTG----------------------------DGVNDSPALKKADIG--  763
Art.AGZ13694.1-         GCQ-RQGAIVAVTG----------------------------DGVNDSPALKKADIG--  752
Art.UPI000672CD46       GCQ-RMGAIVAVTG----------------------------DGVNDSPALKKADIG--  709
Art.XP_023337795.1      GCQ-RMGAIVAVTG----------------------------DGVNDSPALKKADIG--  713
Art.XP_023336146.1      GCQ-RMGAIVAVTG----------------------------DGVNDSPALKKADIG--  710
Art.XP_023323782.1      GCQ-RMGAIVAVTG----------------------------DGVNDSPALKKADIG--  574
Art.AFU25682.1          GCQ-RMGAIVAVTG----------------------------DGVNDSPALKKADIG--  716
Art.AFU25689.1          GCQ-RMGAIVAVTG----------------------------DGVNDSPALKKADIG--  716
Art.EFX88073.1          GCQ-RGGAIVAVTG----------------------------DGVNDSPALKKADIG--  712
Art.EFX88361.1          GCQ-RLGAIVAVTG----------------------------DGVNDSPALKKADIG--  700
Art.XP_021944101.1      GCQ-RQGAIVAVTG----------------------------DGVNDSPALKKADIG--  709
Art.AIM43570.1          GCQ-RMGAIVAVTG----------------------------DGVNDSPALKKADIG--  721
Art.AFM54541.1          GCQ-RMGAIVAVTG----------------------------DGVNDSPALKKADIG--  717
Art.AEX07319.1          GCQ-RMGAIVAVTG----------------------------DGVNDSPALKKADIG--  719
Art.AIR93635.1          GCQ-RMGAIVAVTG----------------------------DGVNDSPALKKADIG--  746
Art.ADN83843.1          GCQ-RMGAIVAVTG----------------------------DGVNDSPALKKADIG--  746
Art.ABD59803.1          GCQ-RMGAIVAVTG----------------------------DGVNDSPALKKADIG--  746
Art.AAG47843.1          GCQ-RMGAIVAVTG----------------------------DGVNDSPALKKADIG--  747
Art.AGF90965.1          GCQ-RMGAIVAVTG----------------------------DGVNDSPALKKADIG--  747
Art.AGM39710.1          GCQ-RMGAIVAVTG----------------------------DGVNDSPALKKADIG--  745
Art.ABA02167.1          GCQ-RMGAIVAVTG----------------------------DGVNDSPALKKADIG--  718
Art.UPI0000085D74       GCQ-RMGAIVAVTG----------------------------DGVNDSPALKKADIG--  747
Art.AJO70000.1          GCQ-RMGAIVAVTG----------------------------DGVNDSPALKKADIG--  746
Art.AJO70183.1          GCQ-RMGAIVAVTG----------------------------DGVNDSPALKKADIG--  746
Art.AKG50106.1          GCQ-RMGAIVAVTG----------------------------DGVNDSPALKKADIG--  746
Prt.CAI99406.1          NAQ-LRGEVVAVTG----------------------------DGVNDAPALKKGDIG--  735
Prt.UPI0004A1BCA5       NNQ-RRGEVVAVTG----------------------------DGVNDAPALKKGDIG--  736
Prt.UPI00014FFB3C       NCQ-RRGEIVAVTG----------------------------DGVNDAPALKKGDIG--  714
```

```
Prt.A0A090M1W3                    NCQ-RRGEIVAVTG-----------------------------DGVNDAPALKKGDIG--  728
Prt.OUS42873.1                    NCQ-RRGEIVAVTG-----------------------------DGVNDAPALKKGDIG--  728
Art.OWR44555.1(Group I)           TFQ-SLDYVVAVTG-----------------------------DGVNDSPALKKADIG--  726
Art.KPJ02649.1(Group I)           TLQ-SLKHVVAVTG-----------------------------DGVNDSPALKKADIG--  725
Art.XP_004931505.2(Group I)       ACQ-SRGDVVAVTG-----------------------------DGVNDAPALRKADIG--  630
Art.OWR53886.1(Group I)           ACQ-EKGNVVAVTG-----------------------------DGVNDAPALRRADIG--  677
Art.KPJ07193.1(Group I)           ACQ-RRGGVVAVTG-----------------------------DGVNDAPALRRADIG--  696
Art.KPI97351.1(Group I)           ACQ-RRGGVVAVTG-----------------------------DGVNDAPALRRADIG--  689
Art.OXA57033.1                    AYQ-KLGHIVAVSGD-----------------------------GVNDASSLKKANIG--  758
Art.ODM90865.1                    AFQ-ELGNIVAATGDV---------------------------KCVNDSPALKKADIG--  782
Art.ODM96221.1                    AFQ-KLGYIVAATGDGNLIQTTNNRINCPIYFRTYIFLFGPSMKGVNDSPALKKADVG--  713
Art.ODM99113.1                    AFQ-DLGYVVASTGD-----------------------------GVNDSPALKKADIG--  733
Rinv.OQV20867.1                   GVQ-RMGCIVAVTG-----------------------------DGVNDSPALRKADIG--  754
Art.K7IWP3(Group II)              SCQ-RLGEIVAVTG-----------------------------DGVNDSPALRKADIG--  732
Art.EZA47803.1(Group II)          SCQ-RLGEIVAVTG-----------------------------DGVNDSPALRKADIG--  723
Art.KYN03549.1(Group II)          SCQ-RLGEIVAVTG-----------------------------DGVNDSPALRKADIG--  733
Art.KYQ48574.1(Group II)          SCQ-RLGEIVAVTG-----------------------------DGVNDSPALRKADIG--  733
Art.KZC11054.1(Group II)          SCQ-RLGEIVAVTG-----------------------------DGVNDSPALRKADIG--  724
Art.KOX77187.1(Group II)          SCQ-RLGEIVAVTG-----------------------------DGVNDSPALRKADIG--  690
Art.OAD46911.1(Group II)          SCQ-RLGEIVAVTG-----------------------------DGVNDSPALRKADIG--  726
Art.KOC67548.1(Group II)          SCQ-RLGEIVAVTG-----------------------------DGVNDSPALRKADIG--  725
Art.OXA54637.1(Group II)          GFQ-RLGYVVAVTGNVDN----------------PVDLTDPHRDGVNDSPALRKADIG--  788
Art.ODN05419.1(Group II)          GFQ-RLGKIVAVTG-----------------------------DGVNDSPALKKADIG--  779
Prt.EGD77429.1                    GCQ-RAGQIVAVTG-----------------------------DGVNDSPALKRADIG--  941
Nem.CEF64940.1(Group II)          SYQ-RLGNVVAVTG-----------------------------DGVNDAPALKKADIG--  728
Prt.EGD75712.1                    GCQ-RRGDVVAVTG-----------------------------DGVNDSPALRAADIG--  821
Rinv.OQV25202.1                   ACQ-ATGAIVAVTG-----------------------------DGVNDSPALSKADIG--  801
Art.XP_023321169.1(Group II)      AFQ-TTGAIVAVTG-----------------------------DGVNDSPALKKGDIG--  693
Art.D6WB95(Group II)              GCQ-RLGEIVAVTG-----------------------------DGVNDAPALKKADIG--  718
Art.XP_018565491.1(Group II)      GCQ-RLGEIVAVTG-----------------------------DGVNDAPALKKADIG--  718
Art.AFU25672.1(Group II)          GCQ-RLGEIVAVTG-----------------------------DGVNDAPALKKADIG--  719
Art.KYN18319.1(Group II)          SCQ-RLHLITAVTG-----------------------------DGVNDSPALKKADIG--  702
Art.EFN88446.1(Group II)          SCQ-RLHLITAVTG-----------------------------DG---------------  688
Art.A0A087ZR23(Group II)          SCQ-RLHLITAVTG-----------------------------DGVNDAPALKKADIG--  705
Art.XP_012279085.1(Group II)      SCH-RLHLITAVTG-----------------------------DGVNDSPALKKADIG--  699
Prt.BAA82752.2                    ENQ-KRGHIVAVTG-----------------------------DGVNDSPALKQADIG--  775
Prt.OQR99879.1                    NCQ-RRKEIVAVTG-----------------------------DGVNDSPALKKADIG--  789
Prt.OQR92998.1                    NCQ-RRKEIVAVTG-----------------------------DGVNDSPALKKADIG--  788
Prt.XP_008607481.1                NCQ-RRKEIVAVTG-----------------------------DGVNDSPALKKADIG--  790
Prt.XP_008604114.1                NCQ-RRGEIVAVTG-----------------------------DGVNDSPALKKADIG--  786
Prt.OQS04799.1                    NCQ-RRGEIVAVTG-----------------------------DGVNDSPALKKADIG--  782
Prt.OQR94535.1                    NCQ-RRGEIVAVTG-----------------------------DGVNDSPALKKADIG--  782
Prt.CCA16430.1                    NCQ-RRKEIVAVTG-----------------------------DGVNDSPALKKADIG--  784
Prt.POM62354.1                    NCQ-RRKEIVAVTG-----------------------------DGVNDSPALKKADIG--  786
Prt.RAW38513.1                    NCQ-RRKEIVAVTG-----------------------------DGVNDSPALKKADIG--  786
Prt.XP_024585310.1                NCQ-RRKEIVAVTG-----------------------------DGVNDSPALKKADIG--  786
Prt.XP_647420.2                   HLQ-KRGEIVAVTG-----------------------------DGVNDSPALKKADLG--  822
Prt.XP_004352438.1                ECQ-KRGEIVAVTG-----------------------------DGVNDSPALKKADLG--  873
Prt.tr|D3BBA2|                    QCQ-NKGDIVAVTG-----------------------------DGVNDSPALKKADLG--  798
Prt.UPI00000795B1                 NAQ-KRKEVVAVTG-----------------------------DGVNDSPALKKADIG--  703
Prt.F1A2S2                        NAQ-RRKEVVAVTG-----------------------------DGVNDSPALKKADIG--  698
Prt.PXF41383.1                    NNQ-RLGNIVAVTG-----------------------------DGVNDSPALKKANIG--  740
Prt.CAI99405.1                    NCQ-RLAKIVAVTG-----------------------------DGVNDSPALKRANIG--  748
Fun.SPPG_08470.2                  RAQ-ALGHIVGVTG-----------------------------DGVNDAAALKKADLG--  799
Fun.UniRef100_A0A4P9WG54          RAQ-AMGHIVGVTG-----------------------------DGINDAAALKKADLG--  711
Fun.AMAG_01211.1                  HAQ-SLGHIVGVTG-----------------------------DGVNDSPALKKADLG--  718
Fun.AMAG_02439.1                  HAQ-SIGHIVGVTG-----------------------------DGVNDSPALKKADLG--  707
Fun.UniRef100_A0A1Y2HI79          RAQ-SMGHIVGVTG-----------------------------DGVNDSPALKKADLG--  703
Fun.UPI000006A49F                 RAQ-AMGHIVGVTG-----------------------------DGVNDSPALKKADLG--  705
Fun.AAF20202.1                    RAQ-AMGHIVGVTG-----------------------------DGVNDSPALKKADLG--  704
Fun.estExt_Genewise1.C_180045     RAQ-ALGHIVGVTG-----------------------------DGVNDSPALKKADLG--  790
Fun.PGTG_03133.2                  RAQ-ALGHIVGVTG-----------------------------DGVNDSPALKKADLG--  780
Fun.CC1G_09151.2                  RAQ-ALGHIVGVTG-----------------------------DGVNDSPALKKADLG--  696
Fun.UniRef100_A0A067M739          RAQ-ALGHIVGVTG-----------------------------DGVNDSPACKHADLG--  762
Fun.UPI0001643CDB                 RAQ-ALGHIVGVTG-----------------------------DGVNDSPALKKADLG--  734
Fun.UniRef100_A0A5E3X872          RAQ-ALGHIVGVTG-----------------------------DGVNDSPALKKADLG--  788
Fun.fgenesh2_pm.C_sc              RAQ-ALGHIVGVTG-----------------------------DGVNDSPALKKADLG--  733
Fun.UniRef100_A0A4Y9ZEZ1          RAQ-ALGHIVGVTG-----------------------------DGVNDSPALKKADLG--  796
Fun.UniRef100_A0A067Q0Q4          HAQ-ALGHIVGVTG-----------------------------DGVNDSPALKKADLG--  777
Fun.e_gwh2.1.49.1                 RAQ-ALGHIVGVTG-----------------------------DGVNDSPALKKADLG--  733
Fun.UniRef100_A0A4S4MS29          RAQ-ALGHIVGVTG-----------------------------DGVNDSPALKKADLG--  780
```

```
Fun.UniRef100_A0A1M2VF35           RAQ-ALGHIVGVTG-----------------------------DGVNDSPALKKADLG--  602
Fun.UniRef100_J4GT94               HAQ-ALGHIVGVTG-----------------------------DGVNDSPALKKADLG--  775
Fun.UniRef100_A0A4Y9Y8I4           HAQ-ALGHIVGVTG-----------------------------DGVNDSPALKRADLG--  781
Fun.UniRef100_S8E4T6               HAQ-ALGHIVGVTG-----------------------------DGVNDSPALKKADLG--  731
Fun.UniRef100_A0A1Y1VYW4           RAQ-SLGHICGVTG-----------------------------DGVNDSPALKKANLG--  724
Fun.UniRef100_A0A2G5BEZ7           RAQ-ELGHIVGVTG-----------------------------DGVNDSPCIRKADLG--  766
Fun.UniRef100_A0A507F1Y9           RAQ-GRGHIVGVTG-----------------------------DGVNDSPALKKADLG--  742
Fun.UniRef100_A0A1Y2BXZ1           RAQ-GRGHIVGVTG-----------------------------DGVNDSPALKKADLG--  636
Fun.UniRef100_A0A1Y2BYC1           RAQ-GRGHIVGVTG-----------------------------DGVNDSPALKKADLG--  698
Fun.UniRef100_A0A4P9WWS6           RAQ-AAGHLVGVTG-----------------------------DGVNDSPALKKADLG--  748
Fun.UniRef100_A0A507BSP0           RAQ-SRGEIVGVTG-----------------------------DGVNDSPALKKADLG--  751
Fun.BDEG_03368.1                   HAQ-ALGHIVGVTG-----------------------------DGVNDSPALKKADLG--  718
Fun.BDEG_05936.1                   HAQ-ALGHIVGVTG-----------------------------DGVNDSPALKKADLG--  722
Fun.UniRef100_A0A507E7U2           RAQ-ARGHIVGVTG-----------------------------DGVNDSPALKKADLG--  769
Fun.SPPG_07476.2                   RAQ-SRGHIVGVTG-----------------------------DGVNDSPALKKADLG--  605
Fun.UniRef100_A0A507EKQ2           RAQ-SRGHIVGVTG-----------------------------DGVNDSPALKKADLG--  811
Fun.UniRef100_A0A1X2ID33           RAQ-SLGHIVGVTG-----------------------------DGVNDAPALKKADLG--  723
Fun.fgenesh1_pm.12_#_37            RAQ-SMRHIVGVTG-----------------------------DGVNDAPALKKADLG--  671
Fun.RO3G_04175.3                   HAQ-SMRHIVGVTG-----------------------------DGVNDAPALKKADLG--  754
Fun.UniRef100_A0A0B7MNY4           RAQ-SMQHIVGVTG-----------------------------DGVNDAPALKKADLG--  726
Fun.UniRef100_A0A0C9MZH6           RAQ-SMGHIVGVTG-----------------------------DGVNDAPALKKADLG--  756
Fun.fgenesh1_pm.01_#_34            RAQ-AMGHIVGVTG-----------------------------DGVNDAPALKKADLG--  665
Fun.UniRef100_S2JVU7               RAQ-SMRHIVGVTG-----------------------------DGVNDAPALKKADLG--  756
Fun.UniRef100_A0A139AWN6           HAQ-SRGHIVGVTG-----------------------------DGVNDSPALKKADLG--  721
Fun.UniRef100_A0A2Z6QEU7           HAQ-SMGHIVGVTG-----------------------------DGVNDSPALKKADLG--  735
Fun.UniRef100_A0A2N0PA04           HAQ-SLGHIVGVTG-----------------------------DGVNDSPALKKADLG--  735
Fun.UniRef100_A0A397VHQ3           RAQ-SMGHIVGVTG-----------------------------DGVNDSPALKKADLG--  746
Fun.SPPG_01615.2                   RAQ-NLGHIVGVTG-----------------------------DGVNDSPALKKADLG--  706

Fun.UniRef100_A0A194X4V3           RAQ-SMGHIVGVTG-----------------------------DGVNDAPALKKADLG--  740
Fun.UniRef100_A0A261Y8Y1           RAQ-SMGHIVGVTG-----------------------------DGVNDSPALKKADLG--  777
Fun.UniRef100_A0A0C3GKR9           RAQ-SMGHIVGVTG-----------------------------DGVNDSPALKKADLG--  770
Fun.UniRef100_A0A2J6SAB3           RAQ-SMGHIVGVTG-----------------------------DGVNDSPALKKADLG--  792
Fun.UniRef100_A0A2J6SIQ3           RGQ-SMGHIVGVTG-----------------------------DGVNDSPALKKADLG--  775
Fun.fgenesh2_pm.7_#_193            RAQ-SMGHIVGVTG-----------------------------DGVNDSPALKAADLG--  767
Fun.UniRef100_A0A090D7D5           RAQ-EMGHIVGVTG-----------------------------DGVNDSPALKKADLG--  748
Fun.UniRef100_A0A447CBT6           RAQ-EMGHIVGVTG-----------------------------DGVNDSPALKKADLG--  748
Fun.UniRef100_A0A507CSE4           HAQ-AIGHIVGVTG-----------------------------DGVNDSPALKKADLG--  775
Fun.UniRef100_A0A4P9Y2Y3           RAQ-SMGHIVGVTG-----------------------------DGVNDSPALKTADLG--  726
Fun.UniRef100_A0A1Y2G5J7           HAQ-SMGHIVGVTG-----------------------------DGVNDSPALKKADLG--  755
Fun.UniRef100_A0A4P9ZM46           RAQ-AMGHIVGVTG-----------------------------DGVNDSPALKKADLG--  744
Fun.UniRef100_A0A4P9Z1Y8           HAQ-SLGHIVGVTG-----------------------------DGVNDSPALKKADLG--  751
Fun.UniRef100_A0A137NQA6           RAQ-SMGHIVGVTG-----------------------------DGVNDSPALKKADLG--  699
Fun.UniRef100_A0A1Y1YKS1           RAQ-AMGHIVGVTG-----------------------------DGVNDSPALKKADLG--  764
Fun.UniRef100_A0A1Y1YU47           HAQ-AMGHIVGVTG-----------------------------DGVNDSPALKKADLG--  760
Nem.KHN74191.1(Group I)            ------------------------------------------------------------  799
Nem.NP_001122529.1(Group I)        EVQ-RRGETVAVTG-----------------------------GGVDDTPVLAHANVG--  830
Nem.tr|O16436|(Group I)            ESQ-KRGECVTVTG-----------------------------DGVNDAPALKKADVG--  759
Nem.tr|O16331|(Group I)            ESQ-KRGECVTVTG-----------------------------DGVNDAPALKKADVG--  763
Nem.KHN88767.1(Group I)            KCQ-HRGEVVTVTG-----------------------------DGVNDAPALKKANVG--  806
Nem.KHN88766.1(Group I)            KCQ-ERGEVVCVTG-----------------------------DGVNDAPALKRANIG--  828
Prt.XP_024578788.1                 LSQ-EVGECVGVTG-----------------------------DGVNDAPALKQADVG--  782
Prt.GAX12878.1                     KCQTLLGAIVAVTG-----------------------------DGVNDAPALKQADVG--  768
Prt.GAX20661.1                     KCQALLGAIVAVTG-----------------------------DGVNDAPALKQADVG--  768
Prt.UPI00015F4774                  NNQ-RLKRVVAVTG-----------------------------DGVNDAPALKKGDIG--  655
Prt.UPI000D26B24D                  NCQ-RRGENVAVTG-----------------------------DGVNDAPALKKANTG--  774
Prt.XP_001427178.1                 GCQ-KRGHIVAVTG-----------------------------DGVNDSPAIKKADIG--  806
Prt.XP_001346890.1                 ACQ-YIGHVVGVTG-----------------------------DGVNDSPAIKQGDIG--  815
Prt.UniRef100_Q23EX6               ACQ-SVGFICAVTG-----------------------------DGVNDSPAIKQADIG--  819
Prt.UniRef100_Q22XZ1               VCQ-REGYIVAGIG-----------------------------NNYYDFKMIKQAEVG--  767
Prt.UniRef100_Q22LQ9               ACQ-MEGFIVAATG-----------------------------DGVNDSPAIKKADIG--  815
Prt.UniRef100_Q245Y8               ACQ-KEGFIVAATG-----------------------------DGVNDSPAIKKADIG--  825
Prt.UniRef100_Q23ZA6               ACQ-KEGFIVAATG-----------------------------DGVNDSPAIKKADIG--  812
Prt.UniRef100_I7M7N1               ACQ-KEGFIVAATG-----------------------------DGVNDSPAIKKADIG--  826
Prt.UniRef100_I7ME52               ACQ-KEGFIVAATG-----------------------------DGVNDSPAIKKADIG--  835
Prt.UniRef100_I7MH18               ACQ-QEKYICAVTG-----------------------------DGVNDSPAIKQGDIG--  819
Prt.UniRef100_I7MD85               ACQ-KEGFICAVTG-----------------------------DGVNDSPAIKQGDIG--  813
Prt.UniRef100_Q23D88               ACQ-AEGYVCAVTG-----------------------------DGVNDSPAIKQGDIG--  824
Prt.UniRef100_I7MHE1               ACQ-KEGFITAVTG-----------------------------DGVNDSPAIKQGDIG--  820
Prt.UniRef100_I7M7R6               ACQ-KEGFTCAVTG-----------------------------DGVNDSPAIKQGDIG--  1023
Prt.UniRef100_Q22P96               ACQ-QEGYICAVTG-----------------------------DGVNDSPAIKQGDIG--  834
Prt.UniRef100_Q22PA2               ACQ-KEGQIVAVTG-----------------------------DGVNDSPAIKQGNIG--  812
```

```
Prt.PXF41326.1              NLQ-RMGEIVTVTG-----------------------------DGCNDAPALKQANTG--  728
Prk.YP_324582.1             AYK-DIGDVVAVTG-----------------------------DGVNDAPALRAAHIG--  697
Prk.NP_440621.1             AYQ-GLGQIVAVTG-----------------------------DGVNDAPALRAANIG--  705
Prk.UPI0008639F5F           ILE-SEDEIVAMTG-----------------------------DGVNDAPALRKADIG--  674
Prk.NP_276630.1             VLE-DSDEIVAMTG-----------------------------DGVNDAPALRKADIG--  653
Prk.YP_502111.1             VLR-ETGEIVAVTG-----------------------------DGVNDAPALKKADIG--  657
Prk.tr|A0A347ZR85|          ALQ-ELGFIVAVTG-----------------------------DGVNDAPALKKANIG--  689
Prk.UPI00032DDB86           NLQ-QLGEVVASTG-----------------------------DGVNDAPALKKADIG--  657
Prk.tr|A0A4R8A713|          MLQ-EMGETVAVTG-----------------------------DGVNDSPALKKADIG--  667
Prk.YP_001963725.1          ILQ-ELGEIVAVTG-----------------------------DGVNDGPALKKADIG--  775
Prk.YP_391334.1             LLQ-ENGEVVAMTG-----------------------------DGVNDSPALKQADIG--  632
Prk.tr|W5W8S0|              ALR-HRGDLVAMTG-----------------------------DGVNDAPALRRADIG--  581
Prk.tr|A0A1M7YB86|          ALQ-QLEKVVAMTG-----------------------------DGVNDAPALKAADVG--  630
Prk.YP_357688.1             ALQ-AMDKLVAMTG-----------------------------DGVNDAPALKAADVG--  636
Prk.tr|A0A1G6WYT0|          ALK-NQDQVVAMTG-----------------------------DGVNDAPALKAADIG--  633
Prk.tr|A0A1M6NFZ1|          AMK-SLGRVVAMTG-----------------------------DGVNDAPALKAADVG--  634
Prk.tr|A0A1H7UXF5|          ILQ-EEGQWVAVTG-----------------------------DGVNDAPALKKADIG--  624
Prk.tr|B5YJF3|              LLQ-ESGQIVAVTG-----------------------------DGVNDAPALKKADIG--  607
Prk.tr|A0A317MXL5|          ALQ-ARGEVVAVTG-----------------------------DGVNDAPALKRADVG--  616
Prk.tr|A0A1H1W1D1|          ALQ-RKGAVVAATG-----------------------------DGVNDAPALRRADIG--  630
Prk.UPI0002387025           ALK-RKGEVVAVTG-----------------------------DGVNDAPALKAADIG--  625
Prk.tr|A0A497XH49|          ALQ-RKGEIVAVTG-----------------------------DGVNDAPALKTADIG--  629
Prk.UPI0000E10E8B           ALK-DMGEVVAVTG-----------------------------DGVNDAPALKRADIG--  638
Prk.UPI000C2216DF           LLK-EMGEVVAVTG-----------------------------DGVNDAPALRRADVG--  660
Prk.UPI000C21D419           LLK-EMGEVVAVTG-----------------------------DGVNDAPALRRADVG--  660
Prk.UPI000B509329           ALQ-RRGEVVAVTG-----------------------------DGVNDAPALKHADIG--  634

Prk.tr|K0C7I7|              ALQ-KKDQIVTVTG-----------------------------DGVNDAPALKNADMG--  628
Prk.UPI00057321B9           ALQ-SMGEVVTVTG-----------------------------DGVNDAPALKNADMG--  629
Prk.UPI000615B8D6           LFQ-AEGEIVTMTG-----------------------------DGVNDAPAIKNADMG--  679
Prk.AKB42896.1              LFQ-AEGEIVTMTG-----------------------------DGVNDAPAIKNADMG--  679
Prk.UPI0006157E2A           LFQ-SEGEIVTMTG-----------------------------DGVNDAPAIKNADMG--  690
Prk.UPI00061563E3           LFQ-SQGEIVTMTG-----------------------------DGVNDAPAIKNADMG--  672
Prk.AKB83839.1              LFQ-SQGEIVTMTG-----------------------------DGVNDAPAIKNADMG--  672
Prk.UPI00003C6559           LFQ-AEGEIVTMTG-----------------------------DGVNDAPAIKNADMG--  683
Prk.UPI0006157AA6           LFQ-AEGEIVTMTG-----------------------------DGVNDAPAIKNADMG--  683
Prk.UPI0006157148           LFQ-AEGEIVTMTG-----------------------------DGVNDAPAIKNADMG--  689
Prk.NP_633093.1             LFQ-AEGEIVTMTG-----------------------------DGVNDAPAIKNADMG--  689
Prk.UPI000615BB49           LFQ-AEGEIVTMTG-----------------------------DGVNDAPAIKNADMG--  683
Prk.UPI0000068661           LFQ-AEGEIVTMTG-----------------------------DGVNDAPAIKNADMG--  663
Prk.UPI0006154E5D           LFQ-AEGEIVTMTG-----------------------------DGVNDAPAIKNADMG--  663
Prk.YP_565169.1             AFQ-SRGEIVTMTG-----------------------------DGVNDAPAIKNADMG--  605
Prk.UPI0008DEB8E8           AFQ-AAGEIVTMTG-----------------------------DGVNDAPAMKNADMG--  638
Prk.UPI000891AE6A           AFQ-EAGEIVTMTG-----------------------------DGVNDAPAMKNADMG--  638
Prk.UPI00079C7F01           AFQ-SAGETVTMTG-----------------------------DGVNDAPAIKNADMG--  641
Prk.UPI00028B8ECD           AFQ-SAGEIVTMTG-----------------------------DGVNDAPAIKNANMG--  638
```
