# Supplementary material for: Bioinformatics approaches for classification and investigation of the evolution of the Na/K-ATPase alpha-subunit
Source: BMC Ecol Evol. 2022 Oct 26;22:122. doi: 10.1186/s12862-022-02071-0 (PMC9609216; doi:10.1186/s12862-022-02071-0)
Supplement: Supplementary file 1 — Additional file 1. Supplementary figures and tables. [file 12862_2022_2071_MOESM1_ESM.zip › Additional file 1 Fig. S9.pdf]

```
a4.XP_004448484.1      KMKRKKKKEDIEELKAEVIMDDHKLSLENLSAKYSVDLTK-GLTAQQAKEILARDGPNSL    89
a4.XP_023103614.1      KMKRRTMKADMEELKKEAVMDDHKLTLEELSTKYSVDLTK-GLSPKKAQEILNRDGPNVL    90
a4.XP_010593170.2      ----------MEELKKEVVMDDHKLTLKQLSAKYSVDLTK-GLTSEQAQEILGREGPNTL    49
a4.XP_020740848.1      KVKRRKKKKDLEELKQEVVMDDHRLTLDELSAKYSVDLRR-GHSPEKAQEILTRDGPNTL    89
a4.XP_023496657.1      KVKRKKNKANIEELKKEVVMDDHKLTLEELSRRYSVDLTK-GLRPEKAQEILDRDGPNTL    95
a4.XP_006096963.1      ISKKKQKKIDLEELKKELVMNDHKLTLEQLSTKYSVDLTK-GLSSEEAQKILARNGPNTL    90
a4.XP_006922963.1      RVKKKRKKIDVEDLKKEVVMDDHKLTLEQLSTKYSVDLTK-GLSPEEAEKILIRNGPNVL    89
a4.XP_011371380.1      RVKKKRKKIDVEELKKEVVMDDHKLTLEQLSTKYSVDLTK-GLSPEEAEKILIRDGPNVL    89
a4.XP_545754.3         KMKRKKKKTDMEELKKEVVLDDHKLTLEELSTKYSVDLMM-GLSPERAQEILKKNGPNAL    90
a4.XP_021537588.1      KMKSN-KKTELEELKKEVVLEDHKLTLEELSSKYSVDLTM-GLSPRRAQEILRQDGPNTL    89
a4.XP_021506251.1      PIKTKKKKKDLEDLKKEADMNDHKLTLDELSAKYCVDLIK-GHSLKTAQEILLLHGPNAL    88
a4.NP_074039..         -PKVKRRKKDLEELKKEVVMDDHKLTLDELSAKYSVDLTK-GLSVTDAQEILTLNGPNVL    85
a4.XP_021014708.1      -PKMKRKKKDLEELKKEVVMDDHKLTLDELSAKYSVDLTK-GLSVVEAQEILFQNGPNAL    89
a4.NP_038762..         -PKMKRRKKDLEELKKEVVMDDHKLTLDELSAKYSVDLTK-GLSILEAQDILFQNGPNVL    89
a4.XP_020024800.1      --KTVKKKKDMEELKKEVLMEDHKLTLEELSAKYSVNLTM-GHNPEKAKEILIRDGPNIL    83
a4.XP_003795244.1      --MKKKNKRDLEELKKEVIMDDHKLTLEELSAKYSVDLTK-GLSPQKAKEILLRDGPNTL    89
a4.XP_004639995.1      NVKRGKKKKDIEELKKEVVMDDHKLTLEELSAKYSVNLTH-GLSPEKAKEILLRDGYNTL    94
a4.XP_021568356.1      -RKAKWKQKDLEELKKEVVMDDHKLTLEELSAKYAVDLTK-GHSPRKAEELLIRDGPNIL    88
a4.XP_012604632.1      --RRKRKKADIEELKKEVAMDDHKLTLEELSAKYSVDLTT-GHSPEKANEILIRDGPNTL    87
a4.NP_653300.          -VKREKQKRNMEELKKEVVMDDHKLTLEELSTKYSVDLTK-GHSHQRAKEILTRGGPNTV    88
a4.XP_003892961.1      -VKRKKQKINMEELKKEVVMDDHKLTLEELSTKYSVDLTK-GHSHQMAREILTRNGPNTL    88
a4.XP_023069991.1      -VKRKKQKSNMEELKKEVVMDDHKLTLEELSAKYSVDLTK-GHSHQMAKEILTRDGPNTL    88
a4.UPI0001C650F7       -QKRKLKRKNVEELKQEVVLDDHRLTLEQLSAKYSVDLSR-GHTPERAKGLLIQNGPNVL    86
a4.XP_013220247.1      -------------MTLLSLQDDHKLTLEELSAKYSVDLNK-GLSPKKAKENLLRDGPNAI    46
a1.XP_023390675.1.     ----------MDELKKEVSMDDHKLSLDELHRKYGTDLSR-GLTSARAAEILARDGPNAL    49
a2.Q98SL3              GSSSDEKKKDLDELKKEVALDDHKLSLTDLASRYGVDLNK-GLTTKRAAEILERDGPNAL    66
a1.BAJ13363.1          -K--AKEKMDKDDLKKEVDLDDHKLTLDELNRKYGTDLAR-GLSSVRAKEILLRDGPNTL    78
a1.UPI00001DFF4A       KK--AKEKMDKDDLKKEVDLDDHKLTLDELNRKYGTDLAR-GLSSVRAKEILLRDGPNTL    79
a1.UPI00025FADDE       GN----KSKDTEDLKKEVDLDDHKLSFDELHRKYGTDLTR-GLSSSKAKEILARDGPNAL    77
a1.NP_571762.1         TKKGKR-KKDVDELKKEVELDDHKLTLDELSRKYGTGMIK-GLSSFRAKEILERDGPNAL    80
a1.NP_571763.1         PKKGKKNKKDMDELKKEVEMDDHKLTLEELSRKYGTDLNK-GLSITRAKEILARDGPNAL    81
a1.NP_835200.1         PKKGKKNKKDMDELKKEVEMDDHKLTMEELSRKYGTDLTK-GLPVSRAMEVLMRDGPNAL    81
a3.UPI000C736357       -NKG-KERRDLDDLKKEVAMTEHKMSVEEVCRKYNTDCVQ-GLTHSKAQEILARDGPNAL    66
a3.F7E0B8.             -GKGV-QKKEIDDLKKEVAMTEHKITVEEVCRKLNTDCVQ-GLTLSKAAEILARDGPNAL    82
a3.UPI0003CD047A       -AKGTKDRRDLDDLKKEVAMTEHKMSVEEVCRKYNTDCVQ-GLTHSKAQEILARDGPNAL    85
a3.UPI000C73EFA7       -NKG-KERRDLDDLKKEVAMTEHKMSVEEVCRKYNTDCVQ-GLTHSKAQEILARDGPNAL    106
a3.UPI00049A9E19       ----TKSKKDMDDLKKEVSMTEHKMSVEEVCRKYNTDCVQ-GLALSKAQEVLARDGPNAL    89
a3.XP_025028557.1      -NKAQQKVRDLDDLKKEVAMTEHKMSIEEVCRKYNTDCVQ-GLTHSKAQEILARDGPNAL    84
a3.XP_020663591.1      -TKAQQKVRDLDDLKKEVAMTEHKMSIEEVCRKYNTDCVQ-GLTHSKAAEILARDGPNAL    145
a3.UPI0000124FC2       -PKKGKGKRDLDDLKKEVAMTEHKMSIEEVCRKYNTDCVQ-GLTHSKAQEILARDGPNAL    67
a3.UPI000BAD5294       -NKG-KERRDLDDLKKEVAMTEHKMSVEEVCRKYNTDCVQ-GLTHSKAQEILARDGPNAL    66
a3.XP_020948935.1      -GKGTKDRRDLDDLKKEVAMTEHKMSVEEVCRKYNTDCVQ-GLTHSKAQEILARDGPNAL    84
a3.XP_012613923.1      -NK-AKERRDLDDLKKEVAMTEHKMSVEEVCRKYNTDCVQ-GLTHSKAQEILARDGPNAL    70
a3.UPI000C2D7C35       -SKGTKDRRDLDDLKKEVAMTEHKMSVEEVCRKYNTDCVQ-GLTHSKAQEILARDGPNAL    87
a3.XP_006903931.1      -------------------MTEHKMSVEEVCRKYNTDCVQ-GLTHSKAQEILARDGPNAL    40
a3.XP_023380497.1      -SKGAKERRDLDDLKKEVAMTEHKMSVEEVCRKYNTDCVQ-GLTHSKAQEILARDGPNAL    141
a3.UPI0002B3612F       -SKGTKDRRDLDDLKKEVAMTEHKMSVEEVCRKYNTDCVQ-GLTHSKAQEILARDGPNAL    80
a3.XP_020726792.1      -------------------------MSVEEVCRKYNTDCVQ-GLTHSKAQEILARDGPNAL    35
a3.UPI000226419C       -SKGTKDRRDLDDLKKEVAMTEHKMSVEEVCRKYNTDCVQ-GLTHSKAQEILARDGPNAL    85
a3.UPI000C7286EF       -NKG-KERRDLDDLKKEVAMTEHKMSVEEVCRKYNTDCVQ-GLTHSKAQEILARDGPNAL    70
a3.UPI0007A6EC9C       -SKAA-KSRDMDDLKKEVAMTEHKMSVEEVCRKYNTDCVQ-GLTHSKAQEILARDGPNAL    70
a3.UPI000C2DAA95       -NKG-KERRDLDDLKKEVAMTEHKMSVEEVCRKYNTDCVQ-GLTHSKAQEILARDGPNAL    76
a3.UPI000C740E55       -NKG-KERRDLDDLKKEVAMTEHKMSVEEVCRKYNTDCVQ-GLTHSKAQEILARDGPNAL    81
a3.XP_021021704.1      -SKA-KERRDLDDLKKEVAMTEHKMSVEEVCRKYNTDCVQ-GLTHSKAQEILARDGPNAL    83
a3.UPI00035B05DE.      -SKAAKERRDMEDLKKEVAMTEHKMSVEEVCRKYNTDCVQ-GLTHSKAQEILARDGPNAL    105
a3.XP_023600635.1      ----------MEDLKKEVAMTEHKMSVEEVCRKYNTDCVQ-GLTHSKAQEILARDGPNAL    49
a3.UPI000C732D5F       -NKG-KERRDLDDLKKEVAMTEHKMSVEEVCRKYNTDCVQ-GLTHSKAQEILARDGPNAL    81
a3.NP_036638.          -SK-AKERRDLDDLKKEVAMTEHKMSVEEVCRKYNTDCVQ-GLTHSKAQEILARDGPNAL    70
a3.UPI000CB4CAB6       -NKG-KERRDLDDLKKEVAMTEHKMSVEEVCRKYNTDCVQ-GLTHSKAQEILARDGPNAL    81
a3.XP_022441242.1      -SKGTKDRRDLDDLKKEVAMTEHKMSVEEVCRKYNTDCVQ-GLTHSKAQEILARDGPNAL    84
a3.UPI000651771D       -NKG-KERRDLDDLKKEVAMTEHKMSVEEVCRKYNTDCVQ-GLTHSKAQEILARDGPNAL    70
a3.UPI000C7355ED       -NKG-KERRDLDDLKKEVAMTEHKMSVEEVCRKYNTDCVQ-GLTHSKAQEILARDGPNAL    95
a3.XP_021590883.1      -SKG-KERRDLDDLKKEVAMTEHKMSVEEVCRKYNTDCVQ-GLTHSKAQEILARDGPNAL    107
a3.XP_024433413.1      -SKA-KERRDMDDLKKEVAMTEHKMSVEEVCRKYNTDCVQ-GLTHSKAQEILARDGPNAL    70
a3.UPI000C2EDFE7       -NKG-KERRDLDDLKKEVAMTEHKMSVEEVCRKYNTDCVQ-GLTHSKAQEILARDGPNAL    81
a3.UPI000C2E3154       -NKG-KERRDLDDLKKEVAMTEHKMSVEEVCRKYNTDCVQ-GLTHSKAQEILARDGPNAL    83
a3.XP_023507169.1      -NKGTKERRDLDDLKKEVAMTEHKMSVEEVCRKYNTDCVQ-GLTHSKAQEILARDGPNAL    71
a3.KFO33633.1          -------------------MTEHKMSVEEVCRKYNTDCVQ-GLTHSKAQEILARDGPNAL    40
a3.UPI000C71DF25       -NKG-KERRDLDDLKKEVAMTEHKMSVEEVCRKYNTDCVQ-GLTHSKAQEILARDGPNAL    81
a3.UPI0001914BDE       -NKG-KERRDLDDLKKEVAMTEHKMSVEEVCRKYNTDCVQ-GLTHSKAQEILARDGPNAL    83
a3.XP_003799510.1      -NKA-KERRDLDDLKKEVAMTEHKMSVEEVCRKYNTDCVQ-GLTHSKAQEILARDGPNAL    83
```

```
a3.XP_008065591.1      -------------------MTEHKMSVEEVCRKYNTDCVQ-GLTHSKAQEILARDGPNAL   40
a3.sp|P13637.3|        -NKG-KERRDLDDLKKEVAMTEHKMSVEEVCRKYNTDCVQ-GLTHSKAQEILARDGPNAL   70
a3.UPI000050D2B6       --KG---GKDMDELKKEVPITEHKMSIEEVCRKFQTDCVQ-GLTNAKAAEYLIRDGPNAL   65
a3.UPI0007F716B9       --KG---GKDMEDLKKEVPITEHKMSVEEVCRKLQTDIVQ-GLTNAKAAEYLLRDGPNSL   66
a3.UPI0000E9CD46       ----GKGQKDMDDLKKEVPITEHKMSIEEICRKLNTDIVQ-GLTNAKAAEFLIRDGPNAL   78
a3.UPI0007F7EA5B       --K---GGKDLDDLKKEVPITEHKMSVEEVCRKYNTDIVQ-GLTNARAAEYLARDGPNAL   79
a3.XP_020466584.1      --KGE--NKNMDDLKKEVPITEHKMSIEEVCRKYQTDIVQ-GLTNAKAAEILQRDGPNAL   80
a3.UPI0006B30A18       --KGP-GAKDMDDLKKEVPITEHKMSVEEVTRKFQTDIVQ-GLTNAKAAEILIRDGPNAL   81
a3.UPI0004448FEC       --K---GAKDMDDLKKEVPITEHKMSVEEVCRKFQTDIVQ-GLTNAKAAEILIRDGPNAL   66
a3.XP_023187147.1      --K---GGKDMDDLKKEVPITEHKMSVEEVCRKFQADMVQ-GLTNAKAAEILIRDGPNAL   79
a3.UPI0000124FC4       --KG--GTKDMDALKKEVPITEHKMSVEEVCRKFQTDVVQ-GLTNAKAAEFLLRDGPNAL   67
a3.UPI00025FB25F       --KG--GTKDMDDLKKEVPITEHKMSVEEVCRKFQTDVVQ-GLTNAKAAEFLLRDGPNAL   67
a3.UPI0000E3A2FA       --KG---AKDMDDLKKEVPITEHKMSIEEVCRKYQTDIVQ-GLTNAKAAEYLIRDGPNAL   80
a3.UPI00003628C3       --G---ATKDMDDLKKEVPITEHKMSVEEVCRKFQTDIVQ-GLTNARAAEFLLRDGPNAL   77
a3.XP_020504733.1      --KGAEGRKDMDDLKKEVPITEHKMSIEEVCRKFQTDIVQ-GLTNAKAAEFLIRDGPNSL   82
a3.UPI00032B9010       --KG--ATKDMDDLKKEVPITEHKMSVEEVCRKFQTDIVQ-GLTNAKAAEFLLRDGPNAL   67
a3.XP_022612296.1      --KA--GAKDMDDLKKEVPITEHKMSVEEVCRKFQTDIVQ-GLTNAKAAEFLIRDGPNAL   67
a3.XP_023285663.1      --KA--GTKDMDDLKKEVPITEHKMSVEEVCRKFQTDIVQ-GLTNAKAAEFLIRDGPNAL   80
a3.XP_021427657.1      DSKADKKTKDMEELKKEVPLTEHKMSIEEVCQKYSTDIVQ-GLTNAKAAEYLARDGPNAL   85
a3.XP_024297426.1      DSKADKKTKDMEELKKEVPLTEHKMSIEEVCQKYSTDIVQ-GLTNAKAAEYLARDGPNAL   90
a3.UPI00001DFF47       --KKNKKGKDMDELKKEVPITEHKMSIEECCRKFNTDIVQ-GLTNAKAAEFLIRDGPNCL   68
a3.UPI0006B7181A       --KKNKKGKDMDELKKEVPITEHKMSIEECCRKFNTDIVQ-GLTNAKAAEFLIRDGPNCL   81
a3.UPI000293B6B0       --KK-KGGKDIDDLKKEVPITEHKMSVEEVCRKYNTDIVQ-GLTNARAAEFLARDGPNAL   79
a3.UPI0000E3AF2C       --KK-KAGKDMDDLKKEVPITEHKMSVEEVCRKYSTDIVQ-GLTNAKAAEYLIRDGPNAL   79
a3.XP_024920682.1      --KKGKGGKDLDDLKKEVPITEHKMSVEEVCRKYNTDIVQ-GLTNAKAAEYLARDGLNAL   80
a3.UPI00016E235F       --KK-KGGKDLDDLKKEVPITEHRMSVEEVCRKYSTDIVQKGLTNAKAAEYLARDGPNAL   82
a3.UPI00032B6FE9       --KKGKGGKDLDDLKKEVPIMEHKMSVEEVCRKYNTDIVQ-GLTNARAAEYLARDGPNAL   80
a3.XP_012711044.2      --KK-KGGKDLDDLKKEVPITEHKMSVEEVCRKYTTDIVQ-GLTNAKAAEFLARDGPNAL   79
a3.UPI00025F91A4       --KK-KGGKDLDDLKKEVPITEHKMSVEEVCRKYNTDIVQ-GLTNARAAEYLARDGPNAL   79
a3.XP_020793662.1      --KK-KGGKDLDDLKKEVPITEHKMSVEEVCRKYNTDIVQ-GLTNARAAEYLARDGPNAL   79
a3.XP_024153267.1      --KK-KGGKDLDDLKKEVPITEHKMSVEEVCRKYTTDIVQ-GLTNARAAEYLARDGLNAL   79
a3.XP_023117914.1      --KK-KGGKDLDDLKKEVPITEHKMSVEEVCRKYNTDIVQ-GLTNAKAAEYLARDGLNAL   79
a3.XP_022053465.1      --KK-KGGKDLDDLKKEVPITEHKMSVEEVCRKYNTDIVQ-GLTNAKAAEYLARDGLNAL   79
a3.NP_571759.2         --KKGKGGKDLDDLKKEVPLTEHKMSIEEVCRKYNTDIVQ-GLTNARAAEYLARDGPNAL   80
a3.W5UML4              --PKKKGGKDLDDLKKEVPLTEHKMSIEEVCQKFNTDIVQ-GLTNAKAAEYLIRDGPNAL   81
a3.W5L4G0.             --PKKKGGKDMDDLKKEVPLTEHKMSIEEVCRKYNTDIVQ-GLTNAKAAEYLARDGPNAL   79
a3.UPI0005D90DB9       --PKKKGGKDLDDLKREVPLTEHKMSVEEVCRKYNTDIVQ-GLTNARAAEYLARDGPNAL   79
a3.XP_023665796.1      --KK--KGKDLDDLKKEVPLTEHKMSVEEVCRKFNTDIVQ-GLTNAKAAEYLIRDGPNAL   79
a3.XP_015461719.2      --KNKKGNKDLDDLKKEVPLTEHKMSVEEVCRKFQTDIVQ-GLTNAKAAEFLQRDGPNAL   81
a3.BAB60722.1          ----KKGTKDLDDLKKEVPLTEHKMSVEEVCRKFQTDIVQ-GLTNAKAREFLARDGPNAL   79
a3.UPI000054C9F5       --K-KKGAKDLDDLKKEVPLTEHKMSVEEVCRKFQTDIVQ-GLTNAKARDFLARDGPNAL   80
a1.NP_571761.1         SKGKKEKDKDMDELKKEVDLDDHKLSLDELTRKYNTDLTR-GLSGTRAKEILARDGPNAL   83
a1.AJR20270.1          SKGKKDKDKDMDELKKEVDLDDHRLSLEDLSRKYGTNLTR-GLTASRAKEILARDGPNAL   83
a1.XP_023690671.1      KP-K-KKEKDMDELKKEVDLDDHKLTLDELHRKYSTDLSR-GLANTRAAEILARDGPNAL   81
a1.XP_008322794.1      KG-K-KKEKDMDELKKEVDMDDHKLTLDELARKYATDLSN-GLTCAKAAENLARDGPNAL   112
a1.XP_020497843.1      KG-K-KKEKDMDELKKEVDMDDHKLTLDELNRKYGTDLNN-GLTSAKAAEILARDGPNAL   93
a1.UPI00066EFDEA.      KG-K-KKEKDMDELKKEVDMDDHKLTLDELNRKYGTDLNN-GLTSAKAAEILARDGPNAL   85
a1.XP_004571307.1      KG-K-KKEKDMDELKKEVDMDDHKLTLDELNRKYGTDLTN-GLTSEKAAEILARDGPNAL   81
a1.XP_022617258.1      KG-K-KKEKDMDELKKEVDMDDHKLTLDELNRKYGTDLSN-GLTSAKAAEILARDGPNAL   81
a1.BAN17691.1          KG-K-KKEKDMDELKKEVDMDDHKLTLDELARKYGTDLSN-GLTGVKAAEVLARDGPNAL   81
a1.XP_012714443.1      KG-K-KKEKDMDELKKEVDMDDHKLNLDELQRKYGTDLSN-GLTGAKAAEILARDGPNAL   81
a1.XP_017282368.1      KG-KGKKEKDMDELKKEVDMDDHKLTLDELNRKYGTDLTN-GLSSAKAAEILARDGPNAL   85
a1.XP_004066573.1      KG-E-KKKKDMDELKKEVDMEDHKLTLDELSRKYETDLSN-GLTGARSAEILARDGPNAL   81
a1.XP_024144684.1      KG-K-KKEKDMDELKKEVDMDDHKLTLDELSRKYGTDLSN-GLTGAKSAEILARDGPNAL   81
a1.sp|P25489.1|        KNKKEKKEKDMDELKKEVDLDDHKLSLEELHRKYGTDLSK-GLSNSRAEEILARDGPNAL   83
a1.XP_022536277.1      KNK--KKDKDMDELKKEVDLDDHKLSLDELHRKYGTDLSR-GLSSSRAQEILARDGPNAL   81
a1.Q9DEU1              KNK--KKEKDMDELKKEVDLDDHKLTLEELNRKYGTDLNR-GLTTARAAEILARDGPNAL   81
a1.sp|Q92030.1|        ---RDKKKKDMDDLKKEVDLDDHKLTLDELHRKYGTDLTR-GLTSSRAAEILARDGPNAL   79
a1.AJR20271.1          KGKK--EEKDMDDLKKEVDLDDHKLTLDELYRKYGSDLNR-GLTASRAKEILARDGPNAL   97
a1.Q90X33              KGKK--KQKDMDELKKEVDLDDHKLTLDELHRKYGTDLTR-GLSSSRAKEVLARDGPNAL   81
a1.XP_004066575.1      K----DKKTDMDDLKKEVDLDDHKLTLEELFRKYGTDGNR-GLSSSRAKEILARDGPNAL   79
a1.XP_024144685.1      K----NKQKDMDELKKEVDLDDHKLTLEELFRKYGTDANR-GLSSSRAKEILARDGPNAL   79
a1.XP_020476182.1      KGKKAKEKKDMEELKKEVDLDDHKLTMEELHRKYETDLTR-GLTASKAKEILARDGPNAL   83
a1.UPI000443A733       KKKKGKQKKDMDELKKEVDLDDHKLTLDELHRKYGTDLSR-GLSASRAKDILARDGPNAL   80
a1.XP_023185631.1      KHKKGKQKKDMDELKKEVDLDDHKLTLDELHRKYGTDLSR-GLSASRAKEILARDGPNAL   80
a1.sp|Q9YH26.2|        KDKKAKAKKDMDDLKKEVDLDDHKLTLDELHRKYGTDLTR-GLSSSRAKEILARDGPNAL   80
a1.UPI00022B0848       KDKKAKAKKDMDDLKKEVDLDDHKLTLDELHRKYGTDLTR-GLSSSRAKEILARDGPNAL   80
a1.XP_020792263.1      KAKKAKEKKDMDDLKKEVDLDDHKLTLDELHRKYGTDLTR-GLSGSRAKEILARDGPNAL   81
a1.AKQ12834.1          KGKKGKSEKDMADLKKEVDLDDHKLTLDELHRKYGTDLSR-GLSNARAKEILDRDGPNAL   82
a1.XP_023275950.1      KKGKG-PKKDMDDLKKEVDLDDHKLTLDELHRKYGTDLSR-GLSTSRAKEILARDGPNAL   81
a1.EMP33651.1          ------------------------------------------------------------   0
a1.XP_004853865.1      -KA--KKERDMDELKKEVSMDDHKLSLDELHRKYGTDLSR-GLTSARAAEILARDGPNAL   80
a1.ACB20771.2          -KA--KKDRDMDELKKEVSMDDHKLSLDELHRKYGTDLSR-GLTSARAAEILARDGPNSL   80
```

a1.XP_023557491.1	----------MDELKKEVSMDDHKLSLDELHRKYGTDLSR-GLTSARAAEILARDGPNSL	49
a1.AAA41671.1	-KA--KKERDMDELKKEVSMDDHKLSLDELHRKYGTDLSR-GLTPARPVEILARDGPNAL	80
a1.XP_005076578.1	-KA--KKERDMDELKKEVSMDDHKLSLDELHRKYGTDLSR-GLTPARAAEILARDGPNAL	80
a1.XP_021504168.1	-KA--KKERDMDELKKEVSMDDHKLSLDELHRKYGTDLSR-GLTPARAAEILARDGPNAL	80
a1.XP_021051287.1	-KA--KKERDMDELKKEVSMDDHKLSLDELHRKYGTDLSR-GLTPARAAEILARDGPNAL	80
a1.XP_021013125.1	-KA--KKERDMDELKKEVSMDDHKLSLDELHRKYGTDLSR-GLTPARAAEILARDGPNAL	80
a1.NP_659149.	-KA--KKERDMDELKKEVSMDDHKLSLDELHRKYGTDLSR-GLTPARAAEILARDGPNAL	80
a1.UPI000C2F2801	-KG--KKDRDMDELKKEVSMDDHKLSLDELHRKYGTDLSR-GLTSARAAEILARDGPNAL	84
a1.XP_020858281.1	-KA--KKEKDMDELKKEVSMEDHKLSLDELHRKYGTDLSR-GLTTARAAEILARDGPNAL	78
a1.XP_004380410.1	-KA--KKERDMDELKKEVSMDDHKLSLDELHRKYGTDLSR-GLTTARAAEILARDGPNAL	78
a1.UPI0001FB338F	-KA--KKERDMDELKKEVSMDDHKLSLDELQRKYGTDLSR-GLTTARAAEILARDGPNAL	78
a1.UPI0002B3D77C	-KA--KKERDMDELKKEVSMDDHKLSLDELHRKYGTDLSR-GLTTARAAEILARDGPNAL	74
a1.UPI0000124FBE	-KA--KKERDMDELKKEVSMDDHKLSLDELHRKYGTDLNR-GLTTARAAEILARDGPNAL	78
a1.XP_020747989.1	-KA--KKERDMDELKKEVSMDDHKLSLDELHRKYGTDLNR-GLTTARAAEILARDGPNAL	78
a1.XP_010587900.1	----------MDELKKEVTMDDHKLSLDELHRKYGTDLSR-GLTSARAAEILARDGPNAL	49
a1.XP_020012504.1	-KA--KKERDMDELKKEVSMDDHKLSLDELHRKYGTDLTR-GLTTARAAEILARDGPNAL	80
a1.XP_005334975.1	-KA--KKERDMDELKKEVSMDDHKLSLDELHRKYGTDLSR-GLTTARAAEILARDGPNAL	80
a1.XP_024426171.1	-KV--KKERDMDELKKEVSMDDHKLSLDELHRKYGTDLNR-GLTSARAAEILARDGPNAL	78
a1.XP_023975434.1	-KA--KKERDMDELKKEVSMDDHKLSLDELHRKYGTDLSR-GLTPARAAEILARDGPNAL	78
a1.XP_022439684.1	-KA--KKERDMDELKKEVSMDDHKLSLDELHRKYGTDLSR-GLTTARAAEILARDGPNAL	78
a1.XP_024620662.1	-KA--KKERDMDELKKEVSMDDHKLSLDELHRKYGTDLSR-GLTTARAAEILARDGPNAL	78
a1.XP_011283388.1	-KA--KKERDMDELKKEVSMDDHKLSLDELHRKYGTDLSR-GLTTARAAEILARDGPNAL	78
a1.XP_006919736.1	----------MDELKKEVSMDDHKLSLDELHRKYGTDLSR-GLTSARAAEILARDGPNAL	49
a1.UPI000C746E0C	-KG--KKDRDMDELKKEVSMDDHKLSLDELHRKYGTDLSR-GLTSARAAEILARDGPNAL	91
a1.UPI000C2E744C	-KG--KKDRDMDELKKEVSMDDHKLSLDELHRKYGTDLSR-GLTSARAAEILARDGPNAL	84
a1.XP_020944376.1	-KA--KKERDMDELKKEVSMDDHKLSLDELHRKYGTDLSR-GLTPARAAEILARDGPNAL	78
a1.XP_008071711.2	-KA--KKDRDMDELKKEVTMDDHKLSLDELHRKYGTDLSR-GLTSARAAEILARDGPNAL	85
a1.UPI000C2E4C26	-KG--KKDRDMDELKKEVSMDDHKLSLDELHRKYGTDLSR-GLTSARAAEILARDGPNAL	76
a1.XP_012663099.1	-KA--KKDRDMDELKKEVTMDDHKLSLDELHRKYGTDLSR-GLTSARAAEILARDGPNAL	80
a1.XP_012617266.1	-KA--KKERDMDELKKEVSMDDHKLSLDELHRKYGTDLSR-GLTSARAAEILARDGPNAL	80
a1.XP_012314296.1	-KV--KKERDMDELKKEVSMDDHKLSLDELHRKYGTDLSR-GLTSARAAEILARDGPNAL	80
a1.UPI0001C9F9BA	-KV--KKERDMDELKKEVSMDDHKLSLDELHRKYGTDLSR-GLTSARAAEILARDGPNAL	80
a1.XP_023078532.1	-KG--KKDRDMDELKKEVSMDDHKLSLDELHRKYGTDLSR-GLTSARAAEILARDGPNAL	80
a1.NP_000692	-KG--KKDRDMDELKKEVSMDDHKLSLDELHRKYGTDLSR-GLTSARAAEILARDGPNAL	80
a1.XP_008971666.1	----------MDELKKEVSMDDHKLSLDELHRKYGTDLSR-GLTSARAAEILARDGPNAL	49
a1.XP_016780478.1	-KG--KKDRDMDELKKEVSMDDHKLSLDELHRKYGTDLSR-GLTSARAAEILARDGPNAL	80
a1.ETE67008.1	GKK----EKDMDELKKEVSLDDHKLSLDELHRKYGTDLSR-GLSVARAAEILARDGPNAL	79
a1.XP_007435355.1	----------MDELKKEVSLDDHKLSLDELHRKYGTDLSR-GLTVARAAEILARDGPNAL	49
a1.XP_020645227.1	----------MEELKKEVSLDDHKLSLDELHRKYGTDLSR-GLTTQRAAEILARDGPNAL	49
a1.XP_025067531.1	----------MDELKKEVSMDDHKLSLDEVHRKYGTDLSR-GLTPARAAEILARDGPNSL	49
a1.XP_005292736.1	GKK--EKKKDMDELKKEVSMDDHKLSLDELHRKYGTDLSR-GLTTARAAEILARDGPNAL	81
a1.XP_006132947.1	----------MDELKKEVSLDDHKLSLDELHRKYGTDLSR-GLTVARAAEILARDGPNAL	49
a1.NP_990852.	--K--AKERDMDELKKEISMDDHKLSLDELHRKYGTDLSR-GLTTARAAEILARDGPNTL	78
a1.XP_021253236.1	--K--AKERDMDELKKEVSMDDHKLSLDELHRKYGTDLNR-GLTTARAAEILARDGPNTL	78
a1.UPI00051ECCC0	--K--KKERDMDELKKEVTMDDHKLSLDELHRKYGTDLSR-GLTTARAAEILARDGPNAL	73
a1.XP_023796730.1	----------MDELKKEVVMDDHKLSLDELHRKYGTDLSR-GLTSARAAEILARDGPNAL	49
a1.XP_021404823.1	-KG--KKERDMDELKKEVVMDDHKLSLDELHRKYGTDLSR-GLTTARAAEILARDGPNAL	78
a1.UPI0004FDA0CB	-KG--KKERDMDELKKEVSMDDHKLSLDELHRKYGTDLSR-GLTSARAAEILARDGPNSL	76
a1.KFW61640.1	GK----KERDMDELKKEVSMDDHKLSLDELHRKYGTDLSR-GLTPARAAEILARDGPNSL	75
a1.XP_005511501.1	----------MDELKKEVSMDDHKLSLDELHRKYGTDLNR-GLTAARAAEILARDGPNSL	49
a1.OPJ66608.1	GKK--KKERDMDELKKEVSMDDHKLSLDELHRKYGTDLSR-GLTTARAAEILARDGPNSL	81
a1.sp|P30714.2|	GK---GKDRDMEELKKEVTMEDHKMTLEELHRKYGTDLTR-GLTTARAAEILARDGPNAL	80
a1.NP_989407.1	GK---GKDKDMDELKKELTMEDHKLSLDELHRKFGTDMQR-GLSTARAAEILARDGPNAL	80
a2.UPI0000F6BCEB	GGKKKQKEKEMDELKKEVAMDDHKLSMEEIGRKYGTDLAK-GLTNARAAEVLAQDGPNAL	78
a2.UPI000226F4AA	GGKKKQKEKELDELKKEVAMDDHKLSLDELGRKYQVDLSK-GLTNQRAQDILARDGPNAL	78
a2.UPI00005E9366	GGKKKQKEKELDELKKEVAMDDHKLSLDELGRKYQVDLSK-GLTNQRAQDILARDGPNAL	78
a2.XP_020835237.1	GGKKKQKEKELDELKKEVAMDDHKLSLDELGRKYQVDLSK-GLTNQRAQDILARDGPNAL	78
a2.XP_023616468.1	GGKKKQKERELDELKKEVAMDDHKLSLDELGRKYQVDLSK-GLTNQRAQDILARDGPNAL	78
a2.XP_021537506.1	GGKKKQKEKELDELKKEVAMDDHKLSLDELGRKYQVDLSK-GLTNQRAQDILARDGPNAL	139
a2.UPI0002B2E326	GGKKKQKEKELDELKKEVAMDDHKLSLDELGRKYQVDLSK-GLTNQRAQDILARDGPNAL	71
a2.XP_020024807.1	GGKKKQKEKELDELKKEVTMDDHKLSLDELGRKYQVDLSK-GLTNQRAQDILARDGPNAL	78
a2.NP_036637.	GGKKKQKEKELDELKKEVAMDDHKLSLDELGRKYQVDLSK-GLTNQRAQDILARDGPNAL	78
a2.XP_003415228.1	GGKKKQKEKELDELKKEVAMDDHKLSLDELGRKYQVDLSK-GLTNQRAQDILARDGPNAL	78
a2.XP_004390257.1	GGKKKQKEKELDELKKEVAMDDHKLSLDELGRKYQVDLSK-GLTNQRAQDVLARDGPNAL	78
a2.XP_004448489.1	GGKKKQKEKELDELKKEVAMDDHKLSLDELGRKYQVDLSK-GLTNQRAQDILARDGPNAL	106
a2.XP_020740887.1	GGKKKQKEKELDELKKEVAMDDHKLSLDELGRKYQVDLSK-GLTNQRAQDILARDGPNAL	78
a2.XP_022415031.1	GGKKKQKEKELDELKKEVAMDDHKLSLDELGRKYQVDLSK-GLTNQRAQDILARDGPNAL	78
a2.XP_007129684.1	GGKKKQKEKELDELKKEVAMDDHKLSLDELGRKYQVDLSK-GLTNQRAQDILARDGPNAL	78
a2.XP_004858786.1	GGKKKQKEKELDELKKEVAMDDHKLSLDELGRKYQVDLSK-GLTNQRAQDILARDGPNAL	78
a2.XP_003466610.1	GGKKKQKEKELDELKKEVAMDDHKLSLDELGRKYQVDLSK-GLTNQRAQDILARDGPNAL	78
a2.XP_004639996.1	GGKKKQKEKELDELKKEVAMDDHKLSLDELGRKYQVDLSK-GLTNQRAQDILARDGPNAL	78

```
a2.XP_005339432.1      GGKKKQKEKELDELKKEVAMDDHKLSLDELGRKYQVDLSK-GLTNQRAQDILARDGPNAL    78
a2.XP_545753.3so       GGKKKQKEKELDELKKEVAMDDHKLSLDELGRKYQVDLSK-GLTNQRAQDILARDGPNAL    78
a2.XP_019677883.4      GGKKKQKEKELDELKKEVAMDDHKLSLDELGRKYQVDLSK-GLTNQRAQDILARDGPNAL    78
a2.XP_023069989.1      GGKKKQKEKELDELKK--------------------------------------------    35
a2.XP_003795245.1      GGKKKQKEKELDELKKEVTMDDHKLSLDELGRKYQVDLSK-GLTNQRAQDILVRDGPNAL    78
a2.NP_000693.          GGKKKQKEKELDELKKEVAMDDHKLSLDELGRKYQVDLSK-GLTNQRAQDVLARDGPNAL    78
a2.XP_008056914.2      GGKKKQKEKELDELKKEVAMDDHKLSLDELGRKYQVDLSK-GLTNQRAQDILARDGPNAL    78
a2.XP_012604635.1      GGKKKQKEKELDELKKEVAMDDHKLSLDELGRKYQVDLSK-GLTNQRAQDILARDGPNAL    78
a2.XP_020653823.1      GGKKKQKEKELDELKKEVNLDDHKLSLDEISRKYQVDLSK-GLTNTRAAEILAKDGPNAL    78
a2.UPI0000124FC0       GGRRKQKEKELDELKKEVNLDDHKLSLDELGRKYQVDLSR-GLSNARAAEVLAQDGPNAL    75
a2.XP_005293820.1      GGKKKQKEKELDELKKEVNLDDHRLSLDELGRKYQVDLSR-GLTNTRAAEILAQDGPNAL    78
a1.KYO43368.1          GGKKKQKEKELDELKKEVNLNDHRLSLDEVGRKYEVDLSR-GLTNARAAESLAQHGPNAL    84
a2.XP_006038189.1      GGKKKQKEKELDELKKEVNLNDHRLSLDEVGRKYEVDLSR-GLTNAQAAESLAQHGPNAL    78
a2.NP_571758.1         GGKRKKKDKDLDELKKEVSLDDHKLTLDELSTRYGVDLAR-GLTHKRAMEILARDGPNAL    75
a2.UPI00000FE1CF       EGKKNKKERDLDELKKEVALDDHKITLDELGKRYGVDLTR-GLTNARAAEILARDGPNAL    66
a2.XP_020507674.1      ------------------------------------------------------------    0
a2.BAO02373.1          GGKRRKKDRDLDELKKEVALDDHKIALDDLGKRYGVDLTR-GLTNARAAEILARDGPNSL    68
```
