# Supplementary material for: Bioinformatics approaches for classification and investigation of the evolution of the Na/K-ATPase alpha-subunit
Source: BMC Ecol Evol. 2022 Oct 26;22:122. doi: 10.1186/s12862-022-02071-0 (PMC9609216; doi:10.1186/s12862-022-02071-0)
Supplement: Supplementary file 1 — Additional file 1. Supplementary figures and tables. [file 12862_2022_2071_MOESM1_ESM.zip › Additional file 1 Figs..docx]

**Supplementary Information**

Additional file 1 Fig. S1: The phylogenetic tree of Na,K-ATPases for all organisms in compressed rectangular shape to show branch lengths and node support values. Different symbols and colors were used to distinguish organisms and the type of isoforms. The scale indicates the number of amino acid substitutions per site.

Additional file 1 Fig. S2: The phylogenetic tree of different pumps in fungal sequences in compressed rectangular shape to show branch lengths and node support values. Different colors were used to distinguish different kind of pumps.

Additional file 1 Fig. S3: The phylogenetic tree for 378 sequences of ssu rRNA (16S/18S rRNA) from various organisms of three life domains which were used for construct NAK phylogenetic tree in compressed rectangular shape to show branch lengths and node support values. Different symbols and colors were used to distinguish organisms and the type of isoforms. The scale indicates the number of amino acid substitutions per site.

Additional file 1 Fig. S4: The phylogenetic tree of Na,K-ATPase for vertebrate organisms in compressed rectangular shape to show branch lengths and node support values. Different symbols and colors were used to distinguish organisms and the type of isoforms. The hollow shapes indicated the isoform was predicted by the phylogenetic tree in this study. Circle, triangle, square and diamond shapes were used to show α1, α2, α3 and α4, respectively. The scale indicates the number of amino acid substitutions per site.

Additional file 1 Fig. S5: The alignment of α1, α2, α3 isoforms along with KYO43368.1 to determine the type of ‎KYO43368.1. The isoform-specific regions within α-subunit of Na,K-ATPase were underlined. ‎

Additional file 1 Fig. S6: Alignment of 753 Na/K ATPase sequences to determine the position of the dipeptide ^142^GC in seven group of organisms. The invertebrate sequences belonging to groups 1 and 2 of the phylogenetic tree are marked with the group I and II in the accession number, respectively.

Art: Arthropod, Fun: Fungus, Nem: Nematod, Prk: Prokaryot, Prt: Protist, Rinv: Rest of invertebrate, Ver: Vertebrate

Additional file 1 Fig. S7: Alignment of 753 Na/K ATPase sequences to determine the position of the dipeptide ^208^GC in seven group of organisms. The invertebrate sequences belonging to groups 1 and 2 of the phylogenetic tree are marked with the group I and II in the accession number, respectively.

Art: Arthropod, Fun: Fungus, Nem: Nematod, Prk: Prokaryot, Prt: Protist, Rinv: Rest of invertebrate, Ver: Vertebrate

Additional file 1 Fig. S8: Alignment of 753 Na/K ATPase sequences to determine the position of the dipeptide ^702^GC in seven group of organisms. The invertebrate sequences belonging to groups 1 and 2 of the phylogenetic tree are marked with the group I and II in the accession number, respectively.

Art: Arthropod, Fun: Fungus, Nem: Nematod, Prk: Prokaryot, Prt: Protist, Rinv: Rest of invertebrate, Ver: Vertebrate

Additional file 1 Fig. S9: Alignment of 231 Na/K ATPase sequences to determine the position of the dipeptide ^41^DH in four vertebrate isoforms. The sequences belonging to type of isoforms are marked with the α_1_, α_2_, α_3_ and α_4_ in the accession number, respectively.

Additional file 1 Fig. S10: Alignment of 231 Na/K ATPase sequences to determine the position of the dipeptide ^431^FK and ^451^KC in four vertebrate isoforms. The sequences belonging to type of isoforms are marked with the α_1_, α_2_, α_3_ and α_4_ in the accession number, respectively.
