# Supplementary material for: Bioinformatics approaches for classification and investigation of the evolution of the Na/K-ATPase alpha-subunit
Source: BMC Ecol Evol. 2022 Oct 26;22:122. doi: 10.1186/s12862-022-02071-0 (PMC9609216; doi:10.1186/s12862-022-02071-0)
Supplement: Supplementary file 1 — Additional file 1. Supplementary figures and tables. [file 12862_2022_2071_MOESM1_ESM.zip › Additional file 1 Table. S1.docx]

| Table S1: The similarity rate of different isoforms within and among different groups of vertebrates | | | | | | | | | | | | | | | | | | |
| --- | --- | --- | --- | --- | --- | --- | --- | --- | --- | --- | --- | --- | --- | --- | --- | --- | --- | --- |
| Organism | Isoform |  | α1 | | | | | α2 | | | | | α3 | | | | | |
|  |  | no. | 1 | 2 | 3 | 4 | 5 | 1 | 2 | 3 | 4 | 5 | | 1 | 2 | 3 | 4 | 5 |
| Amphibious | α1 | 1 | 95.23 | 91.52 | 86.79 | 91.03 | 91.60 | 86.56 | 86.87 | 84.57 | 86.84 | 86.74 | | 84.81 | 86.82 | 85.54 | 85.94 | 85.29 |
| Bird |  | 2 | 91.52 | 95.23 | 87.16 | 92.77 | 93.46 | 86.96 | 87.27 | 84.49 | 87.49 | 87.35 | | 85.24 | 87.48 | 85.44 | 86.50 | 95.61 |
| Fish |  | 3 | 86.79 | 87.16 | 88.72 | 86.51 | 86.78 | 83.94 | 83.95 | 82.77 | 83.94 | 84.16 | | 82.72 | 84.67 | 83.23 | 83.35 | 82.58 |
| Mammalia |  | 4 | 91.03 | 92.77 | 86.51 | 95.92 | 92.16 | 85.84 | 86.24 | 83.96 | 84.84 | 85.98 | | 84.38 | 86.64 | 84.70 | 82.08 | 84.95 |
| Reptile |  | 5 | 91.60 | 93.46 | 86.78 | 92.16 | 93.47 | 84.95 | 86.78 | 84.71 | 85.87 | 88.30 | | 85.72 | 87.45 | 85.40 | 86.44 | 86.39 |
| Amphibious | α2 | 1 | 86.56 | 86.96 | 83.94 | 85.84 | 84.95 | 96.76 | 91.35 | 85.47 | 91.84 | 91.37 | | 84.66 | 86.27 | 84.38 | 85.74 | 85.10 |
| Bird |  | 2 | 86.87 | 87.27 | 83.95 | 86.24 | 86.78 | 91.35 | 100.00 | 85.41 | 93.37 | 94.30 | | 84.94 | 87.08 | 84.80 | 84.06 | 85.58 |
| Fish |  | 3 | 84.57 | 84.49 | 82.77 | 83.96 | 84.71 | 85.47 | 85.41 | 89.42 | 85.56 | 85.78 | | 82.42 | 83.98 | 82.95 | 83.32 | 82.95 |
| Mammalia |  | 4 | 86.84 | 87.49 | 83.94 | 84.84 | 85.87 | 91.84 | 93.37 | 85.56 | 97.65 | 92.71 | | 85.69 | 87.54 | 84.57 | 86.90 | 85.78 |
| Reptile |  | 5 | 86.74 | 87.35 | 84.16 | 85.98 | 88.30 | 91.37 | 94.30 | 85.78 | 92.71 | 95.15 | | 84.95 | 86.78 | 84.71 | 85.87 | 85.63 |
| Amphibious | α3 | 1 | 84.81 | 85.24 | 82.72 | 84.38 | 85.72 | 84.66 | 84.94 | 82.42 | 85.69 | 84.95 | | 95.51 | 92.67 | 89.29 | 92.11 | 91.79 |
| Bird |  | 2 | 86.82 | 87.48 | 84.67 | 86.64 | 87.45 | 86.27 | 87.08 | 83.98 | 87.54 | 86.78 | | 92.67 | 100.00 | 89.87 | 95.58 | 95.61 |
| Fish |  | 3 | 85.54 | 85.44 | 83.23 | 84.70 | 85.40 | 84.38 | 84.80 | 82.95 | 84.57 | 84.71 | | 89.29 | 89.87 | 93.27 | 89.15 | 87.16 |
| Mammalia |  | 4 | 85.94 | 86.50 | 83.35 | 82.08 | 86.44 | 85.74 | 84.06 | 83.32 | 86.90 | 85.87 | | 92.11 | 95.58 | 89.15 | 97.61 | 95.44 |
| Reptile |  | 5 | 85.29 | 95.61 | 82.58 | 84.95 | 86.39 | 85.10 | 85.58 | 82.95 | 85.78 | 85.63 | | 91.79 | 95.61 | 87.16 | 95.44 | 94.97 |
