# Supplementary material for: Bioinformatics approaches for classification and investigation of the evolution of the Na/K-ATPase alpha-subunit
Source: BMC Ecol Evol. 2022 Oct 26;22:122. doi: 10.1186/s12862-022-02071-0 (PMC9609216; doi:10.1186/s12862-022-02071-0)
Supplement: Supplementary file 1 — Additional file 1. Supplementary figures and tables. [file 12862_2022_2071_MOESM1_ESM.zip › Additional file 1 Table. S2.docx]

Table S2: More effective attributes in create decision tree for α-NKA protein in different organism groups based on weighting method

| Weighting method | Attribute | Weight | Weighting method | Attribute | Weight |
| --- | --- | --- | --- | --- | --- |
| Chi-square | aa length | 1.00 |  | Ile/Pro ratio | 0.83 |
|  | Cys count | 0.96 |  | Asn/Gly ratio | 0.82 |
|  | Ala/Cys ratio | 0.93 |  | Ala/GLU ratio | 0.82 |
|  | Cys/Tyr ratio | 0.85 |  |  |  |
|  | Hydrophilic count | 0.85 | Gini index | Gly-Cys count | 1.00 |
|  | Cys/His ratio | 0.82 |  | Cys-Phe count | 0.97 |
|  | Tyr/Cys ratio | 0.79 |  | Arg-Phe count | 0.87 |
|  | Asn-Cys count | 0.75 |  | Cys/Tyr ratio | 0.79 |
|  | Phe-Asn count | 0.75 |  | Tyr/Cys ratio | 0.78 |
|  | Cys/Met ratio | 0.74 |  | Phe-Cys count | 0.77 |
|  | Cys-Ile count | 0.73 |  | Cys-His count | 0.72 |
|  | Cys-Phe count | 0.73 |  | Asn-Phe count | 0.69 |
|  | Phe-Cys count | 0.73 |  | Thr-Trp count | 0.69 |
|  | Aliphatic index | 0.72 |  | Asn-Cys count | 0.68 |
|  | Asp/Cys ratio | 0.71 |  | Asp/Cys ratio | 0.66 |
|  |  |  |  | Cys count | 0.66 |
| info gain | Gly-Cys count | 1.00 |  | Cys/Met ratio | 0.65 |
|  | Cys-Phe count | 0.96 |  |  |  |
|  | Arg-Phe count | 0.80 | info gain ratio | Ala/Cys ratio | 1.00 |
|  | Asn-Cys count | 0.77 |  | Cys count | 0.97 |
|  | Phe-Cys count | 0.76 |  | Cys/His ratio | 0.89 |
|  | Cys/Tyr ratio | 0.75 |  | His/Cys ratio | 0.88 |
|  | Tyr/Cys ratio | 0.74 |  | Gly-Cys count | 0.87 |
|  | Asp/Cys ratio | 0.73 |  | Cys/Tyr ratio | 0.87 |
|  | Thr-Trp count | 0.69 |  | Aliphatic index | 0.86 |
|  | Ala/Cys ratio | 0.69 |  | Leu percentage | 0.86 |
|  | Cys count | 0.66 |  | Tyr/Cys ratio | 0.85 |
|  | Cys-His count | 0.66 |  | Ser/Trp ratio | 0.84 |
|  | Cys/Met ratio | 0.65 |  | Cys-Phe count | 0.83 |
|  | Asn-Phe count | 0.64 |  | Cys/Lys ratio | 0.82 |
|  | Cys-Val count | 0.63 |  | Cys/Asn ratio | 0.81 |
|  | Cys/Asn ratio | 0.59 |  | Leu/Asp ratio | 0.81 |
|  |  |  |  | Ile/Cys ratio | 0.80 |
| deviation | Asp percentage | 1.00 |  | Cys/Gln ratio | 0.80 |
|  | Glu percentage | 0.97 |  | Asp count | 0.80 |
|  | Leu/Gly ratio | 0.94 |  | Hydrophilic count | 0.79 |
|  | Ile/Asn ratio | 0.91 |  | Ala/Trp ratio | 0.79 |
|  | Asp/Asn ratio | 0.89 |  | Gln/Cys ratio | 0.79 |
|  | Ile/Val ratio | 0.87 |  | Asp/Cys ratio | 0.78 |
|  | Val/Gly ratio | 0.85 |  |  |  |
|  | Ala/Gly ratio | 0.84 | PCA | Ala/Cys ratio | 1.00 |
|  | Ile-Thr count | 0.87 |  | Ala/Asp ratio | 0.98 |
|  | His-Glu count | 0.85 |  | Asp/Asn ratio | 0.27 |
| Weighting method | Attribute | Weight | Weighting method | Attribute | Weight |
|  | Phe-Cys count | 0.92 |  | Ala/Cys ratio | 0.97 |
|  | Cys count | 0.90 |  | Gly-Cys count | 0.93 |
|  | Ile/Leu ratio | 0.89 |  | Cys-Phe count | 0.86 |
|  | Ala/Ile ratio | 0.87 |  | Phe-Cys count | 0.85 |
|  | Phe-Ala count | 0.87 |  | Cys count | 0.84 |
|  | His/Cys ratio | 0.87 |  | Cys/Tyr ratio | 0.81 |
|  | Leu/Ile ratio | 0.87 |  | Tyr/Cys ratio | 0.81 |
|  | Pro/Asn ratio | 0.86 |  | Cys-Ile count | 0.77 |
|  | Ala/Glu ratio | 0.86 |  | Cys/Met ratio | 0.73 |
|  | Ala/Gly ratio | 0.86 |  | Asp/Cys ratio | 0.72 |
|  | Leu/Glu ratio | 0.85 |  | Arg-Phe count | 0.72 |
|  | Asp/Pro ratio | 0.83 |  | Trp-Phe count | 0.70 |
|  | Gln/Cys ratio | 0.83 |  | aa length | 0.70 |
|  | Ala percentage | 0.83 |  | Phe-Asn count | 0.68 |
|  | Ala-Trp count | 0.83 |  | Cys-His count | 0.68 |
|  | Ala/Asn ratio | 0.82 |  | Asn-Phe count | 0.67 |
|  | Asn-Cys count | 0.82 |  | Tyr-Asp count | 0.66 |
|  | Val-Leu count | 0.81 |  | Gln/Cys ratio | 0.64 |
|  | Pro/Ile ratio | 0.81 |  | Cys/His ratio | 0.64 |
|  | Cys/Met ratio | 0.81 |  | Thr-Trp count | 0.64 |
|  |  |  |  | Ile/Cys ratio | 0.63 |
| relief | Cys-Asp count | 1.00 |  |  |  |
|  | Asn-Cys count | 0.97 | SVM | Cys-Lys count | 1.00 |
|  | Thr-Trp count | 0.96 |  | Cys-Asp count | 0.96 |
|  | Gly-Cys count | 0.91 |  | Cys-Phe count | 0.96 |
|  | Cys-His count | 0.89 |  | Glu-Cys count | 0.88 |
|  | Arg-Phe count | 0.87 |  | Gly-Cys count | 0.83 |
|  | Cys-Phe count | 0.83 |  | Phe-Cys count | 0.82 |
|  | Cys-Val count | 0.74 |  | Glu-Trp count | 0.81 |
|  | Phe-Cys count | 0.72 |  | Asp-Ile count | 0.80 |
|  | Tyr-His count | 0.72 |  | Pro-Tyr count | 0.80 |
|  | Trp-Phe count | 0.72 |  | Asp-Pro count | 0.77 |
|  |  |  |  | Arg-Phe count | 0.75 |
| Rule | Aliphatic index | 1.00 |  | His-Lys count | 0.74 |
|  | Glu percentage | 0.94 |  | Trp-Phe count | 0.72 |
|  | Ala percentage | 0.86 |  | Trp-Pro count | 0.72 |
|  | Val percentage | 0.84 |  | Gln-Tyr count | 0.70 |
|  | Ser percentage | 0.82 |  |  |  |
|  | Pro percentage | 0.81 |  |  |  |
|  | Ala/Cys ratio | 0.77 |  |  |  |
|  | Met percentage | 0.75 |  |  |  |
|  | Thr percentage | 0.75 |  |  |  |
|  |  |  |  |  |  |
| uncertainty | Asn-Cys count | 1.00 |  |  |  |
