# Supplementary material for: Bioinformatics approaches for classification and investigation of the evolution of the Na/K-ATPase alpha-subunit
Source: BMC Ecol Evol. 2022 Oct 26;22:122. doi: 10.1186/s12862-022-02071-0 (PMC9609216; doi:10.1186/s12862-022-02071-0)
Supplement: Supplementary file 1 — Additional file 1. Supplementary figures and tables. [file 12862_2022_2071_MOESM1_ESM.zip › Additional file 1 Table. S3.docx]

Table S3: More effective attributes in create decision tree for α-NKA protein in different isoforms of vertebrates based on weighting method

| Weighting method | Attribute | Weight | Weighting method | Attribute | Weight |
| --- | --- | --- | --- | --- | --- |
| Chi-square | Phe-Lys count | 1.00 |  | Phe-Pro count | 0.83 |
|  | Leu/Cys ratio | 0.86 |  | Cys-Cys count | 0.82 |
|  | His-Glu count | 0.81 |  | Gln-Ile count | 0.82 |
|  | Leu/Asp ratio | 0.81 |  | Pro-Asp count | 0.81 |
|  | Pro-Asp count | 0.77 |  | Thr-His count | 0.81 |
|  | Leu-Asp count | 0.76 |  | Glu-Asn count | 0.80 |
|  | Ser/Cys ratio | 0.74 |  | Asp-Cys count | 0.79 |
|  | Asp/Lys ratio | 0.74 |  | Thr-Ile count | 0.79 |
|  | Arg-Leu count | 0.74 |  | Asp-Asn count | 0.78 |
|  | Ile-Thr count | 0.73 |  | Val-Asp count | 0.77 |
|  | Cys count | 0.73 |  | Ala-Asn count | 0.76 |
|  | Lys-Val count | 0.71 |  | Asn/Cys ratio | 0.74 |
|  | Cys-Phe count | 0.69 |  | Ser-Tyr count | 0.73 |
|  | Glu-Leu count | 0.69 |  | His-Lys count | 0.72 |
|  | Asp count | 0.68 |  | Ala-Ile count | 0.72 |
|  | Ile/Cys ratio | 0.68 |  | Glu-Leu count | 0.71 |
|  | Asn-Glu count | 0.68 |  | Val-Cys count | 0.70 |
|  | Thr-Glu count | 0.67 |  |  |  |
|  | Asn-Gln count | 0.67 | deviation | Aliphatic index | 1.00 |
|  | Lys-Thr count | 0.67 |  | Leu/Pro ratio | 0.42 |
|  | Phe/Cys ratio | 0.66 |  | Val percentage | 0.41 |
|  | Gln-Pro count | 0.65 |  | Gly/Arg ratio | 0.40 |
|  | Lys/Cys ratio | 0.65 |  | Ala/Asp ratio | 0.34 |
|  | Gly/Cys ratio | 0.64 |  | Gly percentage | 0.33 |
|  | Asp/Phe ratio | 0.64 |  | Pro/Trp ratio | 0.33 |
|  | Asn/Cys ratio | 0.64 |  | Ile/Met ratio | 0.32 |
|  | Val-Asn count | 0.62 |  | Isoelectric point | 0.32 |
|  | Cys/Met ratio | 0.62 |  | Pro/Tyr ratio | 0.31 |
|  | Asp/Ser ratio | 0.62 |  | Glu/Phe ratio | 0.31 |
|  | Tee/Cys ratio | 0.62 |  | Phe/Tyr ratio | 0.30 |
|  | Ile/Asp ratio | 0.61 |  | Val/Lys ratio | 0.29 |
|  | Ile/Arg ratio | 0.61 |  | Ala/Cys ratio | 0.29 |
|  | Lys-Arg count | 0.61 |  | Asp/Tyr ratio | 0.29 |
|  |  |  |  | Leu percentage | 0.28 |
| info gain | Asp-His count | 1.00 |  | Ala/Lys ratio | 0.28 |
|  | Phe-Lys count | 0.98 |  | Glu/Lys ratio | 0.28 |
|  | Cys-Arg count | 0.95 |  | His/Try ratio | 0.28 |
|  | His-Asp count | 0.91 |  | Pro/Met ratio | 0.28 |
|  | Leu-Asp count | 0.90 |  | Glu/Met ratio | 0.28 |
|  | Glu-Asp count | 0.89 |  | Ser/Lys ratio | 0.28 |
|  | Leu/Cys ratio | 0.89 |  | Asp/Glu ratio | 0.27 |
|  | Ile-Thr count | 0.87 |  | Tyr/His ratio | 0.27 |
|  | His-Glu count | 0.85 |  | Asp/Asn ratio | 0.27 |
| Weighting method | Attribute | Weight | Weighting method | Attribute | Weight |
|  | Leu/Lys ratio | 0.27 |  | Ser/Cys ratio | 0.98 |
|  | Ala/His ratio | 0.27 |  | Thr/Cys ratio | 0.98 |
|  | Ala/Thr ratio | 0.27 |  | Lys/Cys ratio | 0.96 |
|  | Lys/Tyr ratio | 0.26 |  | Leu/Cys ratio | 0.96 |
|  | Ala/Met ratio | 0.26 |  | Asp-His count | 0.90 |
|  | Ser/Cys ratio | 0.26 |  | Leu/Ala ratio | 0.89 |
|  | Asp/Tyr ratio | 0.26 |  | Ile/Cys ratio | 0.88 |
|  | Ile/Lys ratio | 0.26 |  | Cys-Arg count | 0.87 |
|  | Arg/Glu ratio | 0.26 |  | Ala/Lys ratio | 0.85 |
|  | Asp/Ser ratio | 0.26 |  | Glu/Cys ratio | 0.85 |
|  |  |  |  | Phe-Lys count | 0.85 |
| Gini index | Phe-Lys count | 1.00 |  | Cys/Met ratio | 0.84 |
|  | Asp-His count | 1.00 |  | Thr/Asp ratio | 0.84 |
|  | Cys-Arg count | 0.97 |  | Cys-Phe count | 0.83 |
|  | His-Asp count | 0.92 |  | Gln-Pro count | 0.83 |
|  | Leu/Cys ratio | 0.88 |  | Gly/Lys ratio | 0.83 |
|  | Val-Ala count | 0.87 |  | Ile/Asp ratio | 0.82 |
|  | Glu-Asn count | 0.87 |  | His-Glu count | 0.81 |
|  | Cys-Cys count | 0.87 |  | Ala/The ratio | 0.80 |
|  | Pro-Asp count | 0.86 |  | Phe-Asp count | 0.80 |
|  | Gln-Ile count | 0.86 |  | Ala/Phe ratio | 0.80 |
|  | Glu-Asp count | 0.86 |  | His-Asp count | 0.79 |
|  | Thr-His count | 0.85 |  | Gly-Ile count | 0.78 |
|  | Ile-Thr count | 0.85 |  | Cys-His count | 0.77 |
|  | Asp-Cys count | 0.85 |  | Glu/Lys ratio | 0.77 |
|  | Ala-Asn count | 0.84 |  | Thr/Arg ratio | 0.77 |
|  | Leu-Asp count | 0.84 |  | Isoelectric point | 0.77 |
|  | Asn-Gln count | 0.83 |  | Asp/Ser ratio | 0.76 |
|  | His-Glu count | 0.83 |  | Leu-Asp count | 0.76 |
|  | Cys-Thr count | 0.81 |  | Val-Ala count | 0.75 |
|  | His-Lys count | 0.81 |  | Glu-Asp count | 0.75 |
|  | Thr-Ile count | 0.80 |  | Cys/Tyr ratio | 0.74 |
|  | Phe-Pro count | 0.79 |  | Leu/Asp ratio | 0.74 |
|  | Asp-Asn count | 0.79 |  | Ile-Thr count | 0.73 |
|  | Glu-Leu count | 0.78 |  | Asp/Phe ratio | 0.73 |
|  | Arg-Leu count | 0.75 |  | Thr-His count | 0.72 |
|  | Asn-His count | 0.74 |  | Cys-Thr count | 0.71 |
|  | Val-Asp count | 0.73 |  | Asn-Gln count | 0.71 |
|  | Ser-Tyr count | 0.73 |  | Asn-Glu count | 0.71 |
|  | Ala-Ile count | 0.70 |  | Ala-Met count | 0.71 |
|  |  |  |  | Gly-Arg count | 0.71 |
| info gain ratio | Asp/Lys ratio | 1.00 |  | Pro-Asp count | 0.71 |
|  | Lys-Cys count | 0.98 |  | Gly-Val count | 0.70 |
|  | Phe/Cys ratio | 0.98 |  | Cys-Cys count | 0.70 |
| Weighting method | Attribute | Weight | Weighting method | Attribute | Weight |
| PCA | Thr/Cys ratio | 1.00 |  | Ala-Leu count | 0.72 |
|  | Lys-Cys count | 0.97 |  | Arg-Leu count | 0.71 |
|  | Ser/Cys ratio | 0.96 |  | His-Val count | 0.71 |
|  | Cys-His count | 0.93 |  | Ala/The ratio | 0.71 |
|  | Lys/Cys ratio | 0.92 |  | Val/Ser ratio | 0.71 |
|  | Phe/Cys ratio | 0.92 |  | Lys-Gln count | 0.70 |
|  | Leu/Cys ratio | 0.92 |  | Asp count | 0.70 |
|  | Phe-Asp count | 0.91 |  |  |  |
|  | Gln-Pro count | 0.91 | relief | Asp-Cys count | 1 |
|  | Thr/Asp ratio | 0.90 |  | Asp-Trp count | 0.96 |
|  | Asp/Lys ratio | 0.90 |  | Gln-Trp count | 0.80 |
|  | Cys/Met ratio | 0.89 |  | Glu-Pro count | 0.73 |
|  | Leu/Asp ratio | 0.85 |  | His-Tyr count | 0.72 |
|  | Asp/Met ratio | 0.85 |  | Cys-Cys count | 0.64 |
|  | Ile/Cys ratio | 0.84 |  | His-Asp count | 0.63 |
|  | Leu/Ala ratio | 0.84 |  | Arg-Thr count | 0.63 |
|  | Cys-Phe count | 0.84 |  | Asn-His count | 0.62 |
|  | Ile/Asp ratio | 0.83 |  | Thr-His count | 0.62 |
|  | Asp/Phe ratio | 0.82 |  | Asn-Gln count | 0.61 |
|  | Isoelectric point | 0.82 |  | Asn-Gly count | 0.61 |
|  | Asp/Ser ratio | 0.81 |  | Ala-Asn count | 0.59 |
|  | Arg-Gln count | 0.78 |  | Tyr-Leu count | 0.58 |
|  | Cys count | 0.78 |  | Ser-His count | 0.58 |
|  | Phe-Asn count | 0.77 |  | Met-Gln count | 0.56 |
|  | Val/Met ratio | 0.77 |  | Val-Ala count | 0.56 |
|  | Gly-Ile count | 0.77 |  | Cys-Gly count | 0.55 |
|  | Ala/Lys ratio | 0.76 |  | Ser-Arg count | 0.55 |
|  | Asp-Arg count | 0.76 |  | Lys-Val count | 0.54 |
|  | Ala/Met ratio | 0.76 |  | Glu-Asn count | 0.53 |
|  | Gln-Glu count | 0.76 |  | His-Arg count | 0.52 |
|  | Asn-Ala count | 0.76 |  | Cys-Thr count | 0.52 |
|  | Glu/Cys ratio | 0.76 |  | Glu-Asp count | 0.51 |
|  | Cys-Val count | 0.75 |  |  |  |
|  | Trp-Asp count | 0.75 | Rule | Ile percentage | 1 |
|  | Ala-Cys count | 0.75 |  | Leu percentage | 0.95 |
|  | Cys-Ile count | 0.75 |  | Arg/Cys ratio | 0.87 |
|  | Leu-Glu count | 0.74 |  | Gln/Cys ratio | 0.87 |
|  | Thr-Phe count | 0.74 |  | Leu/Cys ratio | 0.86 |
|  | Leu/Val rate | 0.74 |  | Asp/Lys ratio | 0.85 |
|  | Arg-Cys count | 0.73 |  | Thr percentage | 0.84 |
|  | Val-Leu count | 0.73 |  | Leu/His ratio | 0.83 |
|  | Cys/Tyr ratio | 0.72 |  | Ile/Cys ratio | 0.80 |
|  | Gly/Met ratio | 0.72 |  | Leu/Arg ratio | 0.78 |
|  | Asp/Tyr ratio | 0.72 |  | Lys/His ratio | 0.78 |
| Weighting method | Attribute | Weight | Weighting method | Attribute | Weight |
|  | Gly/Asp ratio | 0.77 |  | Glu-Leu count | 0.65 |
|  | Thr/Asn ratio | 0.77 |  | Cys-Thr count | 0.65 |
|  | Gly percentage | 0.77 |  | Glu-Asp count | 0.64 |
|  | Gly/Cys ratio | 0.77 |  | Lys-Arg count | 0.64 |
|  | Phe percentage | 0.77 |  | Asn-Glu count | 0.64 |
|  | Ala/Cys ratio | 0.74 |  | Cys count | 0.63 |
|  | Thr/Cys ratio | 0.74 |  | Asp-Cys count | 0.63 |
|  | Iso/Lys ratio | 0.73 |  | Thr-Glu count | 0.62 |
|  | Lys/Arg ratio | 0.73 |  | Phe-Pro count | 0.62 |
|  | Ser/Cys ratio | 0.71 |  | Gln-Ile count | 0.62 |
|  | Leu/Asp ratio | 0.69 |  | Leu/Asp rate | 0.61 |
|  | Leu/Val ratio | 0.69 |  | Val-Asp count | 0.61 |
|  | Leu/Pro ratio | 0.68 |  | Glu-Asn count | 0.60 |
|  | Lys/Asp ratio | 0.68 |  |  |  |
|  | Leu/Try ratio | 0.68 | SVM | Asn-His count | 1.00 |
|  | Ser/Asn ratio | 0.68 |  | Cys-Arg count | 0.95 |
|  | Ala/Glu ratio | 0.67 |  | Gln-Phe count | 0.95 |
|  | Asp/Arg ratio | 0.67 |  | Ser-Tyr count | 0.93 |
|  | Gly/Phe ratio | 0.67 |  | His-Lys count | 0.91 |
|  | NCEC | 0.67 |  | Cys-Cys count | 0.89 |
|  | Met/His ratio | 0.65 |  | Thr-His count | 0.89 |
|  | Lys/Cys ratio | 0.64 |  | Ile-Thr count | 0.89 |
|  | Phe/His ratio | 0.64 |  | Glu-Asn count | 0.88 |
|  | Arg/Try ratio | 0.63 |  | His-Glu count | 0.87 |
|  | Ile/Arg ratio | 0.63 |  | His-Asp count | 0.87 |
|  | Gln/Tyr ratio | 0.62 |  | Lys-Tyr count | 0.87 |
|  | Ile-Thr count | 0.60 |  | Glu-Asp count | 0.86 |
|  |  |  |  | Pro-Asp count | 0.84 |
| uncertainty | Phe-Lys count | 1.00 |  | Phe-Pro count | 0.84 |
|  | His-Glu count | 0.92 |  | His-His count | 0.83 |
|  | Pro-Asp count | 0.85 |  | Asp-Asn count | 0.83 |
|  | Asp-His count | 0.81 |  | Gln-Ile count | 0.81 |
|  | Ile-Thr count | 0.76 |  | Asn-Gln count | 0.81 |
|  | His-Asp count | 0.76 |  | Met-Val count | 0.80 |
|  | Cys-Arg count | 0.76 |  | Phe-Lys count | 0.80 |
|  | Asn-Gln count | 0.73 |  | Ala-Ile count | 0.80 |
|  | Cys-Cys count | 0.71 |  | Asp-Cys count | 0.79 |
|  | Leu-Asp count | 0.70 |  | Glu-Phe count | 0.79 |
|  | Leu/Cys ratio | 0.70 |  | Asp-His count | 0.75 |
|  | Gln-Pro count | 0.68 |  | Asp-Pro count | 0.74 |
|  | Cys-Phe count | 0.67 |  | Ile-Asn count | 0.73 |
|  | Thr-His count | 0.67 |  | Glu-Leu count | 0.73 |
|  | Arg-Leu count | 0.67 |  | His-Arg count | 0.72 |
|  | Lys-Val count | 0.65 |  | Pro-Thr count | 0.72 |
| Weighting method | Attribute | Weight | Weighting method | Attribute | Weight |
|  | Thr-Ile count | 0.72 |  |  |  |
|  | Asn-Glu count | 0.71 |  |  |  |
|  | Ser-Ala count | 0.70 |  |  |  |

NCEC: Non-reduced cysteines extinction coefficient
