# Supplementary material for: Bioinformatics approaches for classification and investigation of the evolution of the Na/K-ATPase alpha-subunit
Source: BMC Ecol Evol. 2022 Oct 26;22:122. doi: 10.1186/s12862-022-02071-0 (PMC9609216; doi:10.1186/s12862-022-02071-0)
Supplement: Supplementary file 1 — Additional file 1. Supplementary figures and tables. [file 12862_2022_2071_MOESM1_ESM.zip › Additional file 1 Table. S4.docx]

| Table S4: The accession number of sequences of Na/K ATPase pumps alpha-subunit from various organisms of three life domains and database providing the relevant sequence | | | | | |
| --- | --- | --- | --- | --- | --- |
| **Accession No.** | **Database** | **Accession No.** | **Database** | **Accession No.** | **Database** |
| ABA02167.1 | NCBI | AGZ13696.1 | NCBI | EFX71104.1 | NCBI |
| ABD59803.1 | NCBI | AGZ87948.1 | NCBI | EFX71105.1 | NCBI |
| ABF58911.1 | NCBI | AHB86584.1 | NCBI | EFX88073.1 | NCBI |
| ABO61332.1 | NCBI | AHB86585.1 | NCBI | EFX88361.1 | NCBI |
| ABO61333.1 | NCBI | AHB86586.1 | NCBI | EGD73524.1 | NCBI |
| ACB20770.2 | NCBI | AHD24596.1 | NCBI | EGD75712.1 | NCBI |
| ACB20771.2 | NCBI | AHH35009.1 | NCBI | EGD77429.1 | NCBI |
| ADB03120.1 | NCBI | AIM43570.1 | NCBI | EGI67709.1 | NCBI |
| ADD60471.1 | NCBI | AIR93635.1 | NCBI | ELK30843.1 | NCBI |
| ADN83843.1 | NCBI | AJO70000.1 | NCBI | ELK32312.1 | NCBI |
| ADY40856.1 | NCBI | AJO70183.1 | NCBI | ELK38498.1 | NCBI |
| ADY40930.1 | NCBI | AJR20270.1 | NCBI | ELK38499.1 | NCBI |
| AEX07319.1 | NCBI | AJR20271.1 | NCBI | ELU12040.1 | NCBI |
| AFM54541.1 | NCBI | AKB42896.1 | NCBI | EMP33651.1 | NCBI |
| AFU25665.1 | NCBI | AKB83839.1 | NCBI | EPQ02424.1 | NCBI |
| AFU25666.1 | NCBI | AKG50106.1 | NCBI | EPQ03777.1 | NCBI |
| AFU25667.1 | NCBI | AKQ12834.1 | NCBI | ERE90024.1 | NCBI |
| AFU25668.1 | NCBI | ALA65287.2 | NCBI | ETE67008.1 | NCBI |
| AFU25670.1 | NCBI | ALB35496.1 | NCBI | ETN62539.1 | NCBI |
| AFU25671.1 | NCBI | ALJ53300.1 | NCBI | EZA47803.1 | NCBI |
| AFU25672.1 | NCBI | AMK38059.1 | NCBI | EZA51212.1 | NCBI |
| AFU25673.1 | NCBI | AOG19177.1 | NCBI | GAX12878.1 | NCBI |
| AFU25675.1 | NCBI | AUG84438.1 | NCBI | GAX20661.1 | NCBI |
| AFU25676.1 | NCBI | BAA32798.1 | NCBI | KFO33633.1 | NCBI |
| AFU25678.1 | NCBI | BAA82752.2 | NCBI | KFP84941.1 | NCBI |
| AFU25679.1 | NCBI | BAB60722.1 | NCBI | KFU90062.1 | NCBI |
| AFU25681.1 | NCBI | BAJ13363.1 | NCBI | NP_001009360.1 | NCBI |
| AFU25682.1 | NCBI | BAN17691.1 | NCBI | NP_001070266.1 | NCBI |
| AFU25683.1 | NCBI | BAO02373.1 | NCBI | NP_0010749 | NCBI |
| AFU25686.1 | NCBI | BAS22117.1 | NCBI | NP_001080440.1 | NCBI |
| AFU25689.1 | NCBI | CAI99405.1 | NCBI | NP_001083112.1 | NCBI |
| AFU25691.1 | NCBI | CAI99406.1 | NCBI | NP_0010840 | NCBI |
| AFU25692.1 | NCBI | CCA16430.1 | NCBI | NP_001117930.1 | NCBI |
| AFU25694.1 | NCBI | CDS22215.1 | NCBI | NP_001122529.1 | NCBI |
| AFU25695.1 | NCBI | CDS36343.1 | NCBI | NP_001125304.1 | NCBI |
| AGF90965.1 | NCBI | CDW54807.1 | NCBI | NP_001137575.2 | NCBI |
| AGM39710.1 | NCBI | CDW55413.1 | NCBI | NP_0011565 | NCBI |
| AGO02179.1 | NCBI | CEF64940.1 | NCBI | NP_0011650 | NCBI |
| AGR45921.1 | NCBI | CRX73232.1 | NCBI | NP_0012526 | NCBI |
| AGR87393.1 | NCBI | EFN85240.1 | NCBI | NP_0012536 | NCBI |
| AGR87394.1 | NCBI | EFN88446.1 | NCBI | NP_0012967 | NCBI |
| AGY54951.1 | NCBI | EFX69525.1 | NCBI | NP_0012969 | NCBI |
| AGZ13694.1 | NCBI | EFX71103.1 | NCBI | NP_0012973 | NCBI |
| NP_036637. | NCBI | AAF17586.1 | NCBI | KYQ48574.1 | NCBI |
| **Accession No.** | **Database** | **Accession No.** | **Database** | **Accession No.** | **Database** |
| NP_036638. | NCBI | AAF20202.1 | NCBI | KYQ51534.1 | NCBI |
| NP_038762. | NCBI | AAG47843.1 | NCBI | KZC06498.1 | NCBI |
| NP_074039. | NCBI | AAL09322.1 | NCBI | KZC11054.1 | NCBI |
| NP_633093.1 | NCBI | AAT48993.1 | NCBI | NP_000692. | NCBI |
| ODM99113.1 | NCBI | AAX09623.1 | NCBI | NP_000693. | NCBI |
| ODN01960.1 | NCBI | KFW61640.1 | NCBI | NP_001116982.1 | NCBI |
| ODN05419.1 | NCBI | KHJ49479.1 | NCBI | NP_276630.1 | NCBI |
| OPJ66608.1 | NCBI | KHN72407.1 | NCBI | NP_440621.1 | NCBI |
| OQR92998.1 | NCBI | KHN74191.1 | NCBI | NP_571758.1 | NCBI |
| OQR94535.1 | NCBI | KHN82508.1 | NCBI | NP_571759.2 | NCBI |
| OQR99879.1 | NCBI | KHN88766.1 | NCBI | NP_571761.1 | NCBI |
| OQS04799.1 | NCBI | KHN88767.1 | NCBI | NP_571762.1 | NCBI |
| OQV17561.1 | NCBI | KKF19362.1 | NCBI | NP_571763.1 | NCBI |
| OQV18895.1 | NCBI | KKF24497.1 | NCBI | NP_653300. | NCBI |
| OQV20867.1 | NCBI | KNC28219.1 | NCBI | NP_659149. | NCBI |
| OQV25202.1 | NCBI | KOC67548.1 | NCBI | NP_732572. | NCBI |
| OUS42873.1 | NCBI | KOX77187.1 | NCBI | NP_835200.1 | NCBI |
| OWK04910.1 | NCBI | KPI92424.1 | NCBI | NP_989407.1 | NCBI |
| OWR44555.1 | NCBI | KPI97351.1 | NCBI | NP_990852. | NCBI |
| OWR53886.1 | NCBI | KPJ02649.1 | NCBI | OAD46911.1 | NCBI |
| OXA54637.1 | NCBI | KPJ07193.1 | NCBI | ODM90865.1 | NCBI |
| OXA57033.1 | NCBI | KPP65694.1 | NCBI | ODM96221.1 | NCBI |
| OXA63786.1 | NCBI | KQK85052.1 | NCBI | ODM98254.1 | NCBI |
| OZC08885.1 | NCBI | KRX24043.1 | NCBI | ODM98837.1 | NCBI |
| P50997.1 | NCBI | KRX35740.1 | NCBI | XP_001346890.1 | NCBI |
| PNI19721.1 | NCBI | KRX38989.1 | NCBI | XP_001427178.1 | NCBI |
| PNI95395.1 | NCBI | KRY10799.1 | NCBI | XP_001742517.1 | NCBI |
| PNJ19200.1 | NCBI | KRY33278.1 | NCBI | XP_001901816.1 | NCBI |
| PNJ46178.1 | NCBI | KRY48181.1 | NCBI | XP_003143231.1 | NCBI |
| POM62354.1 | NCBI | KRY70033.1 | NCBI | XP_003369418.1 | NCBI |
| PXF41326.1 | NCBI | KRY76685.1 | NCBI | XP_003415228.1 | NCBI |
| PXF41383.1 | NCBI | KRZ04306.1 | NCBI | XP_003466610.1 | NCBI |
| RAW38513.1 | NCBI | KRZ50957.1 | NCBI | XP_003795244.1 | NCBI |
| YP_001963725.1 | NCBI | KRZ52541.1 | NCBI | XP_003795245.1 | NCBI |
| YP_324582.1 | NCBI | KRZ73739.1 | NCBI | XP_003799510.1 | NCBI |
| YP_357688.1 | NCBI | KRZ74907.1 | NCBI | XP_003892961.1 | NCBI |
| YP_391334.1 | NCBI | KXJ20388.1 | NCBI | XP_004066573.1 | NCBI |
| YP_502111.1 | NCBI | KXJ20422.1 | NCBI | XP_004066575.1 | NCBI |
| YP_565169.1 | NCBI | KYN03549.1 | NCBI | XP_004074116.1 | NCBI |
| XP_026576074.1 | NCBI | KYN18319.1 | NCBI | XP_004352438.1 | NCBI |
| A0A090M1W3 | NCBI | KYN38456.1 | NCBI | XP_004380410.1 | NCBI |
| AAA41671.1 | NCBI | KYO43368.1 | NCBI | XP_004390257.1 | NCBI |
| XP_004448484.1 | NCBI | XP_012604632.1 | NCBI | XP_020726792.1 | NCBI |
| **Accession No.** | **Database** | **Accession No.** | **Database** | **Accession No.** | **Database** |
| XP_004448489.1 | NCBI | XP_012604635.1 | NCBI | XP_020740848.1 | NCBI |
| XP_004536046.2 | NCBI | XP_012613923.1 | NCBI | XP_020740887.1 | NCBI |
| XP_004536048.1 | NCBI | XP_012617266.1 | NCBI | XP_020747989.1 | NCBI |
| XP_004550929.1 | NCBI | XP_012663099.1 | NCBI | XP_020792263.1 | NCBI |
| XP_004571307.1 | NCBI | XP_012711044.2 | NCBI | XP_020793662.1 | NCBI |
| XP_004639995.1 | NCBI | XP_012714443.1 | NCBI | XP_020796901.1 | NCBI |
| XP_004639996.1 | NCBI | XP_013220247.1 | NCBI | XP_020799552.1 | NCBI |
| XP_004853865.1 | NCBI | XP_013405520.1 | NCBI | XP_020835237.1 | NCBI |
| XP_004858786.1 | NCBI | XP_014205893.1 | NCBI | XP_020858281.1 | NCBI |
| XP_004931505.2 | NCBI | XP_014232354.1 | NCBI | XP_020944376.1 | NCBI |
| XP_005076578.1 | NCBI | XP_014250371.1 | NCBI | XP_020948935.1 | NCBI |
| XP_005292736.1 | NCBI | XP_014271921.1 | NCBI | XP_021013125.1 | NCBI |
| XP_005293820.1 | NCBI | XP_015032883.1 | NCBI | XP_021014708.1 | NCBI |
| XP_005334975.1 | NCBI | XP_015461719.2 | NCBI | XP_021021704.1 | NCBI |
| XP_005339432.1 | NCBI | XP_015585114.1 | NCBI | XP_021051287.1 | NCBI |
| XP_005511501.1 | NCBI | XP_015907346.1 | NCBI | XP_021054982.1 | NCBI |
| XP_005796664.1 | NCBI | XP_015929949.2 | NCBI | XP_021106581.1 | NCBI |
| XP_006038189.1 | NCBI | XP_015929951.2 | NCBI | XP_021196082.1 | NCBI |
| XP_006096963.1 | NCBI | XP_015930974.1 | NCBI | XP_021253236.1 | NCBI |
| XP_006132947.1 | NCBI | XP_016780478.1 | NCBI | XP_021404823.1 | NCBI |
| XP_006903931.1 | NCBI | XP_017270842.1 | NCBI | XP_021426673.1 | NCBI |
| XP_006919736.1 | NCBI | XP_017282368.1 | NCBI | XP_021427657.1 | NCBI |
| XP_006922963.1 | NCBI | XP_018562050.1 | NCBI | XP_021504168.1 | NCBI |
| XP_006922964.1 | NCBI | XP_018565491.1 | NCBI | XP_021506251.1 | NCBI |
| XP_007129684.1 | NCBI | XP_018651572.1 | NCBI | XP_021537506.1 | NCBI |
| XP_007435355.1 | NCBI | XP_019677883.4 | NCBI | XP_021537588.1 | NCBI |
| XP_008056914.2 | NCBI | XP_020012504.1 | NCBI | XP_021568356.1 | NCBI |
| XP_008065591.1 | NCBI | XP_020024800.1 | NCBI | XP_021590883.1 | NCBI |
| XP_008071711.2 | NCBI | XP_020024807.1 | NCBI | XP_021693479.1 | NCBI |
| XP_008322794.1 | NCBI | XP_020294182.1 | NCBI | XP_021915175.1 | NCBI |
| XP_008604114.1 | NCBI | XP_020328431.1 | NCBI | XP_021944101.1 | NCBI |
| XP_008607481.1 | NCBI | XP_020466584.1 | NCBI | XP_022053465.1 | NCBI |
| XP_008971666.1 | NCBI | XP_020476182.1 | NCBI | XP_022078036.1 | NCBI |
| XP_010587900.1 | NCBI | XP_020497843.1 | NCBI | XP_022114884.1 | NCBI |
| XP_010593170.2 | NCBI | XP_020504733.1 | NCBI | XP_022184331.1 | NCBI |
| XP_011283388.1 | NCBI | XP_020507674.1 | NCBI | XP_022210861.1 | NCBI |
| XP_011371380.1 | NCBI | XP_020601998.1 | NCBI | XP_022253133.1 | NCBI |
| XP_012163873.1 | NCBI | XP_020602001.1 | NCBI | XP_022254094.1 | NCBI |
| XP_012269651.1 | NCBI | XP_020602016.1 | NCBI | XP_022323941.1 | NCBI |
| XP_012272094.1 | NCBI | XP_020645227.1 | NCBI | XP_022352592.1 | NCBI |
| XP_012279085.1 | NCBI | XP_020653823.1 | NCBI | XP_022415031.1 | NCBI |
| XP_012314296.1 | NCBI | XP_020663591.1 | NCBI | XP_022439684.1 | NCBI |
| XP_022441242.1 | NCBI | XP_024064252.1 | NCBI | A0A1Y2BYC1 | UniProt |
| XP_022530668.1 | NCBI | XP_024144684.1 | NCBI | A0A1Y2G5J7 | UniProt |
| XP_022536277.1 | NCBI | XP_024144685.1 | NCBI | A0A1Y2HI79 | UniProt |
| XP_022612296.1 | NCBI | XP_024153267.1 | NCBI | A0A261Y8Y1 | UniProt |
| **Accession No.** | **Database** | **Accession No.** | **Database** | **Accession No.** | **Database** |
| XP_022617258.1 | NCBI | XP_024297426.1 | NCBI | A0A2G5BEZ7 | UniProt |
| XP_022668602.1 | NCBI | XP_024426171.1 | NCBI | A0A2J6SAB3 | UniProt |
| XP_022817943.1 | NCBI | XP_024433413.1 | NCBI | A0A2J6SIQ3 | UniProt |
| XP_022903571.1 | NCBI | XP_024502753.1 | NCBI | A0A2N0PA04 P | UniProt |
| XP_023016793.1 | NCBI | XP_024578788.1 | NCBI | A0A2Z6QEU7 | UniProt |
| XP_023069989.1 | NCBI | XP_024585310.1 | NCBI | A0A317MXL5 | UniProt |
| XP_023069991.1 | NCBI | XP_024620662.1 | NCBI | A0A347ZR85 | UniProt |
| XP_023078532.1 | NCBI | XP_024883961.1 | NCBI | A0A397VHQ3 | UniProt |
| XP_023103614.1 | NCBI | XP_024920682.1 | NCBI | A0A447CBT6 | UniProt |
| XP_023117914.1 | NCBI | XP_025028557.1 | NCBI | A0A497XH49 | UniProt |
| XP_023121557.1 | NCBI | XP_025067531.1 | NCBI | A0A4P9WG54 | UniProt |
| XP_023135081.1 | NCBI | XP_025966334.1 | NCBI | A0A4P9WWS6 | UniProt |
| XP_023176860.1 | NCBI | XP_030317504.1 | NCBI | A0A4P9Y2Y3 | UniProt |
| XP_023185013.1 | NCBI | XP_030327328.1 | NCBI | A0A4P9Z1Y8 | UniProt |
| XP_023185631.1 | NCBI | XP_545753.3 | NCBI | A0A4P9ZM46 | UniProt |
| XP_023187147.1 | NCBI | XP_545754.3 | NCBI | A0A4R8A713 | UniProt |
| XP_023221168.1 | NCBI | XP_647420.2 | NCBI | A0A4S4MS29 | UniProt |
| XP_023221169.1 | NCBI | A0A034W3G9 | UniProt | A0A4Y9Y8I4 | UniProt |
| XP_023275950.1 | NCBI | A0A067M739 | UniProt | A0A4Y9ZEZ1 | UniProt |
| XP_023285663.1 | NCBI | A0A067Q0Q4 | UniProt | A0A507BSP0 | UniProt |
| XP_023321169.1 | NCBI | A0A087ZR23 | UniProt | A0A507CSE4 | UniProt |
| XP_023323782.1 | NCBI | A0A090D7D5 | UniProt | A0A507E7U2 | UniProt |
| XP_023336146.1 | NCBI | A0A0B7MNY4 | UniProt | A0A507EKQ2 | UniProt |
| XP_023337795.1 | NCBI | A0A0C3GKR9 | UniProt | A0A507F1Y9 | UniProt |
| XP_023347331.1 | NCBI | A0A0C9MZH6 | UniProt | A0A5E3X872 | UniProt |
| XP_023380497.1 | NCBI | A0A137NQA6 | UniProt | AMAG_01211.1 | UniProt |
| XP_023390675.1 | NCBI | A0A139AWN6 | UniProt | AMAG_02439.1 | UniProt |
| XP_023496657.1 | NCBI | A0A194X4V3 | UniProt | B5YJF3 | UniProt |
| XP_023507169.1 | NCBI | A0A1G6WYT0 | UniProt | BDEG_03368.1 | UniProt |
| XP_023557491.1 | NCBI | A0A1H1W1D1 | UniProt | BDEG_05936.1 | UniProt |
| XP_023600635.1 | NCBI | A0A1H7UXF5 | UniProt | CC1G_09151.2 | UniProt |
| XP_023616468.1 | NCBI | A0A1M2VF35 | UniProt | D3BBA2 | UniProt |
| XP_023653512.1 | NCBI | A0A1M6NFZ1 | UniProt | D6WB95 | UniProt |
| XP_023665796.1 | NCBI | A0A1M7YB86 | UniProt | EKC34610.1 | UniProt |
| XP_023690671.1 | NCBI | A0A1X2ID33 | UniProt | F1A2S2 | UniProt |
| XP_023711705.1 | NCBI | A0A1Y1VYW4 | UniProt | F7E0B8 | UniProt |
| XP_023796730.1 | NCBI | A0A1Y1YKS1 | UniProt | G4VGA0 | UniProt |
| XP_023954931.1 | NCBI | A0A1Y1YU47 | UniProt | I7M7N1 | UniProt |
| XP_023975434.1 | NCBI | A0A1Y2BXZ1 | UniProt | I7M7R6 | UniProt |
| I7MD85 | UniProt | UPI0000085D74 | UniProt | UPI00020239EC | UniProt |
| I7ME52 | UniProt | UPI00000FE1CF | UniProt | UPI000226419C | UniProt |
| I7MH18 | UniProt | UPI0000124FBE | UniProt | UPI000226F4AA | UniProt |
| I7MHE1 | UniProt | UPI0000124FC0 | UniProt | UPI00022B0848 | UniProt |
| J4GT94 | UniProt | UPI0000124FC2 | UniProt | UPI0002387025 | UniProt |
| K0C7I7 | UniProt | UPI0000124FC4 | UniProt | UPI00025F91A4 | UniProt |
| K7IWP3 | UniProt | UPI00001261C4 | UniProt | UPI00025FADDE | UniProt |
| **Accession No.** | **Database** | **Accession No.** | **Database** | **Accession No.** | **Database** |
| O16331 | UniProt | UPI00001DFF47 | UniProt | UPI00025FB25F | UniProt |
| O16436 | UniProt | UPI00001DFF4A | UniProt | UPI000274DF5C | UniProt |
| P13637.3 | UniProt | UPI000027C768 | UniProt | UPI00028A5EEF | UniProt |
| P25489.1 | UniProt | UPI00002BAA33 | UniProt | UPI00028AA5CE | UniProt |
| P30714.2 | UniProt | UPI0000318264 | UniProt | UPI00028AEC41 | UniProt |
| P90735 | UniProt | UPI00003628C3 | UniProt | UPI00028B8ECD | UniProt |
| PGTG_03133.2 | UniProt | UPI00003C6559 | UniProt | UPI00028BBB9D | UniProt |
| PIS80793.1 | UniProt | UPI000050D2B6 | UniProt | UPI000293B6B0 | UniProt |
| Q22LQ9 | UniProt | UPI000054C9F5 | UniProt | UPI000293EB87 | UniProt |
| Q22P96 | UniProt | UPI000056D0DB | UniProt | UPI0002B2E326 | UniProt |
| Q22PA2 | UniProt | UPI00005E9366a2 | UniProt | UPI0002B3612F | UniProt |
| Q22XZ1 | UniProt | UPI0000E10E8B | UniProt | UPI0002B3D77C | UniProt |
| Q23D88 | UniProt | UPI0000E3A2FA | UniProt | UPI00032B6FE9 | UniProt |
| Q23EX6 | UniProt | UPI0000E3AF2C | UniProt | UPI00032B9010 | UniProt |
| Q23ZA6 | UniProt | UPI0000E9CD46 | UniProt | UPI00032DDB86 | UniProt |
| Q245Y8 | UniProt | UPI0000F6BCEB | UniProt | UPI000342C523 | UniProt |
| Q6RWA9.1 | UniProt | UPI00014FFB3C | UniProt | UPI00035B05DE | UniProt |
| Q90X33 | UniProt | UPI000153A0D9 | UniProt | UPI0003B0448B | UniProt |
| Q92030.1 | UniProt | UPI000157ACC7 | UniProt | UPI0003CD047A | UniProt |
| Q98SL3 | UniProt | UPI00015F4774 | UniProt | UPI0003D8328F | UniProt |
| Q9DEU1 | UniProt | UPI0001643CDB | UniProt | UPI000443A733 | UniProt |
| Q9YH26.2 | UniProt | UPI00016E235F | UniProt | UPI0004448FEC | UniProt |
| RO3G_04175.3 | UniProt | UPI0001781834 | UniProt | UPI00049A9E19 | UniProt |
| S2JVU7 | UniProt | UPI0001782835 | UniProt | UPI0004A1BCA5 | UniProt |
| S8E4T6 | UniProt | UPI00017C692D | UniProt | UPI0004E4D157 | UniProt |
| SPPG_01615.2 | UniProt | UPI00017D261C | UniProt | UPI0004FDA0CB | UniProt |
| SPPG_07476.2 | UniProt | UPI000183E9C3 | UniProt | UPI00051ECCC0 | UniProt |
| SPPG_08470.2 | UniProt | UPI000186D98E | UniProt | UPI0005464DB9 | UniProt |
| T1E1Y4 | UniProt | UPI0001914BDE | UniProt | UPI0005474384 | UniProt |
| T1PH35 | UniProt | UPI0001C650F7 | UniProt | UPI0005476AAF | UniProt |
| T2B507 | UniProt | UPI0001C851CE | UniProt | UPI00057321B9 | UniProt |
| U4UIT1 | UniProt | UPI0001C9F9BA | UniProt | UPI0005D90DB9 | UniProt |
| U6PGW0 | UniProt | UPI0001D4FB7A | UniProt | UPI0005FA3D38 | UniProt |
| UPI0000068661 | UniProt | UPI0001E7C907 | UniProt | UPI000601D02E | UniProt |
| UPI000006A49F | UniProt | UPI0001FB338F | UniProt | UPI0006036B9D | UniProt |
| UPI00000795B1 | UniProt | UPI0001FEE5BA | UniProt | UPI0006052FD1 | UniProt |
| UPI00060544A6 | UniProt | UPI0006D327FB | UniProt | UPI000971E6B0 | UniProt |
| UPI000605CA49 | UniProt | UPI0006ED933E | UniProt | UPI0009F0198D | UniProt |
| UPI000605DBB4 | UniProt | UPI0006EDF4F9 | UniProt | UPI000B392785 | UniProt |
| UPI000605F508 | UniProt | UPI0006EE4186 | UniProt | UPI000B509329 | UniProt |
| UPI00060602BA | UniProt | UPI0007085A3B | UniProt | UPI000B551D17 | UniProt |
| UPI00060630B9 | UniProt | UPI0007325ED7 | UniProt | UPI000B76A669 | UniProt |
| UPI000609E432 | UniProt | UPI0007717391 | UniProt | UPI000BAD5294 | UniProt |
| UPI000609E51C | UniProt | UPI000790B508 | UniProt | UPI000BC5B284 | UniProt |
| UPI000609F1C5 | UniProt | UPI00079C7F01 | UniProt | UPI000BE6443D | UniProt |
| UPI00060AD39A | UniProt | UPI00079DB5F5 | UniProt | UPI000C21D419 | UniProt |
| UPI00060CD0E5 | UniProt | UPI00079E2EF3 | UniProt | UPI000C2216DF | UniProt |
| **Accession No.** | **Database** | **Accession No.** | **Database** | **Accession No.** | **Database** |
| UPI00060EAC2D | UniProt | UPI00079E81D7 | UniProt | UPI000C2D7C35 | UniProt |
| UPI0006101D56 | UniProt | UPI00079F518A | UniProt | UPI000C2DAA95 | UniProt |
| UPI0006120BCD | UniProt | UPI0007A15AF4 | UniProt | UPI000C2E3154 | UniProt |
| UPI0006154E5D | UniProt | UPI0007A17F26 | UniProt | UPI000C2E4C26 | UniProt |
| UPI00061563E3 | UniProt | UPI0007A19C37 | UniProt | UPI000C2E744C | UniProt |
| UPI0006157148 | UniProt | UPI0007A1BF54 | UniProt | UPI000C2EDFE7 | UniProt |
| UPI0006157AA6 | UniProt | UPI0007A24416 | UniProt | UPI000C2F2801 | UniProt |
| UPI0006157E2A | UniProt | UPI0007A2DD20 | UniProt | UPI000C71DF25 | UniProt |
| UPI000615B8D6 | UniProt | UPI0007A6EC9C | UniProt | UPI000C7286EF | UniProt |
| UPI000615BB49 | UniProt | UPI0007AA68F1 | UniProt | UPI000C732D5F | UniProt |
| UPI000651771D | UniProt | UPI0007D2379C | UniProt | UPI000C7355ED | UniProt |
| UPI00066EFDEA | UniProt | UPI0007D6117E | UniProt | UPI000C736357 | UniProt |
| UPI000672CD46 | UniProt | UPI0007E80B92 | UniProt | UPI000C73EFA7 | UniProt |
| UPI0006929A96 | UniProt | UPI0007F716B9 | UniProt | UPI000C740E55 | UniProt |
| UPI000692F512 | UniProt | UPI0007F7EA5B | UniProt | UPI000C746E0C | UniProt |
| UPI000692F5BD | UniProt | UPI000818DDE2 | UniProt | UPI000CB4CAB6 | UniProt |
| UPI0006B30A18 | UniProt | UPI00084EC8B0 | UniProt | UPI000CD7670C | UniProt |
| UPI0006B31231 | UniProt | UPI0008639F5F | UniProt | UPI000D26B24D | UniProt |
| UPI0006B37238 | UniProt | UPI000891AE6A | UniProt | V9I6A9 | UniProt |
| UPI0006B7181A | UniProt | UPI0008DEB8E8 | UniProt | W5L4G0 | UniProt |
| UPI0006B74D9B | UniProt | UPI00091201A3 | UniProt | W5U4R1 | UniProt |
| UPI0006B827F3 | UniProt | UPI00091A11F9 | UniProt | W5UML4 | UniProt |
| UPI0006BD3860 | UniProt | UPI000957746B | UniProt | W5W8S0 | UniProt |
