# Supplementary material for: Bioinformatics approaches for classification and investigation of the evolution of the Na/K-ATPase alpha-subunit
Source: BMC Ecol Evol. 2022 Oct 26;22:122. doi: 10.1186/s12862-022-02071-0 (PMC9609216; doi:10.1186/s12862-022-02071-0)
Supplement: Supplementary file 1 — Additional file 1. Supplementary figures and tables. [file 12862_2022_2071_MOESM1_ESM.zip › Additional file 1 Table. S5.docx]

| Table S5: The accession number of sequences of ssu rRNA (16S/18S rRNA) from various organisms of three life domains and database providing the relevant sequence | | | | | | |
| --- | --- | --- | --- | --- | --- | --- |
| **Accession No.** | **Database** | **Accession No.** | **Database** | **Accession No.** | **Database** | |
| AABR07015078.251 | SILVA | AB923887.1 | SILVA | AF173630.1 | SILVA | |
| AACZ04008123.9798 | SILVA | AB973355.1 | SILVA | AF227234.1 | SILVA | |
| AADK01009766.2275 | SILVA | AB973360.1 | SILVA | AF235966.1 | SILVA | |
| AADN05000165.44389 | SILVA | ABDC03037957.8314 | SILVA | AF235971.1 | SILVA | |
| AAEU02004727.6489 | SILVA | ABEG02006517.3670 | SILVA | AF267404.1 | SILVA | |
| AAEX03025866.1346 | SILVA | ABFJ01002139.1 | SILVA | AF296753.1 | SILVA | |
| AAFI02000261.30349 | SILVA | ABGA01165195.44113 | SILVA | AF308337.1 | SILVA | |
| AAFR03070865.1993 | SILVA | ABKE03000005.2938627 | SILVA | AF308735.1 | SILVA | |
| AAGJ05110284.9400 | SILVA | ABLE03009067.286 | SILVA | AF370793.1 | SILVA | |
| AAGU03092596.576 | SILVA | ABRM01124247.12 | SILVA | AFEY01231219.4 | SILVA | |
| AAGW02076165.15900 | SILVA | ABRO02100689.1389 | SILVA | AFFG02006695.4991 | SILVA | |
| AAKN02012213.64179 | SILVA | ABRP02219353.2270 | SILVA | AGBW02008696.10633 | SILVA | |
| AAKO01005049.7915 | SILVA | ABRT02368317.14362 | SILVA | AGKD04000193.118957 | SILVA | |
| AAMC03028735.7191 | SILVA | AC167688.8242 | SILVA | AGRG01008637.1 | SILVA | |
| AANG04003407.38427 | SILVA | AC197289.37429 | SILVA | AGSK01136783.972 | SILVA | |
| AANH01014982.6095 | SILVA | AC278063.51923 | SILVA | AGTA05001152.3050 | SILVA | |
| AANI01013721.2523 | SILVA | ADBW01003330.1 | SILVA | AH012380.71441 | SILVA | |
| AANN01653944.1 | SILVA | ADID01001107.1 | SILVA | AHKG01114378.12 | SILVA | |
| AAPQ01005416.1291 | SILVA | ADMH02002220.5230 | SILVA | AHZZ02094608.1 | SILVA | |
| AAPU01010424.3696 | SILVA | ADND02041479.1485 | SILVA | AJ301859.1 | SILVA | |
| AAQB01005291.2086 | SILVA | ADTU01030630.56 | SILVA | AJ311674.1 | SILVA | |
| AAQQ01381945.17 | SILVA | AEAB01019515.3499 | SILVA | AJ781608.1 | SILVA | |
| AAQR03187491.1 | SILVA | AEHK01400485.183 | SILVA | AJ841435.1 | SILVA | |
| AATS01000005.157233 | SILVA | AELG01008047.1 | SILVA | AJ920356.1 | SILVA | |
| AAWR02049966.14 | SILVA | AEMK02000489.43234 | SILVA | AJFE02008046.261 | SILVA | |
| AB001287.1 | SILVA | AEUI03000021.4526885 | SILVA | AJSA01185644.12351 | SILVA | |
| AB028214.1 | SILVA | AEVX01007365.10630 | SILVA | ALAR01203917.98 | SILVA | |
| AB048358.1 | SILVA | AF007533.1 | SILVA | ALWS01159237.12 | SILVA | |
| AB064942.1 | SILVA | AF007538.1 | SILVA | AM168086.1 | SILVA | |
| AB105163.1 | SILVA | AF013153.1 | SILVA | ANKR01218989.1 | SILVA | |
| AB110542.1 | SILVA | AF018653.1 | SILVA | AONE01045442.161 | SILVA | |
| AB214970.1 | SILVA | AF021880.1 | SILVA | AP012205.2451003 | SILVA | |
| AB231858.1 | SILVA | AF026599.1 | SILVA | AP012304.1159759 | SILVA | |
| AB235853.1 | SILVA | AF030250.1 | SILVA | APAU02000841.1 | SILVA | |
| AB288262.1 | SILVA | AF036588.1 | SILVA | APCI01008749.303 | SILVA | |
| AB370245.1 | SILVA | AF036608.1 | SILVA | APMK01076957.3 | SILVA | |
| AB511834.1 | SILVA | AF113410.1 | SILVA | APWO02000427.2086 | SILVA | |
| AB538282.1 | SILVA | AF113424.1 | SILVA | AQIA01042723.170 | SILVA | |
| AB598277.1 | SILVA | AF116011.1 | SILVA | ASVU01000001.565199 | SILVA | |
| AB699092.1 | SILVA | AF120505.1 | SILVA | AUST01037318.652 | SILVA | |
| AB747095.1 | SILVA | AF157149.1 | SILVA | AVPB01117667.12562 | SILVA | |
| AB769957.1 | SILVA | AF173605.1 | SILVA | AX207386.3 | SILVA | |
| AB917944.1 | SILVA | AF173610.1 | SILVA | AXZI02026981.3997 | SILVA | |
| AB923886.1 | SILVA | AF173614.1 | SILVA | AY040685.1 | SILVA | |
| AY049813.1 | SILVA | CP000142 | SILVA | GBIF01045948.6575 | SILVA | |
| AY216711.1 | SILVA | CP000254.1301079 | SILVA | GBXD01011165.677 | SILVA | |
| AY284682.1 | SILVA | CP000777.1126486 | SILVA | GDFR01001397.453 | SILVA | |
| AY329635.1 | SILVA | CP003083.1778722 | SILVA | GECA01009014.5769 | SILVA | |
| AY329636.1 | SILVA | CP007155.1135239 | SILVA | GECX01047082.29 | SILVA | |
| AY334566.1 | SILVA | CP009505.45021 | SILVA | GEEA01028006.1 | SILVA | |
| AY336764.1 | SILVA | CP009506.49221 | SILVA | GEEF01054809.5881 | SILVA | |
| AY336764.1 | SILVA | CP009513.40520 | SILVA | GEEY01007882.533 | SILVA | |
| AY336788.1 | SILVA | CP009520.44569 | SILVA | GEMX01008541.1 | SILVA | |
| AY349032.1 | SILVA | CP009524.45029 | SILVA | GFBK01030108.398 | SILVA | |
| AY497012.1 | SILVA | CP018799.352707 | SILVA | GFFJ01040029.176 | SILVA | |
| AY508034.1 | SILVA | CP018800.1916659 | SILVA | GFMJ01000202.450 | SILVA | |
| AY555515.1 | SILVA | DQ079763.1 | SILVA | GFMO01011947.869 | SILVA | |
| **Accession No.** | **Database** | **Accession No.** | **Database** | **Accession No.** | **Database** | |
| AY635842.1 | SILVA | DQ094173.1 | SILVA | GFNA01343415.5338 | SILVA | |
| AY662667.1 | SILVA | DQ116748.1 | SILVA | GFOR01006137.643 | SILVA | |
| AY703529.1 | SILVA | DQ133074.1 | SILVA | GFVN01007659.94 | SILVA | |
| AY742745.1 | SILVA | DQ157224.1 | SILVA | GFVZ01102020.672 | SILVA | |
| AY762619.1 | SILVA | DQ222453.1 | SILVA | GGBT01023314.66 | SILVA | |
| AY781436.1 | SILVA | DQ657903.1 | SILVA | GGMX01146371.1205 | SILVA | |
| AY851257.1 | SILVA | DQ882635.1 | SILVA | GGUK01011242.354 | SILVA | |
| AY851259.1 | SILVA | EF173848.1 | SILVA | GGVL01087394.173 | SILVA | |
| AY851261.1 | SILVA | EF502045.1 | SILVA | GQ410576.1 | SILVA | |
| AY851263.1 | SILVA | EU011924.1 | SILVA | GQ410604.1 | SILVA | |
| AY856093.1 | SILVA | EU158084.1 | SILVA | GQ410677.1 | SILVA | |
| AY894303.1 | SILVA | EU434875.1 | SILVA | GU017319.1 | SILVA | |
| AY940359.1 | SILVA | EU780557.2167 | SILVA | HACI01424225.69 | SILVA | |
| AY947720.1 | SILVA | EU823286.1 | SILVA | HAGT01095866.1180 | SILVA | |
| AY947720.1 | SILVA | FJ267399.1 | SILVA | HF569044.1 | SILVA | |
| AYUL01001070.7275 | SILVA | FJ380929.214 | SILVA | HM156711.1 | SILVA | |
| AZBK01062934.707 | SILVA | FJ438821.1 | SILVA | HM641689.1 | SILVA | |
| AZIM01008838.13053 | SILVA | FJ535847.1 | SILVA | HQ440575.1 | SILVA | |
| BBKB01008091.93 | SILVA | FJ710819.1 | SILVA | HQ901722.1 | SILVA | |
| BDQP01001207.293 | SILVA | FJ965974.1 | SILVA | IABY01000175.406 | SILVA | |
| BDQW01004811.1 | SILVA | FKLU01001655.20386 | SILVA | JABR01093782.795 | SILVA | |
| BDUG01002520.144616 | SILVA | FMBV02015990.51175 | SILVA | JACK01010267.3191 | SILVA | |
| BEIS01000013.64394 | SILVA | FMJQ01000982.152089 | SILVA | JBOX02019843.2199 | SILVA | |
| BEXD01004348.128701 | SILVA | FNCA01000018.91 | SILVA | JF509728.1 | SILVA | |
| BX537263.9971 | SILVA | FO904938.2555 | SILVA | JF682232.37353 | SILVA | |
| CAAE01002562.63 | SILVA | FR825014.217 | SILVA | JF773148.1 | SILVA | |
| CABD030100648.615 | SILVA | GBDB01007262.602 | SILVA | JHOM02002763.32724 | SILVA | |
| CABG01000622.26 | SILVA | GBFQ01044321.26 | SILVA | JHUJ02006182.6011 | SILVA | |
| CBLO020000003.1308 | SILVA | GBGO01022746.1 | SILVA | JMDP01000383.3234 | SILVA | |
| CBXY010018041.5 | SILVA | GBHT01005161.209 | SILVA | JMFW02008510.5055 | SILVA | |
| JN635052.1 | SILVA | LADJ01033521.436816 | SILVA | NIUS010588715.2730 | SILVA | |
| JN939989.1 | SILVA | LC036567.1 | SILVA | NXFZ01000158.5688 | SILVA | |
| JNBR01001696.641 | SILVA | LCWJ01006497.6300 | SILVA | OMOY01075861.22 | SILVA | |
| JWKW01000011.3478 | SILVA | LGHO01004302.1701 | SILVA | ONZH01001300.24921 | SILVA | |
| JX481969.1 | SILVA | LGSE01000414.136 | SILVA | PDCF01019561.38 | SILVA | |
| JYDP01000288.4367 | SILVA | LHPF02000195.13164 | SILVA | PDMG02031236.1 | SILVA | |
| JYDT01000267.4714 | SILVA | LHQN01013586.29690 | SILVA | PEHR01048385.9415 | SILVA | |
| JYDW01000396.7330 | SILVA | LIRP01005293.3198 | SILVA | PEKD01006704.697 | SILVA | |
| JZLG01032474.88481 | SILVA | LJIJ01004448.1937 | SILVA | PEKY01000621.8962 | SILVA | |
| KC177300.1 | SILVA | LKEX01010032.9789 | SILVA | PGGR01036000.413 | SILVA | |
| KC425175.1 | SILVA | LKEY01029519.591 | SILVA | PGRV01000004.450055 | SILVA | |
| KC820794.1 | SILVA | LKFA01014869.1945 | SILVA | PGUA01000738.13345 | SILVA | |
| KF160871.3777 | SILVA | LLKC01012342.23959 | SILVA | PKSA01001617.167786 | SILVA | |
| KF875699.1 | SILVA | LMYF01009029.3575 | SILVA | PPUY01000034.1402241 | SILVA | |
| KJ461298.1 | SILVA | LN609412.30867 | SILVA | QANH01000110.501419 | SILVA | |
| KJ668057.1 | SILVA | LNZE02000064.3834 | SILVA | QBEX01001846.1012 | SILVA | |
| KJ774646.1 | SILVA | LR026971.15459 | SILVA | QGTJ01000023.1 | SILVA | |
| KJ774723.1 | SILVA | LT607756.796726 | SILVA | QMEQ01000092.19563 | SILVA | |
| KJ774754.1 | SILVA | LT629751.607966 | SILVA | QMES01000033.440090 | SILVA | |
| KJ774770.1 | SILVA | LVWQ01135728.2 | SILVA | QOIP01000013.4473789 | SILVA | |
| KP098372.1 | SILVA | M10932.164 | SILVA | QPFT01000363.1244 | SILVA | |
| KP419313.1 | SILVA | M59137.1 | SILVA | QPFV01003199.1978 | SILVA | |
| KP760121.1 | SILVA | M84229.1 | SILVA | QRFA01001312.1966 | SILVA | |
| KP760157.1 | SILVA | M91180.1 | SILVA | QUVS01000310.4970 | SILVA | |
| KP761311.1 | SILVA | MCFE01000169.143269 | SILVA | RCCI01000012.1 | SILVA | |
| KT343379.1 | SILVA | MCFL01000080.72090 | SILVA | U06476.1 | SILVA | |
| KT718779.1 | SILVA | MCGO01000275.3049 | SILVA | U15188.1 | SILVA | |
| KU043523.1 | SILVA | MF164266.1581 | SILVA | U91490.1 | SILVA | |
| KU672728.1 | SILVA | MH400902.1 | SILVA | ULFR01003570.2513 | SILVA | |
| **Accession No.** | **Database** | **Accession No.** | **Database** | **Accession No.** | **Database** | |
| KX061879.3672 | SILVA | MH423481.1 | SILVA | URS0000894B85 | RNAcentral | |
| KX061884.3484 | SILVA | MKHE01002368.8761 | SILVA | URS00009CAF68 | RNAcentral | |
| KX573102.1 | SILVA | MKKW01020617.14535 | SILVA | URS00021D0F3A | RNAcentral | |
| KY115616.1 | SILVA | MNAD01000754.611 | SILVA | X02995.1030 | SILVA | |
| KY462829.1 | SILVA | MTTC01000578.15987 | SILVA | X07801.96 | SILVA | |
| L04153.3 | SILVA | MTZO01011598.1594 | SILVA | X65537.1 | SILVA | |
| L04154.3 | SILVA | MUGM01000717.1298 | SILVA | X85132.1 | SILVA | |
| L10828.1 | SILVA | MVBO01000365.753 | SILVA | Y11560.1 | SILVA | |
| L76355.1 | SILVA | MWPT01000122.43634 | SILVA | Y17712.1 | SILVA | |
| LADI01009217.256 | SILVA | MZGG01000088.7207 | SILVA |  | |  |
