# Supplementary figures and images for: Bioinformatics approaches for classification and investigation of the evolution of the Na/K-ATPase alpha-subunit
Source: BMC Ecol Evol. 2022 Oct 26;22:122. doi: 10.1186/s12862-022-02071-0 (PMC9609216; doi:10.1186/s12862-022-02071-0)

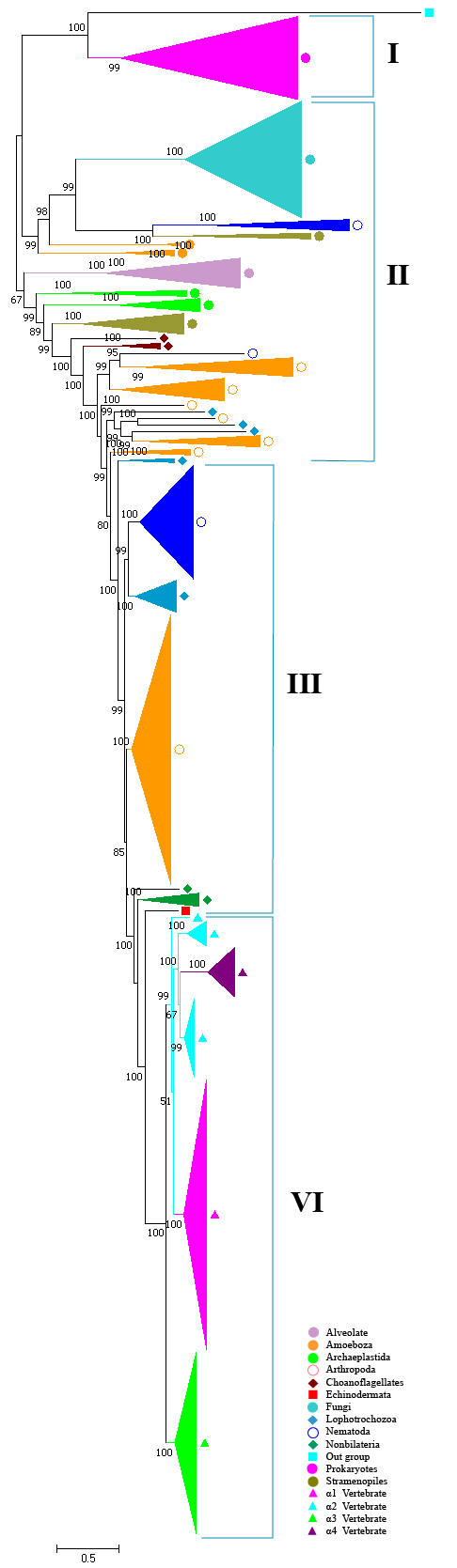

Supplement: Supplementary file 1 — Additional file 1. Supplementary figures and tables. [file 12862_2022_2071_MOESM1_ESM.zip › 1-Additional file 1 Fig. S1.png]

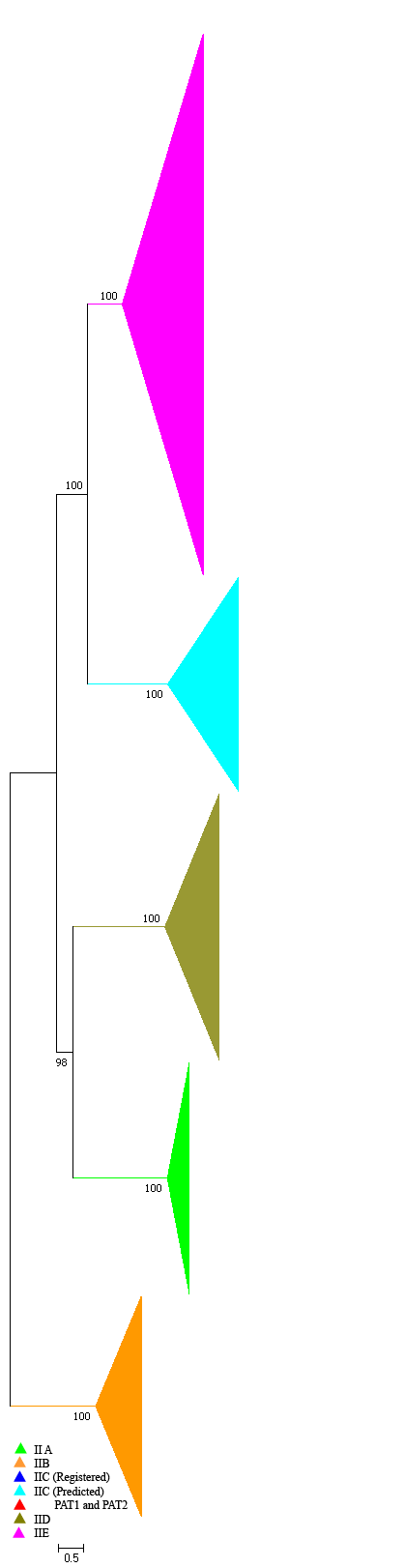

Supplement: Supplementary file 1 — Additional file 1. Supplementary figures and tables. [file 12862_2022_2071_MOESM1_ESM.zip › Additional file 1 Fig. S2.png]

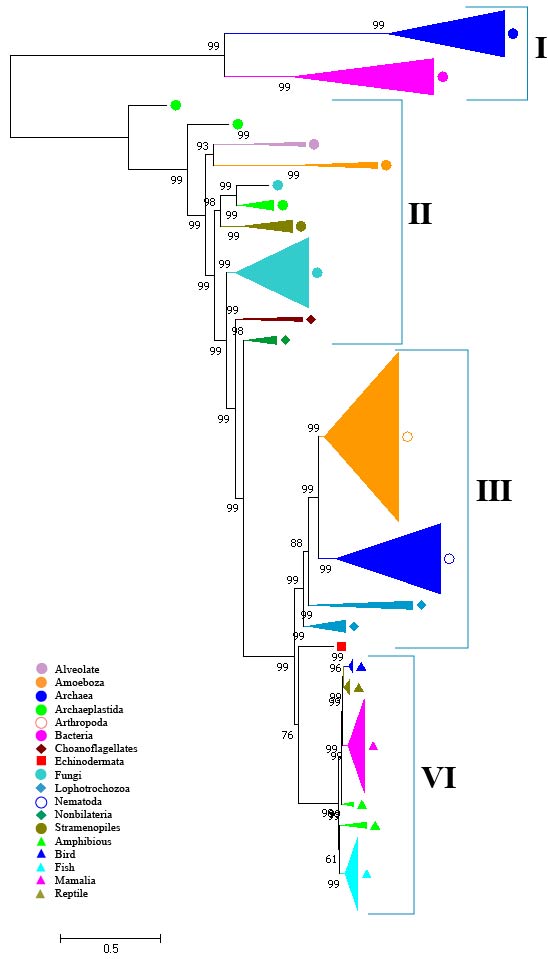

Supplement: Supplementary file 1 — Additional file 1. Supplementary figures and tables. [file 12862_2022_2071_MOESM1_ESM.zip › Additional file 1 Fig. S3.jpg]

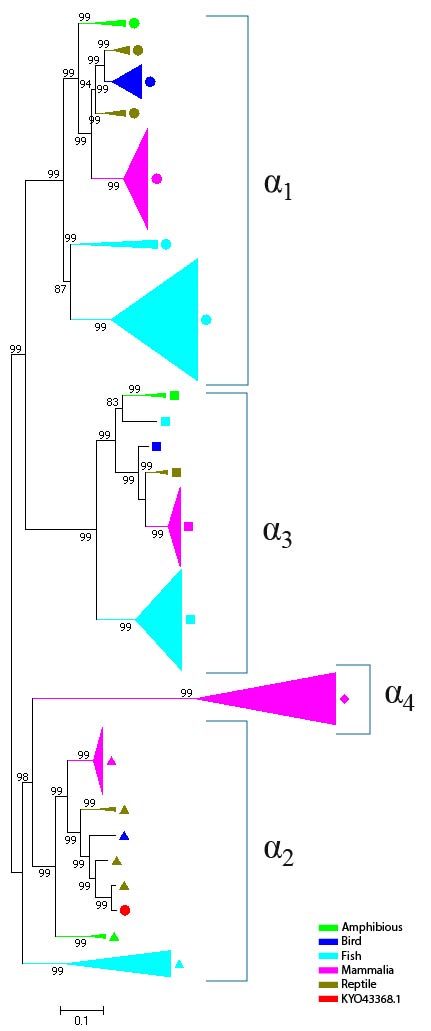

Supplement: Supplementary file 1 — Additional file 1. Supplementary figures and tables. [file 12862_2022_2071_MOESM1_ESM.zip › Additional file 1 Fig. S4.jpg]
